# Systematic construction and validation of an immune prognostic model for lung adenocarcinoma

**DOI:** 10.1111/jcmm.14719

**Published:** 2019-11-28

**Authors:** Chenghan Luo, Mengyuan Lei, Yixia Zhang, Qian Zhang, Lifeng Li, Jingyao Lian, Shasha Liu, Liping Wang, Guofu Pi, Yi Zhang

**Affiliations:** ^1^ Biotherapy Center The First Affiliated Hospital of Zhengzhou University Zhengzhou China; ^2^ Orthopedics Department The First Affiliated Hospital of Zhengzhou University Zhengzhou China; ^3^ Physical Examination Center The First Affiliated Hospital of Zhengzhou University Zhengzhou China; ^4^ Neonatal Intensive Care Unit The First Affiliated Hospital of Zhengzhou University Zhengzhou China; ^5^ Cancer Center The First Affiliated Hospital of Zhengzhou University Zhengzhou China; ^6^ Henan Key Laboratory for Tumor Immunology and Biotherapy Zhengzhou China

**Keywords:** biomarkers, immune prognostic model, immunology, lung adenocarcinoma, overall survival

## Abstract

Lung adenocarcinoma (LUAD), the most common non‐small‐cell lung cancer, is characterized by a dense lymphocytic infiltrate, which indicates that the immune system plays an active role in the development and growth of this cancer. However, no investigations to date have proposed robust models for predicting survival outcome for patients with LUAD in terms of tumour immunology. A total of 761 LUAD patients were included in this study, in which the database of The Cancer Genome Atlas (TCGA) was utilized for discovery, and the Gene Expression Omnibus (GEO) database was utilized for validation. Bioinformatics analysis and R language tools were utilized to construct an immune prognostic model and annotate biological functions. Lung adenocarcinoma showed a weakened immune phenotype compared with adjacent normal tissues. Immune‐related gene sets were profiled, an immune prognostic model based on 2 immune genes (ANLN and F2) was developed with the TCGA database to distinguish cases as having a low or high risk of unfavourable prognosis, and the model was verified with the GEO database. The model was prognostically significant in stratified cohorts, including stage I‐II, stage III‐IV and epidermal growth factor receptor (EGFR) mutant subsets, and was considered to be an independent prognostic factor for LUAD. Furthermore, the low‐ and high‐risk groups showed marked differences in tumour‐infiltrating leucocytes, tumour mutation burden, aneuploidy and PD‐L1 expression. In conclusion, an immune prognostic model was proposed for LUAD that is capable of independently identifying patients at high risk for poor survival, suggesting a relationship between local immune status and prognosis.

## 1 INTRODUCTION

Lung cancer is one of the most common causes of cancer deaths worldwide.^1^, ^2^ For treatment purposes, lung cancers are categorized as either non‐small‐cell lung cancer (NSCLC) or small cell lung cancer. Non‐small‐cell lung cancer comprises approximately 85% of all lung cancers, with lung adenocarcinoma (LUAD) being the most frequently diagnosed histological subtype of NSCLC, followed by squamous cell carcinoma. The high morbidity rate of lung cancer is due to tobacco smoking, genetic alteration, outdoor pollution, indoor air pollution and other factors.^3^, ^4^, ^5^ Because LUAD is prone to metastasis at early stages, the prognosis for LUAD patients is usually poor, with an average 5‐year survival rate of less than 20%.^6^ Although recent progress in targeted therapy and molecular pathology has enhanced clinical therapy, the 5‐year overall survival (OS) rate of LUAD patients remains low.^7^, ^8^ Hence, further understanding of the molecular mechanisms underlying tumorigenesis and progression in LUAD may enhance the overall prognosis and treatment of this disease.

The immune evasion strategy utilized by tumour cells for evading host immune responses and maximizing the possibility for continued growth is a hallmark of cancer.^9^ Immune disorders in cancer are considered to promote oncogenesis and development.^10^ Immune responses stimulated by cancer antigens, which should trigger the elimination of cancer cells, can be suppressed to offer an appropriate microenvironment for cancer growth.^10^ Enormous efforts have been directed at understanding the interaction between the immune system and tumours, and significant success has been achieved in the form of tumour immune therapy to advance tumour treatment; however, this approach can be applied in only a subset of patients, as other patients either fail to respond or are unsuitable.^11^, ^12^

Cancer immunotherapy has attracted considerable attention in recent years because the development of immune checkpoint blockade therapy can achieve durable, long‐term responses in refractory malignancies, including lung cancers.^13^, ^14^ The clinical development of immune checkpoint inhibitors in NSCLC began in patients being treated for metastatic diseases.^15^, ^16^ At present, three immune checkpoint inhibitors have received FDA approval as second‐line NSCLC treatments (atezolizumab, pembrolizumab and nivolumab).^17^ These agents were also authorized in the European Union. Atezolizumab is an immune checkpoint inhibitor that targets programmed cell death ligand 1 (PD‐L1), while pembrolizumab and nivolumab target programmed cell death 1 (PD‐1).^18^ The immune response in the tumour microenvironment is now recognized as a significant factor that determines tumour aggressiveness and progression, as well as responsiveness to immune‐modulating agents. The densities and types of tumour‐infiltrating immune cells, as well as their expression of cytokines and immune genes, have been extensively studied as prognostic biomarkers in lung cancers.^19^, ^20^, ^21^ Certain histopathologic patterns, such as intratumoral infiltration by cytotoxic lymphocytes, have also been linked to better responses in LUAD. Nonetheless, the molecular features illustrating interactions between the immune system and cancer remain to be fully explored in terms of their prognostic potential in LUAD.

In this study, multiple gene expression datasets were combined to develop and validate an individualized immune prognostic model for LUAD on the basis of immune genes. To leverage the complementary value of clinical and molecular features, the immune prognostic model was combined with clinical features to build a composite prognostic nomogram, enabling improved estimation of LUAD prognosis.

## 2 MATERIALS AND METHODS

### 2.1 Gene expression data and clinical information

Level 3 raw counts of the RNA‐seq data, tumour mutation burden, aneuploidy scores and corresponding clinical data from a total of 535 patients with LUAD were acquired from the data portal of The Cancer Genome Atlas (TCGA) as of 16 January 2019. Clinical parameters, including gender, age and pathological stage, were also evaluated. Transcriptome profiling data of 226 patients with LUAD in the http://www.ncbi.nlm.nih.gov/geo/query/acc.cgi?acc=GSE31210 dataset from the GEO database were used for validation.^22^

### 2.2 Identification of differentially expressed mRNAs (DEGs) in LUAD and adjacent normal tissues

To identify DEGs between adjacent normal tissues and LUAD, we performed differential expression analysis using the edgeR package (version: 3.26.5).^23^ The thresholds for screening DEGs were |log_2_ FC (fold‐change) | > 2 and *P* < .01.

### 2.3 Gene‐set enrichment analysis

Gene‐set enrichment analysis (GSEA) was conducted to explore whether immune functions, and the corresponding immune genes were significantly different between adjacent normal tissues and LUAD samples.^24^

### 2.4 Immune prognostic model construction and validation

First, we normalized the RNA‐seq expression value of the immune genes using log_2_ transformation. Then, we performed univariate Cox regression analysis to determine the relationship between patient survival and immune gene expression. Immune genes with *P* < .01 were selected for least absolute shrinkage and selection operator (LASSO) Cox regression analysis. Next, using multivariate Cox regression analysis based on the immune genes obtained from LASSO Cox regression analysis, in which we required selected genes to appear more than 990 times out of 1000 repetitions in total, we built a formula to predict the risk score of each patient. X‐tile software (version: 3.6.1) was used to identify the optimum cut‐off value for dividing patients into low‐ and high‐risk groups according to the highest χ^2^ value defined in the Mantel‐Cox test.^25^, ^26^ Afterwards, we conducted a log rank test to determine the difference between the low‐ and high‐risk groups. We plotted a Kaplan‐Meier OS curve for both groups and calculated the hazard rate (HR). Additionally, we conducted Cox multivariate analysis to test whether the immune prognostic model was independent of clinical characteristics, including age, gender and pathologic stage, and we measured prognostic ability by calculating the area under the curve (AUC) of the receiver operating characteristic (ROC) curve.

### 2.5 The evaluation of immune cells in LUAD

CIBERSORT, a deconvolution algorithm based on normalized gene expression profiles, can quantify the immune cell composition and has greatly expanded the potential of the genomic database.^27^ Because CIBERSORT is superior to other methods, it has received increasing attention and has been successfully used to assess the composition of immune cells in liver and breast cancer.^28^, ^29^ We utilized CIBERSORT to assess the composition of 22 immune cells in the TCGA and GEO LUAD cohorts.

### 2.6 Functional enrichment analysis

To explore the underlying biological processes and pathways of the immune genes, we utilized the Database for Annotation, Visualization, and Integrated Discovery (DAVID; version: 6.7) and KO‐Based Annotation System (KOBAS; version: 3.0) to perform functional enrichment analysis, focusing on significantly enriched (*P* < .05) Kyoto Encyclopedia of Genes and Genomes (KEGG) pathways and Gene Ontology (GO) biological processes.^30^, ^31^

### 2.7 Nomogram development and validation for prognostic risk prediction

To offer quantitative methods to clinicians to predict the 1‐, 3‐, and 5‐year survival probabilities of LUAD patients, a nomogram integrating a variety of clinical risk factors and the immune prognostic model was assembled. Then, validations, including discrimination and calibration, were conducted. The discrimination of the nomogram was calculated by the C‐index using a bootstrap method with 1000 resamples. The concordance index (C‐index) value was between 0.5‐1.0, where 1.0 suggests a perfect capacity for correctly distinguishing outcomes with the model, and 0.5 suggests random chance. We graphically evaluated the nomogram calibration curve by plotting the prediction probability of the nomogram against the rates observed. Overlap with the reference line showed that the agreement of the model was perfect.

## 3 RESULTS

### 3.1 Differentially expressed mRNAs in patients with LUAD

**Figure 1:**
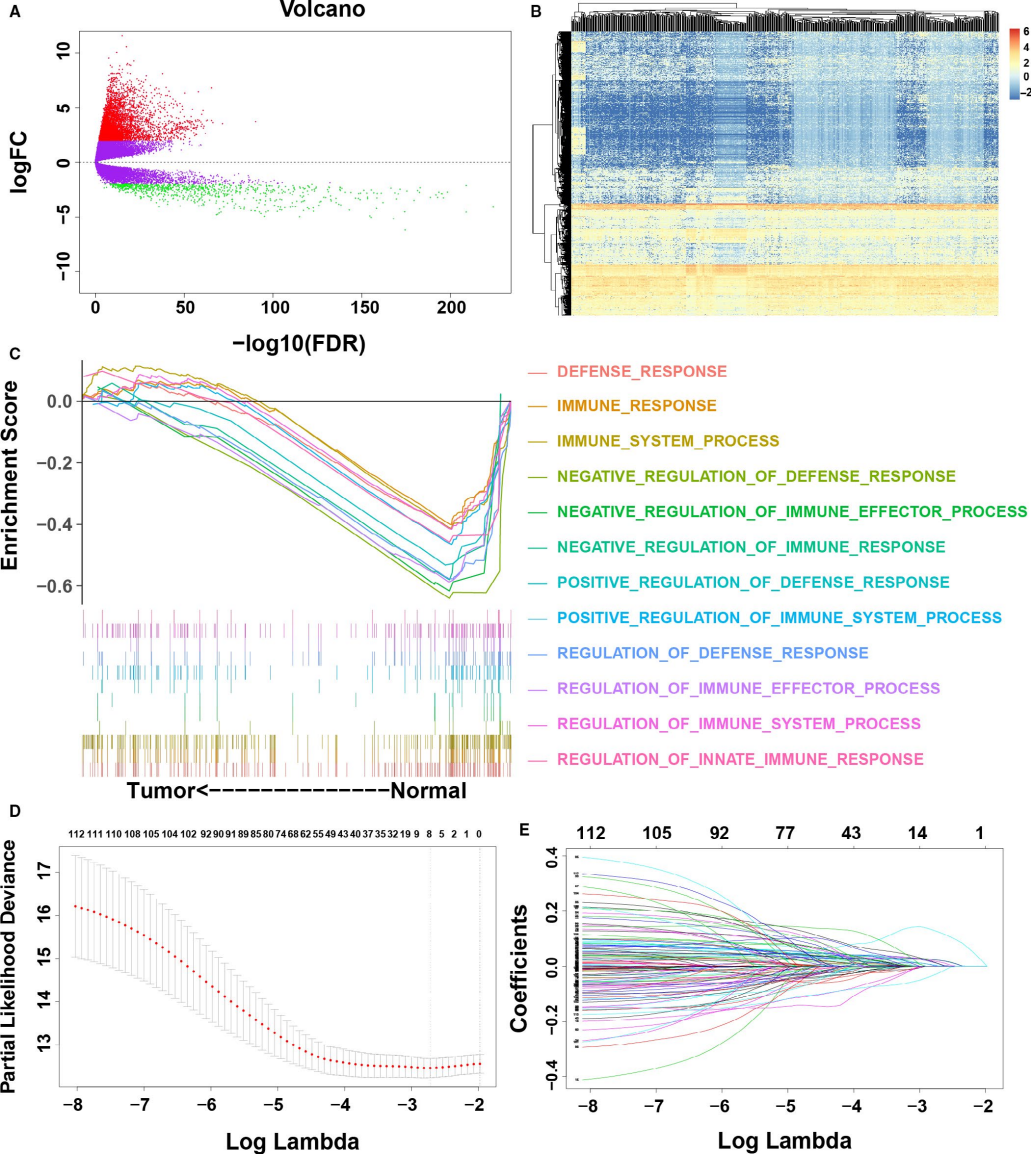
Identification of prognostic immune genes. A, Volcano plot showing the differentially expressed genes (DEGs) between lung adenocarcinoma (LUAD) and adjacent normal tissues. B, Heatmap of DEGs. Rows represent DEGs, and columns represent samples. C, Gene‐set enrichment analysis of DEGs. D, Ten‐time cross‐validation for tuning parameter selection in the least absolute shrinkage and selection operator (LASSO) model. E, LASSO coefficient profiles of the 261 differentially expressed immune genes

Analyses of mRNA expression profiles between adjacent normal tissues and LUAD tissues identified 5774 DEGs in total (Figure 1A). Compared with normal lung samples, 4962 mRNAs were down‐regulated, and 812 were up‐regulated in LUAD samples (Figure 1B; Table S1).

### 3.2 A weakened immune phenotype in LUAD

Considering the different immune status between normal lung samples and LUAD samples, we initially utilized RNA‐seq data for 5774 DEGs from a total of 535 LUAD patients from the TCGA database to identify different immune biological processes and genes. Gene‐set enrichment analysis analysis indicated that LUAD was significantly negatively related to 12 immune biological processes, indicating a weakened local immune response in the LUAD microenvironment (Figure 1C; Table S2). Among the 5774 DEGs, 353 immune genes were enriched in those 12 biological immune processes (Table S3). To validate the relationship between LUAD and immune biological processes, we extracted 353 immune genes for subsequent survival analysis.

### 3.3 Establishment and evaluation of the immune prognostic model with the training dataset

**Figure 2:**
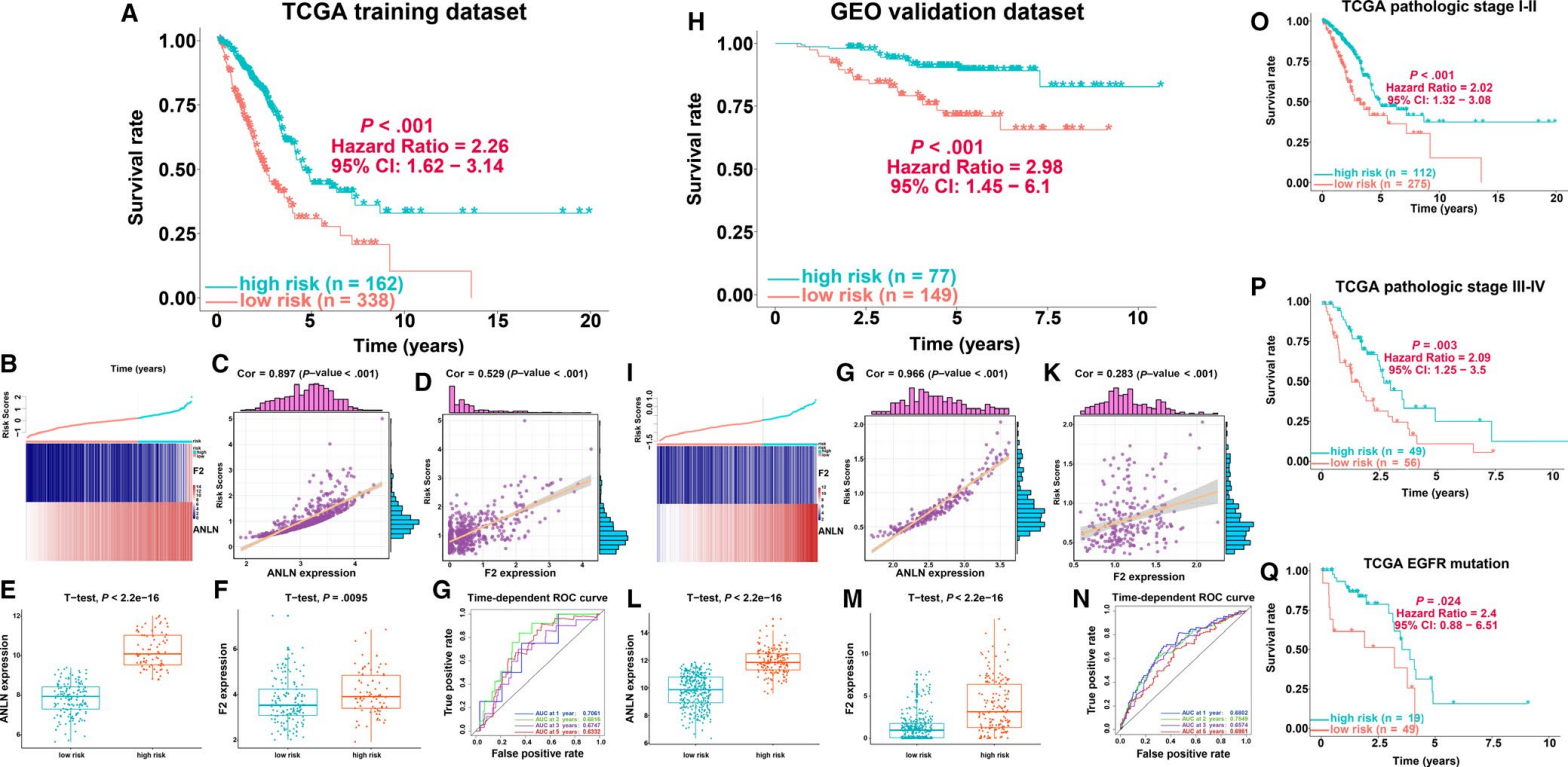
Survival analyses of lung adenocarcinoma (LUAD) patients in the TCGA training and GEO validation datasets. A, Kaplan‐Meier analysis of the immune prognostic model in the TCGA training cohort. B, The distribution of the risk score of patients with LUAD and gene expression of ANLN and F2 in the TCGA training cohort. C, Correlation analysis of risk scores and gene expression of ANLN in the TCGA training cohort. D, Correlation analysis of risk scores and gene expression of F2 in the TCGA training cohort. E, Comparison of ANLN gene expression between the high‐ and low‐risk groups in the TCGA training cohort. F, Comparison of gene expression of F2 between the high‐ and low‐risk groups in the TCGA training cohort. G, The evaluation of the immune prognostic model in the TCGA training cohort. H, Kaplan‐Meier analysis of the immune prognostic model in the GEO validation cohort. I, The distribution of the risk scores of patients with LUAD and gene expression of ANLN and F2 in the GEO validation cohort. J, Correlation analysis of risk scores and gene expression of ANLN in the GEO validation cohort. K, Correlation analysis of risk scores and gene expression of F2 in the GEO validation cohort. L, Comparison of ANLN gene expression between the high‐ and low‐risk groups in the GEO validation cohort. M, Comparison of gene expression of F2 between the high‐ and low‐risk groups in the GEO validation cohort. N, The evaluation of the immune prognostic model in the GEO validation cohort. O, Kaplan‐Meier survival analysis of stage I‐II cases in the TCGA training cohort. P, Kaplan‐Meier survival analysis of stage III‐IV cases in the TCGA training cohort. Q, Kaplan‐Meier survival analysis of epidermal growth factor receptor (EGFR) mutant cases in the TCGA training cohort

To identify immune genes related to the survival of LUAD patients, univariate Cox regression analysis of 353 immune genes was performed. We selected a set of 113 immune genes at a 0.05 significance threshold (Table S4). The 113 immune genes were subjected to LASSO Cox regression analysis, and 2 immune genes were identified (Figure 1D,E). We then performed multivariate Cox regression analysis to establish an immune prognostic model for patients with LUAD on the basis of gene expression levels, as follows: risk value = (0.2518 × ANLN expression) + (0.0879 × F2 expression). The risk scores for patients were calculated, and patients were categorized as low risk or high risk according to the optimal cut‐off. Low‐risk patients had a longer OS than high‐risk patients (*P* < .001; HR = 2.26; 95% confidence interval [CI] = 1.62‐3.14; Figure 2A). The expression of the two immune genes and the distribution of risk scores for each patient were also analysed (Figure 2B). The gene expression levels of ANLN and F2 were significantly correlated with the risk scores, and these genes were significantly highly expressed in the high‐risk group in the TCGA LUAD cohorts (Figure 2C‐F). The ROC curves showed that the AUCs of the immune prognostic model at 1, 2, 3 and 5 years were 0.7061, 0.6816, 0.6747 and 0.6332, respectively (Figure 2G), indicating that the model has high sensitivity and specificity to predict the prognosis of LUAD patients. Recently, Shukla et al^32^ proposed a prognostic model including 4 genes (FRRS1, LINC00941, CD109 and RHOV) for OS prediction in LUAD patients. We calculated the C indexes to compare the prognostic values of their model and our immune model. The C‐index is the most commonly used performance measure for survival models; it ranges from 0.5 to 1 and is equal to the AUC.^33^ The higher the value of the C‐index, the better the predictability of the model. The C‐index of the immune prognostic model (0.6540) exceeded that of the previous model (0.6446), suggesting that our immune prognostic model had favourable efficacy for predicting both short‐ and long‐term prognosis.

### 3.4 Validation and evaluation of the immune prognostic model with the validation dataset

To confirm the robustness of the immune prognostic model, the validation dataset (n = 226) was utilized for further validation analysis. In the validation dataset, we classified all patients into high‐risk and low‐risk groups using the same equation based on the optimal cut‐off value. Consistent with the results of the training dataset, patients with low‐risk scores had markedly longer OS than high‐risk patients (*P* < .001; HR = 2.98; 95% CI = 1.45‐6.1; Figure 2H). Figure 2I shows the expression of the two immune genes and the distribution of risk scores for each patient in the validation dataset, demonstrating results similar to those of the training dataset. The expression levels of ANLN and F2 were significantly correlated with the risk scores, and these genes were significantly highly expressed in the high‐risk group in the GEO LUAD cohorts (Figure 2G‐M). Receiver operating characteristic analysis showed that the AUC of the immune prognostic model reached 0.6802, 0.7549, 0.6574 and 0.6981 at 1, 2, 3 and 5 years, respectively, suggesting that the proposed model performed well in predicting 1‐, 2‐, 3‐ and 5‐year OS with the validation dataset (Figure 2N).

### 3.5 Validation of the immune prognostic model in clinically significant subsets

Stage I‐II LUAD is considered early‐stage disease and may be cured by adjuvant radio/chemotherapy and surgery. On the other hand, stage III‐IV LUAD is considered advanced‐stage disease and is characterized by unfavourable outcome, even with full‐intensity multimodality therapy. Given the properties and clinical importance of early‐stage LUAD, the immune prognostic model for patients with stage I‐II LUAD was evaluated in the TCGA dataset. Low‐risk patients had significantly favourable OS compared to the high‐risk patients (*P* < .001; HR = 2.02; 95% CI = 1.32‐3.08) with stage I‐II LUAD (Figure 2O). We observed similar results in advanced‐stage patients. Low‐risk patients had a favourable prognosis compared with high‐risk patients (*P* = .003; HR = 2.09; 95% CI = 1.25‐3.5; Figure 2P). Consistent with the above analyses, our proposed immune prognostic model was shown to be an independent factor in the survival of patients with LUAD. Because therapeutic epidermal growth factor receptor (EGFR) mutation frequency and the TKI agent response rate are high in LUAD in East Asia, immune prognostic model performance was analysed in patients with EGFR mutant status in the TCGA dataset. Patients in the low‐risk group had significantly favourable OS compared with high‐risk patients (*P* = .024; HR = 2.4; 95% CI = 0.88‐6.51) in EGFR mutant LUAD who may receive EGFR‐TKI as adjuvant therapy (Figure 2Q).

### 3.6 Tumour immune landscape and genomic association

**Figure 3:**
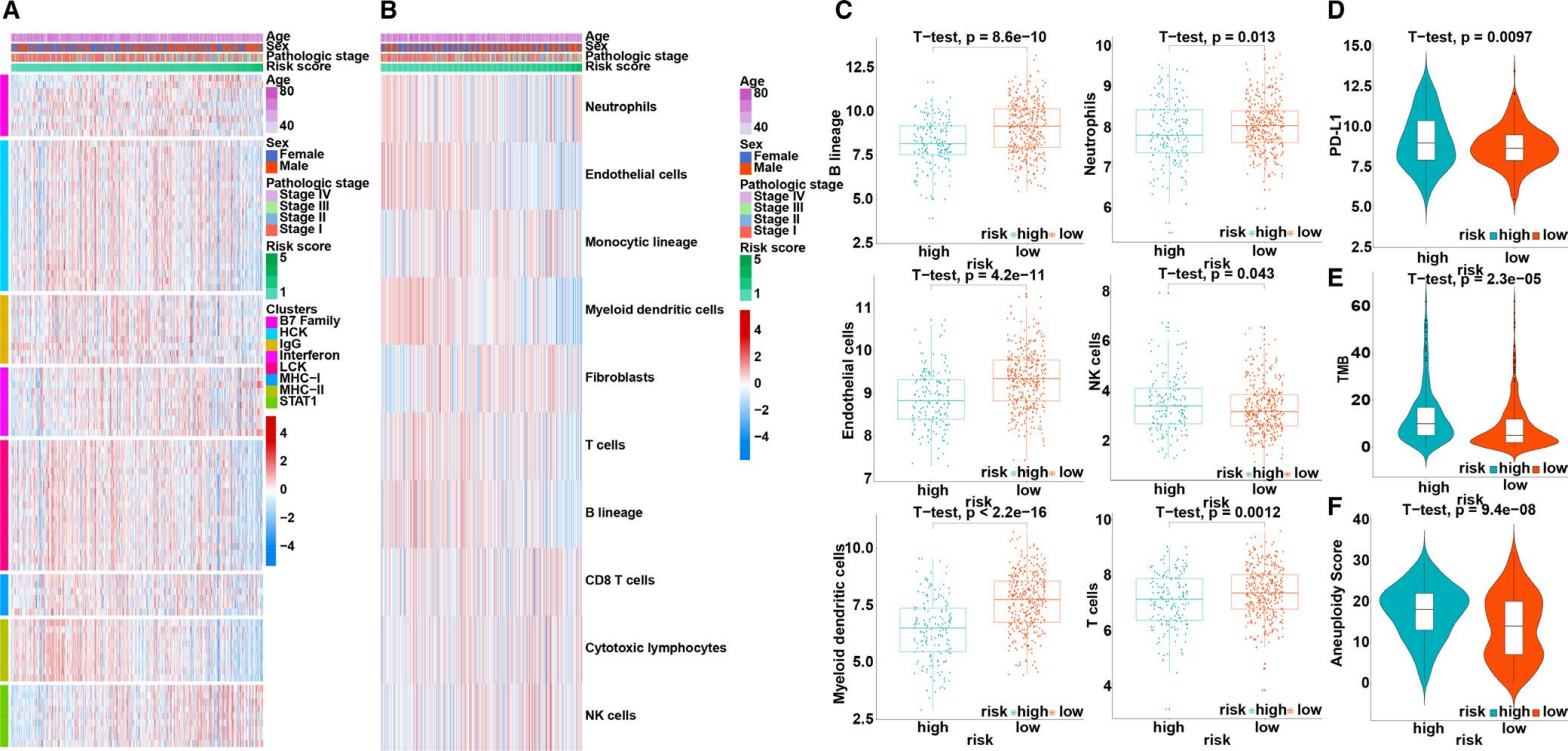
Immune profile related to the immune prognostic model. A, Association between risk score and immune response. B, Associations between risk score and immune cell populations. C, Relative proportion of immune cell expression in the high‐ and low‐risk groups. D, The distribution of programmed cell death ligand 1 (PD‐L1) expression in the high‐ and low‐risk groups. E, The distribution of tumour mutation burden in the high‐ and low‐risk groups. F, The distribution of aneuploidy scores in the high‐ and low‐risk groups

To further explore the correlation between the immune prognostic model and the immune response, B7 family‐related metagenes, as well as seven previously studied inflammatory genes, were taken into consideration.^34^, ^35^ The B7 family, interferon and STAT1 were found to be positively correlated with the risk score, while HCK, IgG, LCK, MHC‐I and MHC‐II were negatively correlated with the risk score (Figure 3A). In addition, the Microenvironment Cell Populations‐counter approach was used to assess the relationship between immune cell populations and risk score.^36^ We found that the immune landscape was obviously different between low‐ and high‐risk patients (Figure 3B). Patients in the low‐risk group had a significantly higher proportion of B lineage cells, endothelial cells, myeloid dendritic cells, neutrophils and T cells, as well as a markedly lower proportion of NK cells, compared with the high‐risk group (Figure 3C).

PD‐L1 (CD274) expression is a biomarker for selecting NSCLC patients for pembrolizumab treatment.^37^, ^38^ Therefore, PD‐L1 expression was investigated in patients stratified by the immune prognostic model. High‐risk patients had markedly elevated PD‐L1 expression compared with low‐risk patients (*P* < .001) and may respond better and have better outcome when receiving pembrolizumab (Figure 3D).

Tumour mutation burden (TMB), meaning all the somatic missense mutations in a baseline tumour sample, serves as a predictor for predicting the efficacy of nivolumab.^39^ Patients with a high TMB have a higher response rate and favourable progression‐free survival when receiving nivolumab treatment.^39^ The TMB of patients stratified by the immune prognostic model was therefore investigated. The *t* test demonstrated a significant difference between the low‐risk and high‐risk groups (*P* < .0001; Figure 3E).

Aneuploidy, also known as somatic cell copy number alteration (SCNA), has been proposed to drive oncogenesis and is widely found in human cancers. Aneuploidy is associated with reduced cytotoxic immune infiltration and tumour cell proliferation. High SCNA levels in cancers are associated with unfavourable survival in melanoma patients, and cancer SCNA scores are good predictors of survival after immunotherapy. Therefore, the aneuploidy of the patients with LUAD stratified by the immune prognostic model was investigated. High‐risk patients had markedly higher aneuploidy scores than low‐risk patients (*P* < .001; Figure 3F).

### 3.7 The relationships of ANLN and F2 with immune cells

**Figure 4:**
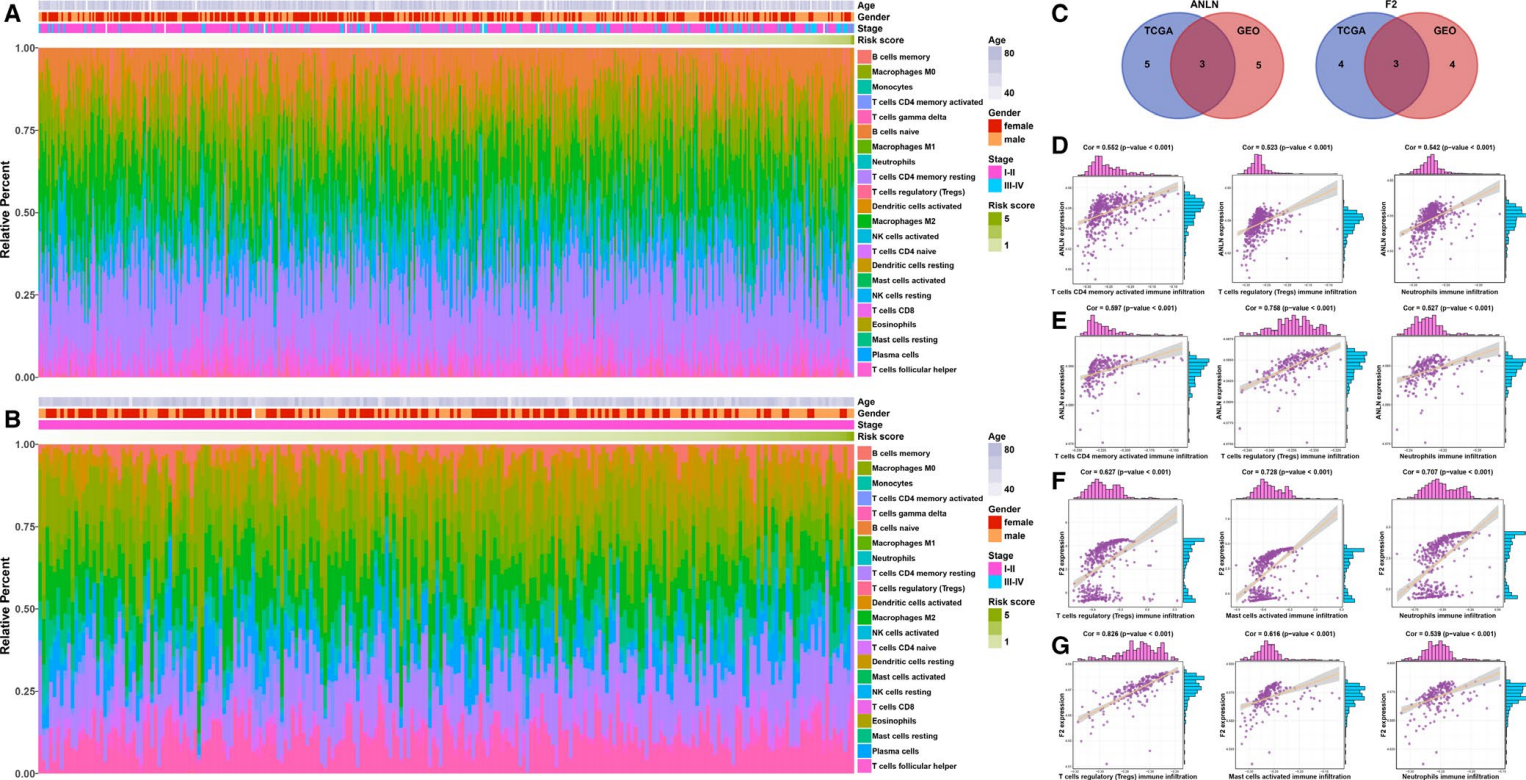
Relationship between immune cells and the immune genes. A, The landscape of immune cell infiltration of LUAD patients in the TCGA training cohort. B, The landscape of immune cell infiltration of LUAD patients in the GEO validation cohort. C, The left panel shows the intersection of immune cells that were significantly associated with the gene expression of ANLN from the TGCA and GEO datasets, respectively. The right panel shows the intersection of immune cells that were significantly associated with gene expression of F2 from the TGCA and GEO datasets, respectively. D, Correlation analyses between three immune cells and gene expression of ANLN in the TCGA training cohort. E, Correlation analyses between three immune cells and gene expression of F2 in the TCGA training cohort. F, Correlation analyses between three immune cells and gene expression of ANLN in the GEO validation cohort. G, Correlation analyses between three immune cells and gene expression of F2 in the GEO validation cohort

To explore which immune cells are associated with ANLN and F2, correlation analyses between immune cells and ANLN or F2 were performed. First, CIBERSORT was applied to assess the relative proportions of 22 immune cells, presenting a comprehensive immune cell landscape of LUAD.^27^ Figure 4A,B shows the composition of 22 immune cells in LUAD in the TCGA and GEO LUAD cohorts. Then, correlation analyses between immune cells and ANLN or F2 were conducted. The immune cells with correlation coefficients >.4 and *P* values <.05 in both the TCGA and GEO LUAD cohorts were considered to be associated with the investigated genes (Figure 4C). As a result, ANLN was associated with three immune cells (T cells CD4 memory activated, T cells regulatory (Tregs) and neutrophils; Figure 4D,E), and F2 was associated with three immune cells (Tregs, mast cells activated and neutrophils; Figure 4F,G).

### 3.8 Identification of immune prognostic model‐related biological processes and pathways

**Figure 5:**
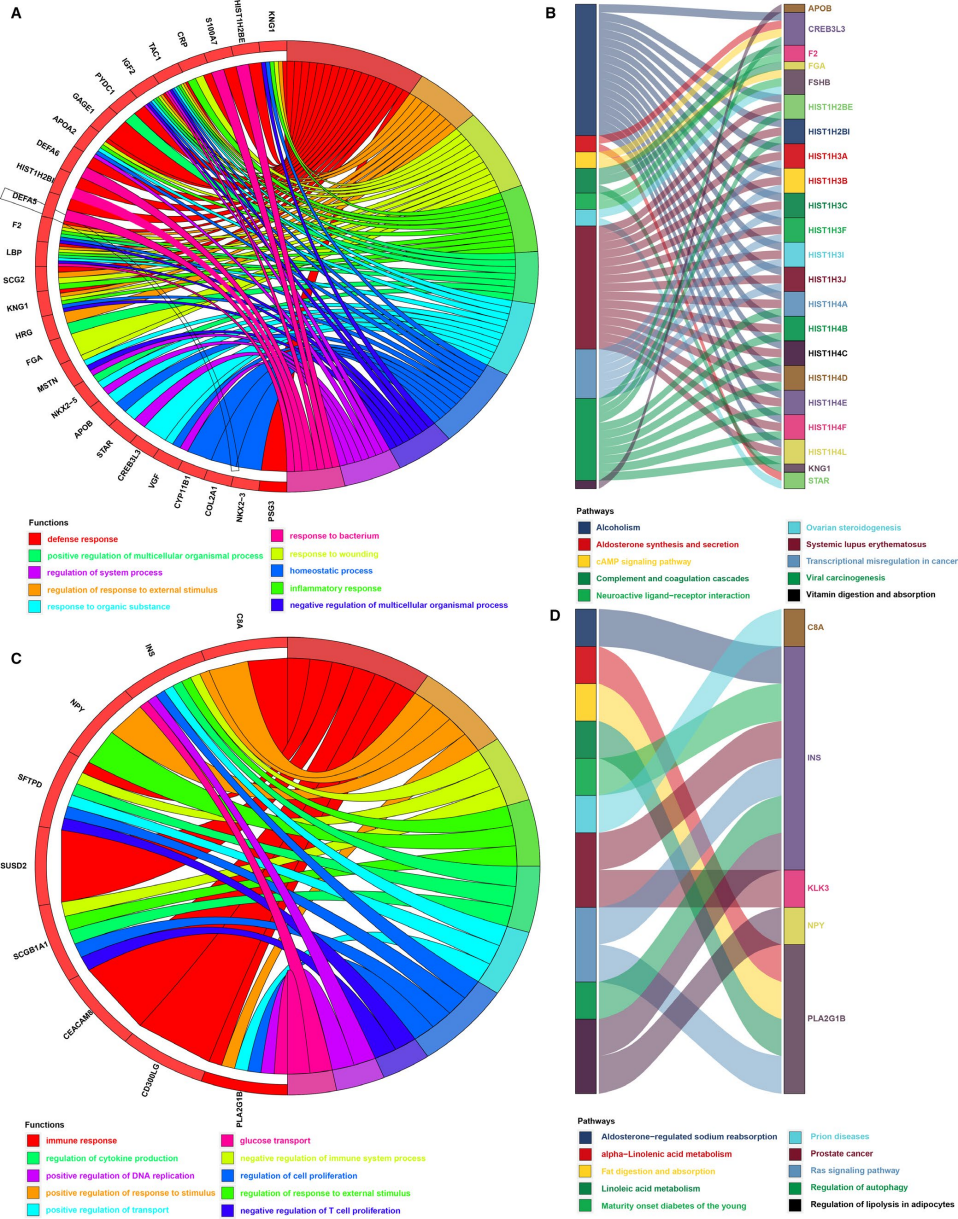
Enrichment analysis of the immune prognostic model. A, Functional enrichment analysis of the highly expressed genes in high‐risk groups (showing the top 10 biological processes). B, Pathway enrichment analysis of the highly expressed genes in high‐risk groups (showing the top 10 enriched pathways). C, Functional enrichment analysis of the highly expressed genes in low‐risk groups (showing the top 10 biological processes). D, Pathway enrichment analysis of the highly expressed genes in low‐risk groups (showing the top 10 enriched pathways)

To identify pathways underlying the immune prognostic model, differential expression analysis was performed on 353 immune genes between the low‐ and high‐risk groups. Forty‐five immune genes were highly expressed (|log_2_ FC | > 2 and *P* < .01) in the high‐risk groups, and 11 immune genes were highly expressed in the low‐risk groups (|log_2_ FC | > 2 and *P* < .01). Then, we performed functional enrichment analysis with the DAVID and KOBAS bioinformatics resources to explore the underlying biological function of these highly expressed genes in the high‐ and low‐risk groups, respectively, revealing 143 enriched biological processes (Table S5) and 12 enriched pathways (Table S6) in the high‐risk groups and 33 enriched biological processes (Table S7) and 39 enriched pathways (Table S8) in the low‐risk groups (*P* < .05). The top three biological processes were defence response, regulation of response to external stimulus and response to wounding in the high‐risk groups (Figure 5A; Table S5) and immune response, positive regulation of response to stimulus, and negative regulation of immune system process in the low‐risk groups (Figure 5C; Table S7). The top three pathways were alcoholism, systemic lupus erythematosus and viral carcinogenesis in the high‐risk groups (Figure 5B; Table S6) and regulation of lipolysis in adipocytes, prostate cancer and Ras signalling pathway in the low‐risk groups (Figure 5D; Table S8).

### 3.9 Relationship between the immune prognostic model and clinical parameters or patient outcome

**Figure 6:**
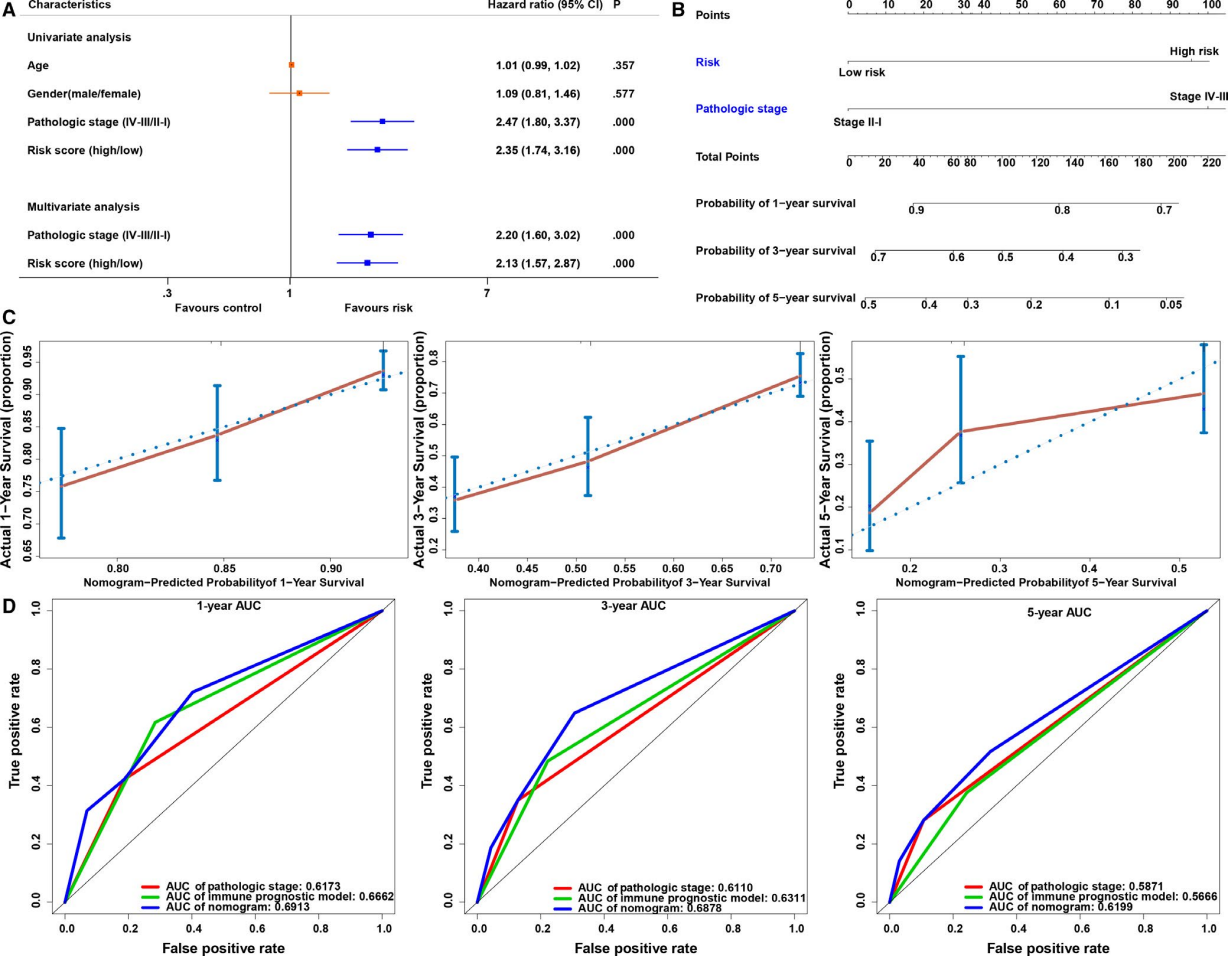
Nomogram for predicting the probability of overall survival (OS) in lung adenocarcinoma (LUAD) patients. A, Univariate and multivariate Cox analysis of clinical characteristics and the immune prognostic model. B, A nomogram for predicting OS in patients with LUAD. C, Calibration plot of the nomogram for the probability of OS at 1, 3 and 5 y. D, Comparison of time‐dependent ROC curves among pathologic stage, immune prognostic model and the nomogram

To further understand the relationship between the immune prognostic model and other clinical data, such as age, gender and pathologic stage, we performed univariate and multivariate Cox analyses, which revealed that the immune prognostic model serves as an independent factor for predicting the prognosis of LUAD patients (Figure 6A).

### 3.10 Development and validation of a predictive nomogram

To facilitate the use of our immune prognostic model, we established a nomogram for predicting LUAD prognosis based on the multivariate Cox analysis in the TCGA database. Our nomogram contained two predictive factors: pathologic stage and immune prognostic model (Figure 6B). Each factor was assigned a score in accordance with the multivariate analysis. We derived the nomogram score in total from the sum of the individual scores of all predictive factors. A high total score was predictive of low 1‐, 3‐ and 5‐year survival; however, a low total score showed the opposite pattern. The C‐index of the established nomogram for OS prediction was 0.6621 (95% CI = 0.6182‐0.7059), and a calibration plot demonstrated good agreement compared with the ideal model, indicating that our proposed nomogram has stability for predicting LUAD patient prognosis in clinical practice (Figure 6C). In addition, prediction accuracy was compared among the immune prognostic model, pathologic stage and the nomogram. The discrimination performance of the nomogram was superior to that of the immune prognostic model or pathologic stage (Table 1). The AUC of the nomogram was also the largest (Figure 6D). These findings show that compared with the individual prognostic factors, the nomogram is the optimal model for predicting the survival of LUAD patients.

**Table 1:** Comparison of predictive accuracy of the pathologic stage, immune prognostic model and nomogram

Models	C‐index (95%, CI)	*P*‐value
Pathologic stage	0.6021 (0.5626‐0.6415)	–
Immune prognostic model	0.6228 (0.5822‐0.6634)	–
Nomogram	0.6620 (0.6182‐0.7059)	–
Nomogram vs pathologic stage	–	<.01
Nomogram vs immune prognostic model	–	<.01

## 4 DISCUSSION

Lung adenocarcinoma, which constitutes approximately 30%‐40% of NSCLCs, is a global public health problem and the most common cause of cancer‐related death.^40^ Given the substantial heterogeneity (eg clinical, molecular, pathology, surgery and radiology) observed in patients with LUAD, developing individualized treatments and predicting outcome for patients with LUAD is challenging.^41^, ^42^ Considering the significance of the immune environment in cancer progression, finding immune biomarkers to predict the prognosis of patients with LUAD is necessary, which might also play an important role in immune therapy.^43^ In the current study, clinical and mRNA expression data from LUAD level 3 RNA‐seq were acquired from the TCGA database. Then, we performed differential expression analysis between normal lung and LUAD tissues. Gene‐set enrichment analysis analyses of DEGs revealed that LUAD was strongly negatively related to 11 immune‐related gene sets, and 353 corresponding immune genes were extracted. We then used univariate, LASSO and multivariate Cox regression analyses to identify survival‐related immune genes. Next, we developed an immune prognostic model based on 2 immune genes to accurately predict LUAD patient prognosis. Receiver operating characteristic analysis suggested that the immune prognostic model has high statistical power. Additionally, multivariate Cox regression analyses revealed that the prognostic value of the immune prognostic model was independent of clinical characteristics. This model could be more routine and cost‐effective in practice as it was based on targeted sequencing of specific genes and decreased the necessity for whole‐genome sequencing.

A nomogram serves as a statistical tool with great clinical applications to assess the overall probability of specific outcome in individual patients. In the current study, a nomogram was built using pathological stage and the immune prognostic model to predict OS probability in LUAD patients. Calibration plots suggested that the actual prognosis closely corresponded to the predicted prognosis, indicating excellent prediction performance of the nomogram. At the same time, the nomogram was found to perform better than the individual risk factors according to AUC and C‐index analyses. Importantly, the prediction power of the nomogram increases by 6% (*P* < .001; C‐index 0.6021 vs 0.6621) vs the conventional pathologic stage; thus, the nomogram might be routinely utilized in the future.

Two immune genes predicted in our study were previously shown to function as potential biomarkers. The anillin actin‐binding protein (ANLN) gene is located on chromosome 7p14.2 and encodes a 1124 amino acid protein that contains four structural domains, including a RhoA‐binding domain, a C‐terminal pleckstrin homology domain and an actin‐ and myosin‐binding domain.^44^, ^45^ The ANLN protein localizes to the cytoplasm, nucleus, cell cortex, cleavage furrow and cytoskeleton and is expressed in the adult testis, placenta and spinal cord, as well as many foetal organs.^46^ ANLN was originally characterized as the human homolog of anillin, a Drosophila actin‐binding protein present in the cortex following breakdown of the nuclear envelope, as well as in the cleavage furrow during cytokinesis.^47^ Anillin plays a significant role in cell cycle progression, as well as in the assembly of the actin and myosin contractile ring separating the daughter cells.^46^ Importantly, anillin has been identified as a substrate of the anaphase‐promoting complex/cyclosome (APC/C), which is a kind of ubiquitin ligase that controls mitotic progression.^48^ Thus, anillin is a conserved protein that functions in cytoskeletal dynamics in cytokinesis and cellularization. Previous studies have shown that anillin knockdown results in ingression of the cleavage furrow and cytokinesis failure in multinucleated monkey BS‐C‐1 cells.^47^ The relationship between carcinoma and cell cycle regulation is well known. ANLN has been shown to be a biomarker of unfavourable prognosis and is related to aggressive tumour phenotypes.^49^ Anillin plays a regulatory role in the cell cycle and an important role in the invasion of pancreatic and breast cancers.^50^, ^51^ ANLN has been developed as a prognostic marker based on immunohistochemistry (IHC) and is clinically applicable to hepatocellular carcinoma.^52^ Suzuki et al^53^ explored the importance of ANLN in lung cancers using cDNA microarrays and found that the growth of NSCLC was inhibited by ANLN small interfering RNAs. Moreover, the induction of exogenous ANLN overexpression enhanced the migratory capability of mammalian cells by interacting with RhoA. Univariate analysis of gene expression from 66 patients with squamous cell carcinoma found that ANLN had a significant prognostic ability, which was validated in an independent cohort study of 26 patients.^53^, ^54^ Our results suggested that ANLN RNA is overexpressed in patients with LUAD. Patients with higher ANLN expression have a poorer prognosis.

The coagulation factor II (F2) gene encodes human prothrombin and is located on the short arm of chromosome 11, at position 11.2.^55^ The F2 gene has fourteen exons that span 21 kb, and the structural integrity of the gene is essential for viability. Mice lacking prothrombin die prematurely at the embryonic stage because of bleeding complications.^56^ Single nucleotide polymorphisms (SNPs) in patients are usually related to moderate to serious bleeding phenotypes, while the G20210A mutation in the 3′ untranslated region of the F2 gene is a well‐established risk factor for thrombophilia.^57^ The interplay among cancer, thrombosis, and haemostasis is well known.^57^ Patients with tumours have a higher risk of venous thromboembolism, and certain coagulation factors are related to the development of various types of cancers.^57^ F2 has been shown to be overexpressed and to act as a hub gene in colorectal cancer liver metastasis.^58^ In the present study, F2 was overexpressed in LUAD and was associated with poor prognosis in patients with LUAD. Our immune prognostic model includes two immune genes that provide reference values for investigators and potential avenues for additional exploration from this perspective.

Correlation analyses showed that ANLN and F2 were significantly related to Tregs and neutrophils in both the TCGA and GEO LUAD cohorts. Guo et al found that Tregs correlated with poor prognosis in LUAD, and Li et al found that the percentage of neutrophil infiltration was significantly higher in the high‐risk immune group than in the low‐risk groups in non‐squamous NSCLC, indicating that Tregs and neutrophils were risky immune cells for LUAD, which is in line with our research results.^59^, ^60^ In our research, ANLN and F2 were risky immune genes related to Tregs and neutrophils.

Our study has a few limitations. Although our study has the advantage of using massive cohorts from the TCGA and GEO databases to construct and validate the immune prognostic model, the present study nevertheless features a retrospective design. Hence, a prospective cohort is needed to validate our model. In addition, further functional studies are needed to explore the molecular functions of the two identified immune genes during LUAD progression.

To the best of our knowledge, our study is the first to identify and validate an immune prognostic model comprising two immune genes (ANLN and F2) in patients with LUAD, which is capable of serving as an independent prognostic marker for patients with LUAD, including early‐stage LUAD. In addition, this model is capable of indicating the intensity of immune responses in the LUAD microenvironment and providing new clinical applications for LUAD, taking into account the immune target as well as immune‐related treatment.

## CONFLICT OF INTEREST

The authors declare no conflict of interest.

## AUTHOR CONTRIBUTIONS

CHL, GFP and YZ participated in the study concept and design. CHL and MYL helped with co‐ordination and drafting of the manuscript. CHL, MYL and YXZ contributed to the data collection. GFP, LPW and YZ supervised the research. CHL, YXZ, QZ, LFL, JYL and SSL performed the data analysis. All authors read and approved the final manuscript.

## Data Availability

All datasets generated for this study are included in the Gene Expression Omnibus (GEO) and The Cancer Genome Atlas (TCGA) databases.

## References

[1] Torre LA , Siegel RL , Jemal A . Lung cancer statistics. Adv Exp Med Biol. 2016;893:1‐19. 26667336 10.1007/978-3-319-24223-1_1

[2] Salavaty A , Rezvani Z , Najafi A . Survival analysis and functional annotation of long non‐coding RNAs in lung adenocarcinoma. J Cell Mol Med. 2019;23(8):5600‐5617. 31211495 10.1111/jcmm.14458 PMC6652661

[3] Personal habits and indoor combustions. Volume 100 E. A review of human carcinogens. IARC Monogr Eval Carcinog Risks Hum. 2012;100:1‐538. PMC4781577 23193840

[4] Hamra GB , Guha N , Cohen A , et al. Outdoor particulate matter exposure and lung cancer: a systematic review and meta‐analysis. Environ Health Perspect. 2014;122:906‐911. 24911630 10.1289/ehp/1408092 PMC4154221

[5] Sato M , Shames DS , Gazdar AF , Minna JD . A translational view of the molecular pathogenesis of lung cancer. J Thoracic Oncol. 2007;2:327‐343. 10.1097/01.JTO.0000263718.69320.4c 17409807

[6] Lin JJ , Cardarella S , Lydon CA , et al. Five‐year survival in EGFR‐mutant metastatic lung adenocarcinoma treated with EGFR‐TKIs. J Thoracic Oncol. 2016;11:556‐565. 10.1016/j.jtho.2015.12.103 PMC4979601 26724471

[7] Qi L , Li Y , Qin Y , et al. An individualised signature for predicting response with concordant survival benefit for lung adenocarcinoma patients receiving platinum‐based chemotherapy. Br J Cancer. 2016;115:1513‐1519. 27855439 10.1038/bjc.2016.370 PMC5155365

[8] Zhang Q , Fan H , Zou Q , et al. TEAD4 exerts pro‐metastatic effects and is negatively regulated by miR6839‐3p in lung adenocarcinoma progression. J Cell Mol Med. 2018;22:3560‐3571. 29667772 10.1111/jcmm.13634 PMC6010880

[9] Corrales L , Matson V , Flood B , Spranger S , Gajewski TF . Innate immune signaling and regulation in cancer immunotherapy. Cell Res. 2017;27:96‐108. 27981969 10.1038/cr.2016.149 PMC5223230

[10] Kim R , Emi M , Tanabe K . Cancer immunoediting from immune surveillance to immune escape. Immunology. 2007;121:1‐14. 17386080 10.1111/j.1365-2567.2007.02587.x PMC2265921

[11] Rooney MS , Shukla SA , Wu CJ , Getz G , Hacohen N . Molecular and genetic properties of tumors associated with local immune cytolytic activity. Cell. 2015;160:48‐61. 25594174 10.1016/j.cell.2014.12.033 PMC4856474

[12] Li B , Severson E , Pignon JC , et al. Comprehensive analyses of tumor immunity: implications for cancer immunotherapy. Genome Biol. 2016;17:174. 27549193 10.1186/s13059-016-1028-7 PMC4993001

[13] Brahmer J , Reckamp KL , Baas P , et al. Nivolumab versus docetaxel in advanced squamous‐cell non‐small‐cell lung cancer. N Engl J Med. 2015;373:123‐135. 26028407 10.1056/NEJMoa1504627 PMC4681400

[14] Hellmann MD , Rizvi NA , Goldman JW , et al. Nivolumab plus ipilimumab as first‐line treatment for advanced non‐small‐cell lung cancer (CheckMate 012): results of an open‐label, phase 1, multicohort study. Lancet Oncol. 2017;18:31‐41. 27932067 10.1016/S1470-2045(16)30624-6 PMC5476941

[15] Gettinger SN , Horn L , Gandhi L , et al. Overall survival and long‐term safety of nivolumab (anti‐programmed death 1 antibody, BMS‐936558, ONO‐4538) in patients with previously treated advanced non‐small‐cell lung cancer. J Clin Oncol. 2015;33:2004‐2012. 25897158 10.1200/JCO.2014.58.3708 PMC4672027

[16] Garon EB , Rizvi NA , Hui R , et al. Pembrolizumab for the treatment of non‐small‐cell lung cancer. N Engl J Med. 2015;372:2018‐2028. 25891174 10.1056/NEJMoa1501824

[17] Malhotra J , Jabbour SK , Aisner J . Current state of immunotherapy for non‐small cell lung cancer. Trans Lung Cancer Res. 2017;6:196‐211. 10.21037/tlcr.2017.03.01 PMC5420529 28529902

[18] Liu B , Song Y , Liu D . Recent development in clinical applications of PD‐1 and PD‐L1 antibodies for cancer immunotherapy. J Hematol Oncol. 2017;10:174. 29195503 10.1186/s13045-017-0541-9 PMC5712158

[19] Brambilla E , Le Teuff G , Marguet S , et al. Prognostic effect of tumor lymphocytic infiltration in resectable non‐small‐cell lung cancer. J Clin Oncol. 2016;34:1223‐1230. 26834066 10.1200/JCO.2015.63.0970 PMC4872323

[20] Suzuki K , Kadota K , Sima CS , et al. Clinical impact of immune microenvironment in stage I lung adenocarcinoma: tumor interleukin‐12 receptor beta2 (IL‐12Rbeta2), IL‐7R, and stromal FoxP3/CD3 ratio are independent predictors of recurrence. J Clin Oncol. 2013;31:490‐498. 23269987 10.1200/JCO.2012.45.2052 PMC3731922

[21] Suzuki K , Kachala SS , Kadota K , et al. Prognostic immune markers in non‐small cell lung cancer. Clin Cancer Res. 2011;17:5247‐5256. 21659461 10.1158/1078-0432.CCR-10-2805

[22] Okayama H , Kohno T , Ishii Y , et al. Identification of genes upregulated in ALK‐positive and EGFR/KRAS/ALK‐negative lung adenocarcinomas. Can Res. 2012;72:100‐111. 10.1158/0008-5472.CAN-11-1403 22080568

[23] Robinson MD , McCarthy DJ , Smyth GK . edgeR: a Bioconductor package for differential expression analysis of digital gene expression data. Bioinformatics (Oxford, England). 2010;26:139‐140. 10.1093/bioinformatics/btp616 PMC2796818 19910308

[24] Subramanian A , Tamayo P , Mootha VK , et al. Gene set enrichment analysis: a knowledge‐based approach for interpreting genome‐wide expression profiles. Proc Natl Acad Sci USA. 2005;102:15545‐15550. 16199517 10.1073/pnas.0506580102 PMC1239896

[25] Camp RL , Dolled‐Filhart M , Rimm DL . X‐tile: a new bio‐informatics tool for biomarker assessment and outcome‐based cut‐point optimization. Clin Cancer Res. 2004;10:7252‐7259. 15534099 10.1158/1078-0432.CCR-04-0713

[26] Tang XR , Li YQ , Liang SB , et al. Development and validation of a gene expression‐based signature to predict distant metastasis in locoregionally advanced nasopharyngeal carcinoma: a retrospective, multicentre, cohort study. Lancet Oncol. 2018;19:382‐393. 29428165 10.1016/S1470-2045(18)30080-9

[27] Newman AM , Liu CL , Green MR , et al. Robust enumeration of cell subsets from tissue expression profiles. Nat Methods. 2015;12:453‐457. 25822800 10.1038/nmeth.3337 PMC4739640

[28] Rohr‐Udilova N , Klinglmuller F , Schulte‐Hermann R , et al. Deviations of the immune cell landscape between healthy liver and hepatocellular carcinoma. Sci Rep. 2018;8:6220. 29670256 10.1038/s41598-018-24437-5 PMC5906687

[29] Ali HR , Chlon L , Pharoah PD , Markowetz F , Caldas C . Patterns of immune infiltration in breast cancer and their clinical implications: a gene‐expression‐based retrospective study. PLoS Medicine. 2016;13:e1002194. 27959923 10.1371/journal.pmed.1002194 PMC5154505

[30] Dennis G Jr , Sherman BT , Hosack DA , et al. DAVID: database for annotation, visualization, and integrated discovery. Genome Biol. 2003;4:P3.12734009

[31] Xie C , Mao X , Huang J , et al. Wei L. KOBAS 2.0: a web server for annotation and identification of enriched pathways and diseases. Nucleic Acids Res. 2011;39:W316‐W322. 21715386 10.1093/nar/gkr483 PMC3125809

[32] Shukla S , Evans JR , Malik R , et al. Development of a RNA‐seq based prognostic signature in lung adenocarcinoma. J Natl Cancer Inst. 2017;109(1):djw200. 10.1093/jnci/djw200 PMC5051943 27707839

[33] Long J , Wang A , Bai Y , et al. Development and validation of a TP53‐associated immune prognostic model for hepatocellular carcinoma. EBioMedicine. 2019;42:363‐374. 30885723 10.1016/j.ebiom.2019.03.022 PMC6491941

[34] Rody A , Holtrich U , Pusztai L , et al. T‐cell metagene predicts a favorable prognosis in estrogen receptor‐negative and HER2‐positive breast cancers. Breast Cancer Res: BCR. 2009;11:R15. 19272155 10.1186/bcr2234 PMC2688939

[35] Ni L , Dong C . New B7 family checkpoints in human cancers. Mol Cancer Ther. 2017;16:1203‐1211. 28679835 10.1158/1535-7163.MCT-16-0761 PMC5568666

[36] Becht E , Giraldo NA , Lacroix L , et al. Estimating the population abundance of tissue‐infiltrating immune and stromal cell populations using gene expression. Genome Biol. 2016;17:218. 27765066 10.1186/s13059-016-1070-5 PMC5073889

[37] Reck M , Rodriguez‐Abreu D , Robinson AG , et al. Pembrolizumab versus chemotherapy for PD‐L1‐positive non‐small‐cell lung cancer. N Engl J Med. 2016;375:1823‐1833. 27718847 10.1056/NEJMoa1606774

[38] Herbst RS , Baas P , Kim DW , et al. Pembrolizumab versus docetaxel for previously treated, PD‐L1‐positive, advanced non‐small‐cell lung cancer (KEYNOTE‐010): a randomised controlled trial. Lancet (London, England). 2016;387:1540‐1550. 10.1016/S0140-6736(15)01281-7 26712084

[39] Carbone DP , Reck M , Paz‐Ares L , et al. First‐line nivolumab in stage IV or recurrent non‐small‐cell lung cancer. N Engl J Med. 2017;376:2415‐2426. 28636851 10.1056/NEJMoa1613493 PMC6487310

[40] Ferlay J , Shin HR , Bray F , Forman D , Mathers C , Parkin DM . Estimates of worldwide burden of cancer in 2008: GLOBOCAN 2008. Int J Cancer. 2010;127:2893‐2917. 21351269 10.1002/ijc.25516

[41] Yoshizawa A , Motoi N , Riely GJ , et al. Impact of proposed IASLC/ATS/ERS classification of lung adenocarcinoma: prognostic subgroups and implications for further revision of staging based on analysis of 514 stage I cases. Mod Pathol. 2011;24(5):653‐664 21252858 10.1038/modpathol.2010.232

[42] Jin X , Di X , Wang R , et al. RBM10 inhibits cell proliferation of lung adenocarcinoma via RAP1/AKT/CREB signalling pathway. J Cell Mol Med. 2019;23:3897‐3904. 30955253 10.1111/jcmm.14263 PMC6533519

[43] Chen DS , Mellman I . Elements of cancer immunity and the cancer‐immune set point. Nature. 2017;541:321‐330. 28102259 10.1038/nature21349

[44] Ota T , Suzuki Y , Nishikawa T , et al. Complete sequencing and characterization of 21,243 full‐length human cDNAs. Nat Genet. 2004;36:40‐45. 14702039 10.1038/ng1285

[45] Strausberg RL , Feingold EA , Grouse LH , et al. Generation and initial analysis of more than 15,000 full‐length human and mouse cDNA sequences. Proc Natl Acad Sci USA. 2002;99:16899‐16903. 12477932 10.1073/pnas.242603899 PMC139241

[46] Straight AF , Field CM , Mitchison TJ . Anillin binds nonmuscle myosin II and regulates the contractile ring. Mol Biol Cell. 2005;16:193‐201. 15496454 10.1091/mbc.E04-08-0758 PMC539163

[47] Oegema K , Savoian MS , Mitchison TJ , Field CM . Functional analysis of a human homologue of the *Drosophila* actin binding protein anillin suggests a role in cytokinesis. J Cell Biol. 2000;150:539‐552. 10931866 10.1083/jcb.150.3.539 PMC2175195

[48] Monzo P , Gauthier NC , Keslair F , et al. Clues to CD2‐associated protein involvement in cytokinesis. Mol Biol Cell. 2005;16:2891‐2902. 15800069 10.1091/mbc.E04-09-0773 PMC1142433

[49] Ronkainen H , Hirvikoski P , Kauppila S , Vaarala MH . Anillin expression is a marker of favourable prognosis in patients with renal cell carcinoma. Oncol Rep. 2011;25:129‐133. 21109967

[50] Wang Z , Chen J , Zhong MZ , et al. Overexpression of ANLN contributed to poor prognosis of anthracycline‐based chemotherapy in breast cancer patients. Cancer Chemother Pharmacol. 2017;79:535‐543. 28243684 10.1007/s00280-017-3248-2

[51] Olakowski M , Tyszkiewicz T , Jarzab M , et al. NBL1 and anillin (ANLN) genes over‐expression in pancreatic carcinoma. Folia Histochem Cytobiol. 2009;47:249‐255. 19995712 10.2478/v10042-009-0031-1

[52] Kim H , Kim K , Yu SJ , et al. Development of biomarkers for screening hepatocellular carcinoma using global data mining and multiple reaction monitoring. PLoS ONE. 2013;8:e63468. 23717429 10.1371/journal.pone.0063468 PMC3661589

[53] Suzuki C , Daigo Y , Ishikawa N , et al. ANLN plays a critical role in human lung carcinogenesis through the activation of RHOA and by involvement in the phosphoinositide 3‐kinase/AKT pathway. Can Res. 2005;65:11314‐11325. 10.1158/0008-5472.CAN-05-1507 16357138

[54] Skrzypski M , Jassem E , Taron M , et al. Three‐gene expression signature predicts survival in early‐stage squamous cell carcinoma of the lung. Clin Cancer Res. 2008;14:4794‐4799. 18676750 10.1158/1078-0432.CCR-08-0576

[55] Degen SJ , Davie EW . Nucleotide sequence of the gene for human prothrombin. Biochemistry. 1987;26:6165‐6177. 2825773 10.1021/bi00393a033

[56] Sun WY , Witte DP , Degen JL , et al. Prothrombin deficiency results in embryonic and neonatal lethality in mice. Proc Natl Acad Sci USA. 1998;95:7597‐7602. 9636195 10.1073/pnas.95.13.7597 PMC22695

[57] Lancellotti S , Basso M , De Cristofaro R . Congenital prothrombin deficiency: an update. Semin Thromb Hemost. 2013;39:596‐606. 23852823 10.1055/s-0033-1348948

[58] Zhang T , Guo J , Gu J , et al. Identifying the key genes and microRNAs in colorectal cancer liver metastasis by bioinformatics analysis and in vitro experiments. Oncol Rep. 2019;41:279‐291. 30542696 10.3892/or.2018.6840 PMC6278419

[59] Guo X , Zhang Y , Zheng L , et al. Global characterization of T cells in non‐small‐cell lung cancer by single‐cell sequencing. Nat Med. 2018;24:978‐985. 29942094 10.1038/s41591-018-0045-3

[60] Li B , Cui Y , Diehn M , Li R . Development and validation of an individualized immune prognostic signature in early‐stage nonsquamous non‐small cell lung cancer. JAMA Oncol. 2017;3:1529‐1537. 28687838 10.1001/jamaoncol.2017.1609 PMC5710196

