# Supplementary material for: Systematic construction and validation of an immune prognostic model for lung adenocarcinoma
Source: J Cell Mol Med. 2019 Nov 28;24(2):1233–44. doi: 10.1111/jcmm.14719 (PMC6991688; doi:10.1111/jcmm.14719)
Supplement: Supplementary file 1 [file JCMM-24-1233-s001.pdf]

Table S1. Differentially expressed genes between LUADs and adjacent normal tissues.

Gene	Log_2 FC	Log_2 CPM	P value	FDR
RTKN2	-4.09793	5.484527	2.12E-229	7.39E-225
OTUD1	-2.13028	4.570319	9.63E-214	1.38E-209
FAM107A	-4.54942	5.21603	1.19E-213	1.38E-209
EPAS1	-2.71803	9.204201	5.31E-203	4.62E-199
TEK	-3.26767	4.35513	1.36E-200	9.44E-197
RGCC	-2.89103	6.184407	6.35E-200	3.69E-196
S1PR1	-2.86127	5.229737	2.27E-199	1.13E-195
SPAAR	-2.73004	0.427936	7.50E-198	3.27E-194
SEMA3G	-3.22479	3.950561	4.22E-197	1.63E-193
STX11	-3.01292	3.556667	4.01E-196	1.40E-192
RAMP3	-3.22979	4.581967	6.96E-193	2.20E-189
PECAM1	-2.32781	8.001773	3.80E-187	1.10E-183
EMP2	-2.7477	8.58403	1.00E-186	2.69E-183
ACVRL1	-2.5806	5.586594	1.35E-185	3.37E-182
EDNRB	-3.50679	5.037285	1.40E-183	3.25E-180
LDB2	-2.62741	4.594229	1.55E-179	3.37E-176
SLC6A4	-6.17078	4.574583	1.67E-178	3.42E-175
FCN3	-4.52488	5.01849	2.93E-178	5.67E-175
PTPN21	-2.46474	4.75799	1.39E-175	2.54E-172
RAMP2	-2.89032	4.410683	1.28E-174	2.22E-171
PRX	-3.37747	4.58795	1.84E-173	3.05E-170
HSPA12B	-2.79577	3.218551	1.20E-172	1.90E-169
AL606469.1	-5.16133	-0.91885	8.87E-172	1.34E-168
WWC2	-2.36809	5.511767	2.31E-170	3.36E-167
HSPC324	-3.69735	-0.64791	7.13E-166	9.93E-163
LIMS2	-2.82077	4.708771	8.07E-165	1.08E-161
ROBO4	-2.84988	5.076414	1.67E-162	2.16E-159
AC093110.1	-3.28597	1.330055	2.31E-162	2.87E-159
ANGPT4	-4.2352	0.268588	3.64E-162	4.37E-159
CALCRL	-2.78637	5.653037	1.40E-160	1.63E-157
JAM2	-2.53142	4.017539	3.56E-159	4.00E-156
AGER	-5.03579	8.257922	4.31E-159	4.69E-156
FABP4	-4.90511	4.365206	2.52E-158	2.66E-155
MYZAP	-3.3347	1.980656	5.19E-157	5.32E-154
SPOCK2	-3.20893	7.093894	5.57E-157	5.54E-154
CD101	-2.81544	2.65801	1.20E-156	1.16E-153
CDH5	-2.5606	6.080047	6.18E-155	5.82E-152
TAL1	-2.81645	2.044138	4.82E-153	4.41E-150
FHL1	-3.43317	6.057542	8.70E-153	7.77E-150
KANK3	-2.85295	2.881967	5.94E-152	5.17E-149

For Peer Review

CAVIN2	-3.31626	6.269772	9.20E-152	7.82E-149
FMO2	-3.29638	5.882069	4.14E-150	3.43E-147
FOXF1	-2.75836	3.68523	1.68E-147	1.36E-144
TMEM100	-4.36666	4.611297	1.22E-146	9.66E-144
PLAC9	-2.73664	2.624307	3.05E-144	2.36E-141
ECSCR	-2.38873	2.007194	1.74E-142	1.32E-139
FENDRR	-3.92081	3.350127	6.59E-142	4.88E-139
SH2D3C	-2.30579	4.597241	1.45E-141	1.05E-138
SMAD6	-2.58099	4.224469	1.64E-141	1.17E-138
CYYR1	-2.28837	4.066661	2.32E-141	1.61E-138
GIMAP8	-2.47542	4.557643	3.29E-141	2.25E-138
SGCG	-3.84536	-0.06415	1.23E-140	8.23E-138
CLEC3B	-3.69797	4.337403	3.67E-139	2.41E-136
GYPE	-3.3147	-0.2579	4.90E-139	3.16E-136
LINC00968	-3.54471	0.587265	2.45E-136	1.55E-133
CAV1	-3.42424	8.089906	4.58E-136	2.85E-133
ADRB2	-3.09643	3.257648	6.07E-136	3.71E-133
CLEC1A	-2.33092	1.997565	2.62E-135	1.54E-132
FGD5	-2.26986	4.840247	5.67E-135	3.29E-132
ARHGEF26	-2.68744	4.60897	1.06E-134	6.06E-132
GRK5	-2.50209	4.975141	2.67E-134	1.50E-131
VIPR1	-3.24821	4.883072	3.88E-133	2.15E-130
LYVE1	-3.21664	3.771929	4.37E-132	2.38E-129
MIR3945HG	-3.57507	0.001129	1.89E-130	1.01E-127
LANCL1-AS1	-3.64066	0.28852	7.37E-130	3.89E-127
GPD1	-3.94911	3.605883	2.97E-129	1.54E-126
MYCT1	-2.27168	3.450224	7.32E-128	3.75E-125
LRRC32	-2.38166	5.552557	1.36E-127	6.85E-125
PTPRB	-2.62774	5.9272	7.63E-127	3.79E-124
ARHGEF15	-2.46302	3.867873	1.07E-126	5.27E-124
GPM6A	-4.46804	3.167915	2.61E-126	1.26E-123
LINC01082	-4.16519	-2.53991	4.02E-126	1.92E-123
STARD13	-2.02874	4.16818	2.03E-125	9.55E-123
CCM2L	-2.52955	2.042161	2.31E-125	1.07E-122
CLEC14A	-2.30748	5.039475	7.92E-124	3.63E-121
HBB	-4.26309	6.872149	1.15E-123	5.21E-121
STARD8	-2.22133	3.920659	1.27E-123	5.65E-121
SOX7	-2.94317	2.929531	3.28E-123	1.44E-120
ERG	-2.16528	4.426154	5.01E-123	2.18E-120
HBA2	-4.16125	4.350919	8.80E-123	3.78E-120
C20orf202	-2.62741	-0.9294	9.54E-123	4.05E-120
CSRNP1	-2.39452	6.151903	1.77E-121	7.42E-119
INMT	-3.52047	5.764038	4.67E-121	1.93E-118

For Peer Review

NOVA2	-2.43572	2.505845	6.59E-121	2.70E-118
HBA1	-4.40078	-0.9801	1.54E-120	6.24E-118
RXFP1	-3.11374	1.137617	1.61E-120	6.45E-118
USHBP1	-2.3333	1.866033	2.64E-120	1.05E-117
TNNC1	-3.57062	3.801053	2.69E-120	1.05E-117
EMCN	-2.75438	4.317529	3.37E-120	1.30E-117
NECAB1	-3.21763	2.387197	7.85E-120	3.00E-117
CLIC5	-3.63325	6.217002	2.21E-119	8.38E-117
AP001189.1	-3.08808	-0.49735	9.60E-119	3.60E-116
GRASP	-2.36349	3.618413	1.04E-118	3.85E-116
THSD1	-2.25695	2.554596	2.59E-118	9.49E-116
CD300LG	-4.97145	1.422046	2.71E-118	9.82E-116
SCUBE1	-3.5725	3.029855	1.20E-116	4.31E-114
AC135012.3	-4.06994	-1.78388	6.98E-116	2.48E-113
SLC39A8	-2.50125	7.46354	1.62E-115	5.68E-113
ARHGAP6	-2.38969	2.600224	3.55E-115	1.23E-112
GPR146	-2.30807	0.964989	3.97E-115	1.37E-112
AOC3	-2.66702	6.295717	1.01E-114	3.44E-112
MMRN2	-2.14228	4.915222	1.52E-114	5.12E-112
MCEMP1	-4.03942	4.657793	2.38E-114	7.97E-112
FXYD1	-2.96222	0.169957	3.74E-114	1.24E-111
GPIHBP1	-3.71619	3.158656	3.86E-114	1.27E-111
ALAS2	-4.10751	-0.93101	4.27E-113	1.39E-110
HID1-AS1	-2.81004	-1.84104	6.10E-113	1.96E-110
RGS9	-2.50151	1.07477	4.47E-112	1.43E-109
CD93	-2.22406	6.924616	2.54E-110	8.05E-108
SOX17	-2.72278	1.8779	5.19E-110	1.63E-107
AC009093.3	-4.15517	1.541765	6.28E-110	1.95E-107
C14orf132	-2.64793	4.569586	3.73E-109	1.15E-106
HIGD1B	-2.79288	2.094576	1.61E-108	4.91E-106
BTNL9	-3.43876	3.632061	3.79E-108	1.14E-105
SLIT2	-2.51272	5.103976	3.94E-108	1.17E-105
CA4	-4.36011	2.780187	4.06E-108	1.20E-105
WNT3A	-4.11414	1.28478	1.80E-107	5.28E-105
STXBP6	-3.39054	3.335454	3.63E-107	1.05E-104
FREM3	-4.32829	-0.8811	4.43E-107	1.27E-104
TNS1	-2.18336	8.374996	1.44E-106	4.11E-104
ANOS1	-2.81836	5.73111	2.00E-106	5.65E-104
RASIP1	-2.31554	3.881375	2.94E-106	8.25E-104
CD5L	-4.07166	-0.97652	4.00E-106	1.10E-103
CLEC4M	-4.48216	-1.3956	1.43E-105	3.85E-103
AL445307.1	-3.44641	-2.9653	4.22E-105	1.13E-102
NCKAP5	-3.54335	3.551129	2.88E-104	7.66E-102

For Peer Review

EFCC1	-2.59582	2.669976	4.73E-104	1.24E-101
OVCH1	-3.97187	-0.3613	4.73E-104	1.24E-101
C10orf67	-3.99082	-0.12354	9.17E-104	2.37E-101
AC084880.1	-2.49234	-0.71962	1.54E-103	3.93E-101
AL109741.1	-2.52816	-1.66766	5.22E-103	1.33E-100
NMUR1	-2.69107	0.774764	6.70E-103	1.69E-100
FEZ1	-2.17042	3.236879	1.58E-102	3.95E-100
AC093890.1	-2.75803	-1.39008	5.61E-102	1.40E-99
JPH4	-2.60419	0.772251	6.55E-102	1.60E-99
LINC00656	-3.49371	-2.45646	8.04E-102	1.96E-99
ST8SIA6	-3.35041	0.91772	1.51E-101	3.65E-99
ARHGAP31	-2.15497	5.695634	2.21E-101	5.30E-99
HEG1	-2.14588	6.676596	6.80E-101	1.62E-98
SEMA6A	-2.583	3.71424	1.45E-100	3.43E-98
ANKRD1	-4.45017	3.625755	2.43E-100	5.71E-98
PCAT19	-2.4363	2.817325	7.62E-100	1.78E-97
AC011899.2	-2.39731	1.928726	9.95E-100	2.31E-97
FAM189A2	-2.94534	4.289866	1.34E-99	3.09E-97
GLIPR2	-2.22258	5.5761	1.84E-99	4.21E-97
GIMAP6	-2.10891	4.902157	1.85E-99	4.22E-97
HBEGF	-2.58504	5.293866	2.10E-99	4.75E-97
MYOC	-4.5413	-0.22107	4.55E-99	1.02E-96
TCF21	-3.12771	3.493456	4.94E-99	1.10E-96
TENT5B	-2.8053	3.233993	5.41E-99	1.19E-96
ADAMTS8	-3.53824	3.343836	6.84E-99	1.50E-96
SCN4B	-2.83001	3.279692	1.28E-98	2.79E-96
TNR	-3.88563	0.572096	1.34E-98	2.89E-96
LINC02016	-4.9432	-1.08356	2.70E-98	5.81E-96
FHL5	-2.79013	1.857086	8.18E-98	1.75E-95
CSF3	-4.67622	3.266315	2.54E-96	5.37E-94
NLRC4	-2.10112	2.533998	3.23E-96	6.77E-94
SIRPB1	-2.86463	3.053508	5.17E-96	1.08E-93
ARC	-3.464	1.336453	5.34E-96	1.11E-93
SLC4A1	-3.43274	-2.07906	9.88E-96	2.03E-93
AC095050.1	-4.53831	-2.86024	2.11E-95	4.33E-93
CAVIN1	-2.08022	7.966133	2.89E-95	5.88E-93
RS1	-3.72693	-0.11216	3.26E-95	6.59E-93
FAM110D	-2.34029	0.884045	2.01E-94	4.00E-92
FRMD3	-2.45645	2.426141	3.37E-94	6.62E-92
UPK3B	-4.15506	4.776757	3.72E-94	7.28E-92
DNASE1L3	-3.50191	2.348313	1.01E-93	1.96E-91
TGFBR3	-2.58934	4.608944	2.35E-93	4.54E-91
THBD	-2.43745	5.947876	3.02E-93	5.80E-91

For Peer Review

PYCR1	3.712005	6.81295	3.03E-93	5.80E-91
AC018647.1	-2.72524	-1.24186	3.62E-93	6.89E-91
LINC01996	-4.4319	-0.69717	5.38E-93	1.01E-90
GIMAP1	-2.02697	3.306238	7.88E-93	1.47E-90
CHRM1	-4.43524	-0.29363	8.60E-93	1.60E-90
AKAP2	-2.56233	0.335777	1.29E-92	2.39E-90
DPP6	-3.27617	-0.02566	5.63E-92	1.04E-89
GSTM5	-2.47084	2.123974	6.14E-92	1.12E-89
CMTM2	-2.32095	-0.87633	9.07E-92	1.65E-89
CGNL1	-2.2332	5.68417	4.34E-91	7.79E-89
AC084030.1	-4.40964	-3.4515	1.10E-90	1.97E-88
LTBP4	-2.23274	7.06693	1.16E-90	2.05E-88
JCAD	-2.11014	5.936738	1.72E-89	3.00E-87
AP001189.3	-2.49957	-0.59023	5.03E-89	8.54E-87
SPN	-2.30961	5.055639	6.64E-89	1.12E-86
SMIM25	-2.49375	3.972236	9.26E-89	1.56E-86
CCDC85A	-2.82696	0.969923	1.29E-88	2.15E-86
NXPH3	-2.43106	1.89208	6.85E-88	1.14E-85
NPR1	-2.62674	4.363086	1.71E-87	2.83E-85
AC026369.3	-3.35646	0.091048	5.27E-87	8.69E-85
AC104984.1	-4.1302	-3.13687	9.34E-87	1.53E-84
AC128709.3	-4.28233	-2.77536	1.33E-86	2.17E-84
PHACTR1	-2.30525	4.390754	4.41E-85	6.98E-83
AC125807.2	-2.1551	1.756319	6.43E-85	1.01E-82
SH3GL3	-4.23367	-0.10301	1.07E-84	1.67E-82
CLDN5	-2.7119	5.305698	1.55E-84	2.40E-82
VWF	-2.33875	8.159045	1.82E-84	2.81E-82
AP001528.3	-2.41637	0.959016	2.09E-84	3.21E-82
RAI2	-2.14059	3.71044	2.14E-84	3.27E-82
ADGRE3	-2.89347	0.556396	2.42E-84	3.67E-82
CDO1	-2.71081	1.529671	4.07E-84	6.13E-82
AC104984.4	-4.05788	-2.52663	4.33E-84	6.49E-82
LDLR	-2.11342	7.088677	1.40E-83	2.10E-81
ASPA	-2.65107	0.840961	8.50E-83	1.26E-80
ADAMTS1	-2.36085	5.819209	2.06E-82	3.03E-80
TUBB1	-2.67063	-0.22642	3.75E-82	5.51E-80
SEMA5A	-2.55773	5.114536	4.49E-82	6.56E-80
DPEP2	-2.21156	2.86506	5.29E-82	7.71E-80
LAMP3	-2.78122	7.385287	5.99E-82	8.69E-80
SSTR4	-3.81491	-3.94892	6.81E-82	9.84E-80
AC008763.3	-3.9893	-3.37139	4.35E-81	6.23E-79
OR6K3	-4.20757	-3.68844	5.03E-81	7.18E-79
ABI3BP	-2.52913	5.358292	3.76E-80	5.28E-78

For Peer Review

LINC00163	-3.40193	-2.12366	4.43E-80	6.19E-78
VEGFD	-3.59673	3.33347	5.37E-80	7.48E-78
MGAT3	-3.0324	3.938899	7.39E-80	1.02E-77
PTPN5	-3.41502	-0.90844	9.92E-80	1.37E-77
FGR	-2.12793	4.859463	2.16E-79	2.97E-77
MFAP4	-2.66731	7.066451	2.66E-79	3.65E-77
MS4A7	-2.16093	5.508708	5.57E-79	7.60E-77
SELP	-2.3407	3.406614	1.04E-78	1.42E-76
PEAR1	-2.02694	3.173483	1.91E-78	2.58E-76
CASP12	-2.52486	-1.34131	2.97E-78	3.99E-76
SLC19A3	-2.92054	2.660037	4.46E-78	5.98E-76
UNC45B	-2.57244	-1.0965	1.88E-77	2.50E-75
CMTM5	-2.91303	-2.65603	2.32E-77	3.06E-75
PRKG2	-2.65045	1.774153	2.46E-77	3.24E-75
GRIA1	-3.74326	1.96871	2.98E-77	3.90E-75
AGRP	-2.98882	-0.21096	3.64E-77	4.75E-75
COX4I2	-2.2845	2.458731	5.84E-77	7.58E-75
FAM162B	-2.30844	1.648428	6.03E-77	7.81E-75
CD36	-2.90498	5.549726	7.32E-77	9.44E-75
ALKAL2	-3.06025	0.605871	1.12E-76	1.44E-74
ABCA8	-2.8698	3.875383	9.35E-76	1.18E-73
TMEM88	-2.15238	1.477253	1.01E-75	1.28E-73
TBX3	-2.14323	3.879506	1.10E-75	1.37E-73
FPR2	-3.01162	2.442679	1.57E-75	1.94E-73
CCDC141	-3.11938	1.489475	1.61E-75	1.99E-73
CD52	-2.37445	5.845789	1.69E-75	2.08E-73
IQSEC3	-2.61457	1.55747	1.78E-75	2.18E-73
CAV2	-2.35153	6.947051	1.94E-75	2.37E-73
Z95114.3	-2.21243	-1.13214	2.78E-75	3.39E-73
NCAPGP2	-4.10309	-2.58759	3.74E-75	4.52E-73
LRRN3	-2.85277	1.836828	7.12E-75	8.58E-73
PI16	-3.5968	1.246807	8.07E-75	9.69E-73
OLR1	-2.56188	5.684688	1.97E-74	2.36E-72
TEDC2	3.510931	2.559846	2.23E-74	2.66E-72
PALMD	-2.0627	3.982599	2.94E-74	3.49E-72
DKK2	-2.31553	2.486023	8.75E-74	1.03E-71
AC087521.1	-2.70377	-0.91873	1.12E-73	1.31E-71
HTR3C	-4.54819	-1.16019	1.70E-73	1.97E-71
ITM2A	-2.04801	4.573472	2.92E-73	3.38E-71
KIF17	-2.0081	1.4129	5.81E-73	6.70E-71
GRIK4	-2.51835	0.108073	6.15E-73	7.07E-71
LGI3	-4.2683	3.798505	9.51E-73	1.09E-70
AC027288.3	-3.18473	1.306406	1.56E-72	1.78E-70

For Peer Review

KIAA1324L	-2.11059	4.639367	1.62E-72	1.85E-70
TSPAN18	-2.02243	4.441396	1.77E-72	2.00E-70
ITLN2	-4.6301	2.383726	1.99E-72	2.25E-70
LINC01081	-3.11497	-3.81541	2.83E-72	3.19E-70
PKNOX2	-2.45324	2.343993	2.99E-72	3.35E-70
MASP1	-2.7099	2.063014	3.64E-72	4.06E-70
GDF10	-3.20304	2.582319	3.73E-72	4.15E-70
SLC14A1	-2.8639	1.755944	4.98E-72	5.52E-70
ADRB1	-3.15641	2.751374	7.69E-72	8.47E-70
SFTPC	-4.73706	11.81612	1.19E-71	1.31E-69
ADAMTS7P3	-3.54768	1.282247	6.24E-71	6.79E-69
ACTN2	-2.82425	0.258727	6.52E-71	7.07E-69
SERTM1	-4.78922	1.34413	6.55E-71	7.08E-69
ANGPTL7	-3.73493	-0.72265	1.11E-70	1.19E-68
CXCR2	-2.61187	1.408357	1.30E-70	1.39E-68
APOLD1	-2.03207	4.766743	1.39E-70	1.49E-68
ACADL	-3.16849	2.613427	1.47E-70	1.56E-68
AL355974.2	-2.91021	-1.87016	2.26E-70	2.37E-68
DES	-3.06343	4.299359	3.42E-70	3.59E-68
LRRC2	-2.47746	0.567316	5.64E-70	5.89E-68
GLDN	-2.54072	3.181233	5.87E-70	6.11E-68
MPIG6B	-2.21678	-0.78801	1.15E-69	1.20E-67
SYNPO2L	-2.68841	-0.61889	1.46E-69	1.52E-67
GCOM1	-2.68371	-0.61691	1.71E-69	1.77E-67
LINC01936	-2.45237	2.009148	1.78E-69	1.83E-67
TGM1	-2.44232	1.721256	2.07E-69	2.11E-67
ABCG2	-2.06408	2.663111	3.33E-69	3.37E-67
RNU6-529P	-2.06509	-2.30918	6.40E-69	6.42E-67
IQGAP3	3.611311	4.869145	7.00E-69	6.98E-67
AP000438.1	-2.95529	-2.95872	9.69E-69	9.61E-67
OR7E47P	-2.17414	1.893152	1.11E-68	1.09E-66
MSR1	-2.32352	6.456231	1.85E-68	1.82E-66
LINC00551	-3.08001	-0.87295	2.47E-68	2.43E-66
LIN7A	-2.54356	2.702857	3.59E-68	3.53E-66
FAM83A	6.80058	7.150792	4.16E-68	4.06E-66
ETV4	3.835254	5.517111	4.29E-68	4.17E-66
OSCAR	-2.23903	4.005945	2.13E-67	2.04E-65
MARCO	-3.03045	6.664781	2.89E-67	2.77E-65
IGSF10	-2.85367	3.496169	5.12E-67	4.87E-65
CNTN6	-3.47006	1.731589	5.13E-67	4.87E-65
PRAM1	-2.16941	2.903245	6.70E-67	6.30E-65
MAMDC2	-2.7223	3.991421	1.05E-66	9.80E-65
DCC	-2.68134	1.165123	1.31E-66	1.22E-64

SLIT3	-2.44405	5.492169	1.57E-66	1.45E-64
DACH1	-2.50068	2.510617	2.35E-66	2.16E-64
FO681492.1	-2.88097	0.436453	2.57E-66	2.35E-64
ADGRE1	-2.71427	1.878995	2.99E-66	2.73E-64
SIGLEC11	-2.44774	0.563593	6.63E-66	6.03E-64
COL6A6	-2.86043	3.672314	7.80E-66	7.07E-64
SULT1C4	-2.30412	1.622059	1.07E-65	9.63E-64
TOP2A	3.86802	6.846106	1.13E-65	1.02E-63
LINC01352	-2.31916	-1.83571	1.48E-65	1.33E-63
SAPCD2	3.774519	3.976815	3.93E-65	3.53E-63
LINC01197	-2.07717	-0.36624	4.26E-65	3.81E-63
CFP	-2.10014	2.256858	6.35E-65	5.67E-63
DLC1	-2.25476	6.776974	6.99E-65	6.23E-63
ODF3L1	-2.36861	-0.32767	7.81E-65	6.93E-63
TNXB	-2.73232	5.271419	1.19E-64	1.05E-62
GOLM1	2.63922	8.154589	1.34E-64	1.17E-62
CORO2B	-2.2001	2.087945	1.35E-64	1.18E-62
SELE	-2.93889	2.301376	1.92E-64	1.67E-62
AC006273.1	-2.5868	-1.87427	3.20E-64	2.76E-62
OTC	-3.63585	-2.22517	3.87E-64	3.34E-62
UBE2T	3.29843	4.151333	7.92E-64	6.77E-62
KCNA4	-3.90439	-0.55399	9.37E-64	7.99E-62
AC011511.5	-2.61332	1.457765	9.75E-64	8.28E-62
AC004947.1	-3.03104	-1.12396	9.75E-64	8.28E-62
IQCN	-2.46809	3.202808	1.78E-63	1.50E-61
AL445423.1	-2.37996	-2.48907	1.86E-63	1.57E-61
TMEM184A	3.121226	5.407563	2.03E-63	1.71E-61
ANGPT1	-2.30579	4.008684	2.12E-63	1.77E-61
TUBB3	4.729563	2.977067	2.55E-63	2.13E-61
AC010776.3	-3.81341	-3.77038	2.80E-63	2.34E-61
VSIG4	-2.39558	6.192122	3.31E-63	2.75E-61
KLF4	-2.29097	5.580683	6.58E-63	5.42E-61
LINC02154	-3.46836	0.379378	8.24E-63	6.76E-61
PTCRA	-2.44638	-0.48195	8.85E-63	7.25E-61
ADM2	3.469205	4.027208	1.00E-62	8.17E-61
ADAMTSL3	-2.31724	3.23931	1.07E-62	8.71E-61
PLA2G4F	-2.73339	4.504057	1.22E-62	9.91E-61
MYH2	-3.18202	-1.5743	1.23E-62	1.00E-60
CD300C	-2.10437	2.207294	1.25E-62	1.01E-60
AC010976.2	-3.01513	-1.20593	1.27E-62	1.03E-60
RPL13AP17	-3.9765	1.488618	1.35E-62	1.09E-60
AC009806.1	-2.34516	-2.73475	1.49E-62	1.20E-60
B3GNT3	4.386821	5.468371	1.62E-62	1.30E-60

For Peer Review

CPED1	-2.02984	3.814883	1.77E-62	1.41E-60
LINC00702	-2.27622	0.854183	2.42E-62	1.92E-60
AC104211.1	-2.86608	-3.07109	2.43E-62	1.93E-60
LINC01447	-2.9701	-2.98022	2.61E-62	2.06E-60
ERCC6L	3.280314	2.158867	3.55E-62	2.79E-60
ADH1B	-3.29225	6.426858	5.00E-62	3.90E-60
CDC25C	3.657284	2.094816	5.21E-62	4.06E-60
S100A3	-2.37288	1.332217	5.69E-62	4.42E-60
SYT15	-2.53591	-0.00386	1.75E-61	1.34E-59
ST6GALNAC5	-2.23675	3.344201	1.80E-61	1.37E-59
HAS1	-3.39539	0.761279	2.91E-61	2.22E-59
MTATP8P1	-3.35034	-0.48346	3.50E-61	2.66E-59
KIF4A	3.835807	4.229293	3.61E-61	2.73E-59
LINC01290	-2.22339	-1.60093	4.47E-61	3.38E-59
GPX3	-2.26865	8.1912	4.60E-61	3.46E-59
ITGA8	-2.12438	4.495529	4.83E-61	3.63E-59
SH2D4B	-2.26355	-1.37826	5.45E-61	4.09E-59
AL354714.1	-4.00259	-1.46157	7.70E-61	5.74E-59
AC104237.3	-3.47358	-2.9736	7.76E-61	5.77E-59
MMRN1	-2.38414	3.684457	9.48E-61	7.02E-59
AL096711.2	-2.54841	-0.60367	1.17E-60	8.62E-59
FAM83A-AS1	6.454833	1.938404	1.46E-60	1.08E-58
KCNT2	-2.59315	1.562903	1.61E-60	1.18E-58
KIF14	3.741822	3.241908	2.54E-60	1.86E-58
CXCR1	-2.87875	0.653884	2.79E-60	2.04E-58
LINC02014	3.907066	-0.12341	2.80E-60	2.04E-58
INMT-MINDY4	-3.15609	-2.7067	3.08E-60	2.24E-58
LINC01506	-2.46018	-2.45455	3.15E-60	2.28E-58
PTPRQ	-3.61116	0.560151	4.73E-60	3.42E-58
UHRF1	3.351507	3.895936	5.17E-60	3.72E-58
XRCC2	3.210616	2.687373	7.52E-60	5.40E-58
ANGPTL1	-2.43162	1.932326	8.94E-60	6.38E-58
ANO2	-2.17333	0.089718	1.56E-59	1.11E-57
EZH2	2.535352	4.298832	1.68E-59	1.19E-57
RSPO1	-3.09864	0.654472	2.43E-59	1.71E-57
ORC6	3.031765	2.867489	2.56E-59	1.80E-57
E2F8	3.172128	2.904638	3.32E-59	2.33E-57
NEK2	3.736956	3.880409	3.54E-59	2.48E-57
STIL	2.547959	3.636532	3.71E-59	2.60E-57
ASPM	3.709946	4.385592	4.09E-59	2.86E-57
CFD	-2.36174	5.090431	4.96E-59	3.46E-57
HJURP	3.858652	3.900362	6.59E-59	4.58E-57
TBX4	-2.20068	3.718518	1.03E-58	7.12E-57

For Peer Review

CASQ2	-2.76211	0.628221	1.06E-58	7.28E-57
VEPH1	-2.66199	4.508449	1.14E-58	7.79E-57
SOSTDC1	-3.7145	2.635268	1.31E-58	8.91E-57
IL6	-2.85525	3.766171	1.37E-58	9.32E-57
ARHGEF39	2.435045	2.759081	1.70E-58	1.15E-56
AC131649.2	-2.69804	2.092755	1.78E-58	1.20E-56
AC104237.2	-3.44835	-3.29213	1.81E-58	1.22E-56
PDZD2	-2.25371	5.133905	1.82E-58	1.23E-56
PLEKHN1	3.128581	2.838803	2.16E-58	1.45E-56
BUB1B	3.39723	3.849806	3.01E-58	2.01E-56
LRRC36	-2.93809	2.642506	3.69E-58	2.45E-56
CCL23	-2.52469	0.76134	3.83E-58	2.54E-56
CHRNA2	-3.60332	-2.23692	4.54E-58	2.99E-56
KIF20A	3.151759	4.21347	5.39E-58	3.53E-56
LGR4	2.700655	6.08523	5.76E-58	3.77E-56
SPAG5	3.095168	4.889017	5.80E-58	3.79E-56
IL7R	-2.07469	6.214405	7.10E-58	4.63E-56
CENPF	3.483951	5.657509	1.10E-57	7.13E-56
LINC01863	-3.2263	-2.05633	1.30E-57	8.43E-56
WISP2	-2.19985	3.85194	1.38E-57	8.91E-56
AC019193.2	-2.30157	-2.69946	1.45E-57	9.29E-56
KIF26B	3.110125	5.476591	1.88E-57	1.20E-55
AC022509.2	-2.14423	-0.80119	2.87E-57	1.83E-55
LINC01977	4.84497	0.608111	3.68E-57	2.34E-55
MED4-AS1	-2.26973	-2.09902	3.80E-57	2.41E-55
DLGAP5	3.68198	3.983687	3.97E-57	2.52E-55
TBX2-AS1	-2.05301	0.464192	5.41E-57	3.42E-55
HSPB6	-2.49242	3.443135	6.27E-57	3.95E-55
FGFBP2	-3.09551	0.511465	7.21E-57	4.52E-55
PDK4	-2.42075	6.120434	9.59E-57	6.00E-55
PTPRD-AS1	-2.30103	-1.16666	1.01E-56	6.31E-55
NUF2	3.701708	3.596317	1.28E-56	7.93E-55
ADRA1A	-3.47878	-0.63724	1.51E-56	9.33E-55
SPAG4	2.893909	3.898156	1.78E-56	1.10E-54
MEX3A	4.188751	4.836319	2.11E-56	1.30E-54
CDC6	3.421125	4.36997	2.13E-56	1.31E-54
AC011899.3	-2.18174	-1.74382	2.33E-56	1.43E-54
PLK1	3.220536	4.627022	2.48E-56	1.52E-54
DNASE2B	-2.9147	0.080915	2.59E-56	1.58E-54
KIF2C	3.57357	4.472236	3.09E-56	1.88E-54
GP9	-3.10019	-2.78735	4.04E-56	2.45E-54
PLAC9P1	-2.68317	-3.64706	4.16E-56	2.52E-54
CDC20	3.774671	5.04975	4.53E-56	2.74E-54

For Peer Review

ZBED2	-2.37437	2.694865	5.91E-56	3.57E-54
EXO1	3.594551	3.192086	1.41E-55	8.46E-54
ACKR4	-2.44101	0.798415	1.52E-55	9.09E-54
EFNA4	2.181346	4.254234	1.59E-55	9.48E-54
C2orf91	-2.91907	-0.77483	1.76E-55	1.05E-53
MMP19	-2.03718	4.593873	2.22E-55	1.31E-53
ANLN	3.7892	5.500627	2.24E-55	1.32E-53
DEPDC1B	3.594803	2.883503	2.26E-55	1.33E-53
MELK	3.615206	3.893269	2.59E-55	1.52E-53
CELA2B	-2.1845	-2.05519	2.87E-55	1.67E-53
CTHRC1	3.764725	6.070735	4.52E-55	2.63E-53
GINS1	3.034064	3.880069	4.56E-55	2.64E-53
AL135960.1	-2.54238	-1.6557	4.60E-55	2.66E-53
CTXND1	-3.11797	1.361457	5.65E-55	3.26E-53
KCNA5	-2.30296	-0.0893	1.15E-54	6.60E-53
KIF18B	3.770584	3.620091	1.28E-54	7.36E-53
FOSB	-3.29646	7.540958	1.31E-54	7.50E-53
AC073585.1	3.685938	-0.02933	1.74E-54	9.91E-53
RAD54L	3.276281	2.573815	1.83E-54	1.04E-52
CDCA8	3.04614	4.211512	1.84E-54	1.05E-52
RECQL4	3.019951	4.520634	2.06E-54	1.17E-52
AC025048.1	-2.53483	-3.71028	2.22E-54	1.26E-52
CXCL2	-2.43211	5.705702	2.58E-54	1.46E-52
AC119424.1	-3.43816	-3.01857	3.36E-54	1.89E-52
TROAP	3.828742	3.724624	3.83E-54	2.16E-52
DEPDC1	3.828767	3.397897	4.36E-54	2.45E-52
DUOX1	-2.57171	6.007397	6.09E-54	3.41E-52
SGO1	3.208325	2.130396	6.44E-54	3.60E-52
FGFR4	-2.47552	4.433957	6.57E-54	3.66E-52
SCN7A	-2.41643	4.583847	7.53E-54	4.20E-52
AC068700.1	-2.35734	-1.33138	7.60E-54	4.23E-52
LINC00511	3.06302	4.502135	9.06E-54	5.03E-52
PIP5K1B	-2.25262	3.567691	1.15E-53	6.39E-52
AC026992.2	-2.75698	-0.03966	1.88E-53	1.04E-51
MND1	2.886872	1.668602	2.80E-53	1.54E-51
CEP55	3.251365	4.461525	4.10E-53	2.24E-51
RMI2	2.279257	3.85557	4.37E-53	2.39E-51
C1QTNF7	-2.4522	2.413503	4.74E-53	2.58E-51
TPX2	3.645262	5.752	5.06E-53	2.75E-51
GUCY1A2	-2.02367	4.010295	6.67E-53	3.62E-51
EFNA3	3.053132	2.500453	6.94E-53	3.76E-51
SPTBN2	2.510547	5.718528	8.07E-53	4.35E-51
SKA3	3.194331	2.755719	8.15E-53	4.39E-51

For Peer Review

AFAP1-AS1	6.334651	6.09258	8.77E-53	4.71E-51
SLC1A1	-2.21059	4.746499	9.98E-53	5.35E-51
BIRC5	3.683127	5.273538	1.24E-52	6.65E-51
ECEL1P2	-3.18294	1.094428	1.26E-52	6.76E-51
MRC1	-2.33732	7.039615	1.34E-52	7.13E-51
AL162595.1	2.324439	0.166119	1.52E-52	8.06E-51
FFAR4	-2.39559	1.981293	1.56E-52	8.28E-51
UBE2C	4.187299	5.192012	1.71E-52	9.02E-51
CCNB1	2.97385	5.289668	3.04E-52	1.59E-50
SIRPD	-2.1804	-2.42637	3.60E-52	1.88E-50
LINC00891	-2.44824	-1.51274	4.88E-52	2.53E-50
AL035409.1	-2.38562	-1.87161	4.88E-52	2.53E-50
PPP1R14B	2.078931	6.492353	5.20E-52	2.69E-50
Z82246.1	-3.38196	-3.48297	6.00E-52	3.10E-50
WFIKKN2	-3.04627	-1.2079	6.91E-52	3.56E-50
AC044810.2	-3.26554	-1.909	8.35E-52	4.30E-50
FUT2	3.182201	4.473144	9.52E-52	4.88E-50
GTSE1	3.102157	3.369342	1.05E-51	5.35E-50
CDCA3	3.154803	3.430124	1.37E-51	6.96E-50
TMPRSS4	4.817697	6.869461	1.37E-51	6.96E-50
FRMD5	3.550239	2.851311	1.44E-51	7.33E-50
KIF11	2.690004	4.64716	1.47E-51	7.47E-50
FIBIN	-2.14793	3.517519	1.61E-51	8.17E-50
LOXHD1	-2.21116	-0.51619	2.29E-51	1.15E-49
MNX1-AS1	5.132333	0.642935	2.37E-51	1.19E-49
CBLC	3.201557	4.238007	2.82E-51	1.41E-49
TMCO2	-2.5893	-3.80237	3.10E-51	1.55E-49
CCNB2	3.044127	4.259079	4.10E-51	2.04E-49
MT1M	-2.76353	2.456935	7.47E-51	3.70E-49
BRIP1	2.666474	2.882477	7.89E-51	3.90E-49
POLQ	3.271519	2.609027	9.15E-51	4.51E-49
ZWINT	2.602441	4.891707	1.32E-50	6.47E-49
LILRA5	-2.05323	2.353566	1.36E-50	6.66E-49
CKAP2L	3.155041	3.348952	1.59E-50	7.75E-49
KNTC1	2.088934	4.806379	1.62E-50	7.85E-49
OCIAD2	2.187095	6.748731	1.65E-50	8.01E-49
MCM10	3.536006	3.149126	2.03E-50	9.83E-49
PERM1	3.026912	1.60973	2.45E-50	1.19E-48
COL10A1	4.577809	5.881967	3.04E-50	1.46E-48
NUSAP1	2.561132	4.826363	3.05E-50	1.47E-48
AC093787.1	-4.00393	-3.33119	3.38E-50	1.62E-48
RRM2	3.224952	5.657068	3.60E-50	1.73E-48
EPHA10	3.620147	3.504656	4.11E-50	1.97E-48

For Peer Review

ABCA12	5.372969	3.652465	4.89E-50	2.33E-48
KRT79	-3.13505	-1.24145	6.62E-50	3.14E-48
CENPA	3.619279	2.620755	8.75E-50	4.15E-48
LINC01624	-2.30031	-2.61404	8.98E-50	4.25E-48
STX1A	3.055881	4.066076	1.03E-49	4.86E-48
AP001453.2	3.471048	0.539243	1.05E-49	4.96E-48
IGSF9	3.240335	4.747551	1.08E-49	5.08E-48
AC004816.1	2.89876	1.984615	1.38E-49	6.48E-48
SLC6A13	-2.6562	-1.00701	1.41E-49	6.62E-48
CCL14	-2.45859	0.973324	1.47E-49	6.91E-48
MFAP3L	-2.10321	2.416485	1.53E-49	7.17E-48
FERMT1	3.231558	4.908072	1.79E-49	8.34E-48
SVEP1	-2.10608	4.930367	2.02E-49	9.41E-48
RASAL1	4.018262	3.406466	2.58E-49	1.20E-47
PVT1	2.693801	3.811988	3.10E-49	1.44E-47
ANGPTL5	-2.49016	-1.83192	4.21E-49	1.95E-47
GPER1	-2.21777	2.2153	4.34E-49	2.00E-47
IQANK1	2.265904	5.233862	5.07E-49	2.34E-47
GHR	-2.05409	1.648525	6.12E-49	2.81E-47
NME1	2.192681	5.894444	6.62E-49	3.03E-47
ARMH2	-3.47396	-2.77754	7.60E-49	3.48E-47
NDC80	3.125871	3.50398	8.36E-49	3.82E-47
AC018529.1	-2.54148	-2.83993	8.77E-49	4.00E-47
PADI4	-2.53351	-1.08707	9.89E-49	4.50E-47
EME1	2.745798	2.055541	1.21E-48	5.51E-47
AURKB	3.445216	3.740482	1.25E-48	5.66E-47
TTK	3.289948	3.325941	1.30E-48	5.89E-47
AC027288.2	-3.04634	-0.89293	1.35E-48	6.10E-47
CYP3A7	-2.15779	-0.40968	1.39E-48	6.27E-47
CDC45	3.154676	3.336352	1.43E-48	6.46E-47
HELLS	2.344256	3.891908	1.74E-48	7.84E-47
PTPRH	5.186634	3.985624	1.75E-48	7.86E-47
HMMR	3.041155	3.847743	1.98E-48	8.89E-47
HMGB3	3.490452	8.099616	2.52E-48	1.13E-46
AC091305.1	-3.09598	-3.16551	2.57E-48	1.15E-46
MKI67	3.1103	6.190367	2.58E-48	1.15E-46
GPT2	3.408178	6.1011	2.58E-48	1.15E-46
AC116312.1	-2.78979	-3.51217	3.19E-48	1.42E-46
ZYG11A	5.316689	1.698747	3.24E-48	1.44E-46
PCLAF	2.823815	4.049447	4.48E-48	1.98E-46
CELSR3	3.366681	4.066717	4.77E-48	2.11E-46
AC140479.2	4.073439	0.609984	5.02E-48	2.21E-46
FGF10	-2.9691	-0.44352	5.05E-48	2.22E-46

For Peer Review

PLPP2	2.416368	6.377333	5.38E-48	2.37E-46
FOXM1	3.378987	5.078008	5.55E-48	2.44E-46
BCHE	-2.47965	2.112672	6.25E-48	2.74E-46
RHBDL2	2.670477	3.333457	6.94E-48	3.04E-46
PKMYT1	2.983253	3.489354	8.21E-48	3.59E-46
RADIL	-2.23805	1.977086	9.39E-48	4.10E-46
EDN1	-2.03001	5.048209	1.02E-47	4.44E-46
AQP4	-2.97562	7.182226	1.09E-47	4.75E-46
NCAPG	3.211375	3.853534	1.14E-47	4.93E-46
EPN3	3.068651	4.225152	1.35E-47	5.87E-46
NCAPH	3.012927	3.734326	1.75E-47	7.59E-46
CRABP2	5.585409	7.439462	1.88E-47	8.13E-46
PARAL1	-3.01406	0.494794	2.23E-47	9.60E-46
FGF2	-2.05002	2.556607	2.74E-47	1.17E-45
RNU5B-4P	-2.086	-3.48521	3.11E-47	1.33E-45
PSAT1	3.490256	4.73862	5.40E-47	2.30E-45
AC112206.2	-2.69519	-0.35667	6.10E-47	2.60E-45
CDCA2	2.95668	2.530078	6.72E-47	2.87E-45
DDX11-AS1	2.416302	-0.29013	8.66E-47	3.68E-45
PRC1	2.52282	4.973095	1.02E-46	4.32E-45
DNA2	2.213461	3.07688	1.03E-46	4.39E-45
ADCYAP1R1	-2.13881	-0.03098	1.19E-46	5.04E-45
RETN	-2.95354	1.792423	1.24E-46	5.27E-45
SLC24A4	-2.04039	0.674726	1.45E-46	6.15E-45
RDM1	3.550406	-0.23192	1.46E-46	6.18E-45
NDRG4	-2.05145	3.612682	1.61E-46	6.78E-45
OTX1	3.329502	2.552775	1.96E-46	8.27E-45
ZFPM2-AS1	5.319243	1.816918	2.01E-46	8.45E-45
KCNIP1	-2.13642	-0.91447	2.34E-46	9.86E-45
FOLR3	-3.30553	0.128666	2.58E-46	1.08E-44
CAV3	-3.07722	-2.31327	2.75E-46	1.15E-44
KDELR3	2.228762	5.64577	3.71E-46	1.55E-44
ERICH4	-2.29634	-3.08407	4.16E-46	1.74E-44
GREM1	4.998412	5.866238	4.25E-46	1.77E-44
AC020907.1	5.763109	0.136969	4.79E-46	1.99E-44
TICRR	2.912704	2.449784	4.97E-46	2.06E-44
CACNA1S	-3.34378	-1.72511	5.16E-46	2.14E-44
RUNDC3A-AS1	3.709582	0.675254	6.65E-46	2.75E-44
SLC5A9	-2.56616	1.75902	6.82E-46	2.82E-44
KNL1	2.605621	3.346767	7.43E-46	3.06E-44
C17orf53	2.731854	2.666311	9.27E-46	3.82E-44
CCNA2	2.900282	4.293767	1.11E-45	4.58E-44

For Peer Review

MDK	3.072999	8.225148	1.13E-45	4.64E-44
SBSPON	-2.31035	2.52857	1.63E-45	6.66E-44
OVCH2	-2.99043	-1.01177	1.80E-45	7.33E-44
FER1L4	4.152154	4.189414	1.80E-45	7.34E-44
CDCA5	2.980099	4.451909	1.81E-45	7.37E-44
UNC5CL	3.093578	3.944265	1.85E-45	7.51E-44
CENPI	2.722536	2.361259	2.03E-45	8.22E-44
KIF15	2.680084	2.969525	2.29E-45	9.25E-44
ESPL1	3.120483	3.844443	2.58E-45	1.04E-43
NECTIN4	2.346822	6.00553	3.43E-45	1.37E-43
ACKR1	-2.39661	4.18118	3.93E-45	1.57E-43
GPRIN1	2.703893	3.314717	4.03E-45	1.61E-43
AL390719.1	2.826342	2.49573	4.32E-45	1.72E-43
PCDH15	-3.09718	0.116219	4.34E-45	1.73E-43
RGS17	3.682918	3.470521	4.51E-45	1.79E-43
FAM111B	2.727314	3.515488	4.61E-45	1.83E-43
AC068228.1	5.390346	-1.65792	4.68E-45	1.86E-43
POLE2	2.330177	2.595351	4.68E-45	1.86E-43
PPP1R14BP3	2.040913	3.817663	5.50E-45	2.17E-43
LINC01270	2.866191	1.662168	6.07E-45	2.39E-43
MME	-2.49499	4.798394	6.53E-45	2.57E-43
EXD1	-2.413	-2.27044	7.07E-45	2.78E-43
GINS2	2.837475	3.51837	7.22E-45	2.84E-43
NEIL3	4.257131	2.232203	8.06E-45	3.16E-43
RAB26	3.611234	1.872305	8.94E-45	3.50E-43
MCM4	2.207713	6.423205	9.23E-45	3.61E-43
IL33	-2.11645	5.184166	1.17E-44	4.56E-43
SPC24	2.66001	3.051176	1.52E-44	5.90E-43
HLX-AS1	-2.29275	-3.28037	1.75E-44	6.79E-43
AFF3	-2.40275	3.443975	1.85E-44	7.16E-43
PAFAH1B3	2.224178	5.116181	1.97E-44	7.58E-43
Z98257.1	6.026836	3.356784	2.14E-44	8.24E-43
HIST1H2BD	2.614465	4.326023	2.35E-44	9.03E-43
TRAPPC3L	-2.05957	-2.70466	2.36E-44	9.05E-43
PCAT6	2.699726	2.14691	2.61E-44	9.99E-43
NTM	-2.11363	4.420798	3.90E-44	1.49E-42
SGCA	-2.03114	2.433408	4.23E-44	1.61E-42
OR52K3P	-2.18012	-1.32814	6.40E-44	2.42E-42
AC080037.1	5.229815	0.708007	7.02E-44	2.65E-42
MYBL2	3.819422	5.629313	8.25E-44	3.10E-42
ONECUT1	6.504004	-0.12013	9.59E-44	3.59E-42
SLC25A10	2.200417	4.103855	1.15E-43	4.27E-42
HBG2	-2.49853	-2.67365	1.26E-43	4.67E-42

For Peer Review

TONSL	2.120428	4.443868	1.28E-43	4.76E-42
ESCO2	2.616617	2.339104	1.30E-43	4.82E-42
SLC2A1	3.514232	7.414953	1.47E-43	5.42E-42
AC147067.2	-2.02573	-2.22488	1.50E-43	5.53E-42
HS6ST2	3.69159	5.416646	1.56E-43	5.77E-42
MIR3677	-2.41781	-2.49435	1.75E-43	6.44E-42
AL049634.1	-2.22511	-2.84285	1.98E-43	7.26E-42
ORC1	2.741358	2.815158	2.11E-43	7.75E-42
MGC27382	-3.02791	-0.3899	2.43E-43	8.89E-42
TSACC	3.027987	-0.27301	2.50E-43	9.13E-42
AC069360.1	-2.25899	-1.94702	2.81E-43	1.02E-41
KL	-2.32491	2.475164	3.67E-43	1.33E-41
HSPB3	-2.52992	-1.67299	3.77E-43	1.37E-41
PACRG-AS3	-3.60318	-2.73875	4.25E-43	1.54E-41
AL357146.1	-2.76234	-3.51865	5.23E-43	1.89E-41
ADTRP	-2.12266	2.670795	6.00E-43	2.16E-41
MNX1	4.269933	1.319753	6.10E-43	2.19E-41
CYP1A2	-3.84203	-1.11249	6.64E-43	2.38E-41
ATP1A2	-2.30421	1.665794	7.06E-43	2.53E-41
ASF1B	2.364032	4.184726	7.88E-43	2.82E-41
FEZF1-AS1	6.121836	2.844023	7.97E-43	2.85E-41
ADAMTS9-AS1	-2.43085	0.534025	9.74E-43	3.48E-41
CENPU	2.375763	3.93199	1.02E-42	3.64E-41
GJB2	4.561231	5.156729	1.03E-42	3.68E-41
AC004947.2	-2.31898	-1.19357	1.59E-42	5.63E-41
HMGB3P10	-2.30012	-3.34914	1.66E-42	5.88E-41
HEMGN	-2.43347	-3.15521	2.01E-42	7.10E-41
PBK	3.388319	3.198271	2.21E-42	7.81E-41
LINC01572	2.912922	-0.425	2.23E-42	7.87E-41
AGMAT	2.599148	1.774873	2.88E-42	1.01E-40
DAO	-2.43453	-3.10105	3.06E-42	1.08E-40
SKA1	3.027309	2.494861	3.76E-42	1.32E-40
FOXD3-AS1	6.847475	0.206278	4.74E-42	1.66E-40
LINC01645	-2.67871	-2.61196	5.99E-42	2.10E-40
BUB1	2.64837	4.53753	6.05E-42	2.12E-40
SALL4	3.378839	1.345004	6.46E-42	2.26E-40
NIM1K	-2.26406	1.105918	6.99E-42	2.43E-40
KISS1R	6.114652	0.821173	7.51E-42	2.61E-40
GRHL3	3.516919	1.805294	8.35E-42	2.90E-40
LINC00857	2.260995	2.014027	1.03E-41	3.56E-40
C1orf140	-2.56777	-3.34246	1.05E-41	3.63E-40
CHEK1	2.227802	4.004225	1.06E-41	3.66E-40

For Peer Review

TK1	2.648592	5.853346	1.10E-41	3.77E-40
CDT1	2.732462	3.997121	1.15E-41	3.96E-40
PLEK2	2.723928	4.212688	1.19E-41	4.07E-40
ECT2	2.161979	5.649261	1.29E-41	4.41E-40
AC007182.1	-2.67408	-2.81469	1.34E-41	4.58E-40
KIFC1	2.745244	4.595286	1.34E-41	4.59E-40
AL355304.1	-2.35525	-2.84428	1.37E-41	4.67E-40
LINC02570	-3.42209	-3.00511	1.43E-41	4.89E-40
CCNE1	3.287394	3.143927	1.48E-41	5.03E-40
SPP1	4.789238	9.217599	2.08E-41	7.06E-40
LINC02416	4.192293	-1.19709	2.31E-41	7.82E-40
MMP11	5.244073	6.583765	2.35E-41	7.95E-40
CENPE	2.638854	3.582144	2.37E-41	8.00E-40
TFR2	3.082804	1.847822	2.41E-41	8.14E-40
AL591686.1	-3.00295	-3.26553	2.61E-41	8.80E-40
ATP10B	5.477725	3.969437	2.74E-41	9.21E-40
BLM	2.072119	3.071574	2.76E-41	9.28E-40
LIPN	-2.55563	-2.02236	3.24E-41	1.09E-39
TFAP2A	3.736941	3.951384	3.31E-41	1.11E-39
ARSH	5.030287	-1.0968	4.28E-41	1.43E-39
AC112777.1	3.261842	-0.63924	5.81E-41	1.93E-39
IL1RL1	-2.87176	3.674786	5.90E-41	1.96E-39
MIR27A	-2.44911	-3.17158	5.91E-41	1.96E-39
ITIH5	-2.10871	4.629362	6.11E-41	2.03E-39
KIF23	2.580392	4.150808	6.33E-41	2.10E-39
CAMP	-2.63866	-0.20997	6.64E-41	2.20E-39
FCGR3B	-2.36831	1.907813	6.95E-41	2.30E-39
AURKA	2.637594	4.503735	7.07E-41	2.34E-39
PIMREG	3.171505	2.663537	7.28E-41	2.41E-39
AC004540.2	-2.01018	-0.49487	7.97E-41	2.63E-39
AC011239.2	-2.36875	-2.51844	8.38E-41	2.75E-39
CLDN18	-3.54814	7.652669	1.08E-40	3.53E-39
MSL3P1	2.76839	-0.20742	1.09E-40	3.56E-39
LHFPL5	4.953953	-1.42926	1.34E-40	4.35E-39
ODAM	-3.14079	-0.52717	1.37E-40	4.43E-39
C1QTNF6	2.270758	4.888545	1.91E-40	6.18E-39
ANKRD29	-2.18397	4.557129	2.05E-40	6.58E-39
CHRDL1	-2.32244	4.798304	3.05E-40	9.76E-39
PRSS35	-2.23711	-0.01808	3.27E-40	1.04E-38
BET1P1	-2.31054	-3.66167	3.88E-40	1.23E-38
LINC01070	-3.38821	-3.97548	4.17E-40	1.33E-38
VWC2	-2.53668	-1.91875	4.35E-40	1.38E-38
AC145343.1	3.0476	0.232687	4.47E-40	1.42E-38

BCL2L15	3.505709	3.759033	5.08E-40	1.61E-38
PITX2	8.080194	2.854209	5.40E-40	1.71E-38
LINC01165	-2.42911	-3.32934	5.44E-40	1.72E-38
DCSTAMP	-2.22099	1.368918	5.77E-40	1.82E-38
CRTAC1	-2.61311	5.497071	1.11E-39	3.44E-38
LINC02104	-2.12463	-2.70971	1.24E-39	3.86E-38
VWFP1	-2.29525	-0.64021	1.35E-39	4.18E-38
SLC24A2	4.998676	0.239231	1.36E-39	4.22E-38
SHCBP1	2.278841	3.430439	2.01E-39	6.21E-38
ST13P20	-2.64904	-3.62774	2.02E-39	6.25E-38
RBP2	-2.88684	-0.32435	2.40E-39	7.39E-38
ERO1A	2.025589	7.789654	2.63E-39	8.10E-38
CCDC150	2.571435	1.247901	2.81E-39	8.61E-38
COL11A1	6.191955	6.072875	2.87E-39	8.80E-38
AC244153.1	-2.48863	0.163913	3.23E-39	9.89E-38
MAD2L1	2.556718	4.254777	3.55E-39	1.09E-37
PCOLCE2	-2.36991	3.369396	3.81E-39	1.16E-37
C5orf34	2.001146	1.946078	5.80E-39	1.76E-37
RHBG	4.551645	0.064159	5.84E-39	1.77E-37
MAST1	3.230415	1.105991	5.88E-39	1.78E-37
DDX12P	2.375887	1.62257	6.81E-39	2.06E-37
STYK1	2.588243	3.246226	6.90E-39	2.08E-37
GCNT3	5.145412	5.63327	7.82E-39	2.36E-37
LBX2	2.078159	0.529962	8.27E-39	2.48E-37
PPP1R14D	5.956905	3.69456	1.01E-38	3.01E-37
XDH	4.042392	4.472244	1.20E-38	3.58E-37
CDKN3	2.879845	3.142004	1.23E-38	3.69E-37
ENTPD8	4.638323	2.312774	1.26E-38	3.75E-37
NR4A3	-2.27341	4.324371	1.28E-38	3.82E-37
DGCR5	3.64663	2.907809	1.70E-38	5.05E-37
CLSPN	2.720505	2.858344	1.71E-38	5.07E-37
SYT12	5.476805	4.852751	1.72E-38	5.11E-37
CHRM2	-3.43331	-1.154	2.02E-38	5.98E-37
LINC00844	-2.83293	-3.56803	2.27E-38	6.69E-37
P3H4	2.068117	5.309475	2.29E-38	6.73E-37
ART4	-2.25523	1.046447	2.36E-38	6.93E-37
PROM2	2.612402	6.401309	2.56E-38	7.51E-37
CDCA7	2.909933	4.651279	2.60E-38	7.61E-37
CENPK	2.209213	2.72723	3.08E-38	9.02E-37
AC090796.1	-2.86252	-2.47618	3.16E-38	9.25E-37
ABCC3	2.530595	7.88989	3.66E-38	1.07E-36
DMBX1	6.315321	0.684815	4.13E-38	1.20E-36
RHBDL1	3.179117	2.355681	4.17E-38	1.21E-36

For Peer Review

P2RY6	2.992491	4.155624	4.90E-38	1.42E-36
AP002856.2	-4.07778	-1.28544	5.55E-38	1.61E-36
ITPKA	5.154605	3.750179	5.60E-38	1.62E-36
ABCA3	-2.11923	8.396717	5.93E-38	1.72E-36
CYP24A1	6.711262	6.528527	6.04E-38	1.75E-36
HNF4G	3.471549	3.078032	6.22E-38	1.80E-36
CCL24	-2.48056	1.027172	6.71E-38	1.94E-36
LINC01985	-2.56089	-3.53477	7.01E-38	2.02E-36
VPS9D1-AS1	2.833285	2.277657	7.13E-38	2.05E-36
E2F2	2.334804	2.76151	7.67E-38	2.20E-36
PTTG1	2.327322	4.51205	7.96E-38	2.28E-36
HHIP	-2.89965	4.823145	8.98E-38	2.57E-36
CDK1	2.449391	5.038544	9.15E-38	2.61E-36
LYPD1	4.073983	2.459626	1.04E-37	2.98E-36
U62317.1	2.842903	1.854571	1.08E-37	3.09E-36
AC026992.1	-2.35228	-2.29013	1.11E-37	3.15E-36
PCARE	-2.98609	-0.97161	1.15E-37	3.27E-36
STAC	-2.08916	3.303446	1.15E-37	3.27E-36
SYT7	3.438406	6.006555	1.23E-37	3.48E-36
MIR4653	3.193408	-1.69601	1.24E-37	3.51E-36
AC011294.1	4.82463	0.494537	1.35E-37	3.81E-36
ADAMTS14	2.642199	2.831646	1.46E-37	4.12E-36
SEMA6D	-2.07199	2.995056	1.50E-37	4.23E-36
AC005884.1	-2.26709	-2.78172	2.35E-37	6.60E-36
LINC01703	2.337525	-0.4688	2.46E-37	6.91E-36
LINC00211	-2.27686	-3.2162	2.47E-37	6.93E-36
TERT	5.358456	0.021264	2.53E-37	7.08E-36
AC112722.1	-2.08607	-1.53877	2.63E-37	7.35E-36
CA3	-2.16692	1.952941	3.36E-37	9.35E-36
TFAP2A-AS1	3.042692	-0.5224	3.47E-37	9.66E-36
RAD51	2.196317	2.723326	3.86E-37	1.07E-35
ECE2	2.081586	3.634996	4.21E-37	1.17E-35
HPSE2	-2.4214	0.573711	4.92E-37	1.36E-35
FGF10-AS1	-3.09372	-3.80374	6.87E-37	1.90E-35
MYEOV	6.287193	4.308895	7.01E-37	1.94E-35
PODXL2	3.238919	5.604234	7.37E-37	2.03E-35
AC134312.1	-2.51547	-2.59348	8.26E-37	2.27E-35
GINS4	2.41291	2.787723	8.93E-37	2.45E-35
SPC25	2.363461	2.223464	1.10E-36	3.00E-35
INAVA	2.192863	5.389686	1.11E-36	3.04E-35
LINC01412	-2.1624	-3.54543	1.35E-36	3.68E-35
C5orf64	-2.05932	-3.21028	1.35E-36	3.68E-35
CDC37P2	3.762637	-1.69626	1.42E-36	3.85E-35

For Peer Review

DDN-AS1	2.51414	-0.03596	1.61E-36	4.35E-35
PLK4	2.032652	3.101855	1.71E-36	4.60E-35
CHIAP2	-3.70623	1.723343	1.79E-36	4.81E-35
KCNN4	3.242719	5.694502	1.80E-36	4.83E-35
AL390036.1	-2.07248	-2.21386	1.80E-36	4.83E-35
AC090502.1	6.790674	-0.03882	1.91E-36	5.11E-35
WNT3	2.058575	3.368027	2.60E-36	6.93E-35
MT1JP	-2.89633	-2.64354	3.60E-36	9.53E-35
SERINC2	2.042557	7.558837	5.64E-36	1.49E-34
B4GALNT4	4.740499	3.466404	7.02E-36	1.84E-34
CABYR	4.632428	3.641609	7.81E-36	2.04E-34
GPA33	-2.73537	2.169218	8.19E-36	2.14E-34
CDC25A	2.343813	2.376132	9.06E-36	2.36E-34
CCDC54	-2.09466	-3.30827	9.39E-36	2.45E-34
GPC3	-2.17107	5.436169	1.18E-35	3.08E-34
WASIR2	3.386929	-0.81984	1.19E-35	3.09E-34
AC133785.1	6.618831	-0.65685	1.28E-35	3.32E-34
BLACAT1	2.926983	2.285236	1.39E-35	3.60E-34
DCST1	2.626327	-0.07921	1.59E-35	4.09E-34
TPPP3	-2.21582	5.957919	1.61E-35	4.15E-34
TMPRSS11E	6.675563	3.684867	1.63E-35	4.20E-34
KIAA0408	-2.43017	-0.90689	1.66E-35	4.25E-34
IL1RAPL2	6.106758	-0.95981	2.27E-35	5.83E-34
AC015921.1	3.109661	-0.75209	2.49E-35	6.38E-34
LINC02535	4.328196	-0.70512	2.94E-35	7.47E-34
LINC01271	2.549657	-0.68495	3.30E-35	8.37E-34
AC105219.3	2.652075	0.180486	3.43E-35	8.67E-34
AL365356.5	5.058523	-0.88539	3.85E-35	9.70E-34
STRA6	4.458934	3.930724	4.82E-35	1.21E-33
MOGAT1	-2.57715	-3.06151	7.32E-35	1.83E-33
GALNT14	3.671704	4.318698	7.90E-35	1.97E-33
HSD17B6	-2.26733	5.035612	7.95E-35	1.98E-33
F12	2.825544	2.3507	8.03E-35	2.00E-33
IL1RL2	2.269229	1.719937	8.90E-35	2.21E-33
THBS2	2.889121	7.677132	1.15E-34	2.85E-33
RNU6-917P	-2.33458	-3.98337	1.33E-34	3.29E-33
AL359378.1	-2.9627	-3.81112	1.33E-34	3.30E-33
DNAJC22	3.443608	3.319944	1.39E-34	3.43E-33
SLCO5A1	3.179313	2.048021	1.60E-34	3.94E-33
PLXNB3	3.381516	4.398154	1.63E-34	4.02E-33
AGBL1	-3.03974	-2.64113	1.64E-34	4.02E-33
AC105046.1	-2.33996	-2.01925	1.85E-34	4.55E-33
LCN6	-2.46988	-2.29556	1.91E-34	4.68E-33

For Peer Review

CCDC173	-2.08949	0.918875	2.08E-34	5.10E-33
RSPO2	-2.63048	1.242783	2.12E-34	5.19E-33
CNTD2	4.296858	2.011183	2.16E-34	5.28E-33
PARPBP	2.087798	2.677395	2.35E-34	5.73E-33
CGREF1	4.10626	2.824398	3.65E-34	8.86E-33
CPNE7	3.184731	2.934315	3.67E-34	8.89E-33
AC004490.1	-2.04249	-2.38721	3.67E-34	8.89E-33
MCM2	2.008328	5.724109	3.73E-34	9.01E-33
AC007128.1	5.401874	-0.20571	4.04E-34	9.75E-33
C1orf220	2.253928	-0.44269	4.12E-34	9.93E-33
HTR3A	5.863172	3.071135	4.19E-34	1.01E-32
AC092691.1	-2.97146	-2.35264	4.20E-34	1.01E-32
CARD14	3.450167	3.296462	4.80E-34	1.15E-32
TDRKH-AS1	2.01337	0.159234	5.10E-34	1.22E-32
AF131215.7	-2.23991	-2.36467	5.13E-34	1.23E-32
RSPO4	-2.32965	1.700653	5.17E-34	1.24E-32
OIP5	2.368713	1.360894	5.19E-34	1.24E-32
AL391056.1	4.303732	1.654825	5.74E-34	1.37E-32
PTGES	2.985747	5.407941	8.17E-34	1.94E-32
AC053503.6	-2.76647	-3.86922	9.02E-34	2.14E-32
AC104257.1	-3.34301	-3.30629	1.08E-33	2.55E-32
SFTPA1	-2.83293	12.03661	1.15E-33	2.72E-32
C2CD4A	4.094806	3.964748	1.27E-33	2.99E-32
EEF1A2	6.369795	5.928759	1.41E-33	3.32E-32
CILP2	3.470533	3.572913	1.43E-33	3.34E-32
AC105384.1	-2.05227	-2.71604	1.77E-33	4.14E-32
GPR17	-2.0902	-0.25161	1.95E-33	4.54E-32
LINC01426	2.497217	2.396469	1.97E-33	4.58E-32
NMU	4.569436	2.403551	2.00E-33	4.66E-32
AC100793.4	-2.21884	-2.80177	2.08E-33	4.84E-32
SNORC	2.470921	0.754493	2.23E-33	5.19E-32
DEFA3	-3.48473	-3.11083	2.35E-33	5.46E-32
ALG1L	3.275479	2.672973	3.03E-33	7.00E-32
ZNF695	4.366254	0.217544	3.11E-33	7.19E-32
CHRNA5	3.600482	2.311563	3.13E-33	7.23E-32
AL365356.4	4.385452	-1.10964	3.30E-33	7.61E-32
ANKRD22	2.774439	4.171543	3.37E-33	7.76E-32
LINC01616	-3.1787	-3.52869	3.46E-33	7.96E-32
ADAM12	3.073825	4.760158	3.59E-33	8.25E-32
BDNF	-2.34179	1.679005	3.66E-33	8.40E-32
FEZF1	5.58064	1.813116	3.89E-33	8.92E-32
AUNIP	2.341549	1.422565	4.05E-33	9.26E-32
SEC14L6	-2.09554	3.13618	4.08E-33	9.33E-32

For Peer Review

AP003559.1	3.502906	-0.36077	4.71E-33	1.07E-31
TRPM8	7.012282	3.274614	4.71E-33	1.07E-31
TCEAL2	-2.52605	0.81192	5.01E-33	1.14E-31
HMGA1	2.29403	8.020737	5.55E-33	1.26E-31
BBOX1-AS1	5.49827	0.857251	6.13E-33	1.39E-31
PITX1	4.534473	3.914265	6.42E-33	1.45E-31
FGF11	3.192164	0.179252	6.53E-33	1.47E-31
DNAJC12	4.232893	4.584831	6.59E-33	1.49E-31
CYP27B1	2.331136	2.866143	6.69E-33	1.51E-31
CACNA2D2	-2.40036	6.47491	7.17E-33	1.61E-31
CCDC169	2.91918	0.629559	7.89E-33	1.77E-31
WDR62	2.341257	3.632528	8.05E-33	1.81E-31
YBX2	4.729227	2.463927	9.10E-33	2.04E-31
AC245041.2	-2.54594	1.466117	9.85E-33	2.20E-31
HSF2BP	2.216209	-0.09076	1.10E-32	2.45E-31
KC877392.1	-2.36349	-3.80484	1.16E-32	2.59E-31
SYNDIG1L	-2.38574	0.394416	1.19E-32	2.65E-31
PCMTD1P3	4.18794	-2.01844	1.39E-32	3.09E-31
ALG1L2	2.005361	-0.46716	1.97E-32	4.36E-31
WFDC3	3.912539	3.387107	1.98E-32	4.38E-31
SPRED3	2.465337	1.756671	2.02E-32	4.47E-31
TMEM63C	3.998764	3.511011	2.13E-32	4.70E-31
TRIP13	2.531629	4.398278	2.15E-32	4.73E-31
SNHG4	2.18108	1.314427	2.27E-32	5.00E-31
AL161740.1	-3.18263	-3.79425	2.30E-32	5.05E-31
ARHGAP11A	2.054068	4.092594	2.30E-32	5.05E-31
AC026120.1	4.358916	-1.56042	2.35E-32	5.16E-31
CCNE2	2.002942	2.379877	2.41E-32	5.28E-31
C2CD4D	2.526295	0.928648	2.41E-32	5.29E-31
PIF1	2.337915	1.976909	2.48E-32	5.43E-31
AC009065.2	3.776547	0.980553	3.17E-32	6.93E-31
AC015849.5	3.601528	-0.86449	3.66E-32	7.98E-31
AC025271.4	-2.13703	-1.58189	4.02E-32	8.73E-31
LINC01614	3.617408	0.879583	4.14E-32	8.98E-31
NAV2-AS2	-2.55203	-2.192	4.69E-32	1.01E-30
PRR19	2.165406	1.026009	5.07E-32	1.09E-30
RBBP8NL	2.090749	2.318697	5.11E-32	1.10E-30
LINC00896	3.316841	-0.2678	5.20E-32	1.12E-30
NRG3	-2.42667	-0.74044	5.20E-32	1.12E-30
KRT80	2.447519	5.915051	5.44E-32	1.17E-30
PRR11	2.260019	4.07147	5.82E-32	1.25E-30
AC084864.1	3.202495	-2.59913	5.92E-32	1.27E-30
FHL2	2.32823	5.958126	6.18E-32	1.32E-30

For Peer Review

GPR19	2.410621	0.177772	7.03E-32	1.50E-30
AC016205.1	3.781812	-0.21546	7.50E-32	1.60E-30
LARGE2	2.466169	5.082536	7.88E-32	1.68E-30
SERPINA2	-3.11483	-3.97445	8.03E-32	1.71E-30
CASKIN1	3.646028	0.131642	8.12E-32	1.72E-30
SCN8A	2.905725	2.221256	8.78E-32	1.86E-30
ARHGAP40	3.671753	3.343408	8.94E-32	1.89E-30
TAS2R2P	5.205481	-2.42245	9.65E-32	2.04E-30
AC027228.2	3.828225	0.69371	1.01E-31	2.13E-30
SLC7A5	2.665809	7.016029	1.05E-31	2.22E-30
SPDEF	3.277413	5.320429	1.06E-31	2.24E-30
CP	3.482432	8.515961	1.33E-31	2.80E-30
MROH6	2.444601	5.410265	1.48E-31	3.10E-30
SYN2	-2.31678	0.159293	1.54E-31	3.22E-30
AL645922.1	-2.23123	-3.63371	1.67E-31	3.48E-30
LINC02332	3.880427	-2.55465	1.79E-31	3.72E-30
ADGRF4	4.485356	2.562416	2.14E-31	4.45E-30
AKR7A3	4.997695	3.285687	2.21E-31	4.58E-30
AC099850.3	2.340775	0.917321	2.53E-31	5.24E-30
SEC14L3	-3.09	0.248117	2.66E-31	5.50E-30
CADM3-AS1	-2.1392	-0.50198	2.69E-31	5.56E-30
PFKP	2.142956	7.328227	2.83E-31	5.84E-30
AGAP11	-2.05926	-2.54803	2.94E-31	6.06E-30
C2orf40	-2.55208	1.782113	3.04E-31	6.26E-30
ERVFRD-1	-2.13882	-1.11086	3.10E-31	6.39E-30
FAM83D	2.26561	3.81681	3.12E-31	6.42E-30
AC009065.5	3.877781	0.865762	3.27E-31	6.71E-30
TBX15	3.720542	3.362077	4.23E-31	8.64E-30
PPP1R17	-2.80923	-2.99998	4.35E-31	8.88E-30
STEAP1	2.814608	4.519911	4.38E-31	8.94E-30
AL133215.2	2.029615	-1.28123	4.38E-31	8.94E-30
FAM172BP	3.823501	-0.6616	4.87E-31	9.91E-30
AL049634.2	-2.24529	-3.86878	6.00E-31	1.22E-29
SLITRK2	-2.44994	-0.67862	6.39E-31	1.30E-29
PLPP4	4.077855	1.222962	6.47E-31	1.31E-29
AL157838.1	2.4075	0.802683	6.53E-31	1.32E-29
KCNK3	-2.20138	4.276627	6.82E-31	1.38E-29
TYMSOS	2.447395	-0.33322	7.07E-31	1.43E-29
MMP13	6.114791	4.952128	7.40E-31	1.49E-29
HHIPL2	6.018933	3.212614	8.35E-31	1.68E-29
GYG2	2.228828	3.601003	8.96E-31	1.80E-29
CEACAM1	2.056935	5.78014	9.56E-31	1.92E-29
AC092834.1	-2.84622	-3.44205	9.58E-31	1.92E-29

For Peer Review

AC120036.1	5.239947	-0.97296	9.89E-31	1.98E-29
P4HA3	2.217937	2.803601	1.05E-30	2.11E-29
AC025580.1	4.051198	1.894308	1.06E-30	2.11E-29
CYP4B1	-2.53115	7.261934	1.09E-30	2.17E-29
AC013268.3	3.624621	-0.9596	1.11E-30	2.21E-29
AL354719.2	3.960703	1.418734	1.12E-30	2.24E-29
AP003385.4	-2.70467	-2.58151	1.17E-30	2.33E-29
CLDN10-AS1	6.395875	0.690495	1.17E-30	2.33E-29
FBXO32	2.088814	5.974256	1.17E-30	2.34E-29
C11orf86	7.902762	1.529446	1.22E-30	2.43E-29
SBK1	2.552395	4.916551	1.24E-30	2.46E-29
BARX2	3.892921	3.460428	1.54E-30	3.07E-29
E2F7	2.729236	2.108821	1.60E-30	3.18E-29
TRGV6	-2.0692	-3.51617	1.69E-30	3.36E-29
LINC00862	3.761603	-0.51921	1.73E-30	3.44E-29
ADAM8	2.130397	6.02945	1.77E-30	3.50E-29
PGM5-AS1	-2.31143	-2.90928	1.77E-30	3.51E-29
HPDL	3.472114	0.988487	2.05E-30	4.04E-29
PLAC1	4.785323	-0.15486	2.21E-30	4.36E-29
AL365181.3	4.437372	3.613711	2.28E-30	4.49E-29
COL1A1	2.872166	11.50711	2.35E-30	4.62E-29
PRAME	7.551403	4.547308	2.86E-30	5.62E-29
NPSR1-AS1	5.813794	-1.47851	3.20E-30	6.26E-29
AC020663.2	2.494976	-1.0434	3.22E-30	6.31E-29
SLC2A1-AS1	2.33766	-0.13157	3.39E-30	6.63E-29
LINC01711	3.789324	-1.61368	3.43E-30	6.70E-29
AL645608.8	3.935694	-1.18023	3.74E-30	7.31E-29
AC026250.1	2.0793	-0.37869	3.77E-30	7.36E-29
SFTPA2	-2.67629	12.37169	4.23E-30	8.25E-29
BMPER	-2.23386	1.961255	4.27E-30	8.32E-29
RAD51AP1	2.060442	3.243417	5.13E-30	9.95E-29
AP001189.5	-2.16518	-3.7845	5.26E-30	1.02E-28
CA9	5.58279	4.320782	5.88E-30	1.14E-28
PHLDA2	2.310589	4.837824	7.54E-30	1.45E-28
PTX3	-2.0697	1.878201	7.71E-30	1.48E-28
AC002398.2	-2.3612	-3.79132	8.21E-30	1.57E-28
AC145207.9	2.384846	-1.3775	8.74E-30	1.67E-28
FBXO43	2.300637	-0.30743	9.12E-30	1.75E-28
FAM189A1	-2.16649	0.48943	9.52E-30	1.82E-28
GAD1	3.753645	1.84064	9.57E-30	1.83E-28
BEAN1	2.847677	3.273846	1.01E-29	1.92E-28
BAIAP2L2	3.684656	2.684972	1.25E-29	2.36E-28
MMP1	5.222361	6.816304	1.32E-29	2.50E-28

For Peer Review

FUT3	2.315841	4.852922	1.37E-29	2.59E-28
FAP	2.241278	4.440475	1.71E-29	3.22E-28
ONECUT2	4.917998	2.159595	1.75E-29	3.30E-28
AL121949.2	5.936151	0.172159	1.84E-29	3.46E-28
SLC22A18AS	2.446377	2.124138	1.88E-29	3.53E-28
CCDC178	-2.22974	-1.65013	1.95E-29	3.66E-28
RNU1-106P	2.968023	-1.66721	2.14E-29	4.00E-28
SRPK3	3.133294	2.474748	2.48E-29	4.63E-28
AL139412.1	4.187883	-1.15125	2.54E-29	4.74E-28
GSG1L	-2.4489	-2.65331	2.90E-29	5.38E-28
SPINK1	6.592539	7.585535	2.92E-29	5.42E-28
AC010776.2	-2.88444	-1.58995	2.94E-29	5.46E-28
AL136369.1	-3.1862	-2.78115	3.17E-29	5.87E-28
WISP1	2.161791	3.66149	3.80E-29	7.01E-28
ADAM28	2.259224	5.129613	3.95E-29	7.30E-28
AOC1	4.9711	5.309959	4.12E-29	7.61E-28
CST1	6.392259	4.351895	4.17E-29	7.68E-28
AL354714.3	-2.93752	-2.50923	4.24E-29	7.81E-28
Z84485.1	2.221884	-0.17241	4.31E-29	7.94E-28
AL135902.1	-2.36508	-4.01898	4.42E-29	8.14E-28
TMEM132A	2.036494	5.806065	4.51E-29	8.28E-28
SPP2	7.341345	0.324369	4.62E-29	8.49E-28
AP005233.2	6.032628	-0.35262	4.95E-29	9.07E-28
TRHDE	-2.64971	1.177461	5.29E-29	9.70E-28
GPC2	3.389306	1.418003	5.44E-29	9.96E-28
GOLGA7B	2.710608	4.654272	5.45E-29	9.97E-28
AL365181.2	5.321436	2.292539	5.80E-29	1.06E-27
AL354707.1	2.927875	0.979115	5.87E-29	1.07E-27
TMEM105	2.198057	1.783343	5.87E-29	1.07E-27
LGSN	4.535457	4.336559	6.77E-29	1.23E-27
PABPC1L	2.123819	5.034678	6.99E-29	1.27E-27
AC092115.3	2.810873	-1.57822	7.48E-29	1.36E-27
ITGA11	2.832846	5.426093	7.58E-29	1.37E-27
RPL23AP32	-2.18924	-2.77567	7.82E-29	1.41E-27
COL3A1	2.501009	11.03048	8.30E-29	1.50E-27
TNFRSF18	2.642338	2.979265	9.12E-29	1.65E-27
AC007128.2	5.997114	-1.78175	9.30E-29	1.68E-27
AL445524.1	2.781304	2.956264	1.03E-28	1.86E-27
TNFSF11	4.053717	1.32124	1.05E-28	1.88E-27
SULF1	2.521877	7.385892	1.07E-28	1.91E-27
LPL	-2.07899	6.2421	1.25E-28	2.25E-27
HIST1H2AI	4.858528	0.359542	1.28E-28	2.30E-27
B3GNT6	5.736179	4.160992	1.29E-28	2.31E-27

For Peer Review

AC098934.1	2.400778	2.571663	1.35E-28	2.42E-27
LINC00634	3.725106	-1.98275	1.35E-28	2.42E-27
RAC3	2.320736	3.808546	1.47E-28	2.62E-27
VIL1	6.947615	4.635888	1.54E-28	2.75E-27
AL121832.3	2.23259	-0.07885	1.54E-28	2.75E-27
NETO1	4.732817	0.484925	1.76E-28	3.12E-27
AC138904.1	3.107945	-0.31118	1.97E-28	3.48E-27
F11	-2.75429	1.083442	2.17E-28	3.84E-27
LINC01460	3.907629	0.290521	2.30E-28	4.05E-27
SEL1L2	-2.44053	-3.83709	2.42E-28	4.26E-27
CNFN	2.653282	1.280857	2.52E-28	4.44E-27
AK4	2.571352	4.684596	2.61E-28	4.58E-27
HIST1H2AM	4.734858	-0.69454	2.82E-28	4.94E-27
CYP4A22-AS1	2.521235	-1.54209	2.88E-28	5.05E-27
IL5RA	-2.13377	1.571061	3.00E-28	5.25E-27
LINC02159	4.15988	0.878773	3.08E-28	5.39E-27
CHRNB4	3.355555	-0.50141	3.09E-28	5.39E-27
AL008628.1	-2.03491	-3.44637	3.36E-28	5.86E-27
BMP8A	2.320872	0.477068	3.42E-28	5.95E-27
GNGT1	6.127733	-0.22125	3.57E-28	6.21E-27
LRRC37A9P	3.707204	-1.99586	3.78E-28	6.56E-27
COCH	3.708362	2.715123	4.68E-28	8.11E-27
CLPSL2	5.317693	-0.79762	5.03E-28	8.67E-27
PROC	3.990564	2.180476	5.20E-28	8.97E-27
MUC3A	4.657754	5.977633	6.74E-28	1.16E-26
SLC29A4	3.109478	4.756026	7.15E-28	1.23E-26
PEBP4	-2.56502	5.163918	7.27E-28	1.25E-26
NKX1-2	7.418489	1.006911	7.53E-28	1.29E-26
IMPDH1P8	2.566091	-1.85933	7.74E-28	1.32E-26
CCDC185	4.45916	-1.2701	8.04E-28	1.37E-26
SFRP5	-2.65642	1.098876	8.39E-28	1.43E-26
SCGB1A1	-3.26192	8.191714	8.57E-28	1.46E-26
AC009005.1	2.099851	1.254957	8.63E-28	1.47E-26
COL22A1	3.562027	2.424738	8.85E-28	1.51E-26
DCST2	2.329426	1.185385	8.91E-28	1.52E-26
AP003469.2	2.723021	-1.29749	1.08E-27	1.83E-26
RXRG	-2.36301	0.990128	1.09E-27	1.85E-26
PSMD10P2	5.045573	-1.1398	1.17E-27	1.98E-26
DLL3	6.785575	1.98023	1.19E-27	2.01E-26
MROH3P	5.039977	-0.35367	1.29E-27	2.18E-26
C6orf222	5.410932	1.038758	1.33E-27	2.25E-26
GGTLC3	-2.34236	-3.07135	1.34E-27	2.26E-26
SSTR1	-2.23135	2.605949	1.38E-27	2.32E-26

For Peer Review

AC013457.1	-3.17102	0.169712	1.38E-27	2.33E-26
DERL3	2.2144	5.278297	1.41E-27	2.37E-26
HBD	-2.29779	-2.5359	1.49E-27	2.50E-26
AL606490.8	5.271408	-2.83095	1.66E-27	2.78E-26
C2orf48	2.992587	-0.57381	1.67E-27	2.79E-26
ANXA8L1	-2.45638	1.548883	1.76E-27	2.94E-26
LRRK2	-2.05812	7.106929	1.91E-27	3.19E-26
ADCY8	-3.33981	-0.15984	1.95E-27	3.26E-26
ACY3	2.890031	1.923747	2.19E-27	3.65E-26
BCL2L10	4.070918	-0.39274	2.45E-27	4.07E-26
EGLN3	3.025582	5.994949	2.57E-27	4.28E-26
HPCA	2.345589	-0.82942	2.60E-27	4.33E-26
AL031599.1	-2.42894	-3.64852	2.64E-27	4.37E-26
INSL3	2.057068	-1.16772	2.89E-27	4.79E-26
MMP12	3.944596	5.056504	2.92E-27	4.84E-26
PRDM12	2.97462	-2.52541	3.05E-27	5.06E-26
PGM5P4	-2.17107	-1.81862	3.28E-27	5.42E-26
IL31RA	4.049086	1.831363	3.68E-27	6.06E-26
DUSP13	6.022552	1.266372	3.69E-27	6.07E-26
EFNA2	5.57891	-1.17014	3.76E-27	6.18E-26
CNTFR	-2.42656	-0.2557	3.98E-27	6.54E-26
TEX11	3.427886	-1.48731	4.09E-27	6.71E-26
NQO1	3.027294	8.212086	4.09E-27	6.71E-26
HELT	-3.14973	-2.99253	4.11E-27	6.73E-26
FGL1	7.20405	5.746078	4.18E-27	6.85E-26
AC022784.1	4.513376	0.344167	4.23E-27	6.92E-26
AL590666.2	3.731628	0.963057	4.34E-27	7.08E-26
C1orf61	5.440259	1.814604	4.61E-27	7.52E-26
SLC2A5	2.415692	3.181903	4.64E-27	7.57E-26
AC027277.2	-2.14272	-1.71429	4.82E-27	7.85E-26
GRIN1	3.081891	1.295655	4.96E-27	8.07E-26
SRRM3	2.279048	2.076293	5.27E-27	8.58E-26
AC010719.1	2.850598	0.120255	5.43E-27	8.82E-26
SYNGR4	4.250962	-1.00804	5.88E-27	9.53E-26
PTBP1P	2.894895	-1.01981	5.89E-27	9.55E-26
SLC17A9	2.484671	4.704613	6.04E-27	9.80E-26
LINC00942	6.526347	3.049426	6.77E-27	1.09E-25
MGAT3-AS1	-2.41805	-3.02274	7.02E-27	1.13E-25
EPHX4	2.618407	1.894634	9.11E-27	1.47E-25
LINC02544	3.511241	-0.75493	9.55E-27	1.53E-25
AL136452.1	-2.16665	-1.13244	9.97E-27	1.60E-25
OGN	-2.2091	3.260769	1.06E-26	1.70E-25
SLC12A9-AS1	2.138953	-1.10596	1.08E-26	1.74E-25

For Peer Review

HAVCR1	5.104653	1.402269	1.16E-26	1.86E-25
KCNMB2-AS1	5.441933	0.992165	1.30E-26	2.07E-25
AC090844.3	-2.1779	-3.5339	1.45E-26	2.30E-25
GTF2IP7	2.911734	-0.04029	1.47E-26	2.34E-25
BEND3P2	-2.36552	-3.57377	1.50E-26	2.38E-25
MB	3.227275	3.223028	1.50E-26	2.38E-25
AC016773.1	2.030992	-0.5812	1.63E-26	2.59E-25
NGEF	3.00341	3.37455	1.67E-26	2.64E-25
AC025470.2	-2.36168	-3.73788	1.68E-26	2.65E-25
AL096828.3	2.022719	-1.0428	1.72E-26	2.71E-25
KRTAP4-1	7.114359	0.206984	1.79E-26	2.82E-25
COL5A2	2.156141	7.647933	1.96E-26	3.09E-25
AL121949.3	5.154348	-0.67692	2.05E-26	3.22E-25
MIR559	2.40784	-1.95702	2.07E-26	3.26E-25
LINC02362	2.583923	1.291519	2.15E-26	3.38E-25
HOXC-AS2	5.055553	-0.69396	2.16E-26	3.40E-25
EPHB2	2.264497	5.276138	2.19E-26	3.44E-25
AL096865.1	2.186678	-1.04652	2.23E-26	3.50E-25
FAM72B	2.273746	0.097631	2.30E-26	3.61E-25
AC010998.1	-2.62742	-1.95532	2.31E-26	3.61E-25
AL079303.1	3.264772	0.341441	2.34E-26	3.67E-25
IL22RA2	3.702154	0.554756	2.39E-26	3.74E-25
AWAT2	-2.57856	-1.71879	2.78E-26	4.33E-25
BARX1	7.490523	3.908973	2.82E-26	4.40E-25
LINC01833	6.588186	0.670967	2.83E-26	4.41E-25
YWHAEP7	3.781966	-0.94244	2.87E-26	4.47E-25
IGF2BP3	3.979612	3.701711	3.05E-26	4.74E-25
AL138760.1	5.22066	-1.64127	3.20E-26	4.97E-25
S100P	5.042722	7.843796	3.25E-26	5.05E-25
LINC01705	3.773513	-1.51147	4.10E-26	6.33E-25
U62317.4	2.834975	-0.02909	4.17E-26	6.43E-25
GUCA1A	4.326958	-1.63788	4.25E-26	6.55E-25
IGHG4	3.81074	8.551313	4.27E-26	6.58E-25
RPL39L	2.321324	3.899822	4.29E-26	6.60E-25
SFTPD	-2.24375	8.180577	4.43E-26	6.82E-25
C15orf48	3.071474	5.920897	4.47E-26	6.87E-25
MESP1	2.3663	2.07673	4.49E-26	6.90E-25
RNF182	-2.04639	0.944317	4.59E-26	7.04E-25
NPSR1	5.987612	-1.0511	5.01E-26	7.66E-25
AC104024.2	3.215893	-0.28199	5.01E-26	7.66E-25
AC013731.1	2.156336	-1.43002	5.05E-26	7.73E-25
CDH3	2.791227	6.878327	5.18E-26	7.91E-25
TESMIN	2.750327	2.356818	5.80E-26	8.82E-25

For Peer Review

OVOL1	2.320376	2.399436	6.09E-26	9.25E-25
RTN4RL2	2.854115	3.773863	6.26E-26	9.50E-25
ZPLD1	4.61344	-0.1074	6.69E-26	1.01E-24
AL353693.1	5.11382	-1.84517	6.69E-26	1.01E-24
MAP7D2	3.349983	3.2898	7.02E-26	1.06E-24
AP002957.1	4.185563	-1.09828	7.33E-26	1.11E-24
ATP6V1C2	2.966911	3.361929	7.52E-26	1.13E-24
DDIT4L	3.278478	3.50168	7.83E-26	1.18E-24
SYNGR3	2.809517	1.987594	8.88E-26	1.33E-24
AQP10	-2.20765	-2.48717	9.05E-26	1.36E-24
DGCR9	3.004041	0.795129	9.50E-26	1.42E-24
GREB1L	3.274567	2.224998	9.89E-26	1.48E-24
LGI2	2.682077	3.740276	1.03E-25	1.55E-24
AC097358.2	3.041764	-2.69455	1.05E-25	1.56E-24
RHOV	3.631942	4.913731	1.05E-25	1.57E-24
SEMA6A-AS2	-2.03147	-4.03311	1.09E-25	1.62E-24
HECW1	2.352641	0.960006	1.10E-25	1.64E-24
CR381670.1	-2.02861	-1.85084	1.11E-25	1.65E-24
PLA2G2C	3.040836	-1.19064	1.15E-25	1.72E-24
LINC01607	2.441165	-0.20187	1.17E-25	1.73E-24
CEACAM5	4.782058	9.433196	1.25E-25	1.85E-24
AP000769.1	2.293411	2.173653	1.29E-25	1.91E-24
LINC02473	4.164392	-2.51427	1.31E-25	1.95E-24
FAM155B	3.182526	1.609496	1.38E-25	2.04E-24
AC099066.2	2.866887	-1.31199	1.49E-25	2.20E-24
IL36RN	6.168963	1.595575	1.50E-25	2.21E-24
MELTF	2.811228	4.661406	1.50E-25	2.21E-24
NLRP3P1	4.52465	-1.87179	1.56E-25	2.30E-24
AP000553.2	2.916776	-1.57757	1.57E-25	2.31E-24
SRPX2	2.296617	4.807418	1.64E-25	2.41E-24
MMP17	2.668642	2.985184	1.69E-25	2.48E-24
BHLHA15	2.87892	1.621341	1.78E-25	2.61E-24
AC007639.1	3.757496	-2.32312	1.83E-25	2.68E-24
TRIM31	4.055099	3.344742	1.85E-25	2.72E-24
MCIDAS	2.909367	1.204592	2.10E-25	3.07E-24
PROK2	-2.05789	-1.25165	2.14E-25	3.13E-24
LINC00629	3.433397	-2.14584	2.19E-25	3.19E-24
PGLYRP4	4.234658	1.094116	2.19E-25	3.19E-24
AP003108.5	3.802441	-0.7582	2.44E-25	3.55E-24
AL163953.1	5.247784	-2.54331	2.47E-25	3.60E-24
ACRV1	2.617873	-1.7226	2.58E-25	3.75E-24
AC084880.3	-2.57125	-3.46572	2.63E-25	3.82E-24
CCBE1	-2.08456	3.467576	2.69E-25	3.90E-24

For Peer Review

U52111.1	2.239008	-0.61581	2.78E-25	4.03E-24
KREMEN2	2.936798	0.523079	3.10E-25	4.48E-24
PAEP	7.637388	6.314549	3.12E-25	4.51E-24
AP000974.1	2.282541	-1.18892	3.47E-25	5.00E-24
SCARA5	-2.33888	3.127747	3.78E-25	5.45E-24
PODNL1	2.789683	3.921201	4.24E-25	6.09E-24
NKPD1	2.509053	-1.1885	4.47E-25	6.41E-24
JSRP1	3.047624	2.435667	4.47E-25	6.42E-24
IGFL2	3.756147	1.355985	4.60E-25	6.60E-24
RASGRF1	-2.10322	4.582018	4.60E-25	6.60E-24
GPX2	5.942204	6.696555	4.64E-25	6.65E-24
AC008496.2	4.447698	-0.45433	4.80E-25	6.86E-24
FLJ34503	-2.31518	-2.70967	4.84E-25	6.92E-24
FAM178B	2.904711	1.742081	5.25E-25	7.50E-24
ZBTB16	-2.20505	3.057116	5.41E-25	7.72E-24
AC005256.1	6.660719	-1.91872	5.51E-25	7.85E-24
PLPPR5	-2.45011	-2.72786	5.98E-25	8.51E-24
LIPM	2.570886	0.422313	6.25E-25	8.87E-24
NKAIN1	4.70435	0.123964	6.39E-25	9.06E-24
PKHD1L1	-2.28505	1.807872	7.01E-25	9.93E-24
NXPH4	4.175596	2.408982	7.29E-25	1.03E-23
ECEL1	5.02433	2.344222	7.43E-25	1.05E-23
DNMT3B	2.042101	2.763554	7.50E-25	1.06E-23
CFAP161	-2.09329	-0.16373	7.91E-25	1.12E-23
RXFP2	-2.79819	-2.27624	7.94E-25	1.12E-23
NPW	5.048017	1.878734	7.98E-25	1.13E-23
METTL7B	2.589107	4.012912	9.78E-25	1.37E-23
RAET1K	2.298819	-1.64283	9.89E-25	1.38E-23
AC079089.1	3.199842	-1.45093	1.00E-24	1.40E-23
XKRX	2.822702	3.905856	1.05E-24	1.47E-23
PRR36	2.152065	4.427186	1.07E-24	1.50E-23
HNRNPA1P33	-2.12953	-3.18997	1.13E-24	1.58E-23
LYPD3	3.445998	4.186842	1.18E-24	1.65E-23
LRRC18	-2.25118	-0.2213	1.49E-24	2.07E-23
AL121949.1	4.15558	2.348708	1.50E-24	2.08E-23
RPL23AP77	3.761076	-1.76488	1.51E-24	2.10E-23
LINC00628	3.580054	-2.75219	1.55E-24	2.15E-23
CBX2	2.480854	4.145087	1.55E-24	2.15E-23
PANX2	2.445745	3.976217	1.63E-24	2.25E-23
C9orf24	-2.41923	2.534862	1.70E-24	2.35E-23
EPHX3	2.230202	4.141457	1.73E-24	2.39E-23
RNFT2	2.195217	2.669552	1.81E-24	2.51E-23
DRP2	3.035267	0.25062	1.91E-24	2.63E-23

For Peer Review

PGLYRP1	-2.04911	-2.66846	1.94E-24	2.68E-23
LINC01395	4.95886	-1.48603	1.96E-24	2.70E-23
INHA	6.344159	3.547294	2.05E-24	2.82E-23
CPB2	-2.74187	3.451619	2.15E-24	2.96E-23
RN7SL475P	-3.05958	-4.0084	2.19E-24	3.01E-23
RCOR2	2.784968	2.324582	2.19E-24	3.01E-23
AC090505.1	4.131949	-2.3268	2.24E-24	3.07E-23
AL354861.3	-2.33615	-3.33364	2.26E-24	3.11E-23
AP002498.1	4.060759	0.075163	2.54E-24	3.47E-23
LINC00887	3.864156	-0.86327	2.60E-24	3.56E-23
HIST1H3H	2.614133	2.139226	2.65E-24	3.62E-23
BNIP3P11	2.089718	0.220936	2.77E-24	3.78E-23
AC005077.4	3.221831	1.994033	2.84E-24	3.88E-23
COL7A1	3.137667	4.738507	3.14E-24	4.27E-23
FOXI3	6.697691	1.519625	3.20E-24	4.34E-23
PLAU	2.591746	7.466242	3.42E-24	4.65E-23
APCDD1L	4.072781	1.206016	3.42E-24	4.65E-23
MESP2	3.116867	0.406725	3.47E-24	4.71E-23
LINC00880	3.279329	-0.75604	3.56E-24	4.82E-23
AL355796.1	4.596757	-0.77001	3.56E-24	4.83E-23
CDKN2A	3.517571	4.57377	3.65E-24	4.95E-23
LINC02003	4.133978	-2.49533	3.97E-24	5.37E-23
AC005324.1	-2.87953	-3.81048	4.03E-24	5.45E-23
AC106900.2	4.433686	-1.75836	4.10E-24	5.54E-23
HMGB3P6	3.35078	-2.7394	4.35E-24	5.88E-23
AC093904.4	3.738708	0.036982	4.44E-24	5.99E-23
AL365199.1	-2.06195	-3.49848	4.50E-24	6.07E-23
AP003064.2	-2.40288	-2.09629	4.70E-24	6.33E-23
AC141557.2	3.519256	-2.14577	4.80E-24	6.46E-23
ERFE	3.275548	2.409865	5.35E-24	7.19E-23
ARNTL2	2.486023	5.144856	5.96E-24	7.99E-23
IGHG1	3.081103	12.41286	6.12E-24	8.20E-23
FAM180B	-2.07611	-3.17087	6.55E-24	8.76E-23
DSP	2.189461	8.495688	6.63E-24	8.86E-23
LINC00525	3.328329	-1.318	7.06E-24	9.42E-23
AC079062.1	6.648695	2.922398	7.47E-24	9.97E-23
IL37	6.331743	3.220951	7.52E-24	1.00E-22
CST4	6.877453	0.255487	7.72E-24	1.03E-22
CELF5	3.327469	0.458767	7.92E-24	1.05E-22
IL23A	2.130962	1.45465	9.29E-24	1.23E-22
CATSPERD	-2.57834	-0.1693	9.98E-24	1.32E-22
PPP2R2C	5.001086	3.490595	1.01E-23	1.34E-22
FUT9	6.520731	1.296275	1.01E-23	1.34E-22

For Peer Review

FA2H	2.529538	4.727536	1.01E-23	1.34E-22
LHX2	4.68019	-0.49948	1.11E-23	1.47E-22
AC107959.3	2.251936	-0.28731	1.15E-23	1.52E-22
AC099509.1	3.721398	0.073727	1.19E-23	1.58E-22
AKR1B15	5.836432	1.943851	1.20E-23	1.59E-22
VGF	5.903888	2.537882	1.22E-23	1.62E-22
PACRG	-2.08005	0.894659	1.28E-23	1.69E-22
STRIP2	2.701824	3.552091	1.29E-23	1.71E-22
PLA2G2F	5.510508	-1.22926	1.38E-23	1.82E-22
RAB3B	4.204002	3.056398	1.47E-23	1.93E-22
DUSP9	4.539927	1.381824	1.47E-23	1.93E-22
HIST1H3J	5.635004	-1.34284	1.52E-23	2.00E-22
PPP1R14BP2	3.178745	-2.32799	1.53E-23	2.01E-22
AC104966.1	3.328879	0.066604	1.56E-23	2.05E-22
TTYH1	2.404366	-0.02304	1.57E-23	2.05E-22
PCP4	5.356598	3.912947	1.58E-23	2.07E-22
GSTA3	-2.54445	-1.10611	1.59E-23	2.08E-22
AP000424.1	3.849144	-2.43719	1.63E-23	2.13E-22
OR7E36P	-2.51685	-2.86815	1.64E-23	2.14E-22
KPNA7	2.236228	1.918585	1.69E-23	2.21E-22
ASPHD1	2.725548	3.432699	1.71E-23	2.24E-22
ANKRD34B	5.024461	1.276162	1.71E-23	2.24E-22
SYPL1P2	2.010566	-1.32034	1.77E-23	2.31E-22
AC120498.4	5.558186	-1.66546	1.83E-23	2.38E-22
RNF186	5.130649	0.011306	1.91E-23	2.48E-22
PADI1	6.354283	2.342396	1.97E-23	2.55E-22
TFF1	9.262854	5.837036	1.99E-23	2.58E-22
AL109615.3	2.940689	2.119378	2.25E-23	2.90E-22
AL355312.3	2.679378	1.448057	2.39E-23	3.08E-22
PPFIA4	2.361475	2.370326	2.46E-23	3.17E-22
C12orf42	2.843438	-0.77155	2.76E-23	3.54E-22
C11orf88	-2.4659	1.692751	2.79E-23	3.58E-22
PRSS50	3.266519	0.855766	2.84E-23	3.65E-22
RTBDN	5.0432	-0.1383	2.96E-23	3.80E-22
AC091057.4	2.404953	-1.75274	3.22E-23	4.12E-22
LINC02474	4.70538	1.04458	3.31E-23	4.22E-22
HES6	2.818453	4.486675	3.31E-23	4.23E-22
CDH17	5.262853	3.664155	3.34E-23	4.26E-22
TLL2	2.947656	0.410961	3.37E-23	4.30E-22
EPYC	5.201731	0.329504	3.49E-23	4.45E-22
RNF183	4.114938	1.747766	3.54E-23	4.50E-22
TMEM156	2.459571	2.727611	3.83E-23	4.87E-22
LINC00461	4.143299	-2.05958	4.02E-23	5.10E-22

For Peer Review

IGHGP	2.803183	6.342021	4.30E-23	5.46E-22
LINC00665	2.275022	4.434592	4.42E-23	5.60E-22
HIST1H2BG	3.989299	1.345169	4.51E-23	5.70E-22
TPSP2	6.8389	-1.20488	4.62E-23	5.85E-22
GJB6	5.112925	2.06182	5.04E-23	6.36E-22
TRIM15	6.19628	0.727385	5.19E-23	6.54E-22
TCN1	6.464353	4.733883	5.52E-23	6.95E-22
MYOSLID	3.327462	0.761353	5.52E-23	6.95E-22
IMPA1P1	-2.44443	-1.75098	5.75E-23	7.23E-22
F11-AS1	-2.28046	-1.3075	6.11E-23	7.67E-22
AC023301.1	4.262302	-0.85966	6.62E-23	8.31E-22
AL355987.5	2.689692	-2.78073	6.63E-23	8.32E-22
IL20RB	3.85623	3.240014	6.66E-23	8.35E-22
SLC7A11	3.067674	5.405342	6.76E-23	8.46E-22
C8B	-2.65674	2.043387	7.03E-23	8.80E-22
MIR30C2	-2.1489	-3.24574	7.41E-23	9.25E-22
LINC02081	2.852607	0.33408	7.68E-23	9.58E-22
AC093904.2	3.408819	-1.71508	7.92E-23	9.87E-22
FAM72D	2.265608	-0.61039	9.14E-23	1.13E-21
GLB1L3	5.181539	4.39814	1.02E-22	1.26E-21
SP8	6.739823	1.146341	1.02E-22	1.27E-21
IGFBP3	2.156872	8.380933	1.05E-22	1.30E-21
PGLYRP3	5.608812	-0.44006	1.09E-22	1.34E-21
MUC13	5.907338	5.9256	1.17E-22	1.44E-21
BCAN	3.041789	1.028432	1.18E-22	1.45E-21
FUT6	3.895763	2.817617	1.23E-22	1.52E-21
COL17A1	4.175565	6.021165	1.24E-22	1.53E-21
FAM72C	2.697576	-1.16253	1.29E-22	1.59E-21
AC236972.3	-2.4528	0.018186	1.32E-22	1.62E-21
AC011352.3	4.686909	-2.88606	1.39E-22	1.71E-21
COL6A4P1	3.388177	-1.64192	1.41E-22	1.73E-21
HMGA2	5.76871	4.120695	1.50E-22	1.84E-21
SUSD2	-2.03842	7.931342	1.59E-22	1.95E-21
AC090673.1	3.324561	-0.2794	1.77E-22	2.16E-21
HIST2H4A	2.22413	-1.12362	1.82E-22	2.22E-21
RPSAP52	5.026409	-0.61024	1.84E-22	2.24E-21
AC078923.1	3.965276	-1.01982	1.90E-22	2.32E-21
CPHL1P	4.084203	0.106729	2.06E-22	2.50E-21
MIOX	4.039704	0.720212	2.15E-22	2.61E-21
FRGCA	3.718908	-1.49962	2.15E-22	2.61E-21
SCG5	2.815866	3.517394	2.30E-22	2.78E-21
B3GNT4	2.113111	1.29676	2.39E-22	2.90E-21
RPL32P33	3.962065	-2.66478	2.68E-22	3.24E-21

For Peer Review

TESC	3.57982	6.285142	2.72E-22	3.29E-21
LINC02265	-2.03126	-1.92687	2.73E-22	3.30E-21
AC111149.2	4.380128	0.390161	2.86E-22	3.44E-21
TOX3	2.795034	4.733298	2.93E-22	3.53E-21
HIST1H1D	6.528051	1.088202	2.99E-22	3.60E-21
CYP27C1	2.992919	1.804794	3.01E-22	3.62E-21
ARTN	2.70847	1.340045	3.01E-22	3.62E-21
CFAP52	-2.10266	1.856375	3.02E-22	3.63E-21
LINC01348	2.629573	1.080998	3.12E-22	3.75E-21
PRB3	4.403917	0.364206	3.27E-22	3.91E-21
AC087269.1	3.809769	-0.83498	3.42E-22	4.08E-21
AC007285.2	2.434346	-2.25604	3.57E-22	4.27E-21
PACSIN1	2.456888	1.345756	3.77E-22	4.50E-21
CPNE4	2.696665	1.906026	3.79E-22	4.52E-21
ZIC2	5.173299	0.838555	3.92E-22	4.67E-21
FCRL5	2.509838	3.789783	3.92E-22	4.67E-21
TNS4	3.925116	5.139711	4.06E-22	4.82E-21
MMP3	3.883445	1.138986	4.12E-22	4.90E-21
LINC00337	2.800145	-0.59002	4.21E-22	5.00E-21
AKR1B10	7.368182	6.328462	4.30E-22	5.10E-21
AC024257.1	-2.12669	-2.94696	4.34E-22	5.14E-21
CRYGN	3.189871	0.112217	4.35E-22	5.16E-21
HOXC13	6.935528	1.055146	4.42E-22	5.23E-21
KCNA10	-2.29305	-3.54158	4.45E-22	5.28E-21
NRK	3.733952	3.182366	4.48E-22	5.30E-21
ZNF887P	2.034859	-2.15556	4.53E-22	5.36E-21
HIST1H1PS1	3.708142	-1.77597	4.59E-22	5.43E-21
MYO3B	3.480007	1.583222	4.61E-22	5.44E-21
SLC7A10	4.722871	1.598549	4.68E-22	5.53E-21
HOXB9	7.14968	3.58789	4.71E-22	5.56E-21
VSX1	3.748078	-1.20307	4.72E-22	5.57E-21
FOXA3	3.262423	3.064606	4.81E-22	5.67E-21
IL36G	4.286353	-0.63184	4.90E-22	5.77E-21
AGR2	2.49019	9.19395	5.30E-22	6.23E-21
PRSS3	5.14646	1.973758	5.51E-22	6.47E-21
UPK3BP1	-2.20334	-3.13805	5.65E-22	6.62E-21
AF127936.2	2.993959	-1.79809	5.91E-22	6.91E-21
LINC00624	2.378022	0.54624	5.99E-22	7.00E-21
HIST1H1E	5.622902	1.833992	6.03E-22	7.04E-21
MNX1-AS2	2.971505	-1.34974	6.19E-22	7.22E-21
MROH9	-2.3877	-0.48091	6.69E-22	7.79E-21
CXCL14	4.304967	7.238446	7.06E-22	8.21E-21
POU4F1	5.31678	0.11755	7.31E-22	8.49E-21

For Peer Review

AL033397.2	4.070015	-0.69298	7.55E-22	8.77E-21
GNG4	4.407982	3.421868	7.64E-22	8.87E-21
SGK2	2.578825	2.027023	7.77E-22	9.00E-21
SLCO1A2	-2.75135	1.490061	7.84E-22	9.08E-21
LINC01993	2.859375	-0.88174	7.91E-22	9.15E-21
UMODL1	4.192018	2.737209	7.96E-22	9.20E-21
HIST1H2BH	4.603273	0.164198	8.06E-22	9.30E-21
STK32A	2.032822	4.45889	8.33E-22	9.61E-21
STK31	2.005493	2.462628	8.46E-22	9.76E-21
HGD	3.792538	4.532816	8.47E-22	9.76E-21
PNMA8C	-2.08461	-3.49027	9.33E-22	1.07E-20
OR2B6	3.455476	-2.09228	9.68E-22	1.11E-20
ALDH3B2	2.839356	3.64691	9.78E-22	1.12E-20
STK32A-AS1	3.091764	0.171108	9.87E-22	1.13E-20
HIST1H3D	3.449964	0.08407	1.00E-21	1.15E-20
LINC01983	3.71541	-0.37392	1.00E-21	1.15E-20
AL133153.1	2.856264	-3.29116	1.00E-21	1.15E-20
FGF19	8.865287	1.29894	1.02E-21	1.16E-20
AC016877.3	3.122983	-1.81304	1.04E-21	1.19E-20
HOXA10	4.187988	1.711038	1.11E-21	1.27E-20
IGKV1-33	4.012568	0.396477	1.15E-21	1.31E-20
CT83	6.597401	1.953296	1.17E-21	1.33E-20
AP000251.1	3.373242	-1.43772	1.20E-21	1.36E-20
SLC25A48	4.16889	-1.59468	1.23E-21	1.40E-20
AC093249.2	2.009784	-2.29287	1.24E-21	1.41E-20
IGKV3-15	2.754711	5.957802	1.26E-21	1.44E-20
AC093895.1	3.549757	-0.58275	1.29E-21	1.47E-20
RAMP1	2.197073	5.075859	1.31E-21	1.48E-20
AK4P1	2.279848	-1.79027	1.31E-21	1.49E-20
EEF1E1P1	2.162493	-2.20428	1.33E-21	1.50E-20
POU3F2	6.758267	2.064365	1.37E-21	1.55E-20
CDH19	-2.22254	-0.05469	1.40E-21	1.58E-20
LINC00160	3.594189	-1.4782	1.42E-21	1.60E-20
HIST1H2AG	3.138272	1.25361	1.46E-21	1.64E-20
TMEM82	4.73793	-1.16085	1.50E-21	1.69E-20
SLC25A5P5	3.854595	-1.09564	1.51E-21	1.70E-20
MUC20	2.315185	5.68363	1.51E-21	1.71E-20
AC078778.1	2.002498	-0.8859	1.56E-21	1.76E-20
SRSF12	2.064764	1.091961	1.57E-21	1.77E-20
MGAT4EP	3.495021	-2.22299	1.60E-21	1.80E-20
ABCA4	3.692938	4.40071	1.61E-21	1.81E-20
TUBB2B	3.922551	3.680384	1.71E-21	1.92E-20
SPTB	3.269754	4.908253	1.72E-21	1.93E-20

For Peer Review

SERPINA4	8.08582	1.718308	1.73E-21	1.94E-20
PLOD2	2.032704	6.897839	1.82E-21	2.04E-20
CPS1	6.636316	7.830533	1.88E-21	2.10E-20
LIPK	6.475078	-1.60411	1.92E-21	2.15E-20
HIST2H2BF	2.710234	1.562352	1.93E-21	2.15E-20
UCN2	3.969896	-0.99247	1.94E-21	2.16E-20
RMDN2-AS1	2.595626	-0.82309	2.00E-21	2.23E-20
VAC14-AS1	2.575083	-0.81855	2.04E-21	2.28E-20
SIX1	2.354489	4.739493	2.20E-21	2.45E-20
GREB1	2.688602	3.381676	2.23E-21	2.48E-20
CLDN10	4.075448	4.309115	2.30E-21	2.55E-20
SHOX2	3.439248	1.244219	2.33E-21	2.59E-20
TRIM31-AS1	2.330939	-0.66648	2.34E-21	2.60E-20
LEMD1	2.775384	1.968033	2.36E-21	2.62E-20
AL158847.1	-2.22248	-3.48439	2.39E-21	2.65E-20
AC113346.1	4.17529	-2.83784	2.54E-21	2.81E-20
GAL	5.328673	1.83582	2.56E-21	2.83E-20
IGF2BP1	6.406443	3.065701	2.65E-21	2.93E-20
SMC1B	3.493377	1.106849	2.91E-21	3.20E-20
DMRTA2	5.006798	1.171261	2.92E-21	3.22E-20
LY6D	5.668921	3.239328	2.98E-21	3.28E-20
COX6B2	3.247859	-0.166	3.05E-21	3.36E-20
NAT8L	3.335765	1.931088	3.09E-21	3.39E-20
CAMK2N2	3.218945	-0.40287	3.33E-21	3.66E-20
AC024560.2	2.025106	0.318063	3.39E-21	3.72E-20
SPERT	4.852266	-0.73312	3.47E-21	3.81E-20
LCN12	2.414552	1.141447	3.49E-21	3.83E-20
COL6A4P2	2.140679	-0.63384	3.50E-21	3.83E-20
HIST1H2BF	4.997922	-0.75279	3.53E-21	3.87E-20
TBC1D26	-2.33197	-3.12745	3.58E-21	3.92E-20
ANXA10	7.511108	3.550325	3.60E-21	3.93E-20
XAGE1A	4.576068	-3.044	3.60E-21	3.94E-20
UCA1	5.570636	2.517498	3.62E-21	3.96E-20
AC079467.1	-2.25739	0.598497	3.81E-21	4.16E-20
AIM2	2.809827	3.330455	3.83E-21	4.18E-20
IL11	2.674346	0.777644	3.93E-21	4.29E-20
AC226101.1	-2.1023	-3.69413	4.01E-21	4.37E-20
KCNK12	3.296442	-0.00518	4.11E-21	4.47E-20
KRT83	5.449233	-0.41226	4.33E-21	4.71E-20
LINC01208	4.132838	-2.81426	4.41E-21	4.78E-20
AC005865.1	2.999414	-1.34853	4.41E-21	4.79E-20
AKR7L	2.951071	0.566689	4.53E-21	4.91E-20
AL139420.1	3.969757	-2.97053	4.53E-21	4.91E-20

For Peer Review

AC061975.6	6.505744	-0.89158	4.55E-21	4.93E-20
AC104260.1	-2.07823	-4.03006	4.69E-21	5.07E-20
METTL11B	4.638121	-2.79682	4.77E-21	5.16E-20
HIST1H2AE	3.832952	0.915532	4.77E-21	5.16E-20
REEP6	2.230948	4.345853	4.98E-21	5.37E-20
IGKC	2.612196	11.74829	5.03E-21	5.42E-20
TAS1R3	2.230621	2.238008	5.07E-21	5.47E-20
TRGJP2	-2.30354	-2.87473	5.24E-21	5.64E-20
C1orf194	-2.21666	1.893637	5.44E-21	5.86E-20
AL138789.1	4.784922	-2.42573	5.51E-21	5.93E-20
AL133153.2	2.407907	-3.1298	5.56E-21	5.97E-20
KRT81	5.912794	4.580335	5.80E-21	6.24E-20
FAM57B	2.997445	-1.26086	5.83E-21	6.27E-20
CASC16	4.228524	-1.99128	6.10E-21	6.54E-20
SNTN	-2.23706	2.749609	6.28E-21	6.74E-20
HIST1H4H	2.424355	1.741111	6.42E-21	6.87E-20
SNORA73B	6.644138	4.911752	6.42E-21	6.88E-20
AL034399.2	4.21966	-2.39425	6.49E-21	6.95E-20
FGB	8.914139	7.862514	7.41E-21	7.90E-20
IGHV1-24	3.377301	5.438213	7.78E-21	8.29E-20
GFAP	3.047118	-0.0843	8.03E-21	8.54E-20
IGHV7-81	2.700246	-0.29294	8.09E-21	8.59E-20
IL17C	4.131223	-0.28875	8.19E-21	8.70E-20
TMEM171	2.878064	0.677541	8.46E-21	8.98E-20
AC012349.1	-2.28651	-3.14295	8.75E-21	9.28E-20
C12orf56	4.771214	1.072595	8.77E-21	9.29E-20
BPIFA2	5.318541	2.362048	8.91E-21	9.44E-20
AC233280.1	2.341547	-2.37162	9.55E-21	1.01E-19
DYNLRB2	-2.01002	0.853434	9.67E-21	1.02E-19
ARL9	2.436375	0.184062	9.68E-21	1.02E-19
PPIAP45	3.216945	-2.25263	1.00E-20	1.06E-19
HIST1H2BO	4.74606	-0.62051	1.06E-20	1.12E-19
IGHG3	2.754161	9.642227	1.07E-20	1.13E-19
ANKRD36BP2	2.702332	2.69754	1.08E-20	1.13E-19
HRASLS	2.780342	0.718135	1.17E-20	1.23E-19
AL731533.2	2.216187	-1.44494	1.18E-20	1.24E-19
TRIM54	3.681385	1.263549	1.29E-20	1.35E-19
FOXH1	4.125101	-0.76603	1.33E-20	1.39E-19
TEKT5	2.337109	-0.71746	1.48E-20	1.55E-19
RBP4	-2.00845	3.246806	1.58E-20	1.65E-19
GPR37	2.743706	2.811452	1.72E-20	1.79E-19
IGHG2	2.597261	9.358112	1.73E-20	1.80E-19
LINC02560	2.933663	0.559846	1.76E-20	1.83E-19

For Peer Review

AC004832.1	-2.18367	-0.07281	1.79E-20	1.86E-19
LINC02576	2.264238	-0.89605	1.79E-20	1.86E-19
KLK6	6.637509	3.098721	1.79E-20	1.86E-19
DEPDC7	2.495619	1.914873	1.87E-20	1.94E-19
TMEM145	2.834235	0.231832	1.90E-20	1.97E-19
CEACAM7	5.33151	3.296191	1.99E-20	2.06E-19
IGHV3-33	2.856045	5.048975	2.03E-20	2.10E-19
HORMAD1	4.413916	2.502275	2.05E-20	2.12E-19
FAM216B	-2.3899	3.186407	2.21E-20	2.28E-19
IGKV4-1	2.813284	8.051966	2.26E-20	2.33E-19
SEPT14P12	3.912881	-3.4814	2.26E-20	2.33E-19
AC010255.1	-2.02885	-2.57999	2.40E-20	2.47E-19
G6PC2	-2.42032	-3.77988	2.46E-20	2.53E-19
AL844908.1	2.845647	0.688283	2.48E-20	2.55E-19
APOBEC1	7.081318	-0.32197	2.58E-20	2.65E-19
AC007750.1	2.097359	-1.11623	2.61E-20	2.68E-19
FAM131C	3.383795	-1.47732	2.80E-20	2.87E-19
AL772337.2	-2.742	-3.17546	2.93E-20	3.00E-19
SPTSSB	4.499759	2.470178	2.94E-20	3.01E-19
ABCC2	4.867635	4.350618	2.98E-20	3.05E-19
FOXO6	2.256736	2.204065	3.04E-20	3.11E-19
LINC01842	3.446148	-0.93268	3.04E-20	3.11E-19
SLC16A9	3.173466	4.678292	3.18E-20	3.25E-19
LINC01765	-2.46999	-1.12637	3.20E-20	3.27E-19
TMEM249	2.806226	-2.03588	3.24E-20	3.30E-19
RFX8	2.108043	-0.35491	3.24E-20	3.31E-19
AL078587.1	2.518171	-2.109	3.45E-20	3.51E-19
IGHV1-46	2.761336	5.506878	3.51E-20	3.57E-19
CRABP1	6.136002	1.99128	3.67E-20	3.73E-19
CNGB1	2.901213	1.334016	3.83E-20	3.89E-19
IGLV3-21	2.80445	7.261548	3.88E-20	3.93E-19
AL645608.2	3.45931	-1.09329	4.08E-20	4.14E-19
MYH16	2.642953	-0.06014	4.17E-20	4.22E-19
TUBA4B	-2.20102	1.491721	4.40E-20	4.45E-19
AC138393.3	2.250271	-1.56749	4.43E-20	4.47E-19
HIST1H3C	6.789815	-0.40863	4.48E-20	4.53E-19
IGHV3-79	2.496999	-1.9435	4.56E-20	4.60E-19
TAF7L	4.286619	-0.36549	4.61E-20	4.65E-19
SNORA26	2.932508	-1.02687	4.73E-20	4.77E-19
PCSK1	6.605949	5.588514	4.93E-20	4.97E-19
SPOCK1	3.094128	3.915077	5.19E-20	5.22E-19
CNGA3	4.788859	2.680052	5.22E-20	5.25E-19
TMEM59L	4.166952	4.538214	5.29E-20	5.31E-19

For Peer Review

BRDT	6.090817	2.661486	5.32E-20	5.34E-19
AC007326.1	4.314141	-2.82952	5.36E-20	5.38E-19
AL031058.1	2.070338	0.612963	5.61E-20	5.62E-19
COLEC10	-2.06872	-0.4571	5.67E-20	5.68E-19
HIST1H3B	6.833341	0.213487	5.74E-20	5.75E-19
FOXB1	4.678979	0.494018	6.23E-20	6.23E-19
CPXM1	2.008049	3.865152	6.43E-20	6.42E-19
ITGB1-DT	3.742557	-0.44758	6.59E-20	6.57E-19
ALOXE3	2.723204	-0.6366	6.65E-20	6.64E-19
KRT16	4.748722	3.915516	6.68E-20	6.65E-19
ANKS4B	6.246958	1.735219	7.21E-20	7.16E-19
GPR87	4.093166	3.351382	7.33E-20	7.28E-19
AC053503.2	2.29822	-2.02689	7.51E-20	7.45E-19
AC120036.4	2.257708	0.11699	7.76E-20	7.69E-19
PAH	6.093824	2.417342	7.80E-20	7.73E-19
ALB	9.030839	6.624274	7.90E-20	7.82E-19
LINC01559	6.357893	1.390474	7.94E-20	7.85E-19
UNC93A	5.724911	-1.25401	8.18E-20	8.08E-19
SMCO2	2.442307	-1.32403	8.21E-20	8.11E-19
GPR35	2.462705	3.205782	8.27E-20	8.16E-19
PDX1	8.158635	0.454756	8.41E-20	8.30E-19
SOHLH2	4.794967	0.568603	8.50E-20	8.38E-19
IGHV3-43	3.042523	3.171286	8.57E-20	8.45E-19
TCTE1	-2.22288	0.538053	8.63E-20	8.50E-19
SLCO1B3	6.876621	1.543317	8.77E-20	8.64E-19
ABCB4	2.124574	1.177074	8.85E-20	8.71E-19
NUS1P2	3.574528	-1.51731	8.85E-20	8.71E-19
SPATA4	-2.0154	-0.83997	8.95E-20	8.80E-19
12-Sep	4.070215	-3.04674	9.43E-20	9.27E-19
HIST1H1B	7.371008	1.393908	9.52E-20	9.35E-19
IGHV4-34	2.716775	5.520953	9.59E-20	9.41E-19
ESPN	2.361294	3.877574	9.62E-20	9.44E-19
KRTAP5-1	2.144823	-0.83305	9.70E-20	9.51E-19
IGLL5	2.420979	6.967395	9.90E-20	9.70E-19
BECN2	3.948097	-2.88725	1.00E-19	9.81E-19
IGKV3-11	2.475802	7.820321	1.06E-19	1.04E-18
AC046195.1	-2.39912	0.484747	1.07E-19	1.05E-18
LINC01303	2.425304	-0.86828	1.09E-19	1.07E-18
AC004221.1	2.877496	-3.14082	1.10E-19	1.07E-18
XKR9	2.149975	2.218005	1.11E-19	1.08E-18
AL353152.1	-2.14238	-3.26613	1.11E-19	1.08E-18
LINGO1	2.491131	2.418615	1.11E-19	1.08E-18
AC134312.5	2.506351	-1.44671	1.12E-19	1.09E-18

For Peer Review

SLC16A8	2.189272	0.583933	1.22E-19	1.18E-18
CDIPTOSP	3.253189	-0.57731	1.23E-19	1.20E-18
SLC16A14	2.905453	5.139748	1.23E-19	1.20E-18
AC015712.6	2.979856	0.654894	1.26E-19	1.22E-18
LINC00866	2.765897	-2.53229	1.27E-19	1.23E-18
SLC15A1	4.011204	1.983533	1.30E-19	1.26E-18
HOXC11	6.792026	1.400416	1.30E-19	1.26E-18
AL049539.1	3.339082	-2.54159	1.31E-19	1.27E-18
EDN3	-2.72054	-1.32245	1.33E-19	1.29E-18
RGS20	2.875831	0.983932	1.35E-19	1.31E-18
S100A2	3.386905	5.511891	1.42E-19	1.37E-18
AC239803.3	2.561002	-2.51541	1.47E-19	1.42E-18
CKMT1A	2.85049	1.314272	1.48E-19	1.43E-18
PLA2G1B	-2.19263	2.567051	1.50E-19	1.44E-18
PKD1L2	3.25597	2.026211	1.52E-19	1.47E-18
AC007490.1	2.713466	0.211475	1.54E-19	1.48E-18
KCNQ5	2.607972	2.277882	1.55E-19	1.50E-18
HOTAIR	6.792181	-0.03796	1.57E-19	1.51E-18
NKAIN3	5.253166	-1.43007	1.58E-19	1.52E-18
AC105118.1	4.447247	-1.01645	1.59E-19	1.53E-18
AC233280.2	2.418016	-1.07665	1.60E-19	1.54E-18
HIST1H2AD	4.272378	-0.00052	1.61E-19	1.55E-18
GLRA3	3.843202	0.882888	1.67E-19	1.61E-18
IGKV1-5	2.640797	7.610898	1.70E-19	1.63E-18
SYT2	2.819796	2.072688	1.71E-19	1.64E-18
SP3P	6.489749	0.375848	1.88E-19	1.79E-18
IGKJ5	2.828149	-0.74107	1.94E-19	1.85E-18
NPC1L1	4.399363	2.24184	1.99E-19	1.89E-18
CKMT1B	2.910962	1.621644	2.01E-19	1.91E-18
IGHV3-23	2.516762	7.000258	2.02E-19	1.92E-18
DUXAP8	2.538598	0.865623	2.02E-19	1.92E-18
RNF144A-AS1	2.146952	0.70465	2.06E-19	1.96E-18
GSDMC	2.54188	2.932206	2.07E-19	1.97E-18
SPINK4	8.916344	3.902751	2.13E-19	2.02E-18
AC079160.1	6.03304	-1.17787	2.19E-19	2.08E-18
AC130686.1	2.433824	-2.53013	2.21E-19	2.10E-18
AC016044.1	6.117549	-1.88388	2.21E-19	2.10E-18
AL352984.1	3.482212	-2.98196	2.23E-19	2.11E-18
NKX3-2	3.511901	-1.16568	2.30E-19	2.18E-18
AC013264.1	-2.02839	0.165814	2.44E-19	2.31E-18
HIST1H2BC	2.199582	2.884548	2.48E-19	2.34E-18
LUCAT1	3.123534	2.243496	2.53E-19	2.39E-18
TRIM58	-2.14381	-0.33354	2.68E-19	2.53E-18

For Peer Review

SOX11	4.813317	2.270835	2.71E-19	2.55E-18
ADAMTS16	2.266222	2.604082	2.78E-19	2.62E-18
PCAT7	2.323525	-0.7499	2.83E-19	2.66E-18
AC009121.1	2.183009	-2.25741	3.26E-19	3.06E-18
FAM177B	3.270121	2.241413	3.33E-19	3.12E-18
CASC9	5.44584	1.794463	3.58E-19	3.35E-18
TPTE2	2.413191	-1.9714	3.59E-19	3.35E-18
PACRG-AS1	-2.01676	-3.54874	3.69E-19	3.44E-18
LINC00460	4.264016	1.431422	3.81E-19	3.55E-18
REG4	10.58943	5.890834	3.98E-19	3.70E-18
SMKR1	2.760119	1.969861	4.02E-19	3.74E-18
DIRAS1	2.584659	2.631399	4.07E-19	3.79E-18
TRPM1	-2.12875	-3.12591	4.10E-19	3.81E-18
SLC27A6	-2.45128	-0.59344	4.12E-19	3.83E-18
EPS8L3	7.185979	2.843311	4.27E-19	3.97E-18
CALML3-AS1	3.259365	-0.3113	4.29E-19	3.98E-18
IGLV1-40	2.515111	6.936729	4.42E-19	4.10E-18
COMP	2.857921	5.401127	4.45E-19	4.13E-18
PLA2G4A	2.426244	5.79418	4.56E-19	4.23E-18
UCHL1	3.455534	6.06814	4.56E-19	4.23E-18
PAX9	2.690075	3.806737	4.76E-19	4.40E-18
AP005435.1	4.14344	-1.47363	4.81E-19	4.44E-18
LINC02323	2.894172	-0.59261	5.31E-19	4.89E-18
DIO2	2.361209	3.781611	5.32E-19	4.90E-18
SMIM24	4.213994	1.399197	5.49E-19	5.05E-18
APOBEC3B	2.204659	3.035242	5.69E-19	5.23E-18
LINC01169	-2.12954	-2.2602	5.70E-19	5.24E-18
STEAP2-AS1	3.216718	-2.78196	5.94E-19	5.45E-18
HIST1H2APS3	4.291092	-3.01825	5.95E-19	5.46E-18
OR51E1	2.978303	-0.304	6.40E-19	5.86E-18
LINC02086	3.897563	0.103539	6.42E-19	5.88E-18
ABCC11	2.376478	-0.74944	6.43E-19	5.89E-18
AC089983.1	4.189046	-0.82913	6.70E-19	6.13E-18
TSHR	2.848378	0.091741	6.73E-19	6.16E-18
ANKRD66	-2.35431	0.658749	6.83E-19	6.24E-18
NR2E1	4.438275	-1.49256	6.98E-19	6.37E-18
C9orf84	2.953429	1.780491	7.18E-19	6.55E-18
LHFPL3-AS2	-2.11501	3.096003	7.27E-19	6.63E-18
ALG1L5P	2.782285	-1.49231	7.29E-19	6.65E-18
QPCT	2.625425	5.491156	7.42E-19	6.76E-18
NKAIN4	3.219976	0.596331	7.46E-19	6.79E-18
BARX1-DT	6.471328	-0.6986	7.57E-19	6.89E-18
AL589935.2	-2.07716	-3.80116	7.83E-19	7.12E-18

For Peer Review

ADH1A	-2.02847	-2.11949	7.92E-19	7.20E-18
SRMS	2.446564	0.874769	8.30E-19	7.53E-18
TGFBR3L	2.483643	-0.60904	8.50E-19	7.71E-18
DOK5	2.237112	1.755003	8.69E-19	7.88E-18
PTGES2-AS1	2.411939	-2.00372	9.02E-19	8.18E-18
AC112721.2	3.143019	-2.1795	9.21E-19	8.34E-18
AC254629.1	4.001898	-1.63856	9.56E-19	8.64E-18
MYO16-AS1	-2.45327	-0.50458	9.61E-19	8.68E-18
C20orf197	3.06969	1.383825	9.90E-19	8.94E-18
MUC21	3.753418	6.471478	1.02E-18	9.19E-18
AC105914.2	-2.77456	-3.03867	1.04E-18	9.35E-18
HOXC13-AS	5.923587	-1.17893	1.07E-18	9.66E-18
CLDN6	5.707494	4.301389	1.08E-18	9.68E-18
HIST1H4K	2.389433	-1.78632	1.08E-18	9.74E-18
AC006064.3	2.062962	-2.51726	1.12E-18	1.00E-17
HIST1H2BE	4.14456	-0.70148	1.13E-18	1.01E-17
AC245041.1	-2.20824	0.044203	1.15E-18	1.03E-17
AC004870.2	4.429649	-1.02158	1.18E-18	1.05E-17
AC002076.1	4.008379	-2.03493	1.19E-18	1.07E-17
AL118505.1	2.003232	-0.58836	1.22E-18	1.10E-17
HIST1H4E	5.176122	0.645647	1.25E-18	1.12E-17
LINC01168	-2.37498	-3.55275	1.30E-18	1.17E-17
TDRD5	3.636941	1.779425	1.31E-18	1.17E-17
PF4	-2.00777	-1.02331	1.34E-18	1.20E-17
ABCC13	-2.05784	-0.95119	1.37E-18	1.22E-17
AC092868.1	3.912018	0.74072	1.37E-18	1.22E-17
EGF	2.233814	3.271777	1.38E-18	1.23E-17
LINC02495	2.979175	-1.92842	1.39E-18	1.24E-17
AC026785.3	7.132832	-0.91197	1.42E-18	1.27E-17
CDC37P1	2.909304	-2.24858	1.50E-18	1.33E-17
AC025031.4	2.524115	-0.66339	1.51E-18	1.34E-17
RN7SL3	5.505091	3.607753	1.51E-18	1.34E-17
AC108868.1	-2.05835	-3.68963	1.58E-18	1.40E-17
AC006329.1	2.134567	0.947021	1.59E-18	1.41E-17
IGHV5-51	2.663293	6.734289	1.59E-18	1.41E-17
AL589765.6	2.226756	-2.19483	1.59E-18	1.41E-17
AC116025.2	2.955458	-3.01034	1.62E-18	1.43E-17
IGKV3D-15	3.004483	2.137586	1.65E-18	1.46E-17
AC243830.1	3.07528	-2.84507	1.81E-18	1.59E-17
HOXC10	6.016367	2.694105	1.83E-18	1.61E-17
RPLP0P2	2.068806	2.506336	1.91E-18	1.68E-17
KIF5A	2.688814	0.847932	1.92E-18	1.69E-17
CLPSL1	4.835591	-1.44448	1.92E-18	1.69E-17

For Peer Review

AC084262.1	3.75197	-2.43483	1.95E-18	1.71E-17
PDIA2	4.758067	2.324188	1.97E-18	1.74E-17
CRLF1	4.023136	7.009267	1.99E-18	1.75E-17
PKP4-AS1	2.417389	0.47472	2.02E-18	1.77E-17
TCAM1P	4.477274	0.459338	2.06E-18	1.80E-17
TRIM67	2.386989	-1.33339	2.07E-18	1.82E-17
DUSP4	2.422636	6.692031	2.10E-18	1.84E-17
HIST1H2AJ	6.459404	-1.12784	2.11E-18	1.85E-17
LHFPL3	-2.117	0.415771	2.33E-18	2.04E-17
TRPM2-AS	3.46489	0.283654	2.38E-18	2.09E-17
C1orf87	-2.42922	1.294847	2.45E-18	2.14E-17
CFAP77	-2.18731	1.075687	2.46E-18	2.15E-17
BCO1	2.455509	1.398823	2.46E-18	2.15E-17
LINC02122	4.627326	-1.53607	2.54E-18	2.22E-17
FNDC1	2.390753	5.328552	2.55E-18	2.22E-17
SATB2-AS1	2.802368	-2.38505	2.81E-18	2.44E-17
CYP4F23P	4.678047	-1.64095	2.83E-18	2.47E-17
KCNE1B	-2.09413	-1.71033	2.84E-18	2.47E-17
C2CD6	2.20617	1.451387	2.85E-18	2.48E-17
AC024592.2	2.402738	-1.35331	2.87E-18	2.50E-17
RNU6-438P	4.329352	-3.59288	3.00E-18	2.60E-17
EN2	4.119514	-0.405	3.04E-18	2.63E-17
GRIN2D	2.508513	2.82661	3.17E-18	2.74E-17
AL031777.1	2.807528	-1.42968	3.24E-18	2.80E-17
AP000577.1	2.937632	-2.31354	3.34E-18	2.88E-17
NANOGP1	3.453896	-3.17588	3.68E-18	3.17E-17
CCDC198	5.336242	0.730333	3.77E-18	3.24E-17
WDR86	2.223041	3.048521	3.93E-18	3.37E-17
GJB3	3.269951	3.618682	3.99E-18	3.43E-17
LINC02163	5.939856	-1.79762	4.01E-18	3.44E-17
IGHV3-15	2.529891	5.938916	4.30E-18	3.69E-17
UPK3A	3.080856	0.602656	4.43E-18	3.79E-17
KIF1A	5.459176	5.103339	4.47E-18	3.82E-17
UGT2B15	6.034138	2.135085	4.54E-18	3.88E-17
AL121772.1	2.006576	0.170328	4.59E-18	3.93E-17
CDHR2	4.110374	2.233626	4.82E-18	4.12E-17
THPO	3.046029	1.228129	4.90E-18	4.18E-17
LINC02313	4.396458	-1.7634	4.98E-18	4.25E-17
TRIM51BP	5.07551	-1.94921	5.09E-18	4.34E-17
SNORD17	5.501917	2.926216	5.13E-18	4.37E-17
C11orf97	-2.51575	-0.66861	5.22E-18	4.44E-17
AC104248.1	2.745853	-1.936	5.23E-18	4.44E-17
AP005230.1	3.809052	-1.19828	5.27E-18	4.48E-17

For Peer Review

NCCRP1	3.748021	2.963967	5.44E-18	4.62E-17
LINC00858	5.071205	0.264704	5.69E-18	4.82E-17
FAM92B	-2.00196	2.213943	5.87E-18	4.97E-17
HCN2	2.317703	0.711427	5.87E-18	4.97E-17
LINC02029	3.970752	-2.43253	6.05E-18	5.12E-17
BPIFB4	6.589031	0.856301	6.08E-18	5.14E-17
LINC02133	4.270239	-1.2304	6.09E-18	5.15E-17
OACYLP	2.279224	-1.9026	6.55E-18	5.51E-17
KCND2	2.208386	1.570023	6.55E-18	5.52E-17
LINC01597	4.126356	1.245417	6.64E-18	5.59E-17
AC103740.2	3.71799	-2.68084	7.01E-18	5.90E-17
AC123023.1	-2.2497	-2.95014	7.33E-18	6.16E-17
AL590644.1	4.969631	-1.46012	7.39E-18	6.20E-17
AC099518.1	2.197084	-0.83517	7.43E-18	6.23E-17
AHNAK2	2.182226	6.725552	8.45E-18	7.06E-17
TBXT	5.853028	0.214848	8.58E-18	7.16E-17
AC105219.1	2.904995	-2.34873	8.59E-18	7.16E-17
AL772284.1	4.160611	-3.16233	8.84E-18	7.37E-17
AC010789.1	5.426407	-1.87778	8.94E-18	7.44E-17
IGKV1OR2-108	2.698441	2.229632	9.24E-18	7.69E-17
MYCN	3.978743	2.541737	9.46E-18	7.85E-17
STRC	2.736566	-2.13493	9.69E-18	8.03E-17
SERPINA9	-2.14584	-1.71835	9.75E-18	8.08E-17
AL645608.6	3.179776	-2.7082	9.85E-18	8.16E-17
FAM133A	5.497624	2.184298	9.93E-18	8.23E-17
FADS6	5.103438	0.160238	1.03E-17	8.55E-17
EFCAB1	-2.09164	2.563318	1.03E-17	8.55E-17
SNORA14B	3.481899	-1.87497	1.04E-17	8.62E-17
TEX48	3.587441	-2.41029	1.05E-17	8.67E-17
HIST1H3G	3.657269	-0.05933	1.10E-17	9.08E-17
AL590666.3	2.853825	-3.19789	1.13E-17	9.29E-17
TMEM132C	-2.17827	-0.37386	1.14E-17	9.39E-17
HOXB13	6.362743	1.412912	1.15E-17	9.52E-17
SIX2	3.291317	2.657503	1.17E-17	9.62E-17
AC010328.1	3.476397	-1.9918	1.17E-17	9.67E-17
AC009262.1	4.346042	-3.44097	1.19E-17	9.77E-17
TM4SF4	5.771006	5.346252	1.19E-17	9.82E-17
SYT14	4.899454	0.506935	1.20E-17	9.90E-17
PNCK	3.184853	0.772956	1.29E-17	1.06E-16
TBX10	5.217101	-1.14444	1.29E-17	1.06E-16
LINC00501	5.270425	-0.90794	1.34E-17	1.10E-16
HIST1H2AL	5.350497	-1.44851	1.47E-17	1.20E-16
TFAP2D	6.974925	0.495259	1.47E-17	1.20E-16

For Peer Review

SMOC1	3.821608	4.498463	1.48E-17	1.21E-16
AP001626.1	2.696666	-1.62685	1.50E-17	1.23E-16
DQX1	2.992599	0.370939	1.53E-17	1.25E-16
AC068305.2	3.041303	-1.24652	1.58E-17	1.28E-16
AC103702.2	4.338797	0.350451	1.62E-17	1.32E-16
RF00100	6.993145	6.463553	1.67E-17	1.36E-16
IGHV1OR15-2	3.031235	1.294248	1.69E-17	1.37E-16
MMP10	3.899918	2.991146	1.70E-17	1.38E-16
AC067930.5	2.272316	-1.63464	1.76E-17	1.43E-16
AC011298.1	7.064146	0.192353	1.77E-17	1.44E-16
GCLC	2.253462	6.78082	1.88E-17	1.52E-16
DEFA4	-2.57255	-3.70703	1.88E-17	1.53E-16
PTPRN	4.356179	2.364448	1.90E-17	1.54E-16
PRSS2	6.152979	5.087527	1.94E-17	1.57E-16
LINC00973	6.067327	0.702669	1.95E-17	1.58E-16
LINC02156	3.259396	-2.8999	2.06E-17	1.66E-16
DUSP5P1	3.183264	-1.45985	2.07E-17	1.67E-16
IGKV3D-11	2.438081	1.820803	2.07E-17	1.68E-16
MIR4713HG	4.099156	-2.40941	2.09E-17	1.69E-16
FOXD3	3.948866	-1.55808	2.11E-17	1.70E-16
TINAG	6.742137	0.318729	2.11E-17	1.70E-16
AC068724.1	2.318225	-3.18873	2.12E-17	1.71E-16
AL138902.1	2.430688	-2.88938	2.14E-17	1.73E-16
IGLV2-14	2.406862	7.028167	2.20E-17	1.77E-16
DDN	2.331184	-0.55235	2.25E-17	1.81E-16
FAM83C-AS1	2.273932	-3.1841	2.26E-17	1.82E-16
DRAIC	3.746925	3.253574	2.34E-17	1.88E-16
B4GALNT1	2.484164	2.478407	2.42E-17	1.94E-16
SCUBE3	2.337282	4.141487	2.44E-17	1.95E-16
FSD1	2.548105	-0.22442	2.51E-17	2.01E-16
U47924.1	2.235499	-2.85693	2.63E-17	2.11E-16
IGFBPL1	2.343851	1.264288	2.69E-17	2.15E-16
SCARNA21	5.516921	0.646161	2.72E-17	2.17E-16
AC013652.1	2.374714	0.087024	2.84E-17	2.27E-16
PTHLH	2.72778	2.736372	2.86E-17	2.28E-16
IGHV1-69D	2.70195	5.3156	2.92E-17	2.33E-16
LINC01926	3.474464	-3.19399	3.01E-17	2.40E-16
IGHV1-17	2.962738	-1.55144	3.07E-17	2.45E-16
MAG	-2.08628	-1.60798	3.10E-17	2.47E-16
AC117386.2	4.753336	-2.01455	3.13E-17	2.49E-16
LINC02471	-2.2742	0.179534	3.18E-17	2.53E-16
NXPH1	5.05741	-1.43191	3.28E-17	2.61E-16
CHGB	5.841191	5.386654	3.41E-17	2.71E-16

For Peer Review

TMPOP2	2.150281	-2.51638	3.44E-17	2.73E-16
CASC8	3.161878	-0.03743	3.52E-17	2.79E-16
OR2W3	-2.25016	-2.64673	3.56E-17	2.82E-16
AP003390.1	2.172468	-1.7729	3.65E-17	2.89E-16
GCKR	3.011536	0.105538	3.68E-17	2.91E-16
IGHV4-55	2.273402	1.681213	3.69E-17	2.92E-16
IGHV1-58	2.995136	2.753826	3.74E-17	2.96E-16
AC116049.2	5.208133	-2.73752	3.77E-17	2.98E-16
PIWIL1	5.084861	-1.06814	3.85E-17	3.04E-16
FIRRE	2.562306	0.005921	3.87E-17	3.06E-16
AC128709.2	-2.33417	-3.30754	3.92E-17	3.10E-16
RUFY4	2.229877	0.673009	3.97E-17	3.13E-16
IGHV3-53	2.47629	3.835047	4.03E-17	3.18E-16
AC089999.2	2.285942	-2.10419	4.12E-17	3.25E-16
DLX6-AS1	4.61833	0.578215	4.14E-17	3.26E-16
AC018892.3	2.524112	-3.07847	4.22E-17	3.32E-16
IGLV3-12	2.979356	-0.05161	4.24E-17	3.34E-16
IGKV1-9	2.493896	5.326866	4.29E-17	3.38E-16
OGDHL	3.436396	1.743703	4.37E-17	3.44E-16
LINC01979	2.380019	-1.57667	4.42E-17	3.48E-16
AC012501.2	5.123648	-1.69986	4.47E-17	3.51E-16
F2	7.1866	0.939066	4.61E-17	3.62E-16
LINC00707	4.442137	0.557356	4.66E-17	3.66E-16
AL356274.2	5.217889	-1.84948	4.74E-17	3.72E-16
LINC01748	4.840855	-0.21872	4.86E-17	3.81E-16
CCNO	2.119717	3.299391	4.88E-17	3.83E-16
NOX5	2.98136	0.86912	4.90E-17	3.84E-16
AC008875.1	2.013782	-1.16141	4.92E-17	3.85E-16
MEIOC	2.653865	0.715839	5.00E-17	3.91E-16
IGHV1OR21-1	2.952107	-0.42318	5.03E-17	3.93E-16
LINC01166	-2.36945	-3.3169	5.22E-17	4.08E-16
LRRC71	-2.09223	0.860463	5.29E-17	4.13E-16
DLX6	4.874952	-0.04656	5.30E-17	4.14E-16
MAFA-AS1	4.183682	-1.58654	5.32E-17	4.15E-16
MORN5	-2.21522	0.88461	5.38E-17	4.19E-16
TRIM72	4.286015	1.166063	5.44E-17	4.24E-16
HIST1H4C	5.875631	0.603344	5.51E-17	4.29E-16
IGLV4-69	2.685869	5.220861	5.55E-17	4.32E-16
CSTL1	4.593336	-2.27124	5.60E-17	4.36E-16
SPINK2	2.738796	0.19898	5.61E-17	4.36E-16
TMEM88B	2.833423	-2.03839	5.91E-17	4.60E-16
LCT	3.943932	0.425934	6.05E-17	4.70E-16
HOXB7	2.070991	3.408193	6.14E-17	4.77E-16

For Peer Review

HOXC9	3.227896	0.676326	6.15E-17	4.77E-16
MYBPC1	5.901494	2.702356	6.20E-17	4.81E-16
ZNF670-ZNF695	2.026526	-2.06853	6.25E-17	4.85E-16
IGHV3-30	2.334484	5.679598	6.36E-17	4.94E-16
HMGB2P1	2.226895	-2.24945	6.38E-17	4.95E-16
OTP	-2.38003	-3.12108	6.45E-17	5.00E-16
SLC39A5	3.944075	0.817067	6.54E-17	5.07E-16
AL139420.2	4.066703	-3.73034	6.56E-17	5.08E-16
HIST1H2BM	6.207155	-1.80833	6.69E-17	5.18E-16
ANKRD34C	-2.29368	-3.64171	6.70E-17	5.19E-16
ANGPTL3	3.831535	-1.28874	6.71E-17	5.19E-16
TEKT1	-2.26954	2.745711	6.72E-17	5.20E-16
TUBB8	2.061412	-1.21712	6.82E-17	5.27E-16
IGHV3OR16-11	3.300602	-1.6756	7.06E-17	5.45E-16
HGFAC	2.899222	-0.57324	7.15E-17	5.52E-16
SH3GL2	-2.25034	0.19778	7.15E-17	5.52E-16
ARL14	5.059601	1.852829	7.16E-17	5.53E-16
AC020891.2	4.403941	-2.55309	7.29E-17	5.62E-16
KRT15	2.630972	5.822362	7.31E-17	5.63E-16
IGHV2-26	2.826514	3.751217	7.39E-17	5.69E-16
CALCA	8.509311	7.76904	7.87E-17	6.05E-16
AC093390.1	-2.04305	-3.60202	7.99E-17	6.14E-16
GLDC	2.879699	2.554983	8.06E-17	6.19E-16
AL355076.2	2.03667	-2.15741	8.23E-17	6.32E-16
AC108058.1	2.381834	-0.27053	8.25E-17	6.34E-16
CACNA1E	2.621681	-0.20054	8.43E-17	6.47E-16
MYADML2	2.433188	-2.59954	8.46E-17	6.49E-16
LHX5	5.115282	-1.03135	8.57E-17	6.57E-16
SCARNA6	5.243361	1.6613	8.57E-17	6.57E-16
AC026368.1	2.501025	-0.73805	8.64E-17	6.62E-16
NUP210L	2.375509	1.104971	8.74E-17	6.70E-16
MZB1	2.086199	5.38242	8.78E-17	6.72E-16
CKM	3.011438	0.591946	8.81E-17	6.74E-16
CNGA1	2.315036	1.002548	8.87E-17	6.78E-16
THEGL	2.851545	-2.48599	8.87E-17	6.78E-16
IGLC3	2.273729	7.908084	9.01E-17	6.89E-16
C6	-2.04466	2.365394	9.11E-17	6.96E-16
AC110285.6	2.37565	-1.93461	9.12E-17	6.96E-16
FBXO36P1	3.805015	-2.42952	9.13E-17	6.97E-16
MCHR1	2.013454	-0.18234	9.28E-17	7.08E-16
LINC00165	-2.52962	-2.72865	9.28E-17	7.08E-16
AL133373.2	2.869017	-0.94014	9.32E-17	7.11E-16

For Peer Review

NAALADL2-AS2	5.250061	-1.01876	9.55E-17	7.27E-16
MUC6	6.353261	6.143666	9.94E-17	7.55E-16
IGKV3OR2-268	2.582787	0.757725	1.00E-16	7.61E-16
CECR7	2.504466	1.317011	1.19E-16	9.00E-16
AC064850.1	2.217111	-2.95494	1.22E-16	9.24E-16
TNNT1	3.214486	4.431377	1.23E-16	9.31E-16
CATSPERB	2.213348	2.205865	1.25E-16	9.41E-16
TFF2	11.59965	5.59832	1.25E-16	9.45E-16
KRT6A	5.470376	6.573662	1.26E-16	9.46E-16
IGHJ2	2.600703	0.341195	1.26E-16	9.48E-16
IGKV1D-33	4.095936	-0.19679	1.28E-16	9.66E-16
AC083809.1	4.950474	1.248534	1.28E-16	9.66E-16
IGHVII-78-1	3.049235	-3.09308	1.29E-16	9.69E-16
IGHJ3P	2.635394	-0.43332	1.31E-16	9.84E-16
TM4SF5	8.225192	1.73266	1.32E-16	9.97E-16
IGKV3-7	2.426328	1.828666	1.34E-16	1.01E-15
AC061975.1	3.154985	-3.09758	1.35E-16	1.02E-15
ACSM2A	-2.08778	-3.81058	1.39E-16	1.04E-15
ENO3	2.518032	3.750208	1.40E-16	1.05E-15
IGHV1OR15-9	2.712836	0.735591	1.41E-16	1.06E-15
LINC00466	4.267319	-3.19766	1.41E-16	1.06E-15
LHB	2.079706	-1.9843	1.42E-16	1.06E-15
ANKRD36C	2.796938	3.184876	1.44E-16	1.08E-15
TMEM75	4.171563	-1.27945	1.44E-16	1.08E-15
OR7E11P	-2.03966	-3.51368	1.49E-16	1.12E-15
RFLNA	2.546241	2.681576	1.50E-16	1.12E-15
IGHV1-18	2.500853	6.519534	1.50E-16	1.12E-15
ADGRG4	-2.39261	-3.42336	1.53E-16	1.14E-15
AC006946.2	2.62578	-0.57296	1.53E-16	1.14E-15
SPRR1B	6.053359	3.058895	1.54E-16	1.15E-15
PRSS1	6.089845	2.102829	1.55E-16	1.15E-15
SALL1	5.729334	1.010724	1.57E-16	1.17E-15
SERPINB5	4.394224	3.975002	1.58E-16	1.18E-15
IGHV3-11	2.457136	5.561004	1.59E-16	1.18E-15
LINC01970	2.015338	-1.93145	1.63E-16	1.21E-15
AL353807.2	2.239547	-3.32531	1.66E-16	1.23E-15
BNIP3P10	2.451144	-2.63281	1.68E-16	1.25E-15
AC004034.1	2.435679	-1.39877	1.71E-16	1.27E-15
SNORA71A	3.975188	-0.12263	1.73E-16	1.29E-15
AL355596.1	5.097866	-0.26761	1.77E-16	1.31E-15
AL161618.1	-2.29883	-1.73427	1.77E-16	1.32E-15
LINC01561	3.139114	-2.21103	1.78E-16	1.32E-15

For Peer Review

AC034105.1	2.928317	-0.72473	1.79E-16	1.33E-15
IGLV6-57	2.614864	6.034926	1.80E-16	1.33E-15
ALOX15	-2.14932	3.407928	1.80E-16	1.34E-15
LINC02321	2.553495	-1.85277	1.84E-16	1.36E-15
ITPRID1	4.507869	1.987618	1.85E-16	1.37E-15
C1orf105	3.133636	-2.62044	1.86E-16	1.37E-15
TFPI2	3.842252	7.485827	1.86E-16	1.37E-15
SNORD14E	2.110433	-1.7829	1.86E-16	1.38E-15
LCN2	2.673229	7.581424	1.88E-16	1.39E-15
GABRA3	5.952117	1.325341	1.92E-16	1.41E-15
MTNR1A	3.637035	-2.1127	1.92E-16	1.41E-15
IGLVI-70	3.727389	-0.04504	1.95E-16	1.44E-15
C4orf54	3.737045	0.187783	1.95E-16	1.44E-15
EYS	2.167356	1.949476	1.96E-16	1.44E-15
CASP1P2	2.078391	-1.90714	1.97E-16	1.45E-15
ACMSD	3.169173	0.828342	2.01E-16	1.48E-15
SYT16	2.974884	0.895665	2.03E-16	1.50E-15
MIR6835	2.267594	-3.21714	2.07E-16	1.52E-15
GDNF	2.905013	-0.12684	2.08E-16	1.53E-15
LHX1	6.712567	-0.2589	2.09E-16	1.53E-15
AC104699.1	2.198501	-0.89051	2.10E-16	1.54E-15
CLCA3P	3.352252	-2.2729	2.11E-16	1.55E-15
WFDC10B	2.347617	0.331065	2.14E-16	1.57E-15
FAM222A-AS1	2.678496	-1.34445	2.16E-16	1.59E-15
CCL25	2.858694	-1.80652	2.29E-16	1.68E-15
IGLON5	2.334468	-0.48631	2.32E-16	1.70E-15
AC125611.2	2.081485	-2.59851	2.32E-16	1.70E-15
HOXC-AS3	5.791516	-1.11975	2.32E-16	1.70E-15
IGHV1-67	2.547626	0.378305	2.34E-16	1.71E-15
GCM1	2.755419	-1.32771	2.34E-16	1.71E-15
AL021920.2	3.31899	-2.2853	2.36E-16	1.72E-15
CHRNA9	5.086753	2.095524	2.41E-16	1.76E-15
IGHV3-52	2.488279	-0.46884	2.49E-16	1.82E-15
ELFN1-AS1	3.809064	-0.28726	2.49E-16	1.82E-15
LINC00519	2.305466	-1.62687	2.50E-16	1.82E-15
IGHV3-71	2.44763	-0.013	2.54E-16	1.85E-15
AC073316.2	3.834669	-1.79175	2.55E-16	1.86E-15
AC108676.1	2.663751	-0.59554	2.65E-16	1.93E-15
ACHE	2.432661	4.062663	2.67E-16	1.95E-15
LINC02588	5.714629	-0.39835	2.71E-16	1.97E-15
LY6K	3.657705	3.457739	2.73E-16	1.98E-15
AL139023.1	6.371328	-1.98982	2.75E-16	2.00E-15
IGHV4-28	2.293781	2.80849	2.80E-16	2.04E-15

For Peer Review

IGHV1-2	2.66275	5.461059	2.87E-16	2.08E-15
WDR97	2.023612	1.339892	2.91E-16	2.11E-15
AC106882.1	2.529217	-1.59806	2.99E-16	2.17E-15
HIST3H2A	2.493949	3.197071	3.14E-16	2.27E-15
PADI3	6.866065	2.28923	3.15E-16	2.27E-15
MEGF10	2.733998	0.349317	3.15E-16	2.28E-15
ALPK2	2.195606	0.716327	3.16E-16	2.28E-15
FSIP2	2.817338	1.694877	3.19E-16	2.30E-15
IGHV4-39	2.424427	6.416359	3.27E-16	2.36E-15
CLDN14	2.138468	-0.71607	3.27E-16	2.36E-15
GRIK2	2.602569	1.13439	3.30E-16	2.38E-15
HIST1H2BL	3.760845	-0.9423	3.34E-16	2.41E-15
KIRREL2	3.582933	0.409456	3.36E-16	2.42E-15
IGLV3-19	2.556587	6.838701	3.41E-16	2.46E-15
LYPD8	4.150334	-0.72403	3.45E-16	2.49E-15
BOLA2P3	2.391934	-3.09636	3.49E-16	2.51E-15
AC090164.3	3.725971	-2.63287	3.55E-16	2.55E-15
SPINK13	3.698501	1.005665	3.58E-16	2.58E-15
NMRAL2P	3.482768	2.440821	3.63E-16	2.61E-15
LINC01214	5.749061	-1.40945	3.65E-16	2.62E-15
AL157931.1	3.27709	-2.18301	3.66E-16	2.63E-15
SNRPCP1	5.192744	-2.49699	3.67E-16	2.63E-15
AGT	3.055786	4.951207	3.74E-16	2.69E-15
CRYBA2	5.190169	-1.35699	3.85E-16	2.76E-15
PIH1D3	-2.24228	-0.05807	3.87E-16	2.77E-15
ACTL8	7.786143	0.196456	3.88E-16	2.78E-15
IGKV2D-28	3.952064	-0.62645	4.00E-16	2.86E-15
IGKV3-20	2.245918	7.826467	4.05E-16	2.89E-15
HNF1A	3.782918	1.172782	4.20E-16	3.00E-15
LRRC14B	3.376123	-2.6591	4.24E-16	3.02E-15
CALML6	2.322257	-1.27755	4.30E-16	3.07E-15
AC023796.2	3.330384	-3.00947	4.30E-16	3.07E-15
HAL	2.638931	3.403179	4.38E-16	3.12E-15
AC018641.1	4.012136	-2.26927	4.43E-16	3.15E-15
IGLV1-47	2.315609	5.928254	4.47E-16	3.18E-15
IGLV1-50	2.454906	0.084884	4.54E-16	3.23E-15
HMGA1P1	2.49587	-3.02153	4.56E-16	3.24E-15
TTLL11-IT1	3.102082	-2.1008	4.58E-16	3.26E-15
IGBP1P4	3.686842	-1.93393	4.59E-16	3.26E-15
ERVV-1	4.420041	-1.75548	4.75E-16	3.38E-15
AC009275.1	2.380385	-0.64955	4.76E-16	3.38E-15
MMP9	2.003436	6.270841	4.76E-16	3.38E-15
TWIST1	2.452224	1.473525	4.95E-16	3.51E-15

For Peer Review

AC093281.2	4.163357	-2.99642	4.99E-16	3.54E-15
IGHV2-5	2.595991	3.26984	5.09E-16	3.61E-15
AL512413.1	2.028343	-1.58645	5.24E-16	3.71E-15
CTSV	2.26418	3.152	5.27E-16	3.73E-15
KCNJ10	2.104402	0.690429	5.37E-16	3.80E-15
GFY	5.929319	-0.90872	5.39E-16	3.81E-15
KIF3AP1	3.49513	-2.02729	5.54E-16	3.91E-15
DUXAP9	2.37368	-1.01695	5.54E-16	3.91E-15
FOXE1	4.400458	1.822315	5.60E-16	3.95E-15
CYCSP6	5.502883	-1.63252	5.66E-16	3.99E-15
CCDC154	2.469987	1.332964	5.78E-16	4.07E-15
MTHFD1P1	2.280825	-1.31242	5.96E-16	4.19E-15
GUCY1B2	2.180328	1.866182	6.00E-16	4.22E-15
SALRNA1	2.622664	-1.28539	6.08E-16	4.28E-15
PPY2P	5.359581	-2.60974	6.23E-16	4.38E-15
TRIM9	2.737566	1.724339	6.27E-16	4.40E-15
FETUB	5.715551	-1.81863	6.38E-16	4.47E-15
AC010329.2	5.223199	-2.308	6.40E-16	4.49E-15
AC092944.2	-2.10767	-3.68563	6.44E-16	4.51E-15
IGSF1	2.949115	0.956935	6.45E-16	4.52E-15
SNORD46	2.224219	-1.90596	6.50E-16	4.56E-15
TF	4.701767	5.449063	6.62E-16	4.64E-15
ZIC5	5.404934	-0.71345	6.65E-16	4.65E-15
AC012640.1	2.279992	-3.30587	6.93E-16	4.84E-15
ENTPD2	2.147392	2.047531	6.99E-16	4.88E-15
WNK2	2.055847	3.822757	7.05E-16	4.93E-15
LINC01615	2.22833	-1.27897	7.28E-16	5.08E-15
CILP	2.42557	4.315231	7.36E-16	5.13E-15
AKR1C2	4.600711	8.330465	7.40E-16	5.16E-15
GCGR	3.506014	-1.96799	7.50E-16	5.22E-15
PRRX2	2.17661	2.427238	7.53E-16	5.24E-15
LINC01971	3.2031	-3.00771	7.63E-16	5.31E-15
AGTR2	-2.40459	3.449709	7.66E-16	5.33E-15
IGKV1-17	2.415588	4.773094	8.25E-16	5.73E-15
LINP1	4.190682	0.27634	8.48E-16	5.89E-15
AL445471.1	2.232301	-2.40383	8.61E-16	5.97E-15
ERVH48-1	4.639745	2.284585	8.86E-16	6.14E-15
AC090954.1	3.584316	-3.04889	9.05E-16	6.27E-15
ADGRB1	3.151234	3.495627	9.08E-16	6.29E-15
CDK5R2	4.344644	0.559152	9.18E-16	6.35E-15
OR13Z2P	4.286293	-3.26324	9.35E-16	6.46E-15
CSAG1	7.132297	1.839436	9.44E-16	6.52E-15
IGLV2-8	2.41507	5.154906	9.54E-16	6.59E-15

For Peer Review

FGF5	5.685647	-0.25595	9.89E-16	6.82E-15
UGT2B11	7.274901	0.339652	1.03E-15	7.07E-15
HTR1D	2.678541	1.650411	1.04E-15	7.14E-15
NPTX1	3.714585	4.9715	1.07E-15	7.33E-15
AL031710.2	2.137673	-2.42048	1.07E-15	7.35E-15
AC092436.2	5.058747	-1.65474	1.09E-15	7.50E-15
TTC29	-2.10653	0.822596	1.09E-15	7.52E-15
SCARNA13	4.273304	1.949483	1.10E-15	7.58E-15
MUC5B	3.961045	9.44112	1.13E-15	7.74E-15
MUC4	2.450164	7.060957	1.13E-15	7.78E-15
SLC6A3	3.978336	3.170218	1.14E-15	7.83E-15
UGT8	2.159313	3.848213	1.15E-15	7.87E-15
AL136221.1	2.220206	-2.39135	1.15E-15	7.90E-15
FAR2P1	6.283263	0.709238	1.18E-15	8.09E-15
CYP2F1	-2.22822	0.756301	1.19E-15	8.16E-15
IGHV5-78	2.103587	-0.18946	1.19E-15	8.18E-15
FLJ12825	2.059197	-1.34805	1.20E-15	8.22E-15
PRSS46P	2.509524	-1.8125	1.21E-15	8.25E-15
HAGLROS	2.465744	-0.29764	1.21E-15	8.30E-15
IGLV3-1	2.382798	5.814185	1.24E-15	8.49E-15
AC127024.8	2.588494	-0.77131	1.26E-15	8.59E-15
AL513123.1	5.117686	-2.82081	1.26E-15	8.59E-15
PRAP1	4.403097	1.481632	1.26E-15	8.63E-15
PKIB	2.011095	3.922402	1.31E-15	8.92E-15
IGKV1-13	3.465062	-0.77714	1.32E-15	9.01E-15
LINC01529	3.979812	-1.73904	1.33E-15	9.04E-15
PNPLA5	5.827522	-1.60173	1.33E-15	9.04E-15
IGHV4-61	2.496365	2.68947	1.38E-15	9.40E-15
IGHV3-35	2.478281	0.191834	1.38E-15	9.40E-15
SCARNA12	3.881683	-0.26823	1.39E-15	9.48E-15
DUSP5P2	2.843627	-3.32683	1.41E-15	9.56E-15
AC008870.3	2.5992	-3.35102	1.47E-15	9.98E-15
AL356417.2	2.175616	-1.68289	1.47E-15	9.98E-15
AL391427.1	3.89993	-0.15259	1.49E-15	1.01E-14
TRIM17	2.148932	2.327354	1.51E-15	1.03E-14
MAGEC1	7.97391	1.272119	1.52E-15	1.03E-14
WDR38	-2.06857	2.006652	1.53E-15	1.03E-14
LNCAROD	5.775536	-1.24223	1.55E-15	1.05E-14
IGLC2	2.045599	8.505462	1.55E-15	1.05E-14
HIST2H2AC	2.815593	0.73227	1.58E-15	1.07E-14
IGKV1-16	2.699013	5.209608	1.59E-15	1.08E-14
CDX2	5.457991	0.110091	1.61E-15	1.09E-14
HIST1H2BJ	2.22913	1.388979	1.61E-15	1.09E-14

For Peer Review

LINC02324	2.716868	-3.10216	1.65E-15	1.12E-14
SULT4A1	3.825703	0.230468	1.67E-15	1.13E-14
SLC28A2	4.375529	2.387366	1.67E-15	1.13E-14
LINC01436	3.166883	3.059758	1.68E-15	1.13E-14
IGKV3D-20	2.35197	3.35871	1.71E-15	1.15E-14
KCNQ2	5.302286	0.769786	1.74E-15	1.17E-14
IGHV3-48	2.40064	3.791817	1.74E-15	1.17E-14
AC122134.1	5.430405	-2.16586	1.74E-15	1.17E-14
TLX1	5.274811	-2.0861	1.76E-15	1.18E-14
AL109761.1	2.622914	-2.06914	1.77E-15	1.19E-14
LINC02475	3.944006	-0.53691	1.79E-15	1.20E-14
IGHV4-4	2.820727	2.172427	1.85E-15	1.24E-14
Z73417.1	2.536236	0.369179	1.85E-15	1.24E-14
AL157400.2	3.051996	-3.64809	1.88E-15	1.26E-14
AC142381.1	2.370817	-0.21947	1.89E-15	1.27E-14
CALB1	5.650255	3.53306	2.03E-15	1.36E-14
AC025575.2	4.998971	-0.13729	2.05E-15	1.37E-14
SCN4A	3.276723	1.252084	2.05E-15	1.37E-14
ELAVL2	3.436297	0.043465	2.10E-15	1.40E-14
AC145676.1	2.335705	-1.38065	2.10E-15	1.41E-14
CEMIP	2.083888	6.113916	2.15E-15	1.44E-14
TMEM190	-2.11691	1.351174	2.21E-15	1.47E-14
NMNAT2	2.612954	4.227343	2.32E-15	1.54E-14
RIMS2	3.778246	1.250934	2.33E-15	1.55E-14
IGHV1-3	3.038192	1.528678	2.34E-15	1.56E-14
IGLV2-23	2.310372	6.978529	2.34E-15	1.56E-14
FAM86LP	2.694596	-2.74815	2.40E-15	1.59E-14
CIT	2.005118	7.06608	2.43E-15	1.62E-14
IGHV3-66	2.377107	2.670228	2.45E-15	1.63E-14
AC113404.1	-2.56943	-3.34379	2.46E-15	1.64E-14
CU639417.4	3.355848	-2.99488	2.48E-15	1.65E-14
WIF1	-2.26418	5.707166	2.57E-15	1.70E-14
IGHV3-41	2.359294	-0.39914	2.57E-15	1.70E-14
RNU6-1161P	2.388334	-2.41423	2.62E-15	1.73E-14
PGAM1P7	2.059189	-2.66193	2.72E-15	1.79E-14
IGHV3OR16-9	2.420094	-0.21435	2.74E-15	1.81E-14
DLX5	2.619259	0.474135	2.77E-15	1.83E-14
FANCD2OS	2.163602	-3.24084	2.80E-15	1.85E-14
LINC01792	3.410649	-3.16816	2.81E-15	1.85E-14
AL358115.1	2.065532	-1.84744	2.88E-15	1.90E-14
AL662890.1	2.633315	-2.39829	2.91E-15	1.92E-14
LINC00491	5.571682	-0.88333	2.94E-15	1.93E-14
CGA	8.822599	3.635747	3.00E-15	1.97E-14

For Peer Review

AL731684.1	3.941634	-2.95123	3.02E-15	1.99E-14
AC092969.1	3.870414	-1.40263	3.11E-15	2.04E-14
AL133284.1	4.870874	-0.98664	3.12E-15	2.05E-14
KLHDC8A	2.091048	1.180653	3.16E-15	2.08E-14
STPG3	2.218636	-0.36089	3.21E-15	2.11E-14
AC010275.1	4.698317	-2.23865	3.29E-15	2.16E-14
AL162431.1	2.713373	-2.38176	3.32E-15	2.18E-14
SNORA53	4.920871	1.083845	3.33E-15	2.19E-14
ESPNP	2.647434	-1.47541	3.33E-15	2.19E-14
AC025431.1	-2.10008	-3.91911	3.37E-15	2.21E-14
INSL4	8.095619	2.23081	3.49E-15	2.28E-14
C1orf158	-2.27701	0.494545	3.55E-15	2.32E-14
EGOT	2.444954	-0.53694	3.56E-15	2.33E-14
AC092920.1	3.507825	-3.61934	3.60E-15	2.36E-14
AC012501.1	5.174388	-3.0695	3.61E-15	2.36E-14
RASGEF1C	3.718731	-0.62597	3.61E-15	2.36E-14
IGHJ1	2.890825	-1.11338	3.66E-15	2.39E-14
NIFKP8	-2.17407	-3.84254	3.74E-15	2.44E-14
PNMA5	5.530881	1.694799	3.79E-15	2.47E-14
AC068189.1	2.5107	-3.45761	3.88E-15	2.53E-14
MYBPH	3.682361	2.094029	3.91E-15	2.55E-14
SLC9A3	2.415057	0.701626	3.91E-15	2.55E-14
MYH6	4.03767	-2.74978	3.94E-15	2.57E-14
AC012213.1	5.302797	-2.01317	4.02E-15	2.62E-14
IGKV1D-16	2.990436	2.476823	4.06E-15	2.64E-14
IGHV3-64	2.966457	2.42203	4.08E-15	2.65E-14
LINC01234	5.404116	0.298766	4.28E-15	2.78E-14
AC105206.1	-2.49312	-2.18066	4.29E-15	2.78E-14
CALCB	4.154699	0.295576	4.32E-15	2.80E-14
CALML3	6.057481	2.123291	4.40E-15	2.86E-14
LRRC15	2.277866	4.788748	4.45E-15	2.89E-14
BCL2L12P1	2.965819	-3.43556	4.52E-15	2.93E-14
HCAR1	2.474551	3.302882	4.54E-15	2.94E-14
IGLV2-28	2.424272	-0.69475	4.56E-15	2.95E-14
IGKV2OR22-4	2.575125	-0.08991	4.60E-15	2.98E-14
AC078820.1	3.228937	-2.05846	4.64E-15	3.01E-14
IGLL1	2.766801	-1.37445	4.64E-15	3.01E-14
BCL9P1	-2.07233	-3.48416	4.76E-15	3.08E-14
AP001574.1	2.753837	-0.89328	4.82E-15	3.12E-14
SLC7A9	2.911063	0.206161	4.88E-15	3.15E-14
AL117329.1	5.668289	-2.20654	4.98E-15	3.22E-14
STPG3-AS1	2.140929	-0.70254	5.14E-15	3.31E-14
ADAMDEC1	2.212859	3.529282	5.20E-15	3.35E-14

For Peer Review

ST8SIA2	3.302283	-0.63354	5.24E-15	3.38E-14
THRA1/BTR	4.524795	-1.8462	5.26E-15	3.39E-14
HIST1H4D	5.318923	-0.0163	5.28E-15	3.40E-14
LINC01775	2.272647	-3.37163	5.36E-15	3.45E-14
GTSF1L	2.497247	-3.40219	5.59E-15	3.60E-14
AL049555.1	3.249565	1.327699	5.81E-15	3.73E-14
AC141930.1	2.615334	-1.28063	5.84E-15	3.75E-14
COL25A1	4.39227	3.025414	5.93E-15	3.81E-14
LINC02043	2.427438	-2.98368	5.93E-15	3.81E-14
COL9A3	2.286707	1.741429	5.96E-15	3.83E-14
AL356234.2	4.21903	-2.04699	6.04E-15	3.88E-14
AC010595.1	5.566628	-2.4051	6.06E-15	3.89E-14
SLCO1B1	6.258745	-1.22236	6.09E-15	3.90E-14
IGHV3OR16-8	2.427825	-0.36311	6.21E-15	3.98E-14
FTCD	2.849573	-0.41685	6.21E-15	3.98E-14
AC090192.2	4.359797	-2.7336	6.23E-15	3.99E-14
PPEF1	2.171468	0.531822	6.26E-15	4.01E-14
CAPSL	-2.09142	1.616548	6.39E-15	4.08E-14
AC097537.1	-2.68726	-3.94442	6.52E-15	4.17E-14
LINC01287	5.819339	0.768559	6.56E-15	4.20E-14
AC120036.3	2.294926	-2.12313	6.59E-15	4.21E-14
SNORD15B	4.178329	0.187991	6.72E-15	4.29E-14
IGHV3OR15-7	2.451751	-0.01248	6.81E-15	4.34E-14
HIST1H3F	6.641743	-0.81104	6.96E-15	4.43E-14
HOXA13	6.431331	-0.19282	7.01E-15	4.46E-14
AFP	8.493696	2.393104	7.07E-15	4.50E-14
AC104461.1	2.759383	-0.93495	7.15E-15	4.54E-14
AC114489.1	3.998108	-2.74925	7.20E-15	4.58E-14
LINC01978	2.150895	-1.87577	7.25E-15	4.60E-14
IGHV3OR16-13	2.4412	0.120375	7.48E-15	4.74E-14
LINC01305	5.140781	-2.11955	7.55E-15	4.79E-14
AC024940.1	2.400542	-0.25598	7.61E-15	4.82E-14
SUN3	3.990987	0.012701	7.81E-15	4.95E-14
PNMA3	2.640892	1.194743	7.86E-15	4.98E-14
AC135068.8	3.872198	-2.01082	7.89E-15	5.00E-14
KLK12	6.113204	5.102319	8.10E-15	5.13E-14
CREG2	2.78278	0.753827	8.15E-15	5.16E-14
LINC00626	3.400234	-2.85305	8.16E-15	5.16E-14
STEAP1B	2.393946	0.803422	8.16E-15	5.16E-14
PRDM13	5.272096	-2.24174	8.19E-15	5.18E-14
AC003965.2	2.070203	-1.68096	8.22E-15	5.20E-14
AC022182.1	2.012065	-1.86787	8.25E-15	5.21E-14
CDH15	2.150266	2.210404	8.33E-15	5.26E-14

For Peer Review

LINC01968	2.582995	-3.37324	8.47E-15	5.35E-14
KHDC1L	5.29762	-0.81697	8.49E-15	5.37E-14
SP5	2.328267	2.460835	8.69E-15	5.49E-14
AC011352.1	3.513076	-3.46875	8.70E-15	5.49E-14
AL133370.1	5.459393	-0.41271	8.80E-15	5.55E-14
IGKV1OR22-1	2.854763	-0.22407	8.91E-15	5.62E-14
LINC02418	7.491305	1.57368	8.96E-15	5.65E-14
LINC01393	2.015212	-1.47284	8.98E-15	5.66E-14
POU6F2	3.949897	2.161818	9.01E-15	5.68E-14
LINC00941	3.183609	0.527039	9.15E-15	5.76E-14
AL136981.1	4.242489	-2.0224	9.33E-15	5.87E-14
AC135068.2	2.442721	-0.56349	9.35E-15	5.88E-14
OLFM4	6.422528	3.883651	9.36E-15	5.88E-14
FCRL4	2.450087	-0.95722	9.43E-15	5.93E-14
INA	4.116891	1.766872	9.66E-15	6.06E-14
AC129507.4	3.540482	0.476137	9.77E-15	6.13E-14
AC020928.1	2.840094	-0.81665	9.97E-15	6.25E-14
LINC02065	3.205279	-3.3203	9.98E-15	6.26E-14
C3orf30	3.64342	-2.11552	9.99E-15	6.26E-14
KCNE4	2.261842	4.481583	1.02E-14	6.39E-14
AKR1C4	5.016005	1.019244	1.03E-14	6.46E-14
AC005537.1	3.545255	-0.70292	1.04E-14	6.54E-14
SNORA23	6.654067	1.081829	1.08E-14	6.74E-14
AOX2P	2.888557	-3.45498	1.08E-14	6.75E-14
LINC02562	2.198415	1.613771	1.09E-14	6.83E-14
IGKV1OR22-5	2.716602	-1.15583	1.10E-14	6.86E-14
KCP	2.148394	2.193431	1.12E-14	7.01E-14
LINC01587	2.892005	-1.8292	1.17E-14	7.31E-14
AC012531.1	3.547092	-1.88506	1.18E-14	7.33E-14
LINC01611	5.596145	-2.63155	1.19E-14	7.42E-14
POU6F2-AS2	6.181821	-1.49187	1.22E-14	7.56E-14
DUXAP10	2.600753	-0.98442	1.22E-14	7.58E-14
MIR137HG	4.887468	-1.77554	1.22E-14	7.58E-14
LINC02387	3.345318	-1.77722	1.22E-14	7.58E-14
AC026462.3	5.274076	-2.43217	1.22E-14	7.60E-14
GRM4	3.296196	0.349361	1.24E-14	7.71E-14
IVL	4.278453	3.087492	1.25E-14	7.74E-14
AKAP14	-2.0811	0.044297	1.25E-14	7.76E-14
CFHR3	2.076878	0.206435	1.32E-14	8.19E-14
AC122710.3	2.386209	-2.91986	1.34E-14	8.31E-14
HOXA11-AS	4.354812	-1.38528	1.40E-14	8.64E-14
UBE2QL1	2.37401	0.847508	1.40E-14	8.65E-14
KRT3	3.519203	-1.80478	1.41E-14	8.69E-14

For Peer Review

MIR9-3HG	2.666806	2.072642	1.44E-14	8.89E-14
IGLV3-9	2.552224	4.137016	1.44E-14	8.89E-14
MAGEA3	8.118929	3.15668	1.45E-14	8.92E-14
KRT78	3.168716	-0.66438	1.51E-14	9.32E-14
A2ML1	4.640901	2.272537	1.55E-14	9.54E-14
SLC44A5	2.435189	3.651714	1.58E-14	9.71E-14
AC010329.5	4.906974	-2.24239	1.58E-14	9.74E-14
LINC01564	2.863677	-0.38994	1.66E-14	1.02E-13
AC127164.1	2.420235	-0.97392	1.66E-14	1.02E-13
LINC02015	2.627777	0.008166	1.70E-14	1.04E-13
IGHV3-49	2.306266	4.956982	1.70E-14	1.04E-13
IGHV1OR16-1	2.86701	-1.71981	1.74E-14	1.07E-13
SLC28A1	2.20737	-1.95328	1.85E-14	1.13E-13
AC144450.1	2.54781	-1.05127	1.88E-14	1.15E-13
BHMT2	2.852236	4.010369	1.88E-14	1.15E-13
TMEM212	-2.21924	0.097016	1.89E-14	1.16E-13
AC025166.1	2.378052	-3.45929	1.90E-14	1.16E-13
SLC22A8	-2.36399	-3.86076	1.93E-14	1.18E-13
AL513327.2	2.406286	-2.90472	1.97E-14	1.20E-13
AC112721.1	2.736992	-2.75203	1.97E-14	1.20E-13
IGKV1-39	2.814719	1.53681	2.03E-14	1.24E-13
PLK5	3.512943	-2.52214	2.07E-14	1.26E-13
AL022324.3	3.555403	-3.40089	2.07E-14	1.26E-13
SLC6A11	3.703782	0.923295	2.10E-14	1.28E-13
AC019155.3	4.850934	-1.55725	2.11E-14	1.29E-13
AC011604.2	5.37537	-2.92938	2.19E-14	1.33E-13
AC034206.1	3.645063	-2.51409	2.20E-14	1.34E-13
HOXC6	2.366747	1.491384	2.23E-14	1.36E-13
AC006262.1	2.455948	-1.0755	2.36E-14	1.43E-13
AC131009.2	3.70315	-2.97586	2.37E-14	1.44E-13
AQP6	2.833146	-0.09248	2.37E-14	1.44E-13
IGHV3-21	2.225978	5.805218	2.38E-14	1.45E-13
AC040174.1	4.367665	-2.06497	2.40E-14	1.46E-13
SLC13A5	3.379205	0.564262	2.44E-14	1.48E-13
C18orf63	-2.08726	-2.20987	2.45E-14	1.49E-13
AF127577.3	5.991807	-1.51879	2.46E-14	1.49E-13
FABP7	5.816564	1.917476	2.48E-14	1.50E-13
AC114803.1	4.091071	-3.71809	2.49E-14	1.51E-13
VSTM2L	2.071257	6.188221	2.50E-14	1.52E-13
AC126696.3	2.950291	-3.38404	2.57E-14	1.56E-13
RPS16P9	4.687254	-2.1816	2.58E-14	1.56E-13
SLC25A21	2.146569	1.119697	2.64E-14	1.60E-13
AL445183.2	3.407168	-0.54557	2.65E-14	1.61E-13

For Peer Review

BTNL10	2.045682	-0.78584	2.68E-14	1.62E-13
PPBP	-2.03378	1.141781	2.69E-14	1.63E-13
DNAI2	-2.01607	1.534846	2.70E-14	1.63E-13
TMPRSS11D	4.153515	-0.47606	2.76E-14	1.67E-13
AC125603.2	4.758084	0.685856	2.84E-14	1.71E-13
IGHV2-70	2.472597	3.529461	2.88E-14	1.73E-13
COL2A1	5.662656	3.658433	2.88E-14	1.73E-13
TEX45	2.056861	-0.34163	2.92E-14	1.76E-13
LINC02398	3.40974	-1.83023	2.94E-14	1.77E-13
TIMM8AP1	2.308816	0.059063	2.98E-14	1.79E-13
ONECUT3	5.792039	0.58407	2.99E-14	1.80E-13
ADGB	-2.06629	0.281564	2.99E-14	1.80E-13
AC105450.1	3.146285	-3.12564	3.00E-14	1.80E-13
PLD5	2.747014	2.449621	3.05E-14	1.83E-13
RNF225	2.53548	-1.15461	3.17E-14	1.90E-13
CA7	2.64743	-2.52156	3.19E-14	1.91E-13
CEACAM8	-2.17501	-0.24561	3.29E-14	1.97E-13
DLX3	2.174881	1.942246	3.29E-14	1.97E-13
BPIFA1	5.171272	8.794411	3.29E-14	1.97E-13
NCAPD2P1	4.250324	-1.88366	3.37E-14	2.02E-13
ALG1L3P	3.886959	-1.95611	3.38E-14	2.02E-13
SYNPR	4.440717	-2.11444	3.38E-14	2.02E-13
LPO	2.341101	-0.81385	3.39E-14	2.03E-13
AC100791.2	3.37541	-3.60335	3.43E-14	2.06E-13
IGFL4	2.711976	-1.64861	3.47E-14	2.07E-13
KRT4	-2.10561	3.838017	3.47E-14	2.07E-13
AC083973.1	2.486494	-2.51111	3.50E-14	2.09E-13
LINC02428	4.289598	-2.44937	3.51E-14	2.10E-13
HIST1H4B	5.345149	-0.63732	3.52E-14	2.10E-13
LINC01213	3.858567	-2.69654	3.54E-14	2.11E-13
IGHV3-76	2.572938	-1.44172	3.72E-14	2.22E-13
LINC01843	2.207378	0.247762	3.74E-14	2.23E-13
SNORD72	2.06925	-3.14949	3.82E-14	2.27E-13
SCARNA5	6.649091	3.120721	3.86E-14	2.30E-13
TAC4	2.802346	0.708748	3.89E-14	2.31E-13
AC005993.1	4.695859	-1.42203	3.90E-14	2.32E-13
EPHA8	3.87054	-2.06738	3.90E-14	2.32E-13
IGHV3OR16-10	2.530326	-1.48337	3.96E-14	2.36E-13
IGKV1-27	2.268475	4.625294	4.05E-14	2.41E-13
AC010343.3	4.030115	-2.45522	4.18E-14	2.48E-13
GPR78	2.497191	-0.73557	4.23E-14	2.51E-13
HRG	4.797704	-1.36207	4.39E-14	2.60E-13
AC026412.2	2.02723	-1.62838	4.47E-14	2.64E-13

For Peer Review

IGHV1-14	2.936948	-0.80658	4.48E-14	2.65E-13
IGHV3-62	2.347628	-1.13959	4.48E-14	2.65E-13
CDA	2.446061	4.054085	4.54E-14	2.68E-13
STOML3	-2.08247	1.239975	4.65E-14	2.75E-13
CELF3	4.454704	1.59525	4.70E-14	2.77E-13
IGLV3-29	3.424772	-1.61402	4.71E-14	2.78E-13
AC010168.1	2.195955	-2.06196	4.72E-14	2.79E-13
FAM83B	3.258103	1.236917	4.80E-14	2.83E-13
IGHV3-19	2.229333	-0.39626	5.10E-14	3.00E-13
USH1C	3.934005	2.596072	5.14E-14	3.02E-13
GCNT1P4	4.061735	-3.41818	5.14E-14	3.02E-13
HIST1H2AB	5.630998	-1.35073	5.16E-14	3.04E-13
LINC01876	2.241266	0.387864	5.17E-14	3.04E-13
AC004925.1	2.447116	-1.54605	5.20E-14	3.05E-13
XAGE1B	3.142669	-2.4015	5.23E-14	3.07E-13
AP003500.1	3.960355	-2.99796	5.31E-14	3.12E-13
C20orf85	-2.26585	3.482418	5.36E-14	3.14E-13
MIR148A	2.574762	-3.41319	5.46E-14	3.20E-13
AC092484.1	4.988659	-1.55955	5.52E-14	3.23E-13
PNMA6A	2.111871	1.307005	5.53E-14	3.24E-13
IGKV1OR9-2	2.998454	-2.59676	5.54E-14	3.24E-13
MUC2	5.724682	2.555208	5.68E-14	3.32E-13
APCDD1L-DT	3.046226	-0.56937	6.00E-14	3.50E-13
AC092964.1	2.887692	-1.15259	6.13E-14	3.58E-13
FP325330.3	4.696927	-3.21072	6.13E-14	3.58E-13
NTSR1	4.603966	1.244938	6.14E-14	3.58E-13
DEFB131E	3.128595	-3.88984	6.18E-14	3.61E-13
NKAIN3-IT1	4.508186	-0.49751	6.27E-14	3.65E-13
ZG16B	2.512683	2.835849	6.30E-14	3.67E-13
SCARNA10	6.320645	2.342315	6.37E-14	3.71E-13
IGKV2-40	4.431945	-2.94716	6.46E-14	3.76E-13
AC026355.2	4.608455	-3.17404	6.61E-14	3.85E-13
IGLV3-10	2.518776	5.421892	6.63E-14	3.85E-13
HOXC12	8.1178	0.999502	6.79E-14	3.94E-13
IGKV2D-29	2.526751	3.428043	6.80E-14	3.95E-13
RGR	-2.10622	-3.9798	6.81E-14	3.95E-13
AC108451.2	3.954064	-1.24348	6.83E-14	3.96E-13
AP004608.1	2.958545	2.889687	6.90E-14	4.00E-13
LINC01518	5.915764	-2.0685	6.94E-14	4.03E-13
AC087667.1	3.180695	-2.14748	7.12E-14	4.12E-13
MAGEA8	5.462452	0.33443	7.21E-14	4.17E-13
AC022098.2	2.778441	-0.56418	7.23E-14	4.18E-13
FNDC1-IT1	2.931657	-3.55122	7.33E-14	4.24E-13

For Peer Review

HIST2H2AB	5.060956	-0.86683	7.35E-14	4.25E-13
IGKV1-6	2.104678	4.379671	7.39E-14	4.27E-13
AC027627.1	3.725079	-3.67601	7.41E-14	4.28E-13
IGKV3D-7	3.349733	-1.41129	7.43E-14	4.29E-13
AP000526.1	3.117511	-3.25158	7.43E-14	4.29E-13
Z99129.3	2.248321	-2.24433	7.45E-14	4.31E-13
LINC00682	5.27175	-2.65942	7.74E-14	4.47E-13
USH1G	3.370671	-1.18688	8.21E-14	4.73E-13
AC090125.1	3.887221	-3.23421	8.41E-14	4.84E-13
WTAPP1	2.278715	-1.06499	8.58E-14	4.94E-13
SRGAP3-AS2	-2.27954	1.664264	8.59E-14	4.94E-13
IGKV1D-8	2.243054	1.777797	8.65E-14	4.98E-13
IGFL2-AS1	4.093863	-0.99954	8.72E-14	5.02E-13
TLX2	2.805178	-1.88287	9.26E-14	5.32E-13
HOXC-AS1	3.258046	-1.87002	9.40E-14	5.40E-13
SALL3	5.472973	-0.13834	9.58E-14	5.50E-13
KRT20	7.644041	4.105678	9.63E-14	5.52E-13
WNT16	3.891168	1.60314	9.78E-14	5.61E-13
SCARNA22	3.786249	-2.41039	9.78E-14	5.61E-13
IGKV2D-40	2.824764	2.12109	9.84E-14	5.64E-13
SLC35F3	2.13993	1.620761	1.03E-13	5.88E-13
EVX1	5.081395	-2.24888	1.04E-13	5.92E-13
ACTG1P22	2.662965	-2.11799	1.04E-13	5.95E-13
IGKV1OR2-6	2.642724	0.162995	1.05E-13	5.98E-13
DLX1	3.327208	-2.087	1.06E-13	6.05E-13
NFE4	4.250128	1.064817	1.07E-13	6.09E-13
RETNLB	4.255923	-2.73472	1.09E-13	6.24E-13
SNORA71C	2.390713	-1.39889	1.11E-13	6.33E-13
AL589642.2	4.598475	-3.33093	1.11E-13	6.33E-13
IGHV3-7	2.110298	1.927575	1.11E-13	6.33E-13
ADGRG7	6.339269	-0.63033	1.14E-13	6.51E-13
AC116345.1	5.08083	-1.05609	1.18E-13	6.70E-13
NAT16	3.08837	-2.19155	1.20E-13	6.81E-13
CNGB3	2.767256	-1.09959	1.20E-13	6.82E-13
IGHV3-20	2.414668	1.835636	1.21E-13	6.87E-13
OR5E1P	-2.07415	-3.89941	1.22E-13	6.92E-13
ZBTB8B	2.640774	-0.84943	1.24E-13	7.06E-13
MC4R	2.675513	-2.74885	1.25E-13	7.10E-13
AL035252.2	3.324541	-1.81495	1.26E-13	7.14E-13
ANKRD18B	2.77835	0.37103	1.27E-13	7.21E-13
KCNK9	3.752497	-0.41402	1.28E-13	7.26E-13
TGM5	3.019066	-1.00062	1.28E-13	7.27E-13
VTCN1	2.809858	3.400717	1.32E-13	7.47E-13

For Peer Review

AC136428.1	2.393365	-0.3001	1.34E-13	7.56E-13
C5orf46	2.078605	0.308043	1.34E-13	7.56E-13
JAKMIP3	2.222651	1.180051	1.35E-13	7.62E-13
ZMAT4	4.790554	0.611844	1.36E-13	7.67E-13
LEMD1-AS1	2.269268	-1.8577	1.36E-13	7.69E-13
STRCP1	2.099658	-1.95595	1.37E-13	7.71E-13
PRAMENP	2.691069	-1.12193	1.38E-13	7.80E-13
C1QL2	4.950771	1.402015	1.48E-13	8.35E-13
CALML5	7.438659	2.540437	1.49E-13	8.36E-13
RAET1L	4.01518	-1.86886	1.49E-13	8.36E-13
AL359764.1	4.010937	-2.4449	1.50E-13	8.45E-13
CALY	3.965026	0.610055	1.51E-13	8.51E-13
IGHV3-47	2.091252	-0.53336	1.53E-13	8.58E-13
IGHV1-69	2.582189	4.039814	1.53E-13	8.61E-13
HEPHL1	3.037211	0.605864	1.55E-13	8.68E-13
ETNPPL	6.164813	0.588183	1.61E-13	9.02E-13
AL049874.3	3.109377	-3.1085	1.61E-13	9.02E-13
SLC30A2	2.926372	0.214248	1.64E-13	9.17E-13
LL22NC03-63E9.3	3.301692	-3.21186	1.66E-13	9.28E-13
AC021087.3	3.116174	-2.99872	1.71E-13	9.55E-13
AC024901.1	2.814437	-1.44235	1.72E-13	9.62E-13
HIST1H2BI	6.291799	-0.99866	1.72E-13	9.63E-13
CA10	4.905623	1.368059	1.73E-13	9.66E-13
NLRP11	4.208051	-0.28071	1.73E-13	9.66E-13
AC004264.1	2.00286	-0.67195	1.75E-13	9.79E-13
LRRIQ4	2.053272	-0.6324	1.77E-13	9.86E-13
SBSN	4.347336	1.036141	1.80E-13	1.00E-12
ERICH3	-2.07648	2.729489	1.80E-13	1.00E-12
SYT13	4.134306	4.056359	1.84E-13	1.02E-12
C6orf15	5.704828	-1.11183	1.84E-13	1.02E-12
AC022733.1	4.529676	1.150764	1.85E-13	1.03E-12
LINC00676	10.04271	2.103452	1.90E-13	1.05E-12
IGHJ3	2.079307	1.214821	1.93E-13	1.07E-12
A1CF	4.445361	-0.30653	1.94E-13	1.08E-12
SLC38A11	2.725925	0.912905	1.94E-13	1.08E-12
AC126323.1	3.137294	0.437739	2.00E-13	1.11E-12
IGKV1D-39	2.996825	0.658641	2.05E-13	1.13E-12
SNORA38B	4.000092	-2.21718	2.05E-13	1.13E-12
PAX7	6.399197	3.414177	2.09E-13	1.15E-12
IGHV3-73	2.788759	4.932211	2.10E-13	1.16E-12
BTBD16	2.285715	0.086695	2.10E-13	1.16E-12
AC012213.4	4.82267	-2.32879	2.18E-13	1.20E-12

For Peer Review

AC024937.2	2.119612	-2.50785	2.22E-13	1.23E-12
RNU4-1	7.120567	2.546649	2.23E-13	1.23E-12
TEX19	4.617647	-0.5029	2.23E-13	1.23E-12
AP000977.1	2.264439	-2.70594	2.26E-13	1.25E-12
SLC5A5	3.486869	2.257456	2.30E-13	1.27E-12
EMX1	3.266516	-1.52379	2.42E-13	1.33E-12
AL122019.1	3.867225	-2.85419	2.42E-13	1.33E-12
CABP7	2.133283	-0.0902	2.42E-13	1.33E-12
AC211433.1	2.725516	-0.532	2.43E-13	1.34E-12
AC244205.1	2.102686	-1.40019	2.44E-13	1.34E-12
IGHV4-31	2.289874	4.376993	2.47E-13	1.36E-12
AL590004.3	2.572496	1.00941	2.47E-13	1.36E-12
AF015262.1	3.543308	-3.6032	2.48E-13	1.36E-12
CDK8P2	4.168948	-2.00255	2.49E-13	1.37E-12
SNORD94	2.436611	-0.39962	2.50E-13	1.37E-12
IGKV7-3	2.462568	-1.70722	2.51E-13	1.38E-12
LINC00567	3.812935	-1.16129	2.53E-13	1.39E-12
CNTNAP4	4.454925	-1.46926	2.55E-13	1.40E-12
KRT75	6.25025	0.334669	2.58E-13	1.41E-12
AGGF1P1	2.516835	-3.38155	2.59E-13	1.42E-12
SLC9A4	5.638291	0.301786	2.62E-13	1.44E-12
AC112907.2	2.089233	-2.48196	2.65E-13	1.45E-12
AC103563.3	2.518534	-0.60327	2.67E-13	1.46E-12
DPP10-AS1	3.217688	0.609999	2.71E-13	1.48E-12
MAGEA12	7.319451	2.275067	2.79E-13	1.53E-12
AC106799.1	4.566678	-3.45876	2.80E-13	1.53E-12
AC104687.2	2.340048	-3.02079	2.82E-13	1.54E-12
IGLV3-32	2.845178	-2.64407	2.85E-13	1.56E-12
PRKCG	2.556379	-1.27234	2.88E-13	1.57E-12
IGLV1-36	2.808189	3.611305	2.88E-13	1.57E-12
IGKV1OR2-11	2.74315	-1.72456	2.89E-13	1.57E-12
RF02247	3.837516	-3.72333	2.95E-13	1.61E-12
TNNC2	2.991814	3.207332	2.99E-13	1.63E-12
RN7SL313P	2.945653	-3.90408	3.06E-13	1.67E-12
SERPINB4	4.979617	1.006083	3.07E-13	1.67E-12
LINC02365	3.285185	-1.44289	3.09E-13	1.68E-12
AL139385.1	2.049162	-0.21752	3.10E-13	1.69E-12
AC015909.1	2.406296	-2.91228	3.11E-13	1.69E-12
DDX4	2.661532	-2.68351	3.12E-13	1.70E-12
PI15	2.443191	3.420605	3.17E-13	1.72E-12
JPH3	2.884267	1.022788	3.17E-13	1.72E-12
MAGEC2	7.737761	1.767001	3.18E-13	1.73E-12
RN7SL4P	3.681667	2.156036	3.19E-13	1.73E-12

For Peer Review

MAGEA6	8.076454	2.834093	3.20E-13	1.74E-12
IGHA2	2.05246	8.300262	3.23E-13	1.75E-12
ASNSP1	6.3628	-1.41621	3.29E-13	1.78E-12
AC022148.1	3.31653	-0.94505	3.46E-13	1.87E-12
DNAH9	-2.03549	3.218651	3.46E-13	1.87E-12
HOXC8	2.657126	-0.00695	3.48E-13	1.88E-12
LINC01633	5.018689	-3.00491	3.58E-13	1.93E-12
PLAC4	4.45056	2.588727	3.59E-13	1.94E-12
HOXD4	2.800526	-1.03032	3.59E-13	1.94E-12
MAGEA10	7.766783	1.872875	3.66E-13	1.97E-12
AL354993.2	3.233948	-2.15736	3.69E-13	1.99E-12
AC106785.2	6.065319	-2.08986	3.71E-13	2.00E-12
RET	2.918887	3.592584	3.71E-13	2.00E-12
CAMK2B	2.921246	1.525956	3.71E-13	2.00E-12
RNU4-2	6.839775	4.164178	3.73E-13	2.01E-12
AC007368.1	4.125718	-1.15648	3.74E-13	2.01E-12
BNIP3P24	2.935346	-3.01672	3.78E-13	2.04E-12
GLYATL2	4.523451	0.84031	3.90E-13	2.10E-12
IGKV5-2	2.700788	2.170892	3.91E-13	2.10E-12
F5	2.353681	4.326832	3.98E-13	2.14E-12
ERVV-2	5.632314	-2.07523	4.04E-13	2.17E-12
AC010894.3	3.178298	-0.29671	4.05E-13	2.18E-12
IGHV3-42	2.450007	-1.98063	4.13E-13	2.22E-12
AD000813.1	3.011561	-3.42173	4.18E-13	2.24E-12
AC087045.2	2.152031	-2.37122	4.24E-13	2.27E-12
EPS15P1	2.970858	-2.3991	4.30E-13	2.30E-12
SNORA79B	4.864984	0.328595	4.33E-13	2.32E-12
ZNF280A	5.098464	-2.06283	4.37E-13	2.34E-12
AC022784.6	2.397233	-2.66132	4.39E-13	2.35E-12
AC004884.2	2.418613	-1.60536	4.45E-13	2.38E-12
SOHLH1	5.457901	-1.74657	4.50E-13	2.41E-12
PANCR	5.609685	-2.59524	4.52E-13	2.42E-12
LINC02490	4.929266	-3.05974	4.67E-13	2.49E-12
UNC13A	2.731517	1.997471	4.71E-13	2.51E-12
IGLV5-45	2.398771	3.234574	4.73E-13	2.52E-12
OPRD1	2.258866	1.005494	4.74E-13	2.53E-12
MUC16	2.864931	5.960081	4.77E-13	2.54E-12
FOXE3	3.118963	-2.56737	4.80E-13	2.56E-12
SNORA71D	6.896538	-0.92568	4.94E-13	2.63E-12
GABRQ	3.452044	-0.86194	5.00E-13	2.66E-12
IGHV3-60	2.155502	-0.70489	5.02E-13	2.67E-12
CEL	3.193254	2.170586	5.10E-13	2.71E-12
AC087273.1	3.852311	-3.63433	5.18E-13	2.75E-12

For Peer Review

VSIG1	3.476458	5.893275	5.20E-13	2.76E-12
THEG	3.43091	-1.26891	5.20E-13	2.76E-12
ANKRD40CL	4.214746	-2.29413	5.35E-13	2.84E-12
HIST1H4J	2.0655	-2.35477	5.49E-13	2.91E-12
LINC00518	4.362053	-3.42492	5.49E-13	2.91E-12
HIST1H4A	5.18697	-1.81746	5.59E-13	2.96E-12
AP000696.1	4.057129	-3.11789	5.59E-13	2.96E-12
IGLV2-33	2.492359	-1.38942	5.60E-13	2.97E-12
ANKRD7	2.062388	-1.54946	5.61E-13	2.97E-12
LINC00342	2.004839	4.410514	5.62E-13	2.98E-12
AP003900.1	5.879666	-2.55317	5.63E-13	2.98E-12
LARP1P1	2.471824	-3.59105	5.70E-13	3.02E-12
DMRT1	4.303236	-2.2698	6.09E-13	3.22E-12
SLC14A2	4.150739	2.794693	6.31E-13	3.33E-12
IGHV2-70D	2.876484	2.903496	6.35E-13	3.35E-12
AC008268.1	-2.14477	2.948695	6.39E-13	3.37E-12
LINC01807	5.017772	-2.01762	6.46E-13	3.41E-12
BCAR4	6.352326	-0.21401	6.53E-13	3.44E-12
IGHV3-36	2.541199	-3.02016	6.54E-13	3.45E-12
HIST1H2AH	5.357954	-1.12388	6.59E-13	3.47E-12
RNY4P19	2.758523	-2.89448	6.64E-13	3.49E-12
MIR34B	-2.36369	-3.91214	6.81E-13	3.58E-12
MIA	3.922924	0.554623	6.91E-13	3.63E-12
SSTR5-AS1	5.176078	-1.06001	6.92E-13	3.64E-12
AC109446.3	2.849212	-0.20025	6.94E-13	3.65E-12
PRAC2	5.114864	-2.0819	7.18E-13	3.77E-12
AC096656.1	5.147778	-2.83566	7.36E-13	3.86E-12
KCNU1	6.342965	-0.74233	7.66E-13	4.01E-12
HIST1H3A	3.695373	-1.49405	7.68E-13	4.02E-12
SNORA12	4.671124	0.378314	7.68E-13	4.02E-12
IGHV1OR16-3	2.92816	-2.0291	7.82E-13	4.09E-12
AL596223.1	3.324966	-2.29316	8.09E-13	4.23E-12
RNF222	2.516776	-2.10082	8.16E-13	4.27E-12
FAR2P4	5.655511	-1.17331	8.17E-13	4.27E-12
HIST1H3I	5.736617	-1.4365	8.26E-13	4.31E-12
F7	2.940726	0.227395	8.27E-13	4.32E-12
SLC6A17	2.552466	0.173552	8.30E-13	4.33E-12
IL17REL	2.46596	-0.07944	8.36E-13	4.36E-12
SLC38A3	3.053831	0.987262	8.40E-13	4.38E-12
KLF2P1	5.812826	-1.93518	8.62E-13	4.49E-12
AMBP	3.520965	3.590222	8.68E-13	4.52E-12
IGHV3OR16-6	2.366546	-0.99993	8.98E-13	4.68E-12
SYT6	2.576002	-1.52824	9.13E-13	4.75E-12

For Peer Review

IGKV6-21	2.635512	2.857696	9.20E-13	4.78E-12
LIN28B	7.690555	-0.2007	9.24E-13	4.80E-12
CDHR5	3.593943	1.966446	9.31E-13	4.83E-12
CAB39P1	2.805679	-3.87387	9.31E-13	4.84E-12
LBP	4.893664	2.986961	9.42E-13	4.89E-12
LINC01511	5.733143	0.277622	9.52E-13	4.94E-12
SPRR2D	4.867374	1.824983	9.74E-13	5.05E-12
VWDE	2.040855	2.032898	9.84E-13	5.10E-12
ADGRF1	2.385586	6.302559	1.01E-12	5.22E-12
KCNC2	5.942796	-0.4334	1.01E-12	5.22E-12
AL133325.3	5.584656	-2.30363	1.04E-12	5.36E-12
B3GAT1	2.249301	2.567765	1.05E-12	5.43E-12
C10orf90	3.806186	-1.63751	1.05E-12	5.45E-12
LINC02594	3.002351	-3.04094	1.05E-12	5.45E-12
IGHV4-59	2.073614	5.923639	1.05E-12	5.45E-12
AC021218.1	3.094754	-0.17017	1.06E-12	5.48E-12
IGLVIVOR22-1	3.372544	-3.6983	1.08E-12	5.58E-12
AC103563.1	2.627173	-1.07784	1.09E-12	5.61E-12
AC058791.1	3.196112	0.927703	1.11E-12	5.71E-12
AC008083.1	2.349165	-2.70171	1.12E-12	5.77E-12
IGKV1OR2-3	2.63575	-1.59274	1.13E-12	5.81E-12
LINC01385	5.24167	-2.84317	1.14E-12	5.84E-12
AC024560.1	2.409203	-2.13427	1.14E-12	5.85E-12
AC114488.1	2.078974	-1.63593	1.14E-12	5.85E-12
INSRR	2.871887	-1.67231	1.15E-12	5.91E-12
GS1-594A7.3	2.837042	-2.41371	1.16E-12	5.96E-12
IGKV2-28	2.700384	0.320446	1.17E-12	5.99E-12
HOXB8	3.470139	1.635027	1.17E-12	6.00E-12
AC005330.1	2.27951	-1.96967	1.17E-12	6.02E-12
IGKV1OR2-9	2.714683	-2.7041	1.19E-12	6.09E-12
CLDN9	2.192635	2.869859	1.24E-12	6.33E-12
AC009097.2	2.122064	-3.09174	1.24E-12	6.37E-12
ALX3	4.829603	-2.0443	1.26E-12	6.45E-12
AC027281.1	-2.06531	-1.48941	1.27E-12	6.51E-12
SPATS1	-2.3003	-2.8916	1.27E-12	6.52E-12
CACNA1I	2.050683	0.966558	1.29E-12	6.63E-12
COL11A2	2.737963	2.391907	1.30E-12	6.66E-12
ITIH6	3.064802	-1.33234	1.30E-12	6.66E-12
DMRTC2	5.452978	-1.23663	1.32E-12	6.75E-12
IGHV7-27	2.431072	-1.64774	1.32E-12	6.76E-12
FSTL4	2.540262	4.24152	1.33E-12	6.82E-12
HNRNPA1P27	2.115465	1.443766	1.34E-12	6.86E-12
WDR87	4.088229	0.114475	1.35E-12	6.88E-12

For Peer Review

TFF3	2.998447	6.947299	1.39E-12	7.10E-12
LUARIS	2.204294	-2.93175	1.39E-12	7.11E-12
CLRN3	2.910552	-0.32599	1.42E-12	7.26E-12
AC092535.1	2.543852	-1.47997	1.43E-12	7.28E-12
INSYN2	2.16643	0.863004	1.43E-12	7.30E-12
C6orf118	-2.00448	0.881194	1.46E-12	7.41E-12
ERN2	3.073801	4.588857	1.46E-12	7.42E-12
LIN28A	6.3178	0.032541	1.46E-12	7.42E-12
SLC38A8	4.239482	-0.60339	1.53E-12	7.79E-12
LINC02257	2.131131	1.347196	1.55E-12	7.89E-12
AC027281.2	2.114822	-3.08688	1.55E-12	7.89E-12
ARSE	2.018995	4.843596	1.56E-12	7.94E-12
KCNA7	2.519976	-2.48712	1.56E-12	7.94E-12
ZNF92P3	2.086236	-2.50852	1.58E-12	8.01E-12
AC020891.3	3.332918	-4.05156	1.58E-12	8.02E-12
AHSG	3.91401	-2.3501	1.58E-12	8.04E-12
IGKV1D-37	3.207736	-3.58453	1.59E-12	8.09E-12
MIR3189	2.57688	-1.33743	1.65E-12	8.34E-12
DSG4	4.645703	-2.55968	1.71E-12	8.64E-12
IGHV1-12	2.282857	0.110896	1.71E-12	8.65E-12
IGHV3-75	2.41892	-1.85609	1.75E-12	8.83E-12
HIST1H2BB	6.11675	-1.09196	1.77E-12	8.96E-12
AC132186.1	3.127277	-3.73002	1.81E-12	9.12E-12
CGB5	7.383666	0.71458	1.87E-12	9.45E-12
AP002754.1	2.351132	-1.24564	1.92E-12	9.69E-12
POU6F2-AS1	4.39925	-3.33718	1.93E-12	9.72E-12
AC106799.2	5.086857	-2.54179	2.03E-12	1.02E-11
RLN3	2.529921	-1.59089	2.07E-12	1.04E-11
LINC01269	2.160147	-0.96585	2.11E-12	1.06E-11
TAC3	5.02707	0.098883	2.24E-12	1.12E-11
ADAMTS20	4.500925	-1.28069	2.26E-12	1.13E-11
GFRA3	2.933189	4.485999	2.28E-12	1.14E-11
AL033397.1	5.595406	2.71082	2.32E-12	1.16E-11
MIR548XHG	6.334203	-1.68941	2.33E-12	1.16E-11
AC245100.6	2.996624	-3.33492	2.36E-12	1.18E-11
AL049830.3	2.419684	-2.10266	2.39E-12	1.20E-11
AL022341.2	2.179865	-2.0403	2.40E-12	1.20E-11
AL022313.2	2.866339	-1.5631	2.41E-12	1.20E-11
SLCO1B7	5.502961	-2.84281	2.44E-12	1.22E-11
AC136475.3	2.096276	3.546971	2.47E-12	1.23E-11
SLC18A3	6.370614	-1.20929	2.47E-12	1.23E-11
AC134879.2	2.342428	-0.09837	2.47E-12	1.23E-11
LINC00824	4.305338	-0.44547	2.49E-12	1.24E-11

For Peer Review

AC106772.1	2.833455	-3.93855	2.50E-12	1.25E-11
SLC1A7	2.982818	4.921233	2.53E-12	1.26E-11
IGHV4OR15-8	2.408885	-2.41081	2.54E-12	1.26E-11
UGT1A10	7.559773	0.906933	2.56E-12	1.27E-11
AC018629.1	2.761535	0.722934	2.56E-12	1.28E-11
SMPX	2.698118	-1.34319	2.57E-12	1.28E-11
AC131206.1	2.950339	-0.06776	2.68E-12	1.33E-11
IGHD3-22	2.898979	-3.4459	2.73E-12	1.35E-11
TM4SF20	6.294018	1.445015	2.75E-12	1.36E-11
CAPN14	2.101976	0.079932	2.75E-12	1.36E-11
TRPA1	2.464698	0.075677	2.79E-12	1.38E-11
IGLV7-43	2.079487	3.443265	2.80E-12	1.39E-11
IGKV1D-12	2.646073	0.254138	2.92E-12	1.44E-11
AL031602.1	2.046845	-2.11262	2.92E-12	1.45E-11
B4GALNT2	3.626806	3.069929	2.95E-12	1.46E-11
AC110619.1	2.000871	0.780624	3.00E-12	1.48E-11
AC106799.3	5.132141	-1.63151	3.01E-12	1.49E-11
AC005726.2	2.102393	-2.45857	3.02E-12	1.50E-11
AC093833.1	3.512229	-3.98058	3.05E-12	1.51E-11
AC008406.3	2.885587	-3.58784	3.06E-12	1.51E-11
AC093515.1	4.360828	-2.7621	3.06E-12	1.51E-11
SMIM31	2.65959	2.332683	3.13E-12	1.55E-11
HOXD11	5.770999	-1.58939	3.14E-12	1.55E-11
AC079466.1	7.023105	-0.56393	3.18E-12	1.57E-11
KRT17	2.714426	6.92715	3.26E-12	1.60E-11
LINC01804	4.918597	-1.81266	3.29E-12	1.62E-11
TNNI3	2.782623	1.680053	3.29E-12	1.62E-11
AC023421.1	4.847767	2.723111	3.30E-12	1.63E-11
SNORA74B	5.054904	-0.42446	3.31E-12	1.63E-11
AL138720.1	2.09316	-2.69404	3.31E-12	1.63E-11
AP003031.1	2.464572	-3.50433	3.31E-12	1.63E-11
IGKV1OR10-1	2.260393	-2.23573	3.44E-12	1.69E-11
AC008063.1	2.120357	-0.9611	3.44E-12	1.69E-11
LINC00251	4.199844	-3.66035	3.45E-12	1.70E-11
AC244250.1	3.140766	-3.90488	3.50E-12	1.72E-11
SP9	5.886645	-1.82777	3.50E-12	1.72E-11
NR5A1	3.316878	-1.9638	3.51E-12	1.72E-11
CNTNAP2	2.885357	2.874305	3.61E-12	1.77E-11
UGT1A6	4.571064	3.222553	3.63E-12	1.78E-11
CA6	4.216299	-0.61933	3.67E-12	1.80E-11
KIAA1549L	2.148182	1.523626	3.81E-12	1.86E-11
IGFL3	4.038062	-2.63864	3.85E-12	1.88E-11
NGB	5.546466	-1.8425	3.87E-12	1.89E-11

For Peer Review

AKR1B10P1	5.743744	-1.45339	3.90E-12	1.91E-11
ASNSP3	2.412086	-3.39525	3.92E-12	1.91E-11
IGKV2-30	2.104488	2.110343	4.05E-12	1.98E-11
FLJ22447	2.744767	1.74091	4.07E-12	1.98E-11
FAM83F	2.406551	3.380781	4.08E-12	1.99E-11
FOXL2NB	4.305696	-2.57095	4.12E-12	2.01E-11
LINC02466	5.202432	-3.05136	4.16E-12	2.03E-11
OR10H1	3.963141	-3.16231	4.30E-12	2.09E-11
AC098936.1	2.137629	-3.01196	4.38E-12	2.13E-11
NPTX2	2.496578	2.920937	4.38E-12	2.13E-11
LINC01687	4.519668	-3.47404	4.39E-12	2.14E-11
NPBWR1	3.3472	-0.6099	4.42E-12	2.15E-11
MYPN	3.795772	-0.96172	4.43E-12	2.15E-11
IGLV2-5	2.209325	-1.29053	4.43E-12	2.16E-11
ARL2BPP4	2.657807	-2.30261	4.55E-12	2.21E-11
COL9A1	2.324511	-1.29856	4.59E-12	2.23E-11
LINC01980	6.151666	-0.40778	4.62E-12	2.24E-11
CPN1	7.722413	-0.80052	4.64E-12	2.25E-11
AC092535.2	2.033486	-1.57284	4.67E-12	2.26E-11
CCDC162P	2.050201	1.223277	4.69E-12	2.27E-11
HOXA10-AS	4.168418	-2.84378	4.71E-12	2.28E-11
CST2	2.255848	1.310537	4.76E-12	2.31E-11
ADGRA1	-2.03706	-1.53649	4.77E-12	2.31E-11
MYCNOS	3.667433	-2.18344	4.79E-12	2.32E-11
UGT2B28	4.947558	-2.54217	4.81E-12	2.33E-11
AC100826.1	5.124856	-0.42199	4.83E-12	2.34E-11
AC069277.1	4.159299	-1.70334	4.91E-12	2.38E-11
DCAF12L2	3.27165	-1.83655	4.93E-12	2.39E-11
MIR2052HG	3.650918	-1.72795	4.94E-12	2.39E-11
OR13K1P	3.436535	-3.41676	4.95E-12	2.39E-11
MIR31HG	3.312549	-1.21729	4.99E-12	2.41E-11
HMGB1P11	2.042342	-3.5742	5.00E-12	2.42E-11
HOXA11	4.194424	-0.77487	5.00E-12	2.42E-11
LHX1-DT	5.57868	-1.84833	5.01E-12	2.42E-11
AL139095.3	3.967952	-3.15195	5.13E-12	2.47E-11
AL136537.2	5.967338	-1.46945	5.14E-12	2.48E-11
IGHV3-25	2.306673	-1.73806	5.22E-12	2.52E-11
RN7SL12P	3.103478	-3.95932	5.25E-12	2.53E-11
AL606970.1	4.347484	-1.54462	5.25E-12	2.53E-11
DISP3	2.203585	-0.20419	5.31E-12	2.56E-11
MRPL45P1	4.692138	-3.37434	5.32E-12	2.57E-11
AC005996.1	3.168409	-2.7828	5.38E-12	2.59E-11
AC011632.1	4.891414	-0.87011	5.40E-12	2.60E-11

For Peer Review

ST7-OT4	2.647033	-1.08518	5.41E-12	2.60E-11
CHODL	2.030615	1.291291	5.45E-12	2.62E-11
NDP	3.206794	0.48249	5.51E-12	2.65E-11
ISM2	3.226884	-1.4503	5.59E-12	2.69E-11
AC087379.1	3.835898	-2.58961	5.66E-12	2.72E-11
AKR1C1	3.242572	8.146617	5.68E-12	2.73E-11
FLJ16779	2.625608	-0.7482	5.71E-12	2.74E-11
RN7SL2	3.247718	9.0867	5.77E-12	2.77E-11
NECAB2	2.589783	0.065684	5.86E-12	2.81E-11
AC023090.1	2.639684	-2.08689	5.92E-12	2.84E-11
AC010735.1	2.775869	-2.38962	5.97E-12	2.87E-11
AP000525.1	2.036422	-1.90923	6.08E-12	2.91E-11
AP000859.1	4.141497	-3.69226	6.12E-12	2.93E-11
GALR2	2.207615	-1.35827	6.23E-12	2.98E-11
C1QL4	3.241964	-2.23149	6.30E-12	3.01E-11
PCSK1N	2.29085	3.426777	6.35E-12	3.04E-11
AL354861.2	2.431439	-3.55202	6.40E-12	3.06E-11
LINC02178	5.727718	-2.49985	6.40E-12	3.06E-11
IGLJ1	2.319353	-2.78023	6.69E-12	3.19E-11
TMEM74	2.28962	-0.84008	6.80E-12	3.24E-11
PURPL	4.038537	-0.70352	6.86E-12	3.26E-11
GDA	3.470745	1.901802	7.00E-12	3.33E-11
DIRC1	2.637525	-3.58334	7.08E-12	3.37E-11
PSG1	8.086185	-0.46036	7.12E-12	3.38E-11
MAGEA11	7.076327	-0.709	7.18E-12	3.41E-11
C8orf74	4.580856	-1.87375	7.19E-12	3.42E-11
AC003092.1	5.202551	-0.91675	7.20E-12	3.42E-11
LINC01194	6.110038	-0.62956	7.23E-12	3.43E-11
IFNWP19	3.405964	-1.49285	7.27E-12	3.45E-11
AC138305.1	3.444981	-2.66646	7.42E-12	3.52E-11
TAF1L	2.765993	-2.2383	7.48E-12	3.55E-11
FZD9	2.545108	1.023352	7.50E-12	3.56E-11
LINC00513	2.17278	0.44091	7.65E-12	3.62E-11
AC245100.3	3.351257	-3.16057	7.69E-12	3.64E-11
LINC00536	3.372335	-3.85585	7.78E-12	3.68E-11
TMPRSS11A	3.984477	-1.95796	7.90E-12	3.74E-11
REG1A	7.587797	2.892011	7.90E-12	3.74E-11
ATOH7	2.101613	-2.43966	7.97E-12	3.77E-11
IGLV2-18	2.144799	2.656429	7.97E-12	3.77E-11
ISL1	4.188732	0.016969	8.05E-12	3.80E-11
BX005040.1	-2.24864	-3.23228	8.16E-12	3.85E-11
RPSAP58	2.820025	-1.4144	8.31E-12	3.92E-11
IGFL1P2	5.05528	-3.04251	8.42E-12	3.97E-11

For Peer Review

LGALS4	3.886126	5.77413	8.67E-12	4.09E-11
AL355076.3	2.041092	-2.95513	8.77E-12	4.13E-11
AC097512.1	5.08526	-2.98366	9.01E-12	4.24E-11
RN7SKP97	2.020678	-1.79433	9.08E-12	4.27E-11
AC008870.5	3.405847	-1.28328	9.26E-12	4.35E-11
AC092828.1	2.418427	-0.08905	9.27E-12	4.36E-11
AP003472.1	2.297784	-2.68359	9.43E-12	4.43E-11
OBP2A	4.270177	0.049012	9.43E-12	4.43E-11
IGKV1OR1-1	3.033608	-2.6617	9.51E-12	4.46E-11
LINC02109	4.513543	-2.41697	9.55E-12	4.48E-11
ERVK-28	3.214583	-3.53359	9.56E-12	4.48E-11
AC007686.1	3.033751	-2.40967	9.91E-12	4.65E-11
MAGEA9B	6.989221	-1.63284	9.95E-12	4.66E-11
NR1I2	2.646995	0.370849	9.96E-12	4.66E-11
AC098798.1	4.528665	-2.92442	9.97E-12	4.67E-11
AC114296.1	3.031013	-2.94642	1.01E-11	4.74E-11
DHRS2	3.262281	3.09099	1.02E-11	4.78E-11
HOXA1	2.030019	0.933769	1.02E-11	4.79E-11
INSM1	4.399367	2.115142	1.03E-11	4.82E-11
LINC01299	5.346421	-2.37852	1.05E-11	4.92E-11
MIR34C	-2.14867	-2.68292	1.05E-11	4.93E-11
AC069499.2	2.509701	-3.74811	1.06E-11	4.93E-11
FOXL2	4.375075	-1.93341	1.06E-11	4.94E-11
IGKV3OR2-5	3.697974	-2.84291	1.06E-11	4.97E-11
NSG2	4.637573	1.378127	1.07E-11	4.99E-11
AP000424.2	2.310674	-3.30058	1.07E-11	5.00E-11
MCF2	2.52375	-0.98092	1.10E-11	5.11E-11
HOXB-AS4	4.148684	-2.31515	1.10E-11	5.11E-11
DSCAM-AS1	7.742351	3.783766	1.10E-11	5.11E-11
AC025252.2	5.383395	-2.92652	1.11E-11	5.15E-11
MAGEA10-MAGEA5	3.288881	-3.13592	1.12E-11	5.20E-11
RNU6-548P	2.183337	-3.5477	1.14E-11	5.30E-11
RUNDC3A	2.184036	1.413732	1.15E-11	5.34E-11
NCAN	2.459742	-3.1223	1.15E-11	5.36E-11
KRTAP2-3	3.906103	-2.63881	1.18E-11	5.46E-11
AC091133.4	3.805955	-3.24914	1.18E-11	5.50E-11
LINC02293	4.475656	-2.27278	1.19E-11	5.54E-11
C4BPB	2.186185	3.36997	1.23E-11	5.72E-11
AL356274.1	4.367973	-3.41645	1.23E-11	5.72E-11
AC021594.1	2.914205	-3.3508	1.24E-11	5.74E-11
RF02246	3.587336	-3.57994	1.24E-11	5.76E-11
LINC00898	4.589955	-2.7854	1.26E-11	5.82E-11

For Peer Review

MSLNL	3.003173	1.763498	1.33E-11	6.13E-11
AC005291.2	3.100678	-2.37391	1.34E-11	6.22E-11
AC098826.2	4.695702	-3.12235	1.35E-11	6.26E-11
ALKAL1	2.02287	0.601777	1.37E-11	6.34E-11
AC010947.1	3.385407	-3.94295	1.37E-11	6.34E-11
TUBB4A	2.309361	1.666524	1.39E-11	6.43E-11
FBXO47	2.539794	-3.2713	1.41E-11	6.49E-11
C20orf141	4.469514	-3.39539	1.41E-11	6.49E-11
AP003174.1	3.322515	-3.58971	1.42E-11	6.54E-11
SNORA80E	2.693972	-2.54717	1.42E-11	6.54E-11
GIP	4.229184	-2.74554	1.45E-11	6.68E-11
AL353770.4	-2.17872	-2.67317	1.46E-11	6.70E-11
MAGEC3	3.853984	-1.45892	1.47E-11	6.77E-11
Z99943.1	2.292544	-2.29593	1.49E-11	6.84E-11
AC137834.2	2.238608	-3.6463	1.49E-11	6.84E-11
AL162411.1	2.45214	-1.93324	1.50E-11	6.89E-11
PHOX2B	6.37686	-1.37141	1.50E-11	6.92E-11
RNU6-1042P	3.293967	-3.67886	1.51E-11	6.93E-11
AP005328.1	3.496295	-2.55189	1.52E-11	7.00E-11
C14orf39	2.487156	-1.36184	1.53E-11	7.04E-11
AKR1D1	2.522661	-2.71214	1.56E-11	7.17E-11
RNU4-52P	2.1782	-3.50242	1.57E-11	7.20E-11
IGLV5-48	2.465118	-0.70945	1.59E-11	7.28E-11
AC104852.1	3.032438	-3.95276	1.59E-11	7.29E-11
AC114316.2	3.468402	-1.83616	1.59E-11	7.29E-11
AL121904.1	3.579824	-1.61066	1.60E-11	7.34E-11
BPIFB2	4.724747	4.611893	1.64E-11	7.49E-11
LINC02492	5.296749	-2.70275	1.64E-11	7.49E-11
IGKV1OR-2	2.312659	-3.47786	1.64E-11	7.51E-11
PRSS56	5.622361	-1.59256	1.65E-11	7.54E-11
H19	3.373363	6.743353	1.66E-11	7.59E-11
AC007848.1	3.590485	-1.6158	1.66E-11	7.60E-11
LINC02253	4.984988	-0.77621	1.67E-11	7.61E-11
COX7B2	7.521141	-0.48228	1.71E-11	7.79E-11
BNIP3P42	3.528574	-2.99525	1.78E-11	8.09E-11
LINC02525	5.621479	-2.23264	1.79E-11	8.15E-11
IGLV4-60	2.522117	2.740982	1.81E-11	8.26E-11
ASCL1	5.025893	5.01278	1.82E-11	8.29E-11
AC007546.2	2.665962	-3.67476	1.84E-11	8.38E-11
RN7SL5P	2.925959	0.249225	1.84E-11	8.39E-11
DSCR8	6.493795	-0.66583	1.87E-11	8.50E-11
CRHR1	3.648335	-1.57408	1.87E-11	8.51E-11
LHX8	5.335199	-1.53373	1.88E-11	8.55E-11

For Peer Review

EIF4E1B	5.982595	-0.83186	1.88E-11	8.55E-11
LINC00659	3.691114	-2.51931	1.88E-11	8.57E-11
AC005336.1	3.509732	0.326847	1.89E-11	8.57E-11
AC010735.2	2.000111	-0.82906	1.89E-11	8.60E-11
THAP12P3	2.575803	-3.80673	1.90E-11	8.63E-11
ODAPH	2.559989	-1.56359	1.91E-11	8.69E-11
IGLV9-49	2.80089	3.659304	1.95E-11	8.84E-11
AP001429.1	2.522347	-1.88997	1.96E-11	8.91E-11
VAX1	5.728028	-2.10311	1.97E-11	8.93E-11
BCAS1	2.259285	4.970241	1.98E-11	8.98E-11
PGGT1BP2	2.868149	-3.11295	2.00E-11	9.07E-11
AC007221.1	-2.03806	-3.85846	2.01E-11	9.09E-11
AC007405.2	2.802402	-3.41942	2.03E-11	9.18E-11
PIWIL3	6.014437	-0.32366	2.03E-11	9.18E-11
ZNF648	2.805771	-2.53788	2.04E-11	9.21E-11
RNY1	7.41923	1.563069	2.06E-11	9.29E-11
RN7SL381P	2.09627	-0.63747	2.10E-11	9.48E-11
MIR181A1HG	3.122543	-0.96341	2.14E-11	9.66E-11
AL359313.1	3.312161	-3.03296	2.14E-11	9.67E-11
AC073316.1	4.30905	-3.12101	2.16E-11	9.72E-11
AL365226.2	3.219404	2.283724	2.16E-11	9.75E-11
RHCG	3.597056	1.57079	2.16E-11	9.76E-11
HIF1A-AS2	2.137929	-0.46073	2.18E-11	9.84E-11
AC109454.3	3.685781	-2.43885	2.26E-11	1.02E-10
HOXD13	6.581396	-0.30684	2.27E-11	1.02E-10
AL032819.2	3.087717	1.347921	2.27E-11	1.02E-10
KLK8	4.900011	1.595898	2.30E-11	1.03E-10
DSG3	4.636279	1.84878	2.31E-11	1.04E-10
KCNV1	3.622876	-0.77893	2.35E-11	1.05E-10
LINC02048	3.974421	-2.75977	2.36E-11	1.06E-10
AC117386.1	4.706699	-3.08897	2.46E-11	1.11E-10
SLC5A12	2.878267	0.200225	2.50E-11	1.12E-10
SCARNA7	4.396351	1.811117	2.52E-11	1.13E-10
SAMSN1-AS1	3.165538	-2.57332	2.53E-11	1.13E-10
SPDYC	4.222528	-0.52322	2.54E-11	1.14E-10
AL023755.1	3.558513	-3.68296	2.55E-11	1.14E-10
OTOG	3.661882	-2.79156	2.55E-11	1.14E-10
HIST2H3D	2.569963	-1.72748	2.59E-11	1.16E-10
AC117402.1	2.82732	-0.19205	2.59E-11	1.16E-10
MAGEA1	7.212233	1.631924	2.60E-11	1.16E-10
HOXD12	4.025926	-3.64375	2.64E-11	1.18E-10
TDRD1	2.786582	1.22718	2.66E-11	1.19E-10
AC073578.1	4.171643	-3.04444	2.67E-11	1.19E-10

For Peer Review

AC008163.1	5.062511	0.192998	2.71E-11	1.21E-10
LINC02331	2.78848	-2.24873	2.76E-11	1.23E-10
MMP7	2.02052	6.654585	2.77E-11	1.23E-10
RGS7	2.933015	0.337462	2.77E-11	1.23E-10
RANBP20P	3.968494	-0.85629	2.77E-11	1.24E-10
NR0B1	6.305241	2.357077	2.78E-11	1.24E-10
AC246787.2	2.063056	-2.44389	2.80E-11	1.25E-10
RN7SL521P	2.091272	-2.26852	2.82E-11	1.26E-10
AC022240.1	2.89255	-3.32687	2.83E-11	1.26E-10
AC079384.1	3.677415	-0.42032	2.86E-11	1.27E-10
AL611929.1	4.532314	-2.66152	2.91E-11	1.29E-10
AL023775.1	2.750177	-3.73873	2.92E-11	1.30E-10
ENPP3	2.152136	3.302706	2.92E-11	1.30E-10
AC131971.1	2.707882	-0.86783	2.92E-11	1.30E-10
LHFPL4	3.46556	1.41123	2.93E-11	1.30E-10
OR7E62P	3.44984	-3.49282	2.94E-11	1.31E-10
ADAM2	4.945244	-2.03511	2.95E-11	1.31E-10
TMEM78	2.370259	-3.91702	3.01E-11	1.34E-10
EPPIN-WFDC6	-2.00883	-3.80657	3.11E-11	1.38E-10
CHRNB2	2.901979	0.506422	3.16E-11	1.40E-10
AC093520.1	2.100482	-2.72264	3.19E-11	1.41E-10
TMPRSS6	2.313781	3.697167	3.23E-11	1.43E-10
AL589182.1	4.949857	-3.22677	3.26E-11	1.44E-10
IGHV3-50	2.698307	-3.00923	3.36E-11	1.48E-10
AL590491.2	-2.22021	-2.8977	3.42E-11	1.51E-10
SIM1	4.219427	-1.42436	3.42E-11	1.51E-10
SCG2	3.168481	4.379091	3.48E-11	1.53E-10
AC239584.1	3.800705	-2.43175	3.48E-11	1.53E-10
AL513548.1	5.111093	-1.04081	3.51E-11	1.55E-10
AC012512.1	3.971567	-0.71258	3.63E-11	1.60E-10
GAPDHP22	2.04316	-3.74449	3.67E-11	1.61E-10
LINC02223	3.574796	-3.00304	3.69E-11	1.62E-10
SNORA2C	3.129373	-1.4164	3.76E-11	1.65E-10
OR4C6	4.235804	-2.71154	3.77E-11	1.65E-10
PDCL2	4.865005	-2.34365	3.79E-11	1.66E-10
AC022165.1	2.040135	-3.26774	3.82E-11	1.68E-10
MSI1	2.288864	2.046257	3.84E-11	1.68E-10
AC125603.1	4.495763	-0.49699	3.86E-11	1.69E-10
PROKR1	2.968923	-1.53267	3.88E-11	1.70E-10
IGKV2OR22-3	2.517653	-1.22904	3.88E-11	1.70E-10
RNA5SP323	2.856844	-3.99193	3.89E-11	1.70E-10
LGALS7B	3.139987	0.177928	3.94E-11	1.73E-10
OFCC1	4.420366	-2.18972	3.95E-11	1.73E-10

For Peer Review

AC126323.6	2.877125	-1.77464	4.00E-11	1.75E-10
DPYSL5	5.640485	0.416192	4.03E-11	1.76E-10
AC021683.2	3.193919	-0.09306	4.08E-11	1.79E-10
AL353572.1	2.428246	-2.8433	4.15E-11	1.81E-10
LINC01344	2.474231	-2.73054	4.26E-11	1.86E-10
IGKV1OR2-118	2.259055	-2.88389	4.29E-11	1.87E-10
GJA3	2.458197	-0.73557	4.40E-11	1.92E-10
LINC01629	3.292697	-2.0538	4.47E-11	1.95E-10
TBC1D3P1-DHX40P1	4.718906	-3.35031	4.57E-11	1.99E-10
IGHV3OR16-12	2.110089	-1.82564	4.58E-11	1.99E-10
DLK1	7.050169	3.780225	4.65E-11	2.02E-10
MAEL	3.6663	1.578051	4.67E-11	2.03E-10
AL133320.1	-2.11091	-1.10009	4.68E-11	2.04E-10
PRB4	8.081284	-0.17421	4.70E-11	2.04E-10
AC008132.1	2.148306	-2.81512	4.70E-11	2.05E-10
LINC01224	2.525088	0.756186	4.71E-11	2.05E-10
Z85994.1	2.999093	-3.27018	4.72E-11	2.05E-10
SNORA5A	2.052353	-2.09435	4.73E-11	2.05E-10
PSG9	5.649549	-2.48912	4.77E-11	2.07E-10
IGLJCOR18	3.287853	-3.36942	4.83E-11	2.10E-10
AC103705.1	4.28756	-3.28746	4.85E-11	2.10E-10
AC099329.2	2.463427	0.463661	4.86E-11	2.11E-10
TMEM229A	4.462866	0.772705	4.96E-11	2.15E-10
PLUT	4.88904	-2.93689	5.03E-11	2.18E-10
AC135068.9	2.703189	-3.34131	5.13E-11	2.22E-10
SLCO6A1	4.395596	-2.20294	5.30E-11	2.29E-10
AC011700.1	3.220837	-2.43092	5.31E-11	2.30E-10
IGHV2OR16-5	2.943008	-3.28544	5.31E-11	2.30E-10
IGHV3-22	2.152774	-1.50943	5.32E-11	2.30E-10
AC090578.2	3.270125	-3.02696	5.42E-11	2.34E-10
AL592148.2	3.133384	-3.53297	5.47E-11	2.36E-10
SOX21	2.661223	2.11601	5.64E-11	2.44E-10
LINC01297	4.171149	-3.2002	5.65E-11	2.44E-10
C17orf78	2.143158	-2.52799	5.70E-11	2.46E-10
SPATA3-AS1	2.260486	-2.94185	5.72E-11	2.47E-10
AC008147.4	2.220818	-3.08277	5.79E-11	2.50E-10
UGT1A8	6.857941	-1.12592	5.92E-11	2.55E-10
S100A7	5.384717	1.744408	5.94E-11	2.56E-10
AVPR1B	2.924435	-2.68825	6.15E-11	2.65E-10
AC138409.1	2.550599	-0.67798	6.22E-11	2.68E-10
DEPDC1-AS1	2.126856	-3.59638	6.26E-11	2.69E-10
AL034345.2	2.751103	-3.48253	6.29E-11	2.70E-10

For Peer Review

ZBTB20-AS1	2.633211	-1.64372	6.31E-11	2.71E-10
PSG3	7.385029	0.03031	6.39E-11	2.75E-10
TTTY16	-2.29499	-3.92612	6.41E-11	2.75E-10
NIFKP3	3.010022	-3.53018	6.72E-11	2.88E-10
TMEM179	3.950505	-0.19339	6.72E-11	2.88E-10
BX276092.7	5.201563	-2.93687	6.82E-11	2.92E-10
RBM46	4.432131	-0.90877	6.88E-11	2.94E-10
AC084026.2	2.634538	-2.4246	6.97E-11	2.98E-10
CFHR4	3.845414	-3.32791	6.99E-11	2.99E-10
OTX2	6.362761	-1.39654	7.06E-11	3.02E-10
RNASE10	2.401803	-2.60845	7.22E-11	3.08E-10
GABRA5	4.53798	-2.34146	7.32E-11	3.13E-10
AC073358.1	3.276795	-3.44728	7.37E-11	3.14E-10
C1QL1	2.2307	-0.63722	7.41E-11	3.16E-10
IGFBP1	4.266228	2.081553	7.64E-11	3.26E-10
TRPM5	2.630942	-0.39656	7.72E-11	3.29E-10
AL109924.2	2.339152	-3.7032	7.85E-11	3.34E-10
BPIFA4P	4.699567	-2.30189	7.96E-11	3.38E-10
BMS1P22	2.689224	-3.14626	7.97E-11	3.39E-10
AC108025.2	4.148381	-3.4296	8.01E-11	3.41E-10
FGA	3.87768	8.517199	8.04E-11	3.42E-10
ARNTL2-AS1	2.986849	-3.31539	8.05E-11	3.42E-10
AC016831.4	3.536304	-0.1033	8.13E-11	3.46E-10
AL596218.1	2.832808	-1.05228	8.16E-11	3.47E-10
AC022028.2	2.544581	-2.70689	8.17E-11	3.47E-10
MIR194-2HG	2.911983	-1.56187	8.26E-11	3.51E-10
IGHV3OR16-7	2.548923	-2.86737	8.39E-11	3.56E-10
AL451054.1	2.537919	-2.9655	8.41E-11	3.57E-10
GAP43	2.286001	-0.36416	8.41E-11	3.57E-10
RN7SL396P	3.1683	-0.51437	8.42E-11	3.57E-10
IGHV3OR16-16	2.467107	-2.44865	8.48E-11	3.60E-10
RN7SL394P	3.928974	-3.67401	8.66E-11	3.67E-10
LINC00184	2.026809	-1.83499	8.67E-11	3.67E-10
CLDN2	3.037205	5.693136	8.77E-11	3.71E-10
PAGE2	7.661462	1.479015	8.95E-11	3.78E-10
AC068722.1	2.972516	-3.6233	8.95E-11	3.78E-10
IGLV3-27	2.334644	3.80334	9.01E-11	3.81E-10
IGHV3-6	2.281084	-2.15793	9.04E-11	3.82E-10
AP003721.1	2.058595	-3.38412	9.06E-11	3.83E-10
BHMT	3.59311	0.879601	9.10E-11	3.84E-10
INHBE	2.053646	-0.16647	9.10E-11	3.84E-10
LRRC66	2.352076	0.426965	9.20E-11	3.88E-10
SNORA54	6.092287	0.271955	9.27E-11	3.91E-10

For Peer Review

CASC19	4.306037	-1.89647	9.29E-11	3.92E-10
KRT6B	3.980027	2.279214	9.31E-11	3.93E-10
AC090502.3	3.702157	-3.67537	9.40E-11	3.96E-10
LINC00524	3.360099	-2.09717	9.51E-11	4.00E-10
AC069120.1	3.601462	-2.45787	9.68E-11	4.07E-10
AC093904.3	3.217472	-3.59491	9.69E-11	4.08E-10
AP000542.2	5.32688	-2.73446	9.75E-11	4.10E-10
SLC35F4	2.983783	-2.49315	9.76E-11	4.10E-10
DPYD-AS1	3.168172	0.381779	9.81E-11	4.13E-10
IGHVIII-51-1	2.349709	-3.01662	9.82E-11	4.13E-10
AC099520.1	4.319617	-3.48145	9.82E-11	4.13E-10
CHL1-AS1	2.515618	-2.83026	9.91E-11	4.17E-10
AL133373.1	2.604052	-3.82238	1.00E-10	4.20E-10
PCDH8	5.33611	0.326204	1.01E-10	4.25E-10
ASIC5	2.91768	-3.31664	1.02E-10	4.26E-10
AP003119.1	3.405428	-1.41048	1.02E-10	4.27E-10
AL390115.1	2.085756	-3.64952	1.02E-10	4.30E-10
CYP4F11	2.939681	3.682542	1.04E-10	4.35E-10
DKK4	5.865998	0.769991	1.04E-10	4.35E-10
TBL1XR1-AS1	3.420041	-2.37061	1.06E-10	4.42E-10
HEPACAM2	3.659243	1.953008	1.06E-10	4.45E-10
CDH18	4.836466	-0.15111	1.08E-10	4.50E-10
TMEFF1	2.854097	-2.88447	1.08E-10	4.52E-10
ZPBP2	4.086188	-2.03659	1.09E-10	4.56E-10
SNORA80A	5.804423	-2.44697	1.10E-10	4.59E-10
AC022748.2	2.676134	-3.7698	1.10E-10	4.60E-10
IGKV1OR-3	2.478013	-2.99937	1.10E-10	4.61E-10
GC	8.137207	1.15208	1.11E-10	4.62E-10
AC025434.1	3.052047	-3.75305	1.11E-10	4.63E-10
AC131097.3	2.130865	-0.82895	1.13E-10	4.70E-10
CD177	3.216526	3.693734	1.13E-10	4.70E-10
SNX18P2	3.329085	-3.38216	1.13E-10	4.73E-10
CAGE1	2.143987	-2.36528	1.14E-10	4.75E-10
MAGEA4	8.714772	3.587879	1.14E-10	4.76E-10
GABRP	3.143337	3.984832	1.15E-10	4.77E-10
GDPD2	2.468222	-0.84965	1.15E-10	4.80E-10
MYT1	2.954657	1.208288	1.16E-10	4.82E-10
LINC01583	2.183509	-3.02028	1.16E-10	4.85E-10
NDUFA5P6	4.875192	-2.74147	1.17E-10	4.88E-10
GPR89P	2.860791	-2.29624	1.18E-10	4.89E-10
MSTN	3.693658	1.338187	1.19E-10	4.96E-10
LINC01967	4.218569	-3.11792	1.20E-10	4.98E-10
ATP4B	2.099007	-1.44249	1.20E-10	4.99E-10

For Peer Review

IGHV6-1	2.095002	1.609496	1.22E-10	5.05E-10
AC145146.1	2.695666	-2.96175	1.27E-10	5.26E-10
IGLV3-6	2.792006	-2.64292	1.27E-10	5.27E-10
AL390729.1	2.581352	-2.38186	1.28E-10	5.32E-10
DCAF4L2	7.733216	-0.51807	1.32E-10	5.46E-10
RN7SL674P	3.119479	-0.25904	1.33E-10	5.50E-10
PRSS29P	5.000814	-0.47061	1.36E-10	5.62E-10
KBTBD12	2.025031	1.964039	1.36E-10	5.63E-10
AC072062.1	2.221048	-2.79426	1.39E-10	5.76E-10
IGLV5-37	2.834374	1.066093	1.40E-10	5.78E-10
CDKN2A-DT	2.792569	-3.3803	1.42E-10	5.89E-10
AC090709.1	2.974473	-3.03766	1.46E-10	6.03E-10
AC009139.1	3.089486	-3.2781	1.48E-10	6.11E-10
IGHD3-3	2.422157	-2.40948	1.48E-10	6.12E-10
CYP21A2	2.154526	-0.26248	1.50E-10	6.21E-10
PYDC1	4.124932	-2.43773	1.54E-10	6.33E-10
AL355075.4	6.443938	1.655057	1.56E-10	6.43E-10
AC107308.1	4.035545	-3.51393	1.57E-10	6.45E-10
AL359955.1	5.681679	-1.41465	1.60E-10	6.58E-10
SLC30A10	5.051019	-1.03159	1.61E-10	6.61E-10
CHAT	5.413676	-1.11364	1.62E-10	6.65E-10
TCF24	2.362821	-1.18119	1.63E-10	6.69E-10
AC073284.1	2.968401	-3.8236	1.66E-10	6.84E-10
AL445072.1	3.711212	-3.89744	1.67E-10	6.85E-10
LINC00589	2.111372	-1.18241	1.69E-10	6.92E-10
SH2D5	2.316809	-0.72479	1.71E-10	7.02E-10
NKX6-1	2.330229	-1.4514	1.73E-10	7.08E-10
RNU5A-1	7.48069	1.381721	1.74E-10	7.12E-10
KIF25-AS1	3.271009	0.512885	1.78E-10	7.28E-10
AC096537.1	2.319179	-2.21418	1.80E-10	7.35E-10
HABP2	2.20992	4.679258	1.81E-10	7.40E-10
MANCR	3.046424	-1.00822	1.81E-10	7.41E-10
NEFH	2.519089	2.896362	1.82E-10	7.43E-10
POU5F2	3.557963	-0.69576	1.83E-10	7.48E-10
HIST1H4L	6.77489	-1.59407	1.84E-10	7.53E-10
STRA8	4.87094	-1.04502	1.86E-10	7.61E-10
AC034223.2	4.319999	-3.33368	1.86E-10	7.62E-10
SOX14	6.177192	0.687368	1.88E-10	7.68E-10
HIST1H1T	2.473955	-3.41751	1.89E-10	7.73E-10
BOLL	2.70659	-2.73456	1.90E-10	7.74E-10
LNCSRLR	2.039969	-2.03111	1.91E-10	7.79E-10
FGF20	2.444378	-1.65181	1.91E-10	7.80E-10
CHST4	2.269616	1.180318	1.96E-10	8.01E-10

For Peer Review

MRPS31P2	2.579042	-3.66283	1.99E-10	8.11E-10
PRDM9	5.442699	-2.51645	2.02E-10	8.21E-10
LINC02208	2.903921	-3.26228	2.07E-10	8.44E-10
IGLV1-41	2.620538	1.46102	2.11E-10	8.56E-10
WDR72	2.575291	3.779996	2.11E-10	8.57E-10
PRKCA-AS1	4.577945	-1.61496	2.15E-10	8.72E-10
AC109588.1	5.61017	-2.55077	2.15E-10	8.74E-10
SST	7.340967	3.526429	2.17E-10	8.81E-10
LINC01225	2.443343	-1.79804	2.19E-10	8.90E-10
FOXG1	5.084468	-0.94567	2.20E-10	8.93E-10
CYP26A1	2.666454	-0.20519	2.25E-10	9.12E-10
IGHV1-68	2.248637	-2.61335	2.26E-10	9.15E-10
AC011742.3	2.083895	-2.51885	2.26E-10	9.16E-10
LINC02477	2.952857	-3.41834	2.26E-10	9.17E-10
AL136298.1	3.063156	-3.26539	2.28E-10	9.25E-10
LINC01510	3.53318	-2.6326	2.30E-10	9.30E-10
SNORA22	3.007733	-1.09052	2.31E-10	9.33E-10
PSG4	7.55472	1.254928	2.34E-10	9.47E-10
AZGP1P1	4.131653	-1.77906	2.39E-10	9.64E-10
TAS2R38	4.218639	-3.29142	2.40E-10	9.68E-10
AC135782.1	3.009058	-3.1185	2.42E-10	9.77E-10
APOA2	4.493274	-0.22976	2.42E-10	9.79E-10
AC013410.2	2.915807	-3.90297	2.45E-10	9.90E-10
LINC02484	5.848683	-2.56696	2.48E-10	9.99E-10
MYO18B	3.134656	-0.13835	2.48E-10	1.00E-09
LINC02377	5.673857	-2.71032	2.50E-10	1.01E-09
AC009743.1	3.40098	-3.01946	2.52E-10	1.02E-09
VCX	4.25219	-1.53742	2.53E-10	1.02E-09
HNF1A-AS1	2.678946	1.181515	2.56E-10	1.03E-09
MEG3	2.085623	5.242428	2.57E-10	1.03E-09
KRT14	3.965186	3.518103	2.58E-10	1.04E-09
KCNQ5-IT1	3.892842	-2.73734	2.58E-10	1.04E-09
STPG4	2.224336	-1.50477	2.63E-10	1.06E-09
DLX2	3.185489	-2.70396	2.63E-10	1.06E-09
MTRF1LP2	2.928806	-3.60578	2.65E-10	1.06E-09
AC112204.3	-2.05045	-3.76225	2.66E-10	1.07E-09
AP001619.2	2.034142	-3.70876	2.68E-10	1.07E-09
SLC2A2	6.23515	-0.86648	2.70E-10	1.08E-09
AC010307.4	2.834162	-2.69772	2.72E-10	1.09E-09
LINC01429	2.807682	-3.54497	2.74E-10	1.10E-09
C5orf66-AS1	3.878843	-2.59784	2.76E-10	1.11E-09
DDX53	5.088281	-1.79366	2.81E-10	1.13E-09
LINC00648	3.952532	0.414075	2.81E-10	1.13E-09

For Peer Review

AC091179.1	4.968968	-2.62066	2.91E-10	1.16E-09
SERPINA5	2.37023	2.019041	2.92E-10	1.17E-09
ERVMER61-1	5.510789	-2.60872	2.94E-10	1.18E-09
MUC17	7.656465	1.344953	2.96E-10	1.18E-09
AC061975.5	2.918947	-3.76911	2.99E-10	1.19E-09
AC064807.2	2.226381	-2.74952	3.00E-10	1.20E-09
AP002358.2	3.103944	-2.55896	3.01E-10	1.20E-09
OTOS	5.251398	-2.43755	3.07E-10	1.22E-09
FGF21	4.585838	-3.20611	3.07E-10	1.22E-09
MAJIN	2.531616	-0.3679	3.09E-10	1.23E-09
RN7SL752P	2.720737	-0.24305	3.12E-10	1.25E-09
AL445647.1	5.061238	-2.84778	3.14E-10	1.25E-09
LINC01524	3.239994	-3.15216	3.15E-10	1.25E-09
AL390334.1	4.282446	-2.82479	3.21E-10	1.28E-09
AC008109.1	4.354153	-1.63312	3.24E-10	1.29E-09
AC040904.1	2.137049	-3.19081	3.25E-10	1.29E-09
RNU6-140P	4.703907	-2.17274	3.27E-10	1.30E-09
NEUROD1	6.703244	1.894137	3.29E-10	1.31E-09
PSG6	5.806712	-2.44973	3.30E-10	1.31E-09
AC002378.1	3.945374	-2.67691	3.31E-10	1.31E-09
PAGE5	5.087439	-0.18839	3.33E-10	1.32E-09
AC079880.2	2.0128	-3.06215	3.34E-10	1.33E-09
DPP10	2.400824	1.963358	3.34E-10	1.33E-09
MAGEB1	6.491809	-1.8489	3.37E-10	1.34E-09
IGLV8-61	2.089176	4.776918	3.37E-10	1.34E-09
AC090833.1	3.073267	-3.07068	3.39E-10	1.35E-09
RWDD4P1	2.083398	-1.97439	3.42E-10	1.36E-09
LINC02263	4.011894	-3.54344	3.45E-10	1.37E-09
LINC01206	3.534547	-2.08312	3.47E-10	1.37E-09
C15orf54	2.204761	-1.50537	3.47E-10	1.37E-09
AC025031.5	2.881151	-2.17926	3.50E-10	1.39E-09
AC011270.2	3.54652	-3.72957	3.61E-10	1.43E-09
IGHV3-65	2.343667	-2.27797	3.62E-10	1.43E-09
AC016769.1	3.304587	-3.01722	3.65E-10	1.44E-09
UGT1A3	4.516761	-3.22919	3.65E-10	1.44E-09
KNOP1P5	4.511247	-2.55055	3.68E-10	1.45E-09
RPL26P29	2.07094	-3.83464	3.76E-10	1.49E-09
SOX21-AS1	2.212003	1.241857	3.77E-10	1.49E-09
AC114402.2	3.221326	-2.17816	3.84E-10	1.51E-09
LYPD6B	2.038803	2.773723	3.84E-10	1.52E-09
C3P1	5.259499	-2.29105	3.86E-10	1.52E-09
RNA5SP18	2.086044	-2.83599	3.89E-10	1.53E-09
AC245517.5	2.316199	-2.88308	3.93E-10	1.55E-09

For Peer Review

VCX3A	5.707643	-1.50955	3.93E-10	1.55E-09
PHYHIPL	2.401072	-0.08415	3.96E-10	1.56E-09
RPL37P3	3.346706	-4.04673	4.00E-10	1.57E-09
ABCA9-AS1	3.234648	-1.92636	4.00E-10	1.57E-09
SYT5	2.593082	0.48608	4.02E-10	1.58E-09
AL109914.1	2.38854	-3.03641	4.02E-10	1.58E-09
MRPL15P1	2.574432	-3.64357	4.12E-10	1.62E-09
LINC00355	4.36275	-0.96422	4.12E-10	1.62E-09
NTS	5.005278	6.230942	4.14E-10	1.63E-09
SNORA74D	5.409826	-2.26641	4.15E-10	1.63E-09
AC084026.3	3.758657	-3.41671	4.27E-10	1.68E-09
CSAG3	5.074601	-0.88516	4.31E-10	1.69E-09
G2E3-AS1	5.144856	-2.3327	4.36E-10	1.71E-09
LINC01356	2.236858	-1.85833	4.37E-10	1.71E-09
AF274573.1	6.58669	-1.62957	4.38E-10	1.72E-09
AC007494.3	3.479643	-2.57788	4.40E-10	1.73E-09
ATP4A	3.883027	-2.05476	4.43E-10	1.73E-09
AC020658.4	2.191023	-3.85097	4.45E-10	1.74E-09
AC040174.2	3.338685	-3.62026	4.46E-10	1.75E-09
MIR4284	2.442062	-3.64589	4.46E-10	1.75E-09
ST18	2.218777	0.589543	4.61E-10	1.81E-09
TBX18	2.636632	1.552325	4.67E-10	1.83E-09
TMEM210	2.843086	-3.21874	4.71E-10	1.84E-09
AC141273.2	3.04541	-3.22047	4.76E-10	1.86E-09
KRT16P6	3.349558	-0.49176	4.76E-10	1.86E-09
CSAG2	5.385075	-1.51412	4.78E-10	1.87E-09
RN7SKP116	2.416332	-3.77271	4.82E-10	1.88E-09
P2RY4	2.32766	-1.66819	4.85E-10	1.89E-09
SNORA74A	6.821882	-0.18113	4.86E-10	1.90E-09
GCG	8.084356	1.204735	4.88E-10	1.90E-09
AC016723.1	4.28852	-2.90724	4.92E-10	1.92E-09
TMBIM7P	2.474381	-3.6957	4.96E-10	1.93E-09
NKX2-4	5.289538	-1.82662	4.97E-10	1.94E-09
IGKV1-37	2.802845	-2.82588	5.04E-10	1.96E-09
SNORD93	2.051221	-3.21922	5.06E-10	1.97E-09
CPA5	2.082028	-1.75391	5.16E-10	2.01E-09
AC108136.1	3.347759	-3.21506	5.30E-10	2.06E-09
MIR3609	6.039962	0.161715	5.32E-10	2.07E-09
GAPDHP55	3.231111	-3.44517	5.34E-10	2.07E-09
NAA11	5.407502	-1.06759	5.35E-10	2.08E-09
AP003472.2	2.163883	-3.61143	5.40E-10	2.10E-09
AL122008.3	2.107887	-2.70549	5.42E-10	2.10E-09
SSTR5	3.82656	-2.46942	5.54E-10	2.15E-09

For Peer Review

AC084026.1	2.940033	-3.51233	5.58E-10	2.16E-09
ZIC1	4.8464	0.65053	5.63E-10	2.18E-09
AC106771.1	5.334333	-2.22392	5.63E-10	2.18E-09
LINC02152	4.461823	-3.07432	5.67E-10	2.20E-09
AP002478.1	2.990364	-1.5635	5.70E-10	2.20E-09
AL160271.1	7.020354	-0.66068	5.78E-10	2.23E-09
GJB4	2.791875	0.216322	5.79E-10	2.24E-09
AC016769.3	3.445996	-3.20634	5.81E-10	2.25E-09
AP005264.5	2.208946	-2.03821	5.84E-10	2.26E-09
UGT1A7	4.468467	-1.93025	5.93E-10	2.29E-09
SLC6A15	3.919239	0.599183	5.98E-10	2.31E-09
PSMC1P11	2.410454	-3.76709	6.00E-10	2.32E-09
AP000842.3	3.01384	-2.9973	6.04E-10	2.33E-09
SNORA49	6.519479	-0.14769	6.07E-10	2.34E-09
CYP2B6	3.911696	0.565827	6.08E-10	2.34E-09
RN7SKP80	2.115057	-1.04041	6.09E-10	2.35E-09
AC010608.2	2.616437	-2.9842	6.17E-10	2.38E-09
AC026336.3	6.20865	-0.37953	6.21E-10	2.39E-09
AC098592.1	5.922311	-2.37852	6.23E-10	2.40E-09
AC148476.1	2.059225	-1.59369	6.31E-10	2.43E-09
IGHV3-37	2.106752	-2.35265	6.34E-10	2.44E-09
LRRC38	4.939557	0.989965	6.36E-10	2.45E-09
AC021876.1	3.849851	-3.71937	6.36E-10	2.45E-09
AC114786.2	6.403411	-1.88985	6.37E-10	2.45E-09
AC025244.1	3.989137	-3.08663	6.38E-10	2.45E-09
SAMMSON	2.560532	-3.23636	6.39E-10	2.46E-09
AK4P3	2.586402	-3.45853	6.42E-10	2.47E-09
HIST1H2APS4	2.666103	-3.75002	6.46E-10	2.48E-09
SLC9A2	2.339366	1.619643	6.58E-10	2.53E-09
SPANXC	5.648936	-2.74521	6.58E-10	2.53E-09
HMX2	4.483957	-2.64031	6.59E-10	2.53E-09
RN7SL767P	3.364671	-1.36327	6.64E-10	2.55E-09
AC123768.1	2.453941	-3.99198	6.64E-10	2.55E-09
STXBP5L	3.623101	-0.27077	6.67E-10	2.56E-09
LINC00221	5.086248	-0.67219	6.77E-10	2.60E-09
LILRP2	2.623337	-1.38471	6.81E-10	2.61E-09
AL356133.2	4.516395	-3.30689	6.86E-10	2.63E-09
CYP4F3	2.995751	3.509274	6.97E-10	2.67E-09
IGHD3-16	3.097864	-3.65465	7.03E-10	2.69E-09
LINC00958	2.420157	1.017775	7.07E-10	2.70E-09
AC024581.1	2.575872	-3.79406	7.13E-10	2.72E-09
AC019171.1	2.405257	-0.35323	7.13E-10	2.73E-09
AC006946.3	2.136464	-2.98829	7.17E-10	2.74E-09

For Peer Review

AC055717.2	5.541762	-2.62332	7.24E-10	2.76E-09
AC020934.1	3.052524	-3.82924	7.30E-10	2.79E-09
LINC01571	-2.00825	-2.06828	7.34E-10	2.80E-09
UGT1A1	4.529932	-1.54038	7.44E-10	2.84E-09
HIST1H2BA	4.494499	-3.39164	7.46E-10	2.84E-09
LINC00601	3.561632	-3.05332	7.49E-10	2.85E-09
THBS4	2.342458	4.263212	7.57E-10	2.88E-09
IGFL1P1	3.10902	-2.974	7.66E-10	2.92E-09
SLC6A10P	5.029552	-2.22706	7.66E-10	2.92E-09
AL589740.1	4.063501	-3.48872	7.79E-10	2.97E-09
RN7SKP9	5.906133	-0.91815	7.80E-10	2.97E-09
AC134349.2	2.997029	-2.23211	7.84E-10	2.98E-09
PSG5	7.339648	0.554166	7.89E-10	3.00E-09
IGLJ3	2.231038	-3.02933	7.93E-10	3.01E-09
PI3	3.037968	3.94496	7.96E-10	3.02E-09
LINC01776	3.742869	-3.27253	7.98E-10	3.03E-09
DSCR4	5.840341	-2.5915	8.00E-10	3.04E-09
RNU7-75P	2.257007	-3.14465	8.07E-10	3.06E-09
AL355512.1	2.007446	-2.68528	8.10E-10	3.07E-09
AC023154.1	2.361768	-2.18766	8.31E-10	3.15E-09
ZIC4	4.951156	-0.93423	8.32E-10	3.15E-09
AC006206.2	3.115721	-0.13497	8.33E-10	3.16E-09
CTD-3080P12.3	-2.08905	-2.13373	8.41E-10	3.18E-09
PRDM7	2.291101	-3.49539	8.45E-10	3.20E-09
ZFHX4-AS1	3.900578	-2.49114	8.68E-10	3.28E-09
ATP11AUN	3.822217	-0.68024	8.71E-10	3.29E-09
FAM71F1	2.341269	-3.29478	8.86E-10	3.35E-09
POM121L2	2.380505	-2.80828	8.90E-10	3.36E-09
IGKV1D-43	2.127903	0.142211	9.00E-10	3.40E-09
IGKV1D-17	2.599592	1.187423	9.09E-10	3.43E-09
UGT2B10	3.956888	-3.6875	9.10E-10	3.43E-09
AKR1C3	2.097338	7.379219	9.12E-10	3.44E-09
LINC01449	2.099492	-3.7249	9.13E-10	3.44E-09
GABRG3	3.556725	-0.22748	9.16E-10	3.45E-09
AC084357.3	4.6941	-3.05404	9.36E-10	3.53E-09
NEFL	3.125214	1.189054	9.44E-10	3.55E-09
ADAM6	5.324566	-2.99211	9.49E-10	3.57E-09
AC005064.1	2.567486	-1.34357	9.73E-10	3.66E-09
AL772337.1	-2.04895	-1.60607	9.81E-10	3.69E-09
LINC01419	7.141383	1.069747	9.85E-10	3.70E-09
LINC01543	3.403812	-3.64771	1.01E-09	3.79E-09
SPRR3	4.517829	1.564259	1.03E-09	3.85E-09
AC022905.1	2.931283	-2.60301	1.03E-09	3.87E-09

For Peer Review

AL355994.2	2.491322	-3.63874	1.04E-09	3.91E-09
CCDC166	3.004673	-3.88073	1.05E-09	3.93E-09
NPAS3	2.382905	2.253485	1.05E-09	3.93E-09
GAGE2A	6.947306	-1.65446	1.05E-09	3.94E-09
RNU5B-1	8.870196	1.522239	1.07E-09	4.02E-09
NEK2P4	3.088247	-3.9655	1.08E-09	4.04E-09
ZNF402P	2.397624	-4.06415	1.08E-09	4.04E-09
PRR32	3.055453	-2.20072	1.09E-09	4.09E-09
AC019206.1	2.137063	-3.58475	1.10E-09	4.10E-09
RPS3AP53	2.116864	-3.66861	1.13E-09	4.21E-09
RN7SL239P	2.089459	-3.36519	1.13E-09	4.22E-09
ALOX15P2	3.068058	-0.7848	1.13E-09	4.23E-09
SLAMF6P1	3.742601	-2.91851	1.14E-09	4.25E-09
AC099487.1	3.737711	-3.4549	1.14E-09	4.27E-09
ATOH1	5.561953	-2.453	1.15E-09	4.29E-09
IBSP	2.530201	1.120006	1.15E-09	4.29E-09
AC023794.2	2.596506	-3.26518	1.16E-09	4.32E-09
AC139491.2	2.148498	-0.05373	1.16E-09	4.33E-09
AC140479.1	2.811985	-3.82181	1.17E-09	4.35E-09
LINC00319	2.626182	-3.31371	1.17E-09	4.36E-09
RNA5SP29	2.257345	-4.04337	1.18E-09	4.40E-09
GOLGA2P11	2.904308	-1.71667	1.19E-09	4.42E-09
GH2	4.595732	-3.30147	1.19E-09	4.43E-09
SPANXB1	6.523135	-0.21228	1.20E-09	4.46E-09
OR1F1	3.721836	-3.65708	1.21E-09	4.50E-09
GGT4P	2.33566	-1.28241	1.21E-09	4.51E-09
GSC	2.496535	-1.32967	1.22E-09	4.55E-09
SLC22A11	2.376829	-2.07313	1.24E-09	4.62E-09
RNU4-25P	2.958224	-3.48748	1.24E-09	4.62E-09
AC078850.2	2.967315	-2.90984	1.25E-09	4.63E-09
MALAT1	2.040499	9.58464	1.25E-09	4.64E-09
VCAN-AS1	3.159407	-2.10716	1.26E-09	4.67E-09
RF00003	2.306384	-3.06177	1.26E-09	4.68E-09
AL135929.2	5.246665	-2.99771	1.26E-09	4.68E-09
FER1L6	3.761175	2.805097	1.28E-09	4.73E-09
AL354993.1	2.173397	-1.67977	1.28E-09	4.76E-09
RPL7P42	2.183958	-3.98334	1.29E-09	4.78E-09
C1orf141	-2.04446	-0.8978	1.29E-09	4.79E-09
FER1L6-AS2	4.402335	-3.21339	1.31E-09	4.86E-09
AC005865.2	2.678773	-2.02122	1.32E-09	4.91E-09
BAGE2	5.717888	-1.41588	1.33E-09	4.91E-09
AL031736.1	2.889682	-2.66036	1.33E-09	4.92E-09
PHF2P2	4.917821	-1.84968	1.34E-09	4.94E-09

For Peer Review

PTCHD3P2	2.36453	-2.01398	1.34E-09	4.97E-09
MAGEA5	3.151255	-3.6451	1.34E-09	4.97E-09
IQCM	3.462613	-3.5665	1.37E-09	5.07E-09
LINC02404	5.573388	-1.64877	1.37E-09	5.07E-09
IMMP1LP1	2.038616	-2.88731	1.41E-09	5.22E-09
MAGEA4-AS1	6.395492	-1.93876	1.42E-09	5.25E-09
LINC00668	3.914755	-0.24603	1.42E-09	5.26E-09
LINC02192	3.410615	-3.46311	1.43E-09	5.27E-09
ISX	5.174753	-0.31451	1.44E-09	5.32E-09
TMED11P	3.37236	-1.78245	1.44E-09	5.32E-09
PRDX2P4	2.479821	-3.64929	1.46E-09	5.39E-09
IGHV1-45	2.035029	1.667409	1.49E-09	5.50E-09
CIDEC	3.25315	-0.34979	1.49E-09	5.50E-09
LINC02335	5.170834	-2.90784	1.51E-09	5.56E-09
AC016550.2	4.238252	-3.50323	1.52E-09	5.58E-09
CSAG4	5.047493	-2.98239	1.52E-09	5.59E-09
IGHV3-13	2.043753	3.3773	1.52E-09	5.61E-09
AC104574.2	3.438087	-3.14895	1.53E-09	5.62E-09
AC008115.2	3.24549	-3.40316	1.54E-09	5.66E-09
AL691420.1	5.010878	-3.0501	1.55E-09	5.70E-09
GNRH2	2.83905	-0.76492	1.56E-09	5.73E-09
LINC01667	5.942342	-0.27511	1.57E-09	5.78E-09
AC096759.2	4.513685	-2.92796	1.58E-09	5.78E-09
RNU5E-1	5.382712	-0.38788	1.58E-09	5.79E-09
AC087855.2	3.576213	-3.95364	1.61E-09	5.89E-09
NKX2-5	4.219313	-1.97133	1.62E-09	5.93E-09
AC009630.2	2.202177	-3.94987	1.62E-09	5.93E-09
CERNA3	2.04244	-3.8051	1.67E-09	6.12E-09
RN7SL128P	2.522852	-0.63124	1.67E-09	6.13E-09
AC011611.2	3.060516	-3.97292	1.68E-09	6.14E-09
AC120193.1	2.055767	-2.66912	1.71E-09	6.26E-09
AL589743.4	2.679305	-3.37285	1.71E-09	6.27E-09
AC010307.2	2.060087	-2.54122	1.72E-09	6.29E-09
MC5R	2.780222	-2.85909	1.73E-09	6.31E-09
AC067863.2	2.861652	-3.89874	1.73E-09	6.32E-09
FAM163A	2.255082	-0.68036	1.74E-09	6.35E-09
AC005291.1	2.845825	-2.74469	1.75E-09	6.38E-09
ZIC3	5.593658	-1.69387	1.75E-09	6.40E-09
IGHV1OR15-6	2.770092	-3.48449	1.77E-09	6.46E-09
LINC01913	2.861751	-1.30352	1.78E-09	6.49E-09
PATE4	3.1439	-3.64836	1.78E-09	6.51E-09
AC243967.1	2.256246	-3.32641	1.79E-09	6.53E-09
AC096734.1	2.137926	-3.36166	1.84E-09	6.70E-09

For Peer Review

TEX15	3.334073	-0.18576	1.86E-09	6.76E-09
AC083801.2	3.568156	-3.53199	1.86E-09	6.77E-09
LINC02476	5.606192	-1.98646	1.87E-09	6.82E-09
AC129926.1	2.314691	-1.66606	1.87E-09	6.83E-09
AC020910.3	2.184844	-2.69325	1.89E-09	6.88E-09
TSPAN8	2.127356	6.545116	1.94E-09	7.06E-09
AL133243.4	2.844689	-1.14796	1.97E-09	7.15E-09
GABRR1	3.446862	-0.86192	1.97E-09	7.17E-09
AP000619.1	2.916751	-3.05823	1.97E-09	7.17E-09
AC024230.1	4.070102	-3.09381	2.00E-09	7.26E-09
AC099342.1	2.719742	-3.91074	2.02E-09	7.32E-09
GJB5	2.368738	1.390088	2.02E-09	7.33E-09
AC090809.1	5.41928	-2.73505	2.03E-09	7.38E-09
AP000721.2	2.074742	-3.82094	2.06E-09	7.45E-09
RFX6	4.58331	-0.55726	2.06E-09	7.46E-09
ZNF716	4.939744	-1.88914	2.07E-09	7.51E-09
AC008496.1	2.975644	-4.01231	2.08E-09	7.53E-09
PSCA	3.400203	5.604439	2.08E-09	7.53E-09
AC092675.1	2.375418	-1.89378	2.08E-09	7.54E-09
OR2W6P	2.456751	-3.76611	2.08E-09	7.54E-09
AF230666.2	2.086133	-3.19063	2.08E-09	7.54E-09
DSPP	3.703465	-3.20416	2.09E-09	7.58E-09
IL19	2.856755	-2.25424	2.14E-09	7.74E-09
TMEM132D-AS1	5.642151	-2.1167	2.17E-09	7.83E-09
AC004888.1	2.894375	-3.24448	2.19E-09	7.92E-09
AL442224.1	3.501537	-1.20196	2.21E-09	7.98E-09
RNU6ATAC	3.2777	-2.23435	2.21E-09	7.98E-09
IGKV1D-22	3.240193	-3.90558	2.22E-09	8.01E-09
AL355483.1	2.634317	-4.01181	2.23E-09	8.05E-09
AC243972.1	2.146331	-3.75203	2.24E-09	8.09E-09
KCNJ6	2.326917	0.270344	2.24E-09	8.10E-09
TEX101	3.572143	-0.52148	2.25E-09	8.12E-09
TPI1P3	2.275162	-3.33776	2.29E-09	8.25E-09
CHRND	2.711712	-2.80021	2.30E-09	8.30E-09
AP000344.1	2.094654	-1.48149	2.34E-09	8.41E-09
FO680682.1	2.097358	-3.41766	2.47E-09	8.89E-09
HAR1B	2.227524	-1.38131	2.48E-09	8.90E-09
SLC8A1-AS1	2.286722	-0.7712	2.48E-09	8.92E-09
SNORA63D	4.700959	-3.27195	2.48E-09	8.92E-09
IFNL2	2.985098	-3.95503	2.52E-09	9.06E-09
IFNE	2.863241	-1.71377	2.52E-09	9.06E-09
LINC00645	4.670747	-3.03961	2.56E-09	9.17E-09

For Peer Review

AC006145.1	4.558035	-2.77479	2.56E-09	9.18E-09
AC099677.4	2.743741	-3.9604	2.58E-09	9.25E-09
CPS1-IT1	4.752522	-3.34657	2.58E-09	9.25E-09
IGKV2-29	3.052067	2.301526	2.63E-09	9.44E-09
IGKV6D-41	3.283675	-1.96086	2.66E-09	9.52E-09
AC110015.1	3.890308	-3.70779	2.66E-09	9.52E-09
MCRIP2P1	2.028896	-2.43304	2.66E-09	9.52E-09
SNORA22C	4.050084	-2.02791	2.67E-09	9.55E-09
LHCGR	2.608437	-1.3774	2.68E-09	9.61E-09
SCN3A	2.699269	2.648475	2.75E-09	9.83E-09
RN7SL434P	4.813998	-2.14798	2.79E-09	9.97E-09
AC131211.1	4.926784	-3.2437	2.81E-09	1.00E-08
RN7SL219P	2.764434	-3.58116	2.83E-09	1.01E-08
RN7SKP255	7.571844	-0.24039	2.85E-09	1.02E-08
IGLV3-30	2.422719	-3.10186	2.86E-09	1.02E-08
MIR4527HG	3.306977	-2.50952	2.87E-09	1.02E-08
AC006357.1	3.849102	-3.49996	2.87E-09	1.03E-08
TTLL8	3.117431	-3.49226	2.89E-09	1.03E-08
PAGE2B	5.058565	-0.55479	2.92E-09	1.04E-08
GRIA4	2.149622	-0.84464	2.98E-09	1.06E-08
AC126323.3	3.22597	-3.1077	3.03E-09	1.08E-08
AL008721.1	2.93292	-3.88622	3.05E-09	1.08E-08
TUBA3E	3.807034	-1.67325	3.08E-09	1.10E-08
FSTL5	4.492663	0.285249	3.11E-09	1.10E-08
AC100774.1	2.731617	-2.53744	3.12E-09	1.11E-08
TMEM151B	2.125738	-1.58782	3.16E-09	1.12E-08
DSG1-AS1	4.589723	-2.79611	3.19E-09	1.13E-08
APOB	3.121092	1.875582	3.19E-09	1.13E-08
AC022336.1	2.007165	-3.45653	3.24E-09	1.15E-08
SPANXD	5.436371	-2.90635	3.25E-09	1.15E-08
FMO9P	3.409763	-2.54374	3.25E-09	1.15E-08
AC113615.1	2.840561	-3.58506	3.25E-09	1.15E-08
IGLL3P	2.0407	-2.19372	3.29E-09	1.17E-08
LRFN2	2.953743	-1.26316	3.29E-09	1.17E-08
AC006065.4	3.655996	-3.20496	3.29E-09	1.17E-08
SHISAL2B	3.325449	-1.67004	3.31E-09	1.17E-08
NANOGP7	2.031936	-2.42067	3.37E-09	1.19E-08
AC103563.4	2.886919	-3.61809	3.41E-09	1.21E-08
FAT2	2.101456	2.461471	3.41E-09	1.21E-08
AC136759.1	3.531862	-3.21671	3.45E-09	1.22E-08
AC122694.1	3.495393	-3.05117	3.45E-09	1.22E-08
AL359851.1	3.257729	-3.46499	3.50E-09	1.24E-08
SNORA84	5.518431	-1.84647	3.50E-09	1.24E-08

For Peer Review

IAPP	3.624827	-3.63711	3.54E-09	1.25E-08
SKOR2	4.778294	-2.9583	3.57E-09	1.26E-08
AL136987.1	2.838877	-3.57151	3.57E-09	1.26E-08
AC005796.1	2.72845	-3.18565	3.57E-09	1.26E-08
AC110741.1	3.130445	-0.82742	3.59E-09	1.27E-08
LINC01924	4.628699	-2.90539	3.60E-09	1.27E-08
AC126177.7	2.150119	-2.50661	3.60E-09	1.27E-08
APOF	3.047903	-2.3569	3.61E-09	1.28E-08
RN7SL151P	5.197802	-2.10762	3.62E-09	1.28E-08
AC015813.4	2.080912	-3.63325	3.65E-09	1.29E-08
RNU6-652P	2.018197	-3.03015	3.71E-09	1.31E-08
KLK14	3.398723	3.459797	3.74E-09	1.32E-08
LINC02343	2.420795	-3.69782	3.74E-09	1.32E-08
CTD-2297D10.2	2.055304	-3.36299	3.77E-09	1.33E-08
AL079305.1	3.665424	-3.9184	3.80E-09	1.34E-08
SLITRK1	4.668669	-1.76931	3.88E-09	1.36E-08
LINC02267	5.408207	-2.39252	3.92E-09	1.38E-08
PSG11	6.890304	-1.71121	3.96E-09	1.39E-08
GAL3ST2	2.295106	-1.10105	3.98E-09	1.40E-08
FBN2	2.265689	2.812743	4.00E-09	1.40E-08
TMEM215	2.480891	-1.72492	4.02E-09	1.41E-08
LINC00393	4.807095	-2.51485	4.02E-09	1.41E-08
AL023754.1	3.42207	-3.67505	4.04E-09	1.42E-08
AC004158.1	3.279484	-3.37168	4.05E-09	1.42E-08
PINCR	4.302107	-3.30954	4.23E-09	1.48E-08
KNOP1P4	2.428562	-4.03408	4.24E-09	1.49E-08
AC008649.1	2.258299	-2.82143	4.32E-09	1.51E-08
APELA	2.015304	-0.3505	4.35E-09	1.52E-08
OXT	2.036911	-2.20666	4.35E-09	1.52E-08
TUSC8	3.77249	-0.62659	4.38E-09	1.53E-08
AL079307.1	2.748763	-3.13579	4.38E-09	1.53E-08
TGIF2LX	4.671151	-3.37135	4.39E-09	1.54E-08
AL365356.1	2.783362	-1.91151	4.42E-09	1.54E-08
AC020922.1	3.22008	-3.89477	4.44E-09	1.55E-08
AC090826.1	2.096414	-2.16416	4.52E-09	1.58E-08
SAGE1	4.385143	-1.05364	4.59E-09	1.60E-08
STUM	2.348939	3.091793	4.60E-09	1.60E-08
LINC01202	4.850048	-3.27118	4.66E-09	1.63E-08
AC015983.1	2.36085	-3.65374	4.68E-09	1.63E-08
LINC02454	2.732471	-2.13512	4.77E-09	1.66E-08
RBFOX1	4.116206	-0.29619	4.80E-09	1.67E-08
SSX1	6.47576	-0.33246	4.82E-09	1.68E-08
AC134981.1	3.077291	-3.20887	4.83E-09	1.68E-08

For Peer Review

AL353583.1	2.929313	-1.32403	4.83E-09	1.68E-08
AC024595.1	4.303314	-3.60284	4.85E-09	1.69E-08
AC007785.1	2.73218	-3.59378	5.01E-09	1.74E-08
BX005019.1	3.68598	-3.67552	5.01E-09	1.74E-08
LINC00330	2.139531	-2.92041	5.05E-09	1.75E-08
AC090673.2	3.362974	-3.5998	5.12E-09	1.78E-08
LINC01249	4.78986	-2.67105	5.12E-09	1.78E-08
FMR1NB	4.55587	-1.87789	5.21E-09	1.81E-08
LINC01257	4.620963	-1.40156	5.22E-09	1.81E-08
AC003986.2	2.424556	-3.67188	5.24E-09	1.82E-08
AL137803.1	3.359202	-3.52869	5.25E-09	1.82E-08
AL606970.3	3.235968	-2.54003	5.27E-09	1.83E-08
AC018470.1	3.863802	-3.74064	5.38E-09	1.87E-08
14-Sep	4.724911	-1.50775	5.45E-09	1.89E-08
AC010547.2	2.404036	-2.12824	5.49E-09	1.90E-08
VGLL2	6.690483	0.563544	5.49E-09	1.90E-08
GBX2	2.751976	-1.99606	5.52E-09	1.91E-08
AC097065.1	2.081069	-3.21514	5.55E-09	1.92E-08
RF00096	2.724453	-3.79345	5.57E-09	1.93E-08
AC083906.3	2.025756	-3.14196	5.65E-09	1.95E-08
AP000542.1	4.30802	-3.48308	5.70E-09	1.97E-08
AC005090.1	4.950408	-2.74628	5.70E-09	1.97E-08
APCS	5.230484	-3.04837	5.71E-09	1.97E-08
AL133467.2	4.034451	-2.35411	5.71E-09	1.97E-08
LCN15	4.579394	0.088879	5.82E-09	2.01E-08
AC122710.1	5.163736	-2.38877	5.85E-09	2.02E-08
NFYAP1	3.497541	-3.57452	5.87E-09	2.03E-08
LINC01345	5.292376	-2.53872	5.90E-09	2.04E-08
AL451137.1	3.479602	-3.80568	5.93E-09	2.05E-08
AF121898.1	4.441447	-2.98214	5.95E-09	2.05E-08
LRRC26	2.47421	-0.20452	5.96E-09	2.06E-08
RNVU1-7	3.754731	-1.6707	5.96E-09	2.06E-08
LINC00543	2.005359	-0.35876	6.07E-09	2.09E-08
AC123905.1	3.513223	-3.64423	6.10E-09	2.10E-08
MOCS1P1	2.623429	-3.6241	6.14E-09	2.12E-08
TRIM10	2.447214	-1.2295	6.23E-09	2.14E-08
LINC02468	2.46754	-2.68951	6.26E-09	2.16E-08
HNF4A	2.847178	2.713077	6.27E-09	2.16E-08
LINC01873	2.529011	-3.75247	6.33E-09	2.18E-08
AC106794.1	4.50065	-3.4818	6.43E-09	2.21E-08
FOXP1-IT1	3.152226	-1.15564	6.47E-09	2.22E-08
GPS2P2	3.02425	-3.90997	6.49E-09	2.23E-08
PATE2	2.572014	-3.78235	6.53E-09	2.24E-08

For Peer Review

AC080129.1	3.396304	-3.67859	6.55E-09	2.25E-08
SNORA80B	3.027117	-1.46834	6.61E-09	2.27E-08
AL590006.1	2.284725	-2.8406	6.61E-09	2.27E-08
LINC01446	4.37901	-0.64739	6.65E-09	2.28E-08
MOGAT2	2.106092	-2.07076	6.65E-09	2.28E-08
AL354824.2	2.867441	-3.77963	6.69E-09	2.29E-08
TEX37	3.845352	-3.60452	6.77E-09	2.32E-08
TENM1	2.265533	3.294313	6.82E-09	2.34E-08
ACTN3	2.418128	-1.38186	6.94E-09	2.37E-08
AC126177.6	2.077806	-2.2293	6.95E-09	2.38E-08
AC048383.1	2.435232	-3.55426	6.95E-09	2.38E-08
CGB3	4.101964	-2.79543	6.96E-09	2.38E-08
RN7SL569P	2.560448	-3.8646	7.05E-09	2.41E-08
LINC01456	4.58003	-2.97767	7.06E-09	2.41E-08
ADGRD2	2.263177	-3.42398	7.06E-09	2.41E-08
KCNJ18	5.423288	-1.30806	7.06E-09	2.41E-08
AC022929.2	2.541396	-3.60244	7.19E-09	2.45E-08
AC067945.1	2.20504	-2.23838	7.32E-09	2.50E-08
AC008750.3	2.075402	-3.54027	7.33E-09	2.50E-08
IGKV2OR2-1	2.276144	-1.92216	7.43E-09	2.53E-08
GABBR2	2.156745	2.291419	7.48E-09	2.55E-08
ALX1	3.202118	-1.36693	7.50E-09	2.55E-08
AC133540.1	2.434931	-2.51195	7.50E-09	2.56E-08
FCF1P8	2.658376	-3.05349	7.52E-09	2.56E-08
AC008738.4	2.71528	-3.68243	7.54E-09	2.57E-08
SPRR1A	3.720204	0.291556	7.55E-09	2.57E-08
RN7SL67P	2.183194	-3.35961	7.67E-09	2.61E-08
TRIM48	7.938703	0.413287	7.70E-09	2.62E-08
AP006333.2	2.500207	-3.78639	7.80E-09	2.65E-08
PPP4R3C	5.47174	-2.65044	7.84E-09	2.66E-08
HIST1H1A	5.774315	-1.9102	7.88E-09	2.68E-08
SNORA14A	3.48071	-2.32773	7.90E-09	2.68E-08
Z97206.1	2.050134	-3.82691	7.92E-09	2.69E-08
RPS27AP13	3.200974	-3.93109	8.06E-09	2.73E-08
GALR3	2.767684	-3.46675	8.12E-09	2.76E-08
RTL1	4.577637	-1.6949	8.14E-09	2.76E-08
AC005808.1	2.780388	-3.91424	8.14E-09	2.76E-08
AC021683.1	2.659522	-0.6451	8.18E-09	2.78E-08
RAP2CP1	3.013869	-3.51301	8.36E-09	2.83E-08
AC027514.1	2.616638	-2.57361	8.36E-09	2.83E-08
Z98742.3	2.061211	-3.63765	8.45E-09	2.86E-08
SPINT4	3.450418	-2.96677	8.49E-09	2.87E-08
AC087857.1	4.00467	-3.37805	8.50E-09	2.88E-08

For Peer Review

AP005271.1	2.085389	-3.37702	8.51E-09	2.88E-08
KRT8P30	2.107108	-3.28341	8.67E-09	2.93E-08
DEFA5	9.552776	1.305673	8.83E-09	2.98E-08
AC114812.2	3.249859	-3.63251	8.84E-09	2.99E-08
IGFL1	3.523112	-0.19845	8.85E-09	2.99E-08
AC069542.1	2.548291	-2.39093	8.88E-09	3.00E-08
TBR1	2.821314	-2.40327	9.01E-09	3.04E-08
AC126696.2	2.290283	-3.98766	9.18E-09	3.10E-08
TPTE	4.948607	-3.04833	9.28E-09	3.13E-08
AC104958.1	2.383162	-1.3213	9.35E-09	3.15E-08
DCAF8L2	4.809176	-2.78787	9.43E-09	3.18E-08
UGT3A1	5.819542	1.468387	9.54E-09	3.21E-08
RNU6-583P	2.09035	-4.04706	9.54E-09	3.21E-08
LINC01098	3.100409	-3.21388	9.59E-09	3.23E-08
AP002981.1	2.044502	-3.90608	9.60E-09	3.23E-08
C4BPAP2	3.171318	-2.1134	9.66E-09	3.25E-08
MUCL1	3.237954	0.913484	9.74E-09	3.27E-08
IGKV2-4	2.083971	-2.65019	9.79E-09	3.29E-08
AC005307.1	3.96023	-2.59207	9.82E-09	3.30E-08
HSPD1P11	2.161718	-1.45064	1.04E-08	3.48E-08
RMRP	6.095058	-0.97498	1.04E-08	3.49E-08
LINC01659	2.040326	-1.15929	1.05E-08	3.52E-08
SNX18P7	2.260852	-2.58116	1.05E-08	3.53E-08
IGKV2-18	2.256251	-2.31599	1.05E-08	3.53E-08
AC025030.1	2.376104	-3.93406	1.06E-08	3.56E-08
LINC00698	2.273745	-3.86086	1.07E-08	3.59E-08
AC012317.1	3.160425	-2.57933	1.07E-08	3.60E-08
DKK1	2.401138	4.115262	1.08E-08	3.60E-08
GLDCP1	2.836889	-3.55005	1.08E-08	3.61E-08
AC068858.1	2.080423	-3.91897	1.08E-08	3.62E-08
AC073323.1	2.376858	-3.54238	1.08E-08	3.62E-08
MEIOB	2.977763	-2.78481	1.09E-08	3.65E-08
NPFFR2	3.094242	-0.24558	1.11E-08	3.70E-08
MOGAT3	3.427305	-2.95565	1.12E-08	3.73E-08
RNY3	6.907614	-1.70786	1.13E-08	3.78E-08
RPS17P11	3.545658	-3.69669	1.13E-08	3.78E-08
KRT31	4.812204	-1.79828	1.15E-08	3.82E-08
C10orf71	3.336155	-1.19092	1.15E-08	3.82E-08
AC015923.1	2.862869	-2.69018	1.15E-08	3.83E-08
NLRP5	5.441997	-1.43822	1.16E-08	3.87E-08
PRB1	5.990608	-1.92119	1.18E-08	3.92E-08
AL157778.1	5.165673	-2.71443	1.20E-08	4.00E-08
UNC13C	2.107476	-1.14839	1.20E-08	4.00E-08

For Peer Review

IFNL3	2.661446	-4.00358	1.22E-08	4.07E-08
HOMER2P1	3.879374	-2.64068	1.24E-08	4.12E-08
AL109954.2	2.986519	-3.59402	1.24E-08	4.13E-08
AC091931.1	3.275612	-4.07116	1.24E-08	4.14E-08
AC092427.1	3.519201	-3.02801	1.25E-08	4.17E-08
AC104791.2	2.127183	-2.79642	1.26E-08	4.18E-08
BNIP3P2	4.711282	-3.27611	1.26E-08	4.18E-08
LINC02433	2.957104	-3.25219	1.26E-08	4.18E-08
AC100757.2	2.284533	-3.38267	1.28E-08	4.27E-08
POLR3GP1	2.114234	-2.88001	1.29E-08	4.28E-08
CCDC177	2.559272	-2.75819	1.30E-08	4.30E-08
KRT8P17	2.439934	-2.95893	1.30E-08	4.31E-08
AC092198.1	2.440871	-1.41453	1.30E-08	4.32E-08
AL513304.1	6.567197	-1.98762	1.32E-08	4.36E-08
F13B	3.630558	-3.9297	1.32E-08	4.38E-08
FAM230C	5.264341	-2.05807	1.36E-08	4.50E-08
RF02250	2.801848	-4.01253	1.38E-08	4.57E-08
AL355076.1	2.286338	-3.9007	1.39E-08	4.62E-08
AL356055.1	2.454392	-2.81703	1.40E-08	4.63E-08
AL353747.1	3.317423	-4.05287	1.41E-08	4.65E-08
SNORA7B	3.414574	-0.86342	1.41E-08	4.67E-08
AL358232.1	2.827924	-3.58346	1.41E-08	4.67E-08
LINC00392	5.645683	-2.75031	1.42E-08	4.70E-08
AC112178.1	4.048281	-3.59697	1.43E-08	4.71E-08
AC009065.6	2.001315	-3.86779	1.43E-08	4.73E-08
IGHV1OR16-4	2.509826	-3.60802	1.43E-08	4.74E-08
IGHV1OR15-1	2.256381	-3.51744	1.44E-08	4.75E-08
MKRN3	2.486304	-0.2448	1.44E-08	4.76E-08
SLC1A6	3.288014	-1.42849	1.44E-08	4.77E-08
PROX1-AS1	2.161542	-0.44573	1.46E-08	4.81E-08
FAM9C	3.969951	-0.89316	1.46E-08	4.81E-08
NT5C1A	2.369207	-1.66882	1.47E-08	4.86E-08
HSD3B2	4.459367	-1.31279	1.48E-08	4.87E-08
TUBA3C	6.625644	0.264374	1.49E-08	4.92E-08
LINC00470	3.104111	-0.61394	1.50E-08	4.94E-08
AL359636.2	2.167198	-3.31587	1.53E-08	5.04E-08
BNIP3P6	4.835874	-3.33511	1.55E-08	5.09E-08
AL592494.1	2.809694	-4.06749	1.56E-08	5.15E-08
NKX2-2	5.104485	-1.7777	1.57E-08	5.15E-08
HIST1H4F	6.272143	-1.39386	1.60E-08	5.27E-08
AC002504.1	2.693111	-4.03206	1.60E-08	5.27E-08
AC084816.1	3.189206	-2.74091	1.61E-08	5.30E-08
AL133467.4	4.149577	-2.81315	1.62E-08	5.33E-08

For Peer Review

CBLN1	2.779705	-0.15126	1.62E-08	5.33E-08
RPL23AP20	2.373464	-4.06835	1.62E-08	5.33E-08
AC025524.2	2.45218	-3.47274	1.62E-08	5.33E-08
AL353803.1	2.416333	-3.83468	1.63E-08	5.34E-08
AC068831.5	2.272918	-3.9244	1.63E-08	5.35E-08
MYH7	2.718939	-2.13031	1.66E-08	5.44E-08
RPSAP13	2.253726	-3.961	1.68E-08	5.51E-08
CNMD	4.766074	3.975819	1.70E-08	5.57E-08
IGKV2D-30	2.244473	-0.62726	1.70E-08	5.59E-08
RNA5-8SP2	7.502816	-1.02189	1.73E-08	5.67E-08
STAR	2.377893	0.060618	1.73E-08	5.68E-08
LINC02506	4.493879	-1.46683	1.75E-08	5.73E-08
RN7SKP106	3.283035	-3.58011	1.75E-08	5.73E-08
MKRN9P	3.365704	-1.85168	1.75E-08	5.74E-08
CYP11B1	6.593387	-0.64522	1.76E-08	5.77E-08
GRM5-AS1	2.174967	-3.22865	1.76E-08	5.77E-08
DEFB126	3.841436	-3.28741	1.76E-08	5.77E-08
EREG	2.556832	5.002468	1.78E-08	5.81E-08
TDRD12	2.273629	0.586579	1.80E-08	5.88E-08
AC022809.1	2.736323	-4.0256	1.81E-08	5.90E-08
AC021127.1	2.202821	-3.44259	1.82E-08	5.93E-08
AF279873.3	4.794801	-2.50952	1.83E-08	5.97E-08
AL353662.1	3.414205	-2.25725	1.86E-08	6.05E-08
AC025154.2	2.157194	1.505366	1.88E-08	6.12E-08
G6PC	5.976737	0.468152	1.90E-08	6.19E-08
ARHGAP36	5.019016	-1.40909	1.92E-08	6.24E-08
SCARNA9	2.097155	0.327762	1.92E-08	6.25E-08
TMEM238L	3.579195	-0.27113	1.94E-08	6.31E-08
AC116666.1	3.63893	-3.72611	1.96E-08	6.37E-08
AC091043.1	3.010839	-2.87014	1.97E-08	6.40E-08
C3orf67-AS1	3.278005	-3.90137	1.99E-08	6.46E-08
LINC01399	2.800255	-3.88266	2.02E-08	6.56E-08
RPS12P2	2.004035	-4.06544	2.03E-08	6.58E-08
AC007494.1	2.555824	-3.16602	2.03E-08	6.58E-08
LINC01096	2.580427	-3.17191	2.04E-08	6.62E-08
AL117190.1	2.488892	-2.20004	2.04E-08	6.62E-08
AL355102.2	2.071634	-3.4708	2.07E-08	6.70E-08
CCDC169-SOHLH2	2.170744	-3.73098	2.08E-08	6.75E-08
AC016687.3	5.049749	-2.69108	2.10E-08	6.82E-08
AC131532.1	4.134373	-2.11076	2.12E-08	6.88E-08
ANKRD20A19P	3.799386	-1.00071	2.15E-08	6.95E-08

For Peer Review

IRX4	4.33648	-1.033	2.17E-08	7.01E-08
IGKV6D-21	2.154508	1.212493	2.19E-08	7.08E-08
MIR5689HG	3.045024	-3.61703	2.19E-08	7.09E-08
SLC26A4	2.215644	3.178308	2.22E-08	7.17E-08
LSAMP-AS1	3.196948	-2.90743	2.25E-08	7.27E-08
PASD1	6.544467	0.031123	2.25E-08	7.28E-08
KCNJ13	3.041929	-1.34709	2.26E-08	7.30E-08
LINC02188	2.808525	0.285609	2.30E-08	7.43E-08
DPPA2	6.268711	-2.04052	2.30E-08	7.44E-08
MGAM2	2.648693	-0.89656	2.31E-08	7.44E-08
AL390718.1	2.584521	-4.02279	2.31E-08	7.45E-08
CTCFL	3.964297	-0.19071	2.32E-08	7.48E-08
AL160408.1	2.766095	-0.69359	2.33E-08	7.51E-08
ARHGAP26-AS1	2.420674	-2.60282	2.33E-08	7.52E-08
IGHV1OR15-4	2.934536	-3.65432	2.34E-08	7.54E-08
AC099811.1	2.337076	-2.15286	2.36E-08	7.59E-08
AC090023.2	4.160762	-3.5449	2.38E-08	7.67E-08
TMPRSS15	3.090071	-2.70996	2.40E-08	7.73E-08
SLC25A39P1	2.794529	-2.68072	2.42E-08	7.77E-08
PKP1	2.254076	5.214915	2.42E-08	7.79E-08
AL049874.1	2.367471	-3.85503	2.42E-08	7.79E-08
AC007431.2	3.785125	-3.74721	2.44E-08	7.83E-08
GPR50	4.255558	-2.31334	2.45E-08	7.88E-08
SLC17A4	3.956432	-2.69222	2.46E-08	7.92E-08
KNG1	3.352336	0.085139	2.48E-08	7.96E-08
IGKV2-26	2.061937	-1.51042	2.53E-08	8.11E-08
GABRR3	2.962502	-3.29609	2.54E-08	8.13E-08
NKX2-3	3.105328	-0.58703	2.54E-08	8.16E-08
AL445435.1	2.449743	-2.37685	2.55E-08	8.17E-08
CR382285.1	2.615419	-1.9651	2.55E-08	8.18E-08
AC243972.2	4.432064	-2.45693	2.59E-08	8.29E-08
AC083841.2	3.229019	-3.84046	2.63E-08	8.42E-08
AC025419.1	3.475807	-0.0644	2.63E-08	8.42E-08
RIMBP2	2.45404	1.596836	2.64E-08	8.46E-08
AL353753.1	2.21724	-3.81119	2.65E-08	8.49E-08
AL359853.1	2.671153	-2.88424	2.69E-08	8.62E-08
PRH2	2.348703	-1.63138	2.69E-08	8.62E-08
DAZL	2.364797	-2.38965	2.71E-08	8.65E-08
AC010478.1	2.689605	-1.00456	2.72E-08	8.71E-08
AP003351.1	2.312125	-4.0271	2.74E-08	8.74E-08
AC100800.1	4.201335	-2.12523	2.74E-08	8.75E-08
AC120498.7	5.507696	-2.61169	2.74E-08	8.75E-08

For Peer Review

LHX3	3.152347	-3.64947	2.77E-08	8.84E-08
MIR8067	2.813019	-3.8772	2.79E-08	8.91E-08
RN7SKP227	5.8387	-2.59833	2.80E-08	8.95E-08
TAAR3P	2.74264	-3.62666	2.81E-08	8.96E-08
EPHA6	2.148473	-0.84398	2.82E-08	8.99E-08
IGHV7-40	2.75212	-3.80436	2.84E-08	9.06E-08
DANT2	2.028419	-1.03098	2.89E-08	9.21E-08
XAGE5	4.468324	-3.49871	2.90E-08	9.23E-08
LINC02037	3.848091	-1.91582	2.90E-08	9.24E-08
AF127577.6	3.624176	-1.07525	2.95E-08	9.38E-08
IGKV3OR22-2	2.591484	-3.36895	3.08E-08	9.80E-08
CREB3L3	2.944817	-0.73689	3.09E-08	9.80E-08
AC073578.2	3.779671	-3.32018	3.11E-08	9.88E-08
LINC01956	3.798483	-3.12256	3.14E-08	9.95E-08
CPLX2	4.185083	4.771726	3.17E-08	1.00E-07
CHODL-AS1	3.569195	-3.54692	3.19E-08	1.01E-07
MAGEB16	6.003457	-1.92843	3.21E-08	1.02E-07
SCARNA1	4.490511	-1.93935	3.21E-08	1.02E-07
AL354685.1	6.471182	-2.03696	3.22E-08	1.02E-07
B3GALT5	2.767609	2.914345	3.29E-08	1.04E-07
LINC00602	3.69229	-3.01073	3.29E-08	1.04E-07
EDDM3A	2.764799	-3.14375	3.32E-08	1.05E-07
AC009264.1	3.443977	-2.20353	3.37E-08	1.06E-07
RPSAP71	4.056069	-3.3382	3.39E-08	1.07E-07
AL138847.2	2.979793	-2.83303	3.42E-08	1.08E-07
RN7SL838P	2.80571	-2.73696	3.43E-08	1.08E-07
IGLV3-17	2.356194	-3.62697	3.46E-08	1.09E-07
RPL23AP23	3.186449	-3.20618	3.47E-08	1.09E-07
LINC01701	2.523531	-3.81578	3.48E-08	1.10E-07
GUCY2EP	3.451647	-1.40132	3.48E-08	1.10E-07
AP003467.2	3.649478	-3.31622	3.51E-08	1.11E-07
FTHL17	6.553025	-1.20566	3.52E-08	1.11E-07
PPIAP51	2.009044	-2.41152	3.55E-08	1.12E-07
H3.Y	4.515993	-3.49475	3.56E-08	1.12E-07
AC119744.1	2.708992	-2.79026	3.56E-08	1.12E-07
ZNF560	3.809919	-0.13108	3.60E-08	1.13E-07
LINC01812	2.676827	-3.89097	3.60E-08	1.13E-07
LRRTM1	3.125986	0.71314	3.62E-08	1.14E-07
AC007848.2	3.068484	-3.71619	3.62E-08	1.14E-07
AC092881.1	2.437657	-0.51307	3.64E-08	1.15E-07
LINC02448	4.491474	-3.5055	3.66E-08	1.15E-07
LINC02582	5.558789	-1.47987	3.69E-08	1.16E-07
AC087783.2	3.032056	-2.92803	3.70E-08	1.16E-07

For Peer Review

FOXD1	2.240415	0.436283	3.70E-08	1.16E-07
IGHVII-51-2	2.536984	-4.05112	3.74E-08	1.18E-07
AC141273.3	2.890445	-3.91074	3.84E-08	1.20E-07
GACAT3	4.808021	-2.67277	3.87E-08	1.21E-07
CRP	3.641617	-1.16195	3.87E-08	1.21E-07
UBTFL8	2.402962	-3.19009	3.88E-08	1.22E-07
UPK1A	2.455626	-1.49545	3.95E-08	1.24E-07
AC069228.1	4.076736	-2.68882	3.97E-08	1.25E-07
PHGR1	3.41788	-0.90491	3.98E-08	1.25E-07
ELOA2	2.321397	-3.71214	3.98E-08	1.25E-07
ANKRD20A18P	2.119036	-4.02177	3.99E-08	1.25E-07
LINC02347	2.859222	-2.91469	4.04E-08	1.27E-07
IGHV3-54	2.052463	-3.10652	4.08E-08	1.28E-07
MUC5AC	3.772596	8.607166	4.08E-08	1.28E-07
UHRF2P1	3.423555	-2.06553	4.09E-08	1.28E-07
AC016559.2	2.259258	-3.876	4.09E-08	1.28E-07
GAGE1	6.080872	-1.97647	4.12E-08	1.29E-07
AGMO	2.068107	-0.02026	4.17E-08	1.30E-07
IGHVII-60-1	2.555862	-3.9227	4.20E-08	1.31E-07
PES1P1	4.299399	-3.51295	4.20E-08	1.31E-07
LINC01681	3.607416	-3.56766	4.23E-08	1.32E-07
FOXR2	4.996379	-3.21123	4.27E-08	1.33E-07
BARHL2	4.222515	-3.20022	4.29E-08	1.34E-07
LINC00871	3.697112	-2.52629	4.30E-08	1.34E-07
AL157937.1	2.829184	-3.79037	4.31E-08	1.34E-07
AL138759.1	2.605001	-3.72335	4.37E-08	1.36E-07
NEUROD4	6.243091	-1.96044	4.41E-08	1.37E-07
PSG8	5.550932	-2.29621	4.42E-08	1.38E-07
AC007431.1	2.842163	-3.1068	4.42E-08	1.38E-07
TH	2.264938	-1.62064	4.44E-08	1.38E-07
AC142384.1	3.772337	-3.74719	4.54E-08	1.41E-07
PTPRZ1	2.004414	3.33258	4.54E-08	1.41E-07
RN7SL507P	4.646209	-2.0163	4.66E-08	1.45E-07
AC063952.1	2.530102	-3.01871	4.66E-08	1.45E-07
HIST1H2BPS2	2.201726	-3.46761	4.68E-08	1.46E-07
Z94160.1	2.175485	-3.36717	4.69E-08	1.46E-07
KLF2P4	4.010715	-3.05357	4.69E-08	1.46E-07
PHKBP2	3.626935	-2.44489	4.71E-08	1.46E-07
KCNK2	2.198472	0.044251	4.82E-08	1.50E-07
AC069061.2	5.330426	-2.67579	4.83E-08	1.50E-07
LINC02438	3.702016	-3.11794	4.85E-08	1.50E-07
LIPF	4.765212	-1.417	4.86E-08	1.51E-07

For Peer Review

MACC1-AS1	3.027757	-2.12492	4.89E-08	1.52E-07
AP004290.1	2.525705	-3.1169	4.90E-08	1.52E-07
PHOX2A	3.761436	-2.94029	4.98E-08	1.54E-07
HMGB3P13	3.355672	-3.98067	5.03E-08	1.56E-07
AL451047.1	3.265455	-3.12555	5.05E-08	1.56E-07
CACNA1B	2.583048	0.220378	5.12E-08	1.58E-07
AC126407.1	2.728445	-3.62579	5.13E-08	1.59E-07
ASH1L-IT1	2.455736	-3.20472	5.16E-08	1.59E-07
AL121652.1	2.051463	-3.06234	5.17E-08	1.60E-07
LINC02417	2.816188	-3.05644	5.18E-08	1.60E-07
AC007389.3	2.278161	-3.91284	5.20E-08	1.61E-07
FAM9A	4.436518	-3.56564	5.21E-08	1.61E-07
ZNF556	2.023566	-1.56102	5.22E-08	1.61E-07
AC104793.1	4.596703	-3.41877	5.25E-08	1.62E-07
AC025881.1	3.899838	-3.79754	5.36E-08	1.65E-07
ADAM18	3.259454	-3.71023	5.37E-08	1.66E-07
GS1-279B7.1	2.60197	-3.30805	5.38E-08	1.66E-07
KRTAP3-1	3.114325	-1.86995	5.40E-08	1.67E-07
AC068580.2	2.492321	-3.71118	5.42E-08	1.67E-07
CNTNAP5	3.74527	-2.85263	5.43E-08	1.67E-07
AL138962.1	2.543487	-3.7511	5.45E-08	1.68E-07
SALL4P7	2.550144	-3.86598	5.48E-08	1.69E-07
SNORA20	3.704477	-1.66007	5.57E-08	1.71E-07
RPS6P12	2.988592	-2.52505	5.57E-08	1.71E-07
PTPN2P1	2.401656	-2.8181	5.58E-08	1.72E-07
AL160408.4	3.700191	-3.71737	5.66E-08	1.74E-07
AC021534.1	4.181138	-3.6923	5.72E-08	1.76E-07
AL391097.1	3.836722	-3.57077	5.93E-08	1.82E-07
SULT1C3	4.185372	-3.35099	5.97E-08	1.83E-07
AC022336.3	2.090028	-2.40518	6.01E-08	1.84E-07
LINC01981	3.256619	-4.07553	6.05E-08	1.85E-07
AC006206.1	3.506951	-3.76453	6.11E-08	1.87E-07
BNIP3P40	2.117442	-2.01708	6.14E-08	1.88E-07
NELL1	3.134525	3.125663	6.17E-08	1.89E-07
DCAF8L1	3.971115	-3.57299	6.19E-08	1.89E-07
RN7SL149P	4.544911	-3.48881	6.20E-08	1.90E-07
KU-MEL-3	2.788796	-3.40072	6.30E-08	1.93E-07
LINC01210	4.805397	-2.79363	6.41E-08	1.96E-07
OR56A3	4.779609	-3.01895	6.43E-08	1.97E-07
AL161729.2	2.636427	-3.3033	6.44E-08	1.97E-07
RNU1-13P	5.439796	-1.93644	6.50E-08	1.98E-07
KLRF2	2.723444	-2.88866	6.60E-08	2.01E-07
CAPNS2	2.571529	0.008186	6.61E-08	2.02E-07

For Peer Review

Z82173.1	3.109259	-3.20992	6.68E-08	2.04E-07
GTF2IP6	5.126443	-2.91448	6.72E-08	2.05E-07
RNU6-915P	2.858469	-3.72433	6.86E-08	2.09E-07
MAGEA9	4.505656	-3.48964	6.87E-08	2.09E-07
HTR3B	4.316955	-1.58491	6.90E-08	2.10E-07
AL512283.1	2.272551	-3.47408	6.92E-08	2.11E-07
TMEM14EP	3.461896	-1.79824	7.14E-08	2.17E-07
AC104794.3	3.894777	-3.50037	7.16E-08	2.18E-07
SPAG11B	6.812609	-1.16897	7.20E-08	2.19E-07
AL391152.1	2.152084	-1.8108	7.22E-08	2.20E-07
AL391811.1	3.19528	-3.3117	7.24E-08	2.20E-07
OVAAL	3.093237	-2.85174	7.30E-08	2.22E-07
SNORA80D	2.469918	-2.29125	7.31E-08	2.22E-07
SPATA16	3.604794	-3.79527	7.32E-08	2.22E-07
NHS-AS1	2.795153	-3.74716	7.33E-08	2.23E-07
AC012594.1	2.544055	-3.6297	7.45E-08	2.26E-07
IGHD2-2	2.045369	-3.46172	7.46E-08	2.26E-07
FAM83C	3.784703	-2.21229	7.52E-08	2.28E-07
AC108865.1	2.85692	-3.49039	7.58E-08	2.30E-07
AC022335.1	4.132367	-3.69636	7.60E-08	2.30E-07
RN7SKP79	4.3274	-3.60181	7.63E-08	2.31E-07
NWD2	3.137881	-3.18388	7.65E-08	2.32E-07
PAGE1	5.135451	-0.47401	7.70E-08	2.33E-07
RNA5SP174	3.285056	-3.70717	7.73E-08	2.34E-07
LILRP1	3.699981	0.602889	7.77E-08	2.35E-07
Z86062.1	2.307384	-3.32851	7.77E-08	2.35E-07
AL133370.2	3.298172	-3.34205	7.84E-08	2.37E-07
ALPI	4.349502	-3.05416	7.86E-08	2.38E-07
AC008554.1	2.236502	-3.13573	7.95E-08	2.40E-07
AL355607.2	2.489923	-3.88118	7.96E-08	2.40E-07
AP005137.2	3.05381	-3.37875	8.05E-08	2.43E-07
EN1	2.969484	-2.28928	8.08E-08	2.44E-07
FGF3	4.488188	-2.60848	8.09E-08	2.44E-07
RPS15AP30	2.008709	-2.87985	8.10E-08	2.45E-07
TAS2R13	2.960603	-2.6662	8.11E-08	2.45E-07
AC145207.1	2.086394	-4.01248	8.21E-08	2.48E-07
AC091212.1	2.017891	-3.27742	8.38E-08	2.53E-07
CLEC2A	4.892817	-1.61009	8.39E-08	2.53E-07
FAM69C	2.272922	-2.23115	8.41E-08	2.54E-07
AC022639.1	4.034745	-3.31581	8.45E-08	2.55E-07
AC027088.3	3.270201	-3.81117	8.60E-08	2.59E-07
AC108471.1	2.668091	-2.73819	8.62E-08	2.60E-07
ACTL6B	3.465372	-1.63674	8.70E-08	2.62E-07

FGF12-AS2	2.393331	-3.43299	8.71E-08	2.62E-07
RN7SKP71	5.038813	-2.56327	8.81E-08	2.65E-07
GAST	3.591408	-3.62169	8.92E-08	2.68E-07
MCCD1	3.830578	-2.43491	8.98E-08	2.69E-07
SPAG11A	6.661566	-1.56743	9.00E-08	2.70E-07
AC090515.4	2.068383	-1.14106	9.07E-08	2.72E-07
AC004704.1	4.347109	-1.91439	9.23E-08	2.77E-07
RNU1-70P	3.139228	-3.70183	9.25E-08	2.77E-07
AC005702.2	2.744406	-3.44773	9.27E-08	2.78E-07
E2F3-IT1	3.638876	-3.39296	9.30E-08	2.79E-07
ST8SIA6-AS1	3.193725	0.402871	9.42E-08	2.82E-07
KRT8P38	2.803286	-3.89995	9.42E-08	2.82E-07
AL445209.1	2.969245	-3.82724	9.47E-08	2.83E-07
LINC00308	3.277724	-4.07015	9.53E-08	2.85E-07
SPIC	3.254586	-1.7602	9.63E-08	2.88E-07
NAALADL2-AS3	4.02689	-3.42073	9.63E-08	2.88E-07
AC108860.1	2.296424	-2.90207	1.01E-07	3.02E-07
AL135787.1	2.062945	-3.58601	1.02E-07	3.03E-07
IGHJ2P	2.059046	-3.64187	1.02E-07	3.03E-07
ZNF114-AS1	2.281722	-3.26626	1.03E-07	3.06E-07
SNORA38	3.670584	-2.38543	1.03E-07	3.07E-07
AC091181.1	2.005776	-1.94157	1.03E-07	3.08E-07
EVX2	3.325027	-3.86251	1.05E-07	3.11E-07
Z99289.2	2.099764	-2.85445	1.05E-07	3.12E-07
AC073530.1	3.570166	-3.88255	1.05E-07	3.12E-07
IGDCC3	2.474711	-0.70926	1.06E-07	3.15E-07
AC140658.5	2.487612	-3.95207	1.06E-07	3.16E-07
RN7SL648P	3.022328	-1.66826	1.07E-07	3.19E-07
SNORA28	2.32698	-2.32425	1.07E-07	3.19E-07
AC002076.2	3.329769	-3.88419	1.07E-07	3.19E-07
AC027243.1	2.255452	-3.69978	1.08E-07	3.22E-07
AC015712.4	2.698442	-3.33388	1.08E-07	3.22E-07
AL391361.3	4.253301	-3.45535	1.09E-07	3.23E-07
KRT33B	3.241985	-3.81967	1.10E-07	3.27E-07
LYZL2	3.491947	-3.91854	1.11E-07	3.29E-07
CGB8	3.542904	-3.21192	1.11E-07	3.31E-07
ANKRD33	2.148124	-2.74885	1.12E-07	3.31E-07
KRT33A	5.20501	-2.55263	1.13E-07	3.35E-07
LINC01910	2.51746	-3.46633	1.13E-07	3.35E-07
AL359095.1	4.503709	-2.43392	1.14E-07	3.37E-07
IGKV2D-18	2.180276	-3.06002	1.14E-07	3.39E-07
AL136018.1	2.47329	-2.99138	1.15E-07	3.40E-07

For Peer Review

RN7SKP203	6.232497	-1.35228	1.18E-07	3.50E-07
IGKV3D-25	2.552776	-3.91961	1.20E-07	3.54E-07
MAGEB2	4.961011	0.830096	1.20E-07	3.54E-07
PTH2R	2.005327	-0.19506	1.20E-07	3.54E-07
AL845331.3	2.430881	-3.66906	1.20E-07	3.56E-07
LINC01079	3.365193	-2.61035	1.21E-07	3.58E-07
AWAT1	2.652727	-3.00281	1.21E-07	3.59E-07
KRT34	3.251292	-3.55173	1.22E-07	3.60E-07
SCARNA3	4.256435	-3.13274	1.24E-07	3.65E-07
AC139792.1	3.089804	-2.87773	1.25E-07	3.68E-07
AC073957.1	2.050239	-3.56598	1.26E-07	3.70E-07
SNORD67	2.478999	-3.36992	1.26E-07	3.72E-07
AC018692.2	3.900255	-3.84603	1.28E-07	3.76E-07
AP003062.1	4.22442	-2.71913	1.28E-07	3.76E-07
IGLCOR22-1	2.308232	-3.76856	1.28E-07	3.77E-07
LINC01731	2.268806	-3.74355	1.29E-07	3.80E-07
LINC02196	3.644836	-3.67677	1.31E-07	3.84E-07
ZNF736P9Y	4.378921	-3.16979	1.31E-07	3.85E-07
AC090164.2	2.340186	-3.23568	1.31E-07	3.85E-07
AP004289.1	3.280739	-2.84342	1.32E-07	3.88E-07
AP005328.2	2.889675	-2.90576	1.32E-07	3.89E-07
AC092573.2	2.206923	-3.70002	1.33E-07	3.90E-07
AC007529.1	3.003631	-3.88469	1.35E-07	3.95E-07
SMILR	2.585824	-2.95229	1.35E-07	3.96E-07
AC091868.2	2.250648	-3.78823	1.35E-07	3.96E-07
AC009884.1	3.76732	-3.24653	1.35E-07	3.97E-07
QRSL1P3	3.076568	-2.24115	1.39E-07	4.06E-07
VN1R54P	3.577209	-3.95024	1.40E-07	4.09E-07
IGKV1D-13	2.04848	1.687475	1.40E-07	4.10E-07
AC068985.1	3.529934	-3.71701	1.40E-07	4.11E-07
DDX43P3	3.671766	-2.78867	1.41E-07	4.13E-07
LINC02167	3.035114	-4.04631	1.41E-07	4.14E-07
LINC01959	3.558473	-3.86169	1.42E-07	4.15E-07
CR392039.3	3.73604	-3.48419	1.43E-07	4.18E-07
LINC01346	4.558973	-2.46955	1.43E-07	4.19E-07
AC103957.1	2.221343	-3.98533	1.43E-07	4.19E-07
RN7SL395P	2.493623	-2.88961	1.43E-07	4.20E-07
CASC20	3.410335	-2.33002	1.44E-07	4.21E-07
GPR22	2.724878	-1.22988	1.44E-07	4.22E-07
AC114786.1	4.772094	-2.71287	1.45E-07	4.23E-07
AL121956.1	2.697713	-2.58696	1.46E-07	4.27E-07
BPIFB6	3.926948	-3.27269	1.46E-07	4.27E-07
AC011933.2	2.656414	-1.27745	1.47E-07	4.29E-07

For Peer Review

CERS3	3.083481	-0.97721	1.47E-07	4.30E-07
AC079915.1	2.74638	-1.85767	1.48E-07	4.33E-07
MAGEB6	3.451465	-3.78053	1.48E-07	4.34E-07
HNRNPA1P3	2.618176	-3.52123	1.49E-07	4.34E-07
HCN1	2.690065	0.170065	1.49E-07	4.34E-07
AL359094.1	2.098767	-2.11989	1.49E-07	4.36E-07
AC090503.2	3.360107	-4.03991	1.50E-07	4.37E-07
LINC01740	3.309291	-1.7799	1.52E-07	4.43E-07
OR10G2	2.275558	-3.81534	1.52E-07	4.44E-07
AC243972.3	3.188805	-3.9487	1.52E-07	4.44E-07
AL450345.1	3.370074	-3.60023	1.53E-07	4.45E-07
AC124290.1	3.967644	-3.76657	1.55E-07	4.50E-07
SNORA46	3.348201	-2.91834	1.55E-07	4.53E-07
PLCZ1	2.688912	-2.41515	1.57E-07	4.58E-07
AC112176.1	2.851703	-3.39021	1.58E-07	4.59E-07
RN7SL665P	3.215015	-2.05274	1.59E-07	4.63E-07
CTAG2	5.597474	0.886291	1.60E-07	4.66E-07
KRT23	2.413963	2.854944	1.61E-07	4.68E-07
AC019211.1	2.464841	-3.96531	1.61E-07	4.68E-07
HOTTIP	3.44511	-3.07402	1.62E-07	4.70E-07
IGKV1OR9-1	2.405803	-3.89074	1.63E-07	4.73E-07
RNU4ATAC	2.444983	-1.95769	1.63E-07	4.73E-07
FRG2	3.929906	-3.79206	1.63E-07	4.73E-07
GLYATL1P4	2.314179	-2.55085	1.64E-07	4.76E-07
LINC02105	3.237038	-3.84166	1.64E-07	4.77E-07
AP005060.1	3.037377	-2.10001	1.65E-07	4.79E-07
AL161431.1	2.897811	1.975319	1.65E-07	4.80E-07
AC099673.1	3.685032	-3.92251	1.66E-07	4.81E-07
IGHD2-15	2.446758	-3.75821	1.66E-07	4.82E-07
DUX4L9	2.981545	-3.4602	1.66E-07	4.82E-07
AIG1P1	3.578192	-3.96226	1.67E-07	4.85E-07
CYP4F8	3.70516	-2.74394	1.68E-07	4.87E-07
SERPINA7	4.126794	-3.37996	1.69E-07	4.90E-07
INS	5.732738	-2.27864	1.70E-07	4.91E-07
AC087473.1	2.303933	-3.96412	1.70E-07	4.92E-07
LINC02411	4.340117	-3.59565	1.75E-07	5.07E-07
AF178030.1	4.152027	-2.78057	1.76E-07	5.10E-07
AC106875.1	4.18061	-1.54028	1.77E-07	5.11E-07
AC092916.1	4.285634	-3.60445	1.77E-07	5.11E-07
SOX3	3.678028	-2.59448	1.77E-07	5.12E-07
SIRLNT	4.95287	-2.38663	1.80E-07	5.21E-07
AF130417.1	3.548233	-3.77039	1.84E-07	5.30E-07
AC005522.1	2.080168	-3.05433	1.85E-07	5.35E-07

CACNG5	2.86557	-3.71438	1.86E-07	5.36E-07
PPP1R1AP2	4.244912	-3.64268	1.86E-07	5.37E-07
TBC1D3P1	2.564999	-3.89377	1.87E-07	5.40E-07
AL512504.1	2.562604	-3.46124	1.87E-07	5.40E-07
KLK3	-2.29953	-2.03561	1.87E-07	5.40E-07
BNIP3P7	3.7977	-3.75452	1.88E-07	5.40E-07
RNU6-77P	3.917475	-3.79019	1.88E-07	5.42E-07
AL158819.1	4.042727	-2.64518	1.89E-07	5.45E-07
GLYATL3	4.485517	-2.83947	1.90E-07	5.48E-07
KC877982.1	4.29129	-3.10084	1.90E-07	5.49E-07
RPS16P5	2.622504	-2.61472	1.91E-07	5.51E-07
LINC01896	5.034886	-2.5229	1.91E-07	5.51E-07
AL139010.1	2.832445	-3.99758	1.92E-07	5.52E-07
AC006484.1	4.61486	-3.43178	1.93E-07	5.55E-07
TAS2R30	3.26364	-1.86863	1.94E-07	5.59E-07
AC083967.1	2.31843	-2.86324	1.95E-07	5.59E-07
AC133561.1	3.377995	-3.53633	1.96E-07	5.63E-07
AC022031.2	3.584872	-2.78516	1.97E-07	5.67E-07
LINC01608	5.201427	-2.55715	2.00E-07	5.73E-07
AC133106.1	3.580323	-3.67999	2.01E-07	5.77E-07
SLC7A3	2.83512	-1.36648	2.02E-07	5.81E-07
IGLV3-24	2.158329	-3.45668	2.03E-07	5.84E-07
LINC01221	3.830595	-3.82114	2.06E-07	5.91E-07
LINC01594	2.047739	-2.69134	2.12E-07	6.06E-07
AP004607.7	3.4453	-3.80218	2.14E-07	6.11E-07
PCNAP4	2.835995	-3.98561	2.14E-07	6.12E-07
GRM7-AS3	3.391263	-4.02714	2.15E-07	6.15E-07
CLLU1OS	2.277953	-1.79699	2.15E-07	6.15E-07
OLIG3	4.96237	-2.46861	2.16E-07	6.18E-07
NEUROG2	3.018503	-3.73372	2.17E-07	6.19E-07
PLCB1-IT1	4.4432	-3.54132	2.20E-07	6.29E-07
AC073365.1	4.803848	-2.13354	2.21E-07	6.30E-07
AC097716.1	3.298532	-3.70536	2.21E-07	6.31E-07
AC037487.2	3.400691	-2.40498	2.22E-07	6.34E-07
TEX13B	3.876608	-3.4971	2.23E-07	6.35E-07
MROH2A	2.233638	0.145072	2.24E-07	6.38E-07
RNA5-8SP4	5.816587	-2.65894	2.24E-07	6.38E-07
AC131097.1	2.278146	-3.30547	2.26E-07	6.45E-07
LINC02141	4.021829	-3.06718	2.27E-07	6.46E-07
AL391863.2	4.625105	-2.66375	2.29E-07	6.53E-07
RSPH6A	2.873927	-3.19447	2.31E-07	6.58E-07
RN7SL296P	4.09563	-2.31814	2.32E-07	6.59E-07
LINC01831	4.955531	-3.25004	2.32E-07	6.60E-07

For Peer Review

AC092168.2	2.099256	-2.34702	2.32E-07	6.61E-07
DNAJB3	3.271321	-3.14258	2.33E-07	6.62E-07
LINC01204	2.353878	-2.87118	2.35E-07	6.69E-07
ADH4	2.534512	-0.63933	2.37E-07	6.74E-07
GPR1-AS	4.517429	-1.99384	2.41E-07	6.83E-07
CARD18	3.91812	-3.46625	2.41E-07	6.86E-07
LINC00706	2.350656	-3.19718	2.43E-07	6.90E-07
BMP2KL	4.641286	-2.41123	2.47E-07	7.00E-07
AP001885.1	2.305115	-1.82825	2.47E-07	7.01E-07
AC090541.1	2.965869	-2.60169	2.50E-07	7.09E-07
OR7E104P	2.879501	-3.93214	2.52E-07	7.13E-07
AC073525.1	3.094006	-3.2795	2.56E-07	7.26E-07
AC008277.1	2.840785	-1.82565	2.57E-07	7.27E-07
LINC02111	2.780316	-3.7617	2.58E-07	7.29E-07
AC011287.1	2.297834	-3.42146	2.59E-07	7.34E-07
FGF4	4.515445	-2.92158	2.63E-07	7.45E-07
RN7SL342P	4.792687	-3.22074	2.67E-07	7.56E-07
MMP20	2.724418	-3.3858	2.68E-07	7.57E-07
AC104088.1	2.955692	-2.77434	2.68E-07	7.58E-07
RN7SL664P	3.577754	-3.06162	2.73E-07	7.70E-07
RAX	3.498572	-2.23859	2.74E-07	7.72E-07
AC104763.3	3.092346	-3.24638	2.77E-07	7.82E-07
AC005828.2	3.044812	-3.2936	2.80E-07	7.88E-07
AC090502.4	3.704824	-3.57546	2.80E-07	7.90E-07
AL158801.1	2.604423	-2.53148	2.81E-07	7.91E-07
AL358216.1	2.383639	-2.54336	2.83E-07	7.96E-07
AC092903.1	2.556368	-3.09817	2.83E-07	7.96E-07
AC016710.1	4.065005	-3.72642	2.84E-07	8.00E-07
AC022173.1	3.191149	-2.32987	2.87E-07	8.08E-07
AL354824.1	2.433548	-4.00031	2.88E-07	8.11E-07
AC007159.1	4.73327	-2.11384	2.89E-07	8.13E-07
ANKRD30BP2	3.578644	-3.94675	2.89E-07	8.13E-07
SCHLAP1	3.953489	-3.56042	2.93E-07	8.25E-07
LINC01502	2.821344	-1.75528	2.93E-07	8.25E-07
LINC01411	2.798267	-3.59111	2.95E-07	8.29E-07
AC091173.1	2.77079	-2.66753	3.06E-07	8.60E-07
AC005972.1	4.218534	-3.66334	3.07E-07	8.60E-07
UGT2A3	4.917588	-0.24038	3.09E-07	8.67E-07
POU3F3	3.932039	-1.47783	3.10E-07	8.69E-07
GAPDHP46	2.140906	-3.83911	3.11E-07	8.71E-07
AL445531.1	2.168003	-3.72363	3.11E-07	8.72E-07
ZG16	2.37859	-1.40464	3.11E-07	8.73E-07
AL022097.1	2.100975	-2.75418	3.15E-07	8.81E-07

For Peer Review

AC078950.1	2.956699	-3.53496	3.16E-07	8.83E-07
LINC02294	3.051474	-3.68636	3.19E-07	8.92E-07
AC012174.1	2.939983	-2.24773	3.19E-07	8.93E-07
BTF3P12	2.276712	-2.73498	3.21E-07	8.97E-07
AL022314.1	2.510522	-3.54168	3.21E-07	8.98E-07
AC005326.1	2.062846	-3.52381	3.24E-07	9.04E-07
RPE65	2.96903	-2.15224	3.24E-07	9.04E-07
AC053503.1	2.233783	-3.93133	3.24E-07	9.05E-07
NPM1P47	2.419339	-3.96314	3.27E-07	9.14E-07
PSMA2P2	2.752226	-3.73673	3.28E-07	9.15E-07
CFHR5	2.628744	-3.80782	3.29E-07	9.20E-07
AC106794.2	3.628611	-3.9205	3.40E-07	9.47E-07
AC092143.1	2.222134	-4.02403	3.40E-07	9.47E-07
AC005550.2	2.962328	-1.13993	3.44E-07	9.58E-07
RPL5P13	3.213274	-3.69271	3.46E-07	9.64E-07
AC131182.1	3.148009	-2.46307	3.47E-07	9.66E-07
AL135978.1	2.094119	-3.37534	3.49E-07	9.73E-07
AC096721.1	3.513281	-3.98104	3.50E-07	9.75E-07
RN7SL564P	4.281517	-2.76699	3.52E-07	9.79E-07
AC092801.1	2.836561	-2.24025	3.61E-07	1.00E-06
AL139274.1	4.506506	-2.22594	3.65E-07	1.01E-06
BANF2	2.952927	-3.84723	3.66E-07	1.02E-06
AP001442.1	2.578681	-1.74903	3.66E-07	1.02E-06
OR51E2	2.39028	-2.23639	3.68E-07	1.02E-06
AC010424.1	2.298931	-4.03014	3.71E-07	1.03E-06
GPR52	2.53017	-2.71464	3.71E-07	1.03E-06
NOTO	2.562319	-1.48979	3.75E-07	1.04E-06
OSTCP8	2.56004	-3.18589	3.75E-07	1.04E-06
LINC02327	3.971522	-2.66949	3.75E-07	1.04E-06
SINHCAFP3	2.511384	-3.97305	3.82E-07	1.06E-06
RF00139	2.272443	-3.96746	3.83E-07	1.06E-06
LINC01992	4.182435	-2.63691	3.83E-07	1.06E-06
RN7SL217P	4.535362	-3.49482	3.89E-07	1.08E-06
LINC01391	3.281008	-3.53419	3.92E-07	1.09E-06
NKX6-3	2.24896	-2.78683	3.93E-07	1.09E-06
AL162413.1	3.104185	-2.46601	3.95E-07	1.09E-06
CLEC2L	2.479816	-0.58207	3.98E-07	1.10E-06
AC104164.2	3.306331	-3.87122	3.99E-07	1.10E-06
AC105760.1	2.854333	-3.51655	4.02E-07	1.11E-06
AF127577.2	2.87901	-2.29529	4.03E-07	1.11E-06
DUOXA2	2.291493	1.778459	4.03E-07	1.11E-06
NTSR2	3.7134	-3.10393	4.04E-07	1.11E-06
SNORA40B	4.109415	-3.22398	4.08E-07	1.13E-06

For Peer Review

AC110760.3	2.637719	-3.54924	4.10E-07	1.13E-06
AC006372.1	3.418501	-3.24231	4.10E-07	1.13E-06
AL135929.1	3.88838	-3.79417	4.15E-07	1.15E-06
AC126773.3	2.313787	-3.66411	4.16E-07	1.15E-06
TESC-AS1	2.23428	-2.91977	4.17E-07	1.15E-06
Z82205.1	3.306972	-3.81591	4.20E-07	1.16E-06
AL512326.1	3.380308	-2.79675	4.21E-07	1.16E-06
GALP	3.055208	-4.01601	4.21E-07	1.16E-06
C11orf44	2.297468	-3.4521	4.23E-07	1.17E-06
PIGFP2	4.329466	-3.6094	4.25E-07	1.17E-06
LINC01312	3.225693	-3.42689	4.25E-07	1.17E-06
LINC02463	2.299399	-3.80257	4.28E-07	1.18E-06
STMN2	2.084358	0.955271	4.29E-07	1.18E-06
CU104787.1	3.840377	-3.83106	4.31E-07	1.19E-06
RN7SL116P	3.744538	-2.6558	4.36E-07	1.20E-06
APOA4	3.918407	-2.92498	4.43E-07	1.22E-06
GJA10	3.451879	-3.99833	4.49E-07	1.23E-06
AC002511.2	2.576204	-2.54275	4.50E-07	1.24E-06
ZNF444P1	2.306558	-3.04832	4.58E-07	1.26E-06
AC007494.2	2.711165	-2.7332	4.64E-07	1.27E-06
AC009135.1	3.855669	-3.83283	4.64E-07	1.27E-06
AC010486.3	3.769997	-3.87934	4.67E-07	1.28E-06
AC023824.6	4.826832	-1.02156	4.71E-07	1.29E-06
RNU1-120P	2.255146	-3.94208	4.76E-07	1.30E-06
HSPA8P13	3.657211	-3.92319	4.77E-07	1.30E-06
AC007431.3	2.353974	-3.76414	4.82E-07	1.32E-06
AC100782.1	2.398673	-3.492	4.84E-07	1.32E-06
IL20RB-AS1	2.527855	-3.86225	4.84E-07	1.32E-06
VDAC1P4	2.425668	-3.74622	4.90E-07	1.34E-06
KIF18BP1	3.645895	-3.75906	4.96E-07	1.35E-06
AC093817.1	2.515273	-3.5701	5.03E-07	1.37E-06
SNORA47	2.420474	-2.3082	5.10E-07	1.39E-06
AP005901.1	3.299794	-3.86341	5.13E-07	1.40E-06
IGLV3-15	2.378392	-3.85138	5.13E-07	1.40E-06
AL356057.1	4.356044	-2.60216	5.17E-07	1.41E-06
RLIMP1	3.173513	-1.26921	5.21E-07	1.42E-06
AL355336.1	2.271556	-2.2679	5.22E-07	1.42E-06
AP005119.2	2.857174	-2.73116	5.25E-07	1.43E-06
AC018816.2	2.157786	-3.61379	5.27E-07	1.44E-06
AC078852.2	2.005123	-2.96934	5.29E-07	1.44E-06
RN7SL546P	3.348433	-0.95368	5.31E-07	1.44E-06
AC091812.1	3.25175	-3.59986	5.33E-07	1.45E-06
AC104651.2	4.068832	-3.60476	5.34E-07	1.45E-06

For Peer Review

GGT8P	2.229678	-3.27544	5.44E-07	1.48E-06
AC074367.1	2.462042	-3.31251	5.48E-07	1.49E-06
RNU5A-8P	4.198293	-3.4994	5.50E-07	1.49E-06
TRIM49	3.477199	-3.88068	5.54E-07	1.51E-06
AC034105.3	2.86008	-3.37404	5.60E-07	1.52E-06
AC009303.2	2.350866	-2.36976	5.65E-07	1.54E-06
MTDHP4	2.174193	-3.38933	5.70E-07	1.55E-06
RN7SL508P	3.537449	-3.02409	5.74E-07	1.56E-06
RNU1-88P	5.065512	-2.21929	5.74E-07	1.56E-06
HNRNPA1P52	2.113918	-1.96212	5.78E-07	1.57E-06
AC005150.1	3.715302	-3.90403	5.80E-07	1.57E-06
AC011287.2	2.848196	-3.41633	5.91E-07	1.60E-06
AC112495.1	5.271813	-2.50653	5.95E-07	1.61E-06
CASR	2.771041	0.922912	5.96E-07	1.62E-06
AC073347.1	4.046406	-3.34951	6.02E-07	1.63E-06
AC244107.1	3.997877	-3.424	6.04E-07	1.63E-06
ANKRD30BP3	2.700144	-3.29259	6.07E-07	1.64E-06
HRH3	3.923626	-2.09752	6.07E-07	1.64E-06
TUBBP6	2.10238	-3.40677	6.07E-07	1.64E-06
DUTP2	2.010945	-3.32732	6.11E-07	1.65E-06
AC092685.1	2.342651	-3.94405	6.26E-07	1.69E-06
INSL6	2.929256	-1.78512	6.28E-07	1.70E-06
RPS16P8	3.311221	-3.46694	6.36E-07	1.72E-06
LGALS13	4.267605	-3.64322	6.39E-07	1.73E-06
RNA5-8SP6	4.775586	-1.867	6.46E-07	1.74E-06
AC109830.1	4.126437	-3.56792	6.48E-07	1.75E-06
AC022540.1	2.043681	-2.2497	6.51E-07	1.76E-06
RN7SL786P	3.924424	-3.81312	6.53E-07	1.76E-06
RN7SL732P	3.50935	-3.09078	6.56E-07	1.77E-06
DMRTB1	3.330601	-4.05307	6.63E-07	1.79E-06
DRGX	2.160044	1.207446	6.68E-07	1.80E-06
AC023824.5	5.182152	-2.89054	6.73E-07	1.81E-06
SNRPEP10	3.614596	-3.05059	6.75E-07	1.82E-06
CACNG7	3.127367	-3.32734	6.85E-07	1.84E-06
OTOR	2.889204	-3.54863	6.86E-07	1.84E-06
AC073651.1	3.364067	-2.27282	6.86E-07	1.85E-06
AL589655.1	4.704215	-3.00127	6.94E-07	1.86E-06
RN7SL359P	3.558817	-3.16713	6.98E-07	1.87E-06
CST11	2.593822	-4.06122	7.02E-07	1.88E-06
EMX2	3.734752	-0.95527	7.09E-07	1.90E-06
C21orf91-OT1	2.341402	-3.23226	7.15E-07	1.92E-06
AC126323.4	2.817328	-3.98723	7.17E-07	1.92E-06
AC092139.2	3.959441	-1.9187	7.20E-07	1.93E-06

For Peer Review

CNBD1	2.915588	-3.87173	7.25E-07	1.95E-06
IGF2	2.158021	7.457535	7.26E-07	1.95E-06
AC093844.1	2.824294	-3.51682	7.28E-07	1.95E-06
SNORA37	3.452904	-2.20481	7.31E-07	1.96E-06
ST7-AS2	2.425561	-1.05303	7.33E-07	1.96E-06
AC099811.4	2.237607	-2.89926	7.34E-07	1.97E-06
PRLHR	3.957842	-1.84636	7.36E-07	1.97E-06
AC013724.1	2.998871	-3.91956	7.39E-07	1.98E-06
AL136131.2	2.696631	-3.57392	7.41E-07	1.98E-06
AL162578.1	2.746574	-2.7707	7.54E-07	2.02E-06
AC006148.1	3.050412	-3.26219	7.55E-07	2.02E-06
HHLA1	3.430392	-3.27634	7.70E-07	2.06E-06
LINC01885	3.232075	-4.08451	7.75E-07	2.07E-06
AL049775.2	3.572893	-3.60262	7.79E-07	2.08E-06
RPS7P2	2.089177	-3.9116	7.81E-07	2.08E-06
AC023813.2	4.082631	-2.87108	7.86E-07	2.10E-06
AC007161.1	2.409781	-2.63253	7.93E-07	2.12E-06
POLD2P1	2.202684	-3.48737	7.96E-07	2.12E-06
AP000688.2	2.633996	-0.90989	7.98E-07	2.13E-06
NF1P8	3.980441	-3.65	8.00E-07	2.13E-06
KLK15	3.232721	-3.57441	8.02E-07	2.14E-06
ZNF90P1	2.509543	-2.86224	8.02E-07	2.14E-06
BHMG1	2.034784	-3.24897	8.12E-07	2.16E-06
FOXI1	3.003131	-0.72789	8.15E-07	2.17E-06
FAM242F	2.229935	-3.72896	8.19E-07	2.18E-06
LUZP4	3.270406	-3.94003	8.24E-07	2.19E-06
AL356320.2	2.881971	-2.82386	8.26E-07	2.20E-06
AC148477.4	3.212328	-2.71057	8.31E-07	2.21E-06
AL162584.1	3.03051	-3.31273	8.34E-07	2.22E-06
AC009032.1	2.169393	-1.83871	8.38E-07	2.23E-06
Z97206.2	2.421456	-4.00174	8.38E-07	2.23E-06
KLK13	2.241669	2.776352	8.42E-07	2.24E-06
AL035401.1	2.335193	-3.24042	8.48E-07	2.25E-06
AC092910.1	2.959888	-2.94338	8.66E-07	2.29E-06
ZBTB20-AS5	3.428476	-1.89129	8.73E-07	2.31E-06
SNORD8	3.138762	-3.2768	8.98E-07	2.38E-06
NPM1P29	2.03133	-2.78482	9.07E-07	2.40E-06
NLRP13	4.054333	-3.02422	9.11E-07	2.41E-06
AP000619.3	2.659329	-4.00821	9.12E-07	2.41E-06
HNRNPRP1	2.336354	-2.72772	9.16E-07	2.42E-06
PHACTR2-AS1	2.898572	-2.54685	9.16E-07	2.42E-06
AC104984.2	2.07976	-3.57255	9.19E-07	2.43E-06
AC012186.3	2.077462	-3.92039	9.24E-07	2.44E-06

For Peer Review

LINC01444	3.559693	-2.77958	9.38E-07	2.47E-06
HHLA2	2.237673	4.917264	9.40E-07	2.48E-06
RNA5SP282	2.231722	-3.45312	9.41E-07	2.48E-06
AC092325.1	3.482002	-4.00372	9.42E-07	2.49E-06
AC004672.1	2.330663	-3.16644	9.51E-07	2.51E-06
IGKV1D-27	2.038602	0.225069	9.54E-07	2.51E-06
RFPL4B	3.318828	-3.12621	9.60E-07	2.53E-06
FLJ36000	3.951952	-3.34653	9.65E-07	2.54E-06
DPYD-AS2	3.577936	-2.29349	9.69E-07	2.55E-06
MIR3907	2.179081	-4.05787	9.72E-07	2.56E-06
GABRG2	4.150982	-1.14357	9.72E-07	2.56E-06
MTCO1P19	3.322502	-3.67811	9.74E-07	2.56E-06
RN7SL356P	2.611185	-3.91731	9.75E-07	2.56E-06
AC008063.2	2.222398	-3.55564	9.81E-07	2.58E-06
AC108174.1	2.245749	-3.69794	9.88E-07	2.60E-06
AC018797.3	2.153878	-2.2609	9.97E-07	2.62E-06
AC019278.1	4.156338	-3.70568	1.01E-06	2.65E-06
AC074237.1	3.403922	-3.93496	1.02E-06	2.66E-06
RN7SL513P	2.365972	-3.88937	1.02E-06	2.67E-06
Z84478.1	2.760888	-2.68675	1.02E-06	2.68E-06
GPRC6A	3.201848	-2.82745	1.03E-06	2.70E-06
PCK1	3.422201	1.889483	1.03E-06	2.71E-06
RN7SL635P	3.161515	-3.5631	1.04E-06	2.73E-06
AC107419.1	2.73067	-3.22254	1.05E-06	2.74E-06
AC016868.1	3.231273	-3.65132	1.05E-06	2.75E-06
AL356311.1	2.983951	-3.95918	1.05E-06	2.76E-06
IFITM5	2.248803	-1.752	1.07E-06	2.79E-06
HDGFL1	3.820068	-3.4943	1.07E-06	2.79E-06
RN7SL769P	4.566436	-2.85163	1.07E-06	2.80E-06
ADH1C	2.075978	4.485806	1.08E-06	2.83E-06
AP000439.2	2.96058	-2.2683	1.09E-06	2.84E-06
AC073174.1	3.994151	-3.31368	1.10E-06	2.87E-06
LINC01697	2.50319	-1.90652	1.10E-06	2.87E-06
NPY	5.018794	2.426024	1.10E-06	2.88E-06
CCDC92B	3.301253	-3.35269	1.11E-06	2.89E-06
KCNK10	2.117964	-0.7102	1.11E-06	2.91E-06
LINC01143	2.504532	-3.08075	1.13E-06	2.95E-06
MTND2P2	4.064822	-3.28469	1.15E-06	2.99E-06
LINC02443	2.589941	-3.86616	1.15E-06	2.99E-06
AC068389.3	3.091188	-3.20182	1.16E-06	3.02E-06
FBXL21	3.42962	-2.79628	1.17E-06	3.04E-06
AC091987.1	3.565594	-3.68338	1.17E-06	3.04E-06
AP000851.2	2.46272	-2.69328	1.17E-06	3.06E-06

For Peer Review

AC091133.2	3.569075	-3.88787	1.18E-06	3.07E-06
LINC00705	2.541338	-3.5631	1.18E-06	3.08E-06
TTR	3.331297	1.084436	1.19E-06	3.09E-06
RN7SL363P	2.180232	-4.03483	1.20E-06	3.11E-06
AC099788.1	2.859794	-3.819	1.20E-06	3.12E-06
BCAP31P1	2.023931	-3.17251	1.21E-06	3.14E-06
AC091151.1	3.716907	-3.61215	1.21E-06	3.15E-06
AC097065.2	2.059772	-3.82419	1.22E-06	3.16E-06
CES5AP1	2.208098	-2.77304	1.22E-06	3.17E-06
ELK2AP	2.499774	-4.04476	1.22E-06	3.17E-06
ABCG8	2.6583	-3.12311	1.22E-06	3.18E-06
AL592043.1	3.100853	-3.97801	1.24E-06	3.22E-06
FAM9B	2.615229	-1.05978	1.27E-06	3.30E-06
AC132807.2	3.215208	-2.7938	1.28E-06	3.32E-06
IGHD3-10	2.075308	-3.71885	1.29E-06	3.34E-06
AC113146.1	2.342922	-3.21761	1.30E-06	3.37E-06
AP001025.1	2.292876	-2.84438	1.31E-06	3.38E-06
AC093274.1	2.086155	-3.76046	1.31E-06	3.39E-06
TUBB4BP2	2.131428	-3.49013	1.32E-06	3.41E-06
AL590632.1	3.419419	-3.10267	1.32E-06	3.41E-06
TAC1	4.835849	2.00483	1.32E-06	3.42E-06
OR1J1	2.016763	-3.94668	1.33E-06	3.43E-06
AL358178.1	3.008638	-3.32156	1.33E-06	3.45E-06
SNX31	2.097963	-1.48098	1.34E-06	3.47E-06
LINC00051	2.90514	-3.18504	1.36E-06	3.50E-06
RN7SL736P	3.194761	-2.972	1.37E-06	3.53E-06
AC010974.1	2.16419	-3.78198	1.37E-06	3.53E-06
RAPGEF4-AS1	2.563532	-3.51464	1.37E-06	3.54E-06
RBMXP1	2.433222	-3.86202	1.38E-06	3.56E-06
HNRNPA1P29	4.07644	-3.51562	1.38E-06	3.56E-06
BRINP2	2.075973	-1.06061	1.39E-06	3.58E-06
PMPCAP1	2.890988	-3.66628	1.39E-06	3.59E-06
HBE1	2.917876	-1.07567	1.42E-06	3.67E-06
FAHD2P1	2.515155	-3.0138	1.43E-06	3.68E-06
AC112236.1	2.599534	-3.65365	1.44E-06	3.70E-06
MCHR2	2.793692	-3.64462	1.44E-06	3.71E-06
AC139792.3	3.561254	-3.67934	1.44E-06	3.71E-06
C11orf53	2.626899	0.304513	1.44E-06	3.72E-06
IGKV2D-26	2.318459	-3.3726	1.45E-06	3.73E-06
LINC01854	3.501472	-3.91349	1.45E-06	3.73E-06
AC092436.1	2.203129	-3.97749	1.45E-06	3.74E-06
AC133561.2	2.034737	-3.4039	1.45E-06	3.74E-06
AC005828.1	2.420338	-2.63297	1.46E-06	3.75E-06

For Peer Review

MUCL3	2.572294	6.197287	1.47E-06	3.78E-06
AL031258.1	2.267353	-2.88348	1.48E-06	3.79E-06
SLURP1	2.188772	-3.6414	1.48E-06	3.80E-06
RPL31P58	2.389663	-3.22513	1.48E-06	3.81E-06
AC008443.2	2.226975	-2.75572	1.49E-06	3.82E-06
CYP1A1	-2.00545	1.963137	1.49E-06	3.83E-06
PSG7	4.198152	-3.52717	1.49E-06	3.84E-06
AC025211.1	2.6889	-3.54187	1.50E-06	3.84E-06
AL513485.1	2.214999	-3.71859	1.50E-06	3.85E-06
IGLV7-35	2.062613	-3.34189	1.50E-06	3.86E-06
RN7SL300P	3.687897	-3.037	1.51E-06	3.87E-06
AC103853.1	3.058533	-3.52947	1.51E-06	3.87E-06
BRWD1P3	3.092728	-3.32026	1.52E-06	3.90E-06
AC117465.1	2.948072	-3.70429	1.54E-06	3.95E-06
AC008456.1	2.463955	-4.04271	1.54E-06	3.95E-06
NBPF4	2.999383	-2.85584	1.54E-06	3.96E-06
AC090774.2	3.033516	-3.49986	1.54E-06	3.96E-06
OR1H1P	2.002264	-3.62135	1.55E-06	3.96E-06
SRMP2	2.255168	-3.1802	1.55E-06	3.98E-06
AC018692.3	3.456709	-4.03026	1.56E-06	3.98E-06
AC020551.1	3.485061	-4.00229	1.59E-06	4.06E-06
AL731563.1	2.304684	-3.36578	1.59E-06	4.07E-06
AL356364.1	4.223903	-3.58888	1.59E-06	4.08E-06
LINC02505	3.401486	-3.41718	1.60E-06	4.09E-06
AC046158.1	2.719007	-3.06079	1.61E-06	4.12E-06
AL049830.4	2.700973	-2.67387	1.62E-06	4.13E-06
RN7SL398P	3.745174	-3.32127	1.63E-06	4.16E-06
KRT17P2	2.007635	-4.00159	1.64E-06	4.19E-06
AC064872.1	3.875025	-3.84077	1.65E-06	4.21E-06
CKS1BP2	2.233569	-3.77338	1.66E-06	4.24E-06
OPRM1	2.274137	-3.94286	1.68E-06	4.29E-06
MIR1205	3.128849	-3.83371	1.69E-06	4.31E-06
IDH2-DT	2.470911	-3.25016	1.71E-06	4.35E-06
BX324167.1	2.05375	-3.02651	1.73E-06	4.42E-06
FGF12-AS3	2.392761	-3.80298	1.75E-06	4.45E-06
IGF2-AS	2.539741	-2.1069	1.76E-06	4.48E-06
GSG1L2	3.990667	-3.41052	1.77E-06	4.50E-06
RN7SL329P	2.798138	-2.84468	1.77E-06	4.50E-06
OTX2-AS1	3.012487	-3.30354	1.78E-06	4.53E-06
RNU6-522P	3.668252	-3.93032	1.79E-06	4.55E-06
RN7SKP189	4.529877	-3.35081	1.79E-06	4.55E-06
AC007001.1	2.723964	-3.0219	1.79E-06	4.56E-06
PAQR9-AS1	3.572347	-2.97105	1.80E-06	4.57E-06

For Peer Review

AC110813.1	3.754893	-2.11902	1.82E-06	4.61E-06
AC004865.1	3.283238	-2.97147	1.82E-06	4.63E-06
RNU5F-1	2.022124	-2.4544	1.83E-06	4.63E-06
AL022323.2	2.587871	-2.37904	1.83E-06	4.64E-06
AC009948.5	3.136796	-2.08388	1.83E-06	4.65E-06
UCN3	2.565804	3.450904	1.84E-06	4.66E-06
AL512638.1	3.220848	-3.98181	1.84E-06	4.66E-06
IFNL4	2.631511	-3.51478	1.84E-06	4.66E-06
SCG3	2.203865	1.825214	1.84E-06	4.67E-06
AL360091.2	3.071056	-3.19524	1.85E-06	4.68E-06
CACNA1C-IT3	3.919971	-3.71883	1.88E-06	4.78E-06
OR51B5	2.517147	-1.6936	1.89E-06	4.80E-06
AC006262.2	2.354827	-4.01085	1.90E-06	4.80E-06
AL139120.1	3.902407	-2.94242	1.90E-06	4.82E-06
SCAND3P1	2.550456	-3.95201	1.91E-06	4.84E-06
RN7SL760P	2.898049	-3.40755	1.92E-06	4.86E-06
RN7SL466P	3.724783	-3.90552	1.92E-06	4.86E-06
AC072039.1	2.513754	-4.00391	1.92E-06	4.86E-06
DDX18P5	2.73648	-2.98777	1.92E-06	4.86E-06
TCEAL3-AS1	2.025294	-3.44243	1.94E-06	4.90E-06
AP000997.1	2.504714	-4.09216	1.94E-06	4.91E-06
AP000756.2	3.248459	-3.76115	1.95E-06	4.94E-06
RPL23AP11	2.365889	-3.33652	1.97E-06	4.98E-06
AC127029.2	2.865143	-3.61098	1.99E-06	5.03E-06
RN7SL643P	3.707407	-3.91075	2.01E-06	5.07E-06
RN7SL191P	3.74521	-3.89306	2.01E-06	5.08E-06
AC005722.2	2.12694	-1.39031	2.02E-06	5.10E-06
SNORA11B	2.940237	-3.87307	2.03E-06	5.12E-06
ESX1	3.77265	-3.69147	2.04E-06	5.14E-06
ZBTB46-AS1	2.551233	-3.15394	2.04E-06	5.15E-06
AC026117.1	3.610388	-3.86989	2.05E-06	5.16E-06
AL008638.2	4.444449	-3.44025	2.05E-06	5.17E-06
AC105917.1	3.355214	-3.30336	2.07E-06	5.21E-06
OSBPL10-AS1	2.284288	-2.66342	2.08E-06	5.25E-06
MTND4P20	2.324614	-1.45849	2.08E-06	5.25E-06
AC022973.1	2.598848	-2.99446	2.09E-06	5.26E-06
AC024405.2	3.734061	-3.86735	2.09E-06	5.27E-06
AC106053.1	2.394905	-3.95099	2.11E-06	5.31E-06
SOX1	3.690274	-2.05677	2.13E-06	5.35E-06
SERPINB7	2.439159	0.829826	2.13E-06	5.36E-06
SSX3	4.144072	-3.56013	2.16E-06	5.42E-06
AP000641.1	3.27586	-4.00962	2.20E-06	5.51E-06
AL627308.1	2.868944	-2.85228	2.20E-06	5.52E-06

MIR196A1	2.755478	-4.01075	2.21E-06	5.53E-06
AC026320.1	3.164661	-4.09861	2.21E-06	5.54E-06
LINC01819	2.557599	-0.41669	2.21E-06	5.55E-06
RN7SL273P	3.385368	-3.47088	2.25E-06	5.63E-06
ASB11	2.069399	-0.75494	2.25E-06	5.63E-06
AC015818.2	2.085056	-4.04415	2.25E-06	5.64E-06
AC092120.2	2.702618	-2.82112	2.26E-06	5.65E-06
PKLR	2.077891	-3.19299	2.26E-06	5.65E-06
AC007533.1	2.694342	-3.11595	2.28E-06	5.71E-06
AL391863.1	3.227374	-2.89507	2.28E-06	5.72E-06
UGT1A2P	2.731025	-3.86015	2.29E-06	5.73E-06
AC105460.1	4.392356	-3.02229	2.37E-06	5.93E-06
LINC00456	2.940698	-3.96376	2.38E-06	5.95E-06
AC006262.4	2.555295	-3.4813	2.38E-06	5.95E-06
AP003419.1	2.225	-3.8854	2.39E-06	5.97E-06
TPH2	2.423207	-3.10275	2.40E-06	5.99E-06
GBX1	2.41737	-2.83757	2.40E-06	6.00E-06
AC074389.2	3.436562	-3.08992	2.41E-06	6.01E-06
AC237221.2	2.93456	-3.39555	2.41E-06	6.02E-06
AC139718.1	3.103607	-3.92952	2.42E-06	6.05E-06
AC068305.1	2.234805	-3.60397	2.46E-06	6.13E-06
GALNTL5	2.493589	-4.00893	2.48E-06	6.18E-06
EDDM3CP	3.687505	-3.91955	2.48E-06	6.19E-06
DYNAP	2.685168	-3.93534	2.50E-06	6.23E-06
RN7SKP180	2.537987	-2.85374	2.51E-06	6.26E-06
XIAPP2	2.44827	-3.03501	2.53E-06	6.29E-06
AL353662.3	2.006464	-3.64024	2.54E-06	6.34E-06
LINC02266	2.804251	-2.22136	2.55E-06	6.35E-06
AP000904.1	2.203268	-2.41276	2.56E-06	6.37E-06
AC008937.2	2.289935	-2.86792	2.56E-06	6.38E-06
AL355483.3	2.373956	-3.82614	2.56E-06	6.38E-06
AC109446.4	2.225092	-3.68511	2.58E-06	6.41E-06
RNU2-61P	3.937281	-3.80813	2.58E-06	6.42E-06
CBX3P5	2.078286	-3.51793	2.59E-06	6.43E-06
AF254983.1	3.774938	-3.72302	2.59E-06	6.45E-06
AC108688.1	2.878586	-3.03829	2.60E-06	6.45E-06
ATCAY	2.316063	-2.49086	2.64E-06	6.55E-06
FER1L6-AS1	4.135796	-3.03634	2.66E-06	6.59E-06
ZNF729	3.504644	-3.12798	2.66E-06	6.61E-06
AC090193.1	2.471068	-4.0592	2.68E-06	6.65E-06
AC139792.2	2.397732	-3.55159	2.69E-06	6.68E-06
AC012065.1	3.452719	-4.00015	2.69E-06	6.69E-06
SCARNA18	2.796122	-3.29609	2.72E-06	6.75E-06

For Peer Review

PDSS1P1	2.751509	-2.95283	2.73E-06	6.78E-06
RN7SL836P	2.689733	-3.01106	2.73E-06	6.78E-06
CCKAR	3.375895	-2.61123	2.74E-06	6.78E-06
RN7SL280P	2.029734	-3.34577	2.75E-06	6.82E-06
MED15P9	3.708743	-3.26028	2.76E-06	6.83E-06
FAM25A	2.943097	-3.65277	2.80E-06	6.94E-06
TIMM9P1	3.57403	-3.25808	2.80E-06	6.94E-06
AC079416.3	3.067952	-3.76408	2.82E-06	6.98E-06
PCAT18	2.390195	-1.75316	2.82E-06	6.99E-06
ACNATP	2.654709	-3.7556	2.83E-06	7.01E-06
AC104232.2	3.678829	-3.3467	2.85E-06	7.04E-06
AP005131.1	2.019567	-0.79684	2.86E-06	7.06E-06
AC116096.1	2.664475	-3.77297	2.86E-06	7.07E-06
SNRPGP18	4.086169	-2.85375	2.87E-06	7.09E-06
AL031668.1	2.564429	-3.01027	2.88E-06	7.12E-06
CLPS	2.657485	-2.87595	2.92E-06	7.21E-06
RNU6-681P	4.106429	-3.40413	2.93E-06	7.23E-06
AC084783.1	3.250439	-3.20482	2.94E-06	7.25E-06
RPL7P49	2.089316	-3.63854	2.94E-06	7.26E-06
LINC01886	4.025279	-2.33635	2.96E-06	7.29E-06
AC008781.2	2.885424	-1.45863	2.97E-06	7.32E-06
11-Mar	4.273024	-1.77221	3.01E-06	7.42E-06
AC073551.1	2.725495	-3.61996	3.04E-06	7.50E-06
AL359551.1	2.41789	-3.5393	3.06E-06	7.53E-06
HAO1	3.258927	-2.72302	3.06E-06	7.54E-06
AC013391.3	2.668291	-4.03911	3.08E-06	7.59E-06
FRG2C	2.154761	-3.33203	3.08E-06	7.59E-06
AL031767.1	4.277667	-3.21347	3.09E-06	7.60E-06
AC087612.1	3.022246	-3.05735	3.15E-06	7.75E-06
AP001207.2	2.502725	-3.72164	3.17E-06	7.78E-06
AC009313.2	3.234063	-3.83369	3.18E-06	7.82E-06
TAF9BP1	2.333752	-3.18794	3.18E-06	7.82E-06
C10orf113	2.635775	-2.96585	3.18E-06	7.83E-06
LINC02303	3.703798	-2.62123	3.20E-06	7.88E-06
LYPD4	2.88673	-3.47941	3.21E-06	7.90E-06
SPRR2A	3.327053	0.372495	3.22E-06	7.90E-06
AC006270.3	3.974564	-3.25169	3.22E-06	7.92E-06
EIF1AXP2	3.809885	-3.61853	3.23E-06	7.92E-06
AC124069.1	2.482911	-2.46523	3.26E-06	7.99E-06
AC024451.1	2.289998	-2.794	3.28E-06	8.04E-06
AL034349.1	3.545806	-2.969	3.28E-06	8.04E-06
RN7SL230P	2.605248	-3.06023	3.34E-06	8.18E-06
ASTN2-AS1	3.132477	-3.34857	3.34E-06	8.19E-06

For Peer Review

RNU1-89P	3.819531	-3.85616	3.36E-06	8.23E-06
RN7SL791P	3.834598	-3.77424	3.37E-06	8.26E-06
RNU1-11P	4.702718	-2.1287	3.39E-06	8.30E-06
LINC02260	2.670576	-3.83373	3.48E-06	8.50E-06
PSG2	4.17643	-3.01743	3.48E-06	8.51E-06
AL627308.2	3.290993	-3.49758	3.49E-06	8.53E-06
SNRPGP3	2.045613	-3.99374	3.50E-06	8.55E-06
AC093001.1	4.051116	-1.24264	3.50E-06	8.55E-06
LINC02527	3.23617	-3.32786	3.52E-06	8.60E-06
PCNPP3	2.530637	-3.56383	3.53E-06	8.62E-06
AC069431.2	2.95304	-2.60868	3.55E-06	8.67E-06
SPATA31C2	2.276823	-3.78747	3.62E-06	8.83E-06
AC055876.1	2.68506	-3.75507	3.65E-06	8.89E-06
PNLIPRP3	3.254237	-2.11969	3.65E-06	8.91E-06
FOXN4	2.292714	-0.73935	3.66E-06	8.92E-06
SERPINA11	2.891138	-2.2332	3.66E-06	8.93E-06
LINC00609	2.281057	-3.12703	3.67E-06	8.95E-06
LINC01593	2.337232	-3.94645	3.68E-06	8.97E-06
AC027018.1	2.442185	-2.71844	3.73E-06	9.08E-06
OR1J4	2.05863	-3.78573	3.74E-06	9.10E-06
GPR26	3.494079	-1.01591	3.74E-06	9.11E-06
LINC02312	2.974658	-3.72775	3.78E-06	9.20E-06
ILF2P1	2.016022	-3.25488	3.81E-06	9.26E-06
CDH12	2.472541	-0.80589	3.84E-06	9.34E-06
ANXA13	2.143186	0.940489	3.88E-06	9.44E-06
CYMP	2.816953	-2.00327	3.90E-06	9.48E-06
RN7SL7P	3.776034	-3.64536	3.92E-06	9.53E-06
CYMP-AS1	2.918163	-2.17815	3.93E-06	9.54E-06
AC034229.1	2.013491	-3.98002	3.97E-06	9.65E-06
IL36A	2.473767	-4.03783	4.02E-06	9.74E-06
RAD51AP1P1	3.965411	-3.20236	4.02E-06	9.75E-06
AC027279.4	3.318598	-2.82452	4.04E-06	9.79E-06
AC114812.3	2.932139	-4.03664	4.07E-06	9.87E-06
AC021818.1	2.120509	-3.86067	4.08E-06	9.88E-06
AC007496.3	2.315158	-1.1333	4.08E-06	9.88E-06
AL159987.1	4.404397	-2.5814	4.09E-06	9.92E-06
AL583808.1	2.958823	-2.75777	4.14E-06	1.00E-05
RNU6-122P	2.796965	-3.53164	4.14E-06	1.00E-05
HMGB3P18	3.751536	-3.88706	4.15E-06	1.00E-05
AC012568.1	3.467554	-3.2139	4.17E-06	1.01E-05
AL357054.1	3.043498	-3.7062	4.18E-06	1.01E-05
NDUFA8P1	3.47403	-3.48942	4.20E-06	1.02E-05
AC092800.1	2.709991	-3.68336	4.21E-06	1.02E-05

For Peer Review

RN7SKP90	3.686192	-3.43891	4.29E-06	1.04E-05
AL133372.2	3.095207	-3.71884	4.31E-06	1.04E-05
C8orf17	2.443767	-3.24394	4.32E-06	1.05E-05
RN7SL126P	3.345962	-3.65866	4.34E-06	1.05E-05
MAGEA2	2.939034	-4.10677	4.35E-06	1.05E-05
LINC00879	4.262947	-3.43478	4.35E-06	1.05E-05
AC004080.2	3.07141	-3.93457	4.40E-06	1.06E-05
AC148477.2	2.334335	-2.76846	4.41E-06	1.07E-05
AC005186.1	3.545153	-3.71426	4.41E-06	1.07E-05
AL139327.1	4.957126	-2.03961	4.44E-06	1.07E-05
MRGPRG-AS1	3.271076	-3.87432	4.45E-06	1.07E-05
AC067940.1	2.309714	-3.6941	4.46E-06	1.07E-05
AC108159.1	2.306039	-3.5379	4.53E-06	1.09E-05
ADAM7	2.706298	-4.04402	4.61E-06	1.11E-05
AC005176.1	3.084728	-3.79769	4.65E-06	1.12E-05
KRTAP29-1	2.292994	-3.65758	4.66E-06	1.12E-05
LINC01250	2.228472	-3.81771	4.68E-06	1.13E-05
LINC01087	2.746037	-3.40816	4.69E-06	1.13E-05
AC093730.1	3.497946	-3.6819	4.70E-06	1.13E-05
YBX1P7	3.634819	-3.41261	4.74E-06	1.14E-05
AC063926.3	2.093855	-3.20046	4.75E-06	1.14E-05
AL645608.3	2.06676	-3.53031	4.81E-06	1.16E-05
AC008060.1	3.378143	-3.91277	4.82E-06	1.16E-05
LINC01665	3.269955	-3.72415	4.83E-06	1.16E-05
SUMO2P8	3.095981	-3.22915	4.84E-06	1.16E-05
AC022022.2	3.733044	-2.94042	4.85E-06	1.16E-05
LINC02300	2.413336	-1.06571	4.86E-06	1.17E-05
NFIA-AS1	2.270646	-2.47428	4.88E-06	1.17E-05
AC096577.1	2.606785	-3.36744	4.90E-06	1.17E-05
AC025575.1	2.550204	-4.00938	4.91E-06	1.18E-05
AC008680.1	2.203135	-4.0301	4.91E-06	1.18E-05
AL138974.1	3.499849	-3.43137	4.92E-06	1.18E-05
AC114878.2	2.77427	-2.49608	4.92E-06	1.18E-05
AL121772.2	2.131846	-3.8089	4.93E-06	1.18E-05
PCSK2	3.008546	6.364929	4.95E-06	1.19E-05
AQP12B	3.209097	-3.80554	4.98E-06	1.19E-05
RN7SL711P	2.870356	-3.32054	4.98E-06	1.19E-05
AC087821.1	2.330261	-3.54544	4.99E-06	1.19E-05
RN7SL464P	3.940352	-3.57884	5.00E-06	1.20E-05
PAX3	2.493164	-2.90051	5.01E-06	1.20E-05
MAGEB17	2.221763	-2.08528	5.04E-06	1.21E-05
RN7SL801P	3.450832	-3.77808	5.06E-06	1.21E-05
UMOD	-2.34458	-2.53285	5.07E-06	1.21E-05

AC010609.1	2.70522	-3.71527	5.07E-06	1.21E-05
SRARP	2.56628	-1.20605	5.08E-06	1.22E-05
AC002075.1	3.06959	-3.90531	5.10E-06	1.22E-05
RN7SL659P	3.307446	-3.13031	5.10E-06	1.22E-05
AF131216.3	2.483799	-2.97843	5.11E-06	1.22E-05
LINC01433	2.158483	-2.62416	5.11E-06	1.22E-05
AC104984.6	2.399486	-2.9883	5.15E-06	1.23E-05
AC108102.1	2.759487	-3.14564	5.16E-06	1.23E-05
AC073569.1	2.483402	-2.65687	5.24E-06	1.25E-05
AL353803.2	2.116304	-2.77303	5.25E-06	1.25E-05
AC010997.5	3.486501	-1.84896	5.30E-06	1.26E-05
LINC01744	2.451159	-4.03532	5.32E-06	1.27E-05
AC108865.2	2.445146	-4.03348	5.34E-06	1.27E-05
AC013444.1	2.022195	-2.2917	5.38E-06	1.28E-05
FTLP6	2.089627	-3.69975	5.39E-06	1.29E-05
HMGB1P7	2.945069	-3.70928	5.46E-06	1.30E-05
LINC02385	3.471582	-2.74057	5.48E-06	1.31E-05
NIP7P3	2.784487	-3.36152	5.55E-06	1.32E-05
AL390318.1	4.806846	-2.35714	5.56E-06	1.32E-05
AC023824.1	4.315826	-2.28105	5.57E-06	1.33E-05
KCNJ4	2.078876	-3.47675	5.59E-06	1.33E-05
AC087258.1	2.83803	-3.71946	5.60E-06	1.33E-05
AC007993.2	3.08276	-2.78771	5.61E-06	1.33E-05
AC092803.1	2.214034	-3.87494	5.65E-06	1.34E-05
RN7SL14P	3.572795	-3.72364	5.79E-06	1.38E-05
TAS2R64P	2.154609	-2.05578	5.92E-06	1.40E-05
RN7SKP237	2.682468	-3.45788	5.93E-06	1.41E-05
RF00002	4.535706	-3.01065	5.93E-06	1.41E-05
KRT40	2.194486	0.61594	5.98E-06	1.42E-05
RPL23AP30	2.471171	-3.41425	6.00E-06	1.42E-05
AC010099.4	2.519457	-2.98692	6.00E-06	1.42E-05
ACOT12	2.494776	-3.55358	6.01E-06	1.43E-05
AC022973.3	2.100465	-2.18721	6.03E-06	1.43E-05
CLYBL-AS2	2.073995	-3.96062	6.04E-06	1.43E-05
LINC02522	3.316146	-3.47256	6.07E-06	1.44E-05
CYP2C19	2.302358	-3.5333	6.08E-06	1.44E-05
AC104332.1	3.644512	-2.51372	6.09E-06	1.44E-05
CT55	2.326185	-3.08505	6.11E-06	1.45E-05
AP005131.4	2.631034	-2.25687	6.12E-06	1.45E-05
AL513324.1	2.871193	-4.07347	6.15E-06	1.46E-05
ANKRD30BL	2.521699	-3.79263	6.17E-06	1.46E-05
RN7SL37P	2.358214	-3.79222	6.19E-06	1.46E-05
AC027088.4	2.271087	-3.82154	6.25E-06	1.48E-05

For Peer Review

AL513164.1	2.407984	-3.72504	6.34E-06	1.50E-05
RN7SL30P	2.791645	-3.88178	6.40E-06	1.51E-05
AC135626.1	3.308423	-3.59494	6.42E-06	1.52E-05
MIR7-3HG	3.124604	-2.4277	6.50E-06	1.53E-05
AL079304.1	3.251189	-3.85857	6.54E-06	1.54E-05
AP001547.1	2.485671	-3.79946	6.56E-06	1.55E-05
LINC01450	2.107647	-3.88496	6.63E-06	1.56E-05
NBPF6	2.773524	-3.54034	6.65E-06	1.57E-05
AC040963.1	2.943054	-3.74225	6.65E-06	1.57E-05
AC104982.1	2.774423	-3.20125	6.66E-06	1.57E-05
SVOP	2.231757	-2.17977	6.82E-06	1.61E-05
LINC02348	2.18286	-3.77843	6.85E-06	1.61E-05
AC106873.1	2.891944	-2.77633	6.91E-06	1.62E-05
LINC02470	2.973614	-2.47695	6.97E-06	1.64E-05
LINC02078	2.068197	-2.77115	7.00E-06	1.65E-05
LINC00867	2.176313	-3.36238	7.04E-06	1.65E-05
TDRG1	2.720308	-3.90736	7.05E-06	1.66E-05
MDGA2	2.871618	-1.612	7.09E-06	1.66E-05
CDC20B	2.033217	1.402234	7.12E-06	1.67E-05
AC025183.1	3.290608	-3.89537	7.18E-06	1.68E-05
BTBD17	2.5719	-2.93662	7.18E-06	1.68E-05
PNMA6F	3.494673	-3.40177	7.24E-06	1.70E-05
SNHG27	3.280808	-3.26643	7.27E-06	1.70E-05
AC126564.1	2.123688	-1.26805	7.27E-06	1.70E-05
RNASE7	2.027358	-1.7138	7.30E-06	1.71E-05
MIR133A1HG	2.187452	-1.33925	7.31E-06	1.71E-05
AL118556.2	3.464232	-3.38237	7.32E-06	1.72E-05
GPR12	2.350126	-1.08732	7.32E-06	1.72E-05
SCGB2A2	3.910634	-3.42566	7.33E-06	1.72E-05
SCARNA11	2.854248	-3.88867	7.35E-06	1.72E-05
LINC01448	3.103332	-3.98813	7.37E-06	1.73E-05
AC130895.1	3.443062	-2.57334	7.41E-06	1.74E-05
AP002833.2	3.043796	-3.92006	7.50E-06	1.75E-05
AC114912.1	2.783369	-4.07375	7.50E-06	1.76E-05
AL157402.2	3.820761	-2.38771	7.51E-06	1.76E-05
KRT9	2.672129	-3.62374	7.53E-06	1.76E-05
CTSL3P	3.194699	-3.90733	7.54E-06	1.76E-05
AF106564.1	2.216847	-2.77989	7.63E-06	1.78E-05
AC022973.2	2.037167	-2.47268	7.65E-06	1.79E-05
AC008953.1	2.498015	-3.01404	7.69E-06	1.80E-05
TRIM53AP	2.923207	-3.95853	7.70E-06	1.80E-05
CBX5P1	2.505568	-3.3773	7.73E-06	1.81E-05
C18orf15	2.538147	-3.23553	7.74E-06	1.81E-05

For Peer Review

AC007368.2	2.526997	-3.90056	7.76E-06	1.81E-05
AL132709.1	3.387442	-3.85337	7.78E-06	1.82E-05
ZNF679	3.619349	-3.50629	7.94E-06	1.85E-05
AP000919.3	2.543344	-2.52383	8.01E-06	1.87E-05
LINC01322	2.793571	-2.17691	8.01E-06	1.87E-05
DIMT1P1	3.742509	-3.88327	8.02E-06	1.87E-05
AL365258.1	3.479172	-3.51573	8.02E-06	1.87E-05
AK3P5	2.044368	-2.35716	8.03E-06	1.87E-05
AL445223.1	3.046867	-2.55435	8.05E-06	1.87E-05
AC017048.3	2.076688	-2.99287	8.07E-06	1.88E-05
DUTP7	2.872688	-3.73603	8.08E-06	1.88E-05
AC034223.1	2.770783	-3.91431	8.08E-06	1.88E-05
AL161938.1	3.026001	-3.92093	8.11E-06	1.89E-05
AL353746.1	2.149903	-2.49277	8.12E-06	1.89E-05
AC009686.1	2.760437	-3.60276	8.14E-06	1.89E-05
MAPK6P3	2.071249	-2.24328	8.16E-06	1.90E-05
AL078601.2	2.169035	-2.10196	8.18E-06	1.90E-05
AC007448.2	2.836879	-3.52273	8.22E-06	1.91E-05
AC018541.1	4.521889	-3.30015	8.22E-06	1.91E-05
LARP1BP1	3.57141	-3.62626	8.27E-06	1.92E-05
RNU7-84P	2.916422	-3.62981	8.29E-06	1.93E-05
PPP1R2P10	3.835871	-3.24677	8.51E-06	1.98E-05
AC006141.1	2.239667	-2.63486	8.55E-06	1.99E-05
TMPRSS11F	2.398756	-3.78996	8.56E-06	1.99E-05
AC096669.1	3.094927	-4.04833	8.57E-06	1.99E-05
AL133343.2	2.053752	-3.67718	8.58E-06	1.99E-05
AC023481.1	2.174071	-3.88208	8.63E-06	2.00E-05
CACYBPP1	2.339967	-3.16786	8.68E-06	2.01E-05
SPATA31D1	3.092964	-4.04283	8.70E-06	2.02E-05
RPS20P15	2.608523	-3.01453	8.71E-06	2.02E-05
RNA5SP111	2.030412	-3.42076	8.73E-06	2.02E-05
RPS27AP8	2.969725	-3.02735	8.76E-06	2.03E-05
MTOR-AS1	2.628076	-3.56854	8.77E-06	2.03E-05
AC002463.1	3.250214	-3.77338	8.78E-06	2.04E-05
AL390774.2	2.929909	-3.44077	8.80E-06	2.04E-05
LINC02046	3.376137	-3.56762	8.85E-06	2.05E-05
AL512604.1	2.792334	-3.39863	8.95E-06	2.07E-05
RN7SL709P	2.79388	-3.88777	8.97E-06	2.08E-05
AC090220.1	3.328677	-2.77449	9.00E-06	2.08E-05
XKR7	2.635111	-0.19661	9.02E-06	2.09E-05
CDH7	2.11123	-0.90501	9.05E-06	2.09E-05
AC079866.2	2.763062	-3.47649	9.09E-06	2.10E-05
DRAXINP1	3.580893	-3.8876	9.11E-06	2.11E-05

For Peer Review

AC068535.1	3.758697	-3.91034	9.15E-06	2.11E-05
CTNNA2	2.068588	-1.01291	9.17E-06	2.12E-05
SIX6	2.239345	-3.81921	9.18E-06	2.12E-05
AL163193.1	2.686283	-3.3469	9.24E-06	2.13E-05
AC093702.1	2.08491	-3.24464	9.26E-06	2.14E-05
RN7SKP186	4.301496	-3.362	9.29E-06	2.15E-05
TSPY1	3.683779	-3.87058	9.31E-06	2.15E-05
AC115220.1	3.680751	-3.39651	9.43E-06	2.18E-05
GAPDHP49	2.504201	-2.79615	9.47E-06	2.18E-05
AC110921.1	2.817299	-3.68869	9.56E-06	2.21E-05
AC110769.3	2.742091	-2.75907	9.60E-06	2.21E-05
CCNYL2	2.296582	-1.51223	9.60E-06	2.21E-05
ZBTB8OSP1	2.995303	-3.68641	9.60E-06	2.21E-05
AL512324.1	2.565043	-4.03017	9.65E-06	2.22E-05
LNX1-AS1	2.555809	-3.6303	9.66E-06	2.23E-05
AL138713.1	3.319926	-2.98952	9.77E-06	2.25E-05
ABCB10P1	3.169498	-3.07101	9.91E-06	2.28E-05
AC007599.1	3.356788	-3.70816	9.93E-06	2.28E-05
RN7SL314P	3.918198	-3.68175	9.93E-06	2.28E-05
AC106744.1	3.995812	-3.54162	9.94E-06	2.29E-05
OR4A16	3.562832	-3.92502	9.95E-06	2.29E-05
RAD17P2	3.423602	-2.52455	9.96E-06	2.29E-05
AL731563.2	2.182852	-3.56024	9.98E-06	2.29E-05
AC011483.1	2.532464	-3.88696	1.00E-05	2.30E-05
PAX2	2.833793	-2.40175	1.00E-05	2.31E-05
AC119751.4	2.536883	-3.83499	1.01E-05	2.32E-05
AL034418.1	2.010132	-2.70733	1.01E-05	2.33E-05
HOXB1	2.591847	-2.38466	1.02E-05	2.34E-05
HHATL	2.37721	1.048243	1.02E-05	2.34E-05
AC018442.2	2.944464	-3.73599	1.03E-05	2.36E-05
PABPC1L2B	3.377033	-3.659	1.03E-05	2.37E-05
LINC02315	2.102802	-0.71386	1.04E-05	2.37E-05
OPRK1	2.026374	0.407894	1.04E-05	2.39E-05
BNIP3P8	2.762036	-3.72785	1.04E-05	2.39E-05
DEFA9P	4.909941	-1.6415	1.05E-05	2.40E-05
ZP2	2.585011	-0.42033	1.05E-05	2.40E-05
AC104651.1	2.312566	-3.39459	1.05E-05	2.40E-05
MIR1245A	3.102842	-3.98429	1.05E-05	2.40E-05
FAM238B	2.53663	-3.5144	1.05E-05	2.40E-05
LPP-AS1	3.518971	-2.90062	1.05E-05	2.41E-05
RN7SL480P	3.814906	-2.93656	1.06E-05	2.41E-05
EEF1B2P8	3.144311	-3.78584	1.06E-05	2.42E-05
AL831737.1	2.586732	-3.41277	1.07E-05	2.44E-05

For Peer Review

EIF4BP5	2.264153	-2.96461	1.07E-05	2.45E-05
FP236383.1	2.499352	-3.48008	1.08E-05	2.46E-05
AL357153.4	3.070119	-3.30842	1.08E-05	2.47E-05
GAGE12J	3.32857	-4.03923	1.09E-05	2.48E-05
AC084819.1	2.549193	-4.04143	1.09E-05	2.50E-05
CNPY1	2.105771	-3.81809	1.10E-05	2.52E-05
AC110792.2	2.91545	-2.41011	1.11E-05	2.53E-05
AL445687.2	3.754397	-3.7833	1.11E-05	2.54E-05
ELK2BP	2.549893	-3.91609	1.11E-05	2.54E-05
AC090772.2	2.061832	-3.41195	1.12E-05	2.55E-05
MIR561	2.32446	-3.7462	1.13E-05	2.56E-05
AC012370.1	2.188041	-2.73352	1.13E-05	2.57E-05
CRCT1	3.684457	-2.34739	1.13E-05	2.57E-05
CEACAM18	4.060703	-3.22097	1.14E-05	2.59E-05
ATP5MC1P6	2.364626	-3.97051	1.15E-05	2.61E-05
AC239800.2	2.044656	-2.38161	1.15E-05	2.61E-05
CHCHD4P5	3.437029	-3.30071	1.15E-05	2.61E-05
DIO2-AS1	2.426337	-4.06307	1.16E-05	2.64E-05
DEFA8P	4.91629	-1.4682	1.16E-05	2.64E-05
AL606517.2	4.094316	-3.30833	1.17E-05	2.66E-05
AC138965.3	2.352881	-3.59201	1.18E-05	2.67E-05
DPRXP1	2.974734	-3.86947	1.19E-05	2.69E-05
CLCA4	2.150811	-0.69505	1.20E-05	2.73E-05
AC114878.1	3.594296	-3.47001	1.20E-05	2.73E-05
AC004009.1	3.357515	-3.97678	1.21E-05	2.73E-05
LINC02533	2.38268	-2.92721	1.24E-05	2.81E-05
DOCK4-AS1	2.374508	-1.89577	1.24E-05	2.81E-05
AC011595.1	2.57605	-3.12411	1.25E-05	2.82E-05
NEUROG3	2.299906	-1.8764	1.26E-05	2.84E-05
AC120349.2	3.512228	-3.48911	1.26E-05	2.84E-05
AL033504.1	2.694402	-2.41072	1.27E-05	2.88E-05
LINC01845	2.122215	-3.68746	1.28E-05	2.88E-05
AURKAP2	2.071279	-4.03433	1.28E-05	2.88E-05
AL360091.3	2.234416	-3.38885	1.29E-05	2.90E-05
ATP11A-AS1	2.216086	-1.30493	1.32E-05	2.96E-05
AC087241.4	2.174329	-3.02092	1.32E-05	2.98E-05
MTND5P26	3.823108	-3.09168	1.32E-05	2.98E-05
AL627311.1	2.686264	-4.00562	1.33E-05	2.99E-05
EIF4E2P1	3.486582	-3.78647	1.35E-05	3.04E-05
AC096947.1	2.22083	-2.72517	1.36E-05	3.06E-05
AP003497.2	2.740845	-3.75154	1.36E-05	3.07E-05
RN7SL197P	3.601318	-3.40307	1.37E-05	3.08E-05
MTCO2P19	3.089445	-3.58058	1.38E-05	3.10E-05

For Peer Review

ZNF723	3.173473	-2.69697	1.38E-05	3.10E-05
PIK3C2G	2.078841	1.955692	1.38E-05	3.10E-05
NLRP8	2.699936	-3.39293	1.39E-05	3.13E-05
RN7SKP48	3.921689	-3.68395	1.39E-05	3.13E-05
MTND1P32	3.25302	-3.41957	1.40E-05	3.15E-05
LINC01163	2.029691	-2.55132	1.41E-05	3.16E-05
AL391839.1	2.660662	-3.61009	1.41E-05	3.16E-05
MEI4	2.194859	-1.39839	1.43E-05	3.20E-05
RNU6-577P	2.107247	-3.99198	1.43E-05	3.21E-05
LINC01788	2.908377	-3.82996	1.43E-05	3.21E-05
APOC3	3.95285	-1.57232	1.43E-05	3.21E-05
LINC02307	3.1267	-3.87427	1.44E-05	3.23E-05
AL591719.1	3.184504	-3.58038	1.45E-05	3.25E-05
AC004817.4	2.418586	-3.95746	1.45E-05	3.26E-05
AP004289.2	3.259414	-3.15175	1.47E-05	3.30E-05
EPHA5-AS1	2.892663	-3.37647	1.47E-05	3.30E-05
C8A	2.324902	0.269352	1.50E-05	3.35E-05
AC131391.1	2.206789	-3.49243	1.50E-05	3.36E-05
RF00598	2.627487	-3.81252	1.51E-05	3.37E-05
AL358394.1	2.241022	-3.11395	1.52E-05	3.39E-05
RN7SKP8	3.577828	-3.04833	1.52E-05	3.39E-05
RNF113B	2.134941	-3.16387	1.53E-05	3.42E-05
AP005131.3	2.112456	-2.75787	1.53E-05	3.42E-05
AC004986.1	2.852975	-3.81905	1.53E-05	3.43E-05
Z99127.1	2.002035	-2.52931	1.54E-05	3.45E-05
AC023825.1	2.273379	-3.10665	1.55E-05	3.46E-05
CLEC3A	2.972307	-2.83417	1.56E-05	3.49E-05
OR7A19P	3.41144	-3.48232	1.57E-05	3.51E-05
SPRR2F	2.896151	-0.58235	1.60E-05	3.57E-05
RN7SL735P	3.472726	-3.02567	1.61E-05	3.58E-05
RN7SL302P	3.322648	-3.87105	1.63E-05	3.62E-05
TLX3	3.220327	-3.27348	1.63E-05	3.62E-05
AC020893.1	2.462901	-3.26637	1.63E-05	3.63E-05
AC112493.1	2.333799	-3.5425	1.63E-05	3.64E-05
CAPZA3	2.366876	-3.59624	1.64E-05	3.65E-05
AC093281.1	2.269744	-4.03449	1.64E-05	3.65E-05
DGAT2L6	2.124597	-3.30885	1.64E-05	3.65E-05
ARHGEF3-AS1	3.18933	-3.84008	1.67E-05	3.71E-05
MTND4P32	3.310484	-3.31494	1.68E-05	3.73E-05
AP000997.2	2.237718	-3.88479	1.70E-05	3.78E-05
AC113398.2	3.996946	-3.37698	1.71E-05	3.80E-05
AC104984.3	2.075389	-3.0395	1.71E-05	3.81E-05
REG1B	3.570275	-3.67565	1.72E-05	3.81E-05

For Peer Review

AL122126.1	3.324914	-3.81531	1.72E-05	3.81E-05
AC078899.4	2.691705	-1.99632	1.72E-05	3.82E-05
RN7SL18P	3.39332	-3.61246	1.74E-05	3.85E-05
NDST4	2.91691	-2.51938	1.75E-05	3.88E-05
RN7SL505P	2.588154	-3.66934	1.75E-05	3.89E-05
EEF1A1P32	2.427268	-2.61007	1.76E-05	3.91E-05
U51244.1	2.328057	-3.06557	1.76E-05	3.91E-05
TUNAR	3.810121	-1.36151	1.77E-05	3.92E-05
AC026785.1	2.110435	-3.44585	1.77E-05	3.93E-05
AC003989.2	3.290237	-3.71538	1.79E-05	3.96E-05
AC139722.1	2.784985	-4.01558	1.79E-05	3.97E-05
SOX1-OT	3.140151	-3.78067	1.80E-05	3.99E-05
RPTN	3.061067	-2.66728	1.80E-05	3.99E-05
AC009268.1	2.57772	-3.28823	1.81E-05	4.00E-05
AL162582.1	3.77156	-3.6247	1.81E-05	4.00E-05
DCAF12L1	2.94941	-2.13699	1.82E-05	4.03E-05
WFDC5	3.48994	1.712233	1.82E-05	4.03E-05
HSD3B1	3.332071	-2.68757	1.82E-05	4.03E-05
RN7SL141P	3.176759	-3.04734	1.87E-05	4.12E-05
SDCBPP1	2.026337	-3.22263	1.87E-05	4.14E-05
PRORY	4.079855	-3.55851	1.87E-05	4.14E-05
ZNF72P	3.310346	-3.9675	1.89E-05	4.17E-05
AC025254.1	3.267573	-3.97087	1.90E-05	4.18E-05
AL512363.1	2.078856	-3.51072	1.90E-05	4.20E-05
AC096741.1	3.456691	-2.20217	1.91E-05	4.21E-05
RN7SL43P	2.482425	-3.98531	1.92E-05	4.24E-05
AC013410.1	2.750351	-3.98194	1.95E-05	4.29E-05
RN7SL430P	2.989989	-3.77465	1.95E-05	4.29E-05
AC005476.1	2.304959	-2.79491	1.97E-05	4.34E-05
AC099494.3	2.176729	-3.51561	1.97E-05	4.34E-05
RNU2-23P	4.177406	-3.40511	2.01E-05	4.41E-05
TAS2R63P	2.564661	-3.24809	2.01E-05	4.42E-05
UNC5D	2.566405	0.807148	2.04E-05	4.49E-05
BLID	2.767481	-3.37746	2.05E-05	4.51E-05
RN7SL57P	3.164273	-3.60132	2.06E-05	4.51E-05
AC064874.1	2.596384	-3.3281	2.12E-05	4.65E-05
NEFM	2.155694	-0.33703	2.13E-05	4.67E-05
TSPY2	3.94256	-3.61886	2.14E-05	4.69E-05
HSPE1P13	2.718283	-3.11585	2.15E-05	4.70E-05
C10orf71-AS1	2.186352	-3.45289	2.15E-05	4.71E-05
AC013429.2	2.51077	-3.32381	2.16E-05	4.73E-05
TAS2R46	2.316524	-3.16127	2.21E-05	4.84E-05
NPPB	2.742974	-3.12226	2.22E-05	4.85E-05

For Peer Review

AC091925.2	2.618852	-3.19365	2.22E-05	4.85E-05
AC021766.1	2.989149	-3.63077	2.22E-05	4.86E-05
ZDHHC4P1	2.139754	-2.87632	2.23E-05	4.87E-05
MTCYBP32	3.830159	-3.34773	2.23E-05	4.88E-05
AP001207.1	2.392529	-3.6036	2.24E-05	4.89E-05
AL161658.1	3.940362	-1.14639	2.25E-05	4.91E-05
AP003696.1	2.480392	-2.47886	2.25E-05	4.91E-05
AC079316.2	2.677372	-2.56966	2.26E-05	4.95E-05
RPS15AP29	2.248909	-2.8522	2.27E-05	4.95E-05
TRIM40	2.311796	-2.27893	2.27E-05	4.95E-05
LINC01551	2.883262	-3.70088	2.30E-05	5.01E-05
PRODH2	3.760943	-1.88318	2.31E-05	5.03E-05
RN7SL166P	2.654193	-3.22683	2.31E-05	5.05E-05
LINC01192	2.923171	-3.69843	2.31E-05	5.05E-05
AC002381.1	3.042583	-3.87474	2.33E-05	5.08E-05
BNIP3P25	2.123765	-3.07291	2.35E-05	5.13E-05
CASP14	3.83314	1.072462	2.35E-05	5.13E-05
RASA2-IT1	2.695301	-3.16452	2.36E-05	5.13E-05
AL513188.1	2.814212	-3.73985	2.36E-05	5.14E-05
TMPRSS11GP	2.480959	-3.89292	2.37E-05	5.16E-05
AL512324.3	2.370176	-3.68397	2.38E-05	5.17E-05
CD177P1	2.748554	-2.33543	2.39E-05	5.20E-05
H2BFWT	2.861854	-3.96403	2.42E-05	5.27E-05
AC016251.1	2.087419	-3.13256	2.42E-05	5.27E-05
AC092638.2	2.889816	-4.0273	2.43E-05	5.28E-05
ZNF75BP	3.82841	-3.44326	2.43E-05	5.28E-05
RHOXF2B	3.328489	-3.95519	2.46E-05	5.34E-05
ARL4AP1	3.012706	-2.93111	2.50E-05	5.43E-05
AL160281.1	2.699991	-3.49841	2.51E-05	5.45E-05
AC063926.1	2.602534	-3.85239	2.52E-05	5.47E-05
GKN1	3.69341	-1.91155	2.53E-05	5.49E-05
AL513412.1	2.68667	-3.51811	2.53E-05	5.49E-05
AC092447.1	2.864616	-3.97983	2.55E-05	5.52E-05
AC008883.1	2.217434	-3.88985	2.56E-05	5.54E-05
AC005722.3	2.26102	-3.01052	2.58E-05	5.58E-05
AC109439.2	2.882306	-3.72484	2.60E-05	5.62E-05
AC007731.1	2.310219	-3.79288	2.61E-05	5.64E-05
CT47B1	3.303327	-3.99655	2.61E-05	5.65E-05
AL807757.1	2.401122	-2.96234	2.66E-05	5.75E-05
GNAT3	3.422694	-3.26025	2.67E-05	5.77E-05
AP000462.2	3.901272	-2.64083	2.69E-05	5.81E-05
AL450344.2	2.43282	-3.31061	2.70E-05	5.82E-05
FAM8A2P	2.791335	-4.13922	2.70E-05	5.83E-05

For Peer Review

SNORD70	2.020074	-3.85539	2.70E-05	5.83E-05
ARHGAP26-IT1	2.247839	-3.41432	2.72E-05	5.87E-05
CARM1P1	2.543816	-3.87305	2.75E-05	5.92E-05
FCF1P5	2.130367	-3.16907	2.77E-05	5.98E-05
AC087482.1	2.163243	-3.32114	2.80E-05	6.03E-05
AC114763.2	2.550515	-3.70272	2.80E-05	6.03E-05
ATP5PBP3	2.195337	-3.63163	2.83E-05	6.09E-05
AMER3	2.535843	-2.4758	2.84E-05	6.11E-05
AC144521.1	3.347733	-3.5792	2.86E-05	6.14E-05
SHISA9	2.499274	-0.09586	2.86E-05	6.16E-05
AC010145.1	3.359172	-4.02047	2.87E-05	6.16E-05
DLGAP1-AS5	2.281252	-1.32277	2.89E-05	6.22E-05
RNY4	3.756311	-3.36577	2.92E-05	6.27E-05
USP24P1	3.359874	-3.9297	2.93E-05	6.30E-05
AC034154.1	2.005581	-3.98675	2.96E-05	6.36E-05
LINC01878	2.83556	-4.01095	2.97E-05	6.37E-05
AC004491.1	2.247828	-2.9301	2.97E-05	6.38E-05
AL121900.1	2.934309	-3.59696	3.02E-05	6.47E-05
AC009313.1	2.85608	-3.81819	3.03E-05	6.49E-05
AC091805.1	2.164878	-3.81119	3.04E-05	6.52E-05
S100A7A	3.416109	-1.64036	3.08E-05	6.60E-05
AC020687.1	2.904545	-4.00713	3.09E-05	6.62E-05
AMBN	2.38085	-3.78102	3.15E-05	6.75E-05
AC003989.1	3.242916	-3.3175	3.16E-05	6.77E-05
MYOD1	3.064473	-3.90608	3.20E-05	6.84E-05
AL356273.1	2.147788	-3.73205	3.21E-05	6.86E-05
AC079866.1	2.23657	-3.07656	3.23E-05	6.91E-05
BIRC6-AS1	3.07396	-3.178	3.26E-05	6.96E-05
RN7SL630P	3.453522	-3.45513	3.26E-05	6.97E-05
AC008498.2	2.825712	-3.20846	3.26E-05	6.97E-05
ATP5MC1P1	3.295062	-3.82638	3.28E-05	7.00E-05
AL133492.1	2.131103	-3.54338	3.29E-05	7.03E-05
COQ10BP2	2.21453	-3.81785	3.36E-05	7.17E-05
AC131902.1	2.109566	-2.92552	3.37E-05	7.18E-05
AC126323.5	2.136247	-4.01831	3.37E-05	7.19E-05
AC017037.3	2.395506	-3.80431	3.38E-05	7.20E-05
AC106744.2	3.543225	-3.75887	3.41E-05	7.27E-05
HTR2C	2.850086	-0.63707	3.43E-05	7.31E-05
AL445523.1	3.32288	-3.79251	3.44E-05	7.33E-05
AC120024.4	2.805109	-2.83461	3.48E-05	7.40E-05
RN7SL308P	3.302351	-3.7679	3.50E-05	7.44E-05
RN7SL408P	3.328927	-3.13576	3.51E-05	7.47E-05

For Peer Review

AC003958.2	2.34988	-3.81109	3.57E-05	7.59E-05
AP001803.2	2.416804	-3.37356	3.58E-05	7.60E-05
AC100801.1	2.355291	-3.35471	3.60E-05	7.64E-05
SPINT5P	2.75839	-3.8149	3.60E-05	7.64E-05
RNU4-24P	2.648163	-3.53131	3.65E-05	7.74E-05
ATG10-AS1	3.319933	-3.81693	3.66E-05	7.76E-05
PDHA1P1	2.866738	-3.18767	3.68E-05	7.80E-05
AP000462.1	3.045434	-2.60833	3.72E-05	7.88E-05
AC093770.1	2.784047	-3.7371	3.78E-05	8.01E-05
LINC00488	3.013432	-3.60624	3.79E-05	8.02E-05
AC091814.1	2.1629	-3.58852	3.79E-05	8.02E-05
DPPA5	3.466698	-2.90664	3.80E-05	8.05E-05
AF107885.2	2.710357	-3.30576	3.89E-05	8.23E-05
MAGI1-AS1	2.901816	-2.65053	3.91E-05	8.26E-05
AL137789.1	2.098123	-3.68459	3.92E-05	8.28E-05
AC015574.1	3.490351	-3.43738	3.92E-05	8.29E-05
AC073916.1	2.045927	-3.97079	3.93E-05	8.31E-05
MIR576	2.699477	-3.99758	3.95E-05	8.34E-05
AC078962.3	2.599978	-3.64927	3.97E-05	8.38E-05
REG3A	4.120394	-2.24092	4.00E-05	8.44E-05
ATG12P2	2.028768	-3.22115	4.00E-05	8.45E-05
SMAD1-AS2	2.66892	-3.05479	4.01E-05	8.47E-05
MIR4300HG	2.445475	-3.90279	4.04E-05	8.53E-05
TATDN2P1	3.030443	-2.47054	4.05E-05	8.55E-05
PCA3	2.371298	-2.94294	4.08E-05	8.61E-05
AF121897.1	2.020776	-3.91479	4.14E-05	8.72E-05
RPL23AP36	2.271122	-4.00041	4.15E-05	8.74E-05
AL391421.1	2.120918	-3.20085	4.18E-05	8.80E-05
AC017099.1	3.343733	-3.64644	4.19E-05	8.81E-05
RPL6P12	2.612542	-3.36553	4.22E-05	8.87E-05
RN7SL646P	3.386246	-3.64064	4.23E-05	8.89E-05
TRIM43	2.856719	-3.9463	4.25E-05	8.94E-05
AC108729.1	3.018621	-3.87851	4.27E-05	8.98E-05
AL591926.3	2.123362	-3.96952	4.29E-05	9.03E-05
EGLN3-AS1	2.169893	-4.06463	4.32E-05	9.09E-05
RNU2-63P	2.416717	-3.74009	4.33E-05	9.10E-05
RN7SL362P	3.166659	-3.97647	4.34E-05	9.11E-05
AC012506.3	2.558638	-4.05161	4.37E-05	9.18E-05
RN7SL38P	3.351636	-3.84493	4.38E-05	9.20E-05
RN7SL706P	2.41261	-4.021	4.39E-05	9.22E-05
AC079209.2	2.543729	-3.3059	4.40E-05	9.23E-05
RPL35AP25	2.489774	-3.96939	4.43E-05	9.31E-05
RPL36AP33	2.231721	-3.86997	4.48E-05	9.39E-05

For Peer Review

AC023905.1	2.685704	-3.96545	4.49E-05	9.41E-05
IFNK	2.926324	-2.79458	4.50E-05	9.43E-05
MRGPRX3	2.23211	-3.61478	4.56E-05	9.55E-05
Z99496.1	2.414192	-3.00729	4.57E-05	9.56E-05
AC108693.1	2.397401	-2.82679	4.57E-05	9.57E-05
RN7SKP133	3.332493	-3.90522	4.59E-05	9.60E-05
KRT6C	2.412173	-0.57288	4.60E-05	9.63E-05
GFRA4	2.745845	-3.99883	4.60E-05	9.63E-05
MIR3140	3.422737	-3.65442	4.62E-05	9.67E-05
AL035634.1	2.924872	-3.30746	4.63E-05	9.69E-05
LINC01976	2.147683	-4.05138	4.66E-05	9.75E-05
AC131902.3	2.243679	-3.11772	4.66E-05	9.75E-05
AC022616.1	2.311644	-4.11298	4.67E-05	9.76E-05
RN7SKP230	2.22609	-3.80334	4.67E-05	9.77E-05
DSCR10	2.578495	-4.04197	4.74E-05	9.92E-05
RPL9P30	2.131068	-3.3056	4.76E-05	9.94E-05
PRSS38	2.919857	-3.84231	4.76E-05	9.95E-05
RNU6-82P	3.39182	-3.54025	4.76E-05	9.95E-05
RN7SL144P	2.607102	-3.66065	4.77E-05	9.97E-05
LINC00485	2.302806	-3.73012	4.79E-05	0.000100078
NDUFAF4P4	2.555848	-3.71629	4.81E-05	0.000100416
AC004593.1	2.612697	-3.46803	4.81E-05	0.000100512
UPK1B	2.620126	3.566704	4.89E-05	0.000102066
RF01169	2.494406	-3.79105	4.93E-05	0.00010281
LINC01470	2.117052	-3.88342	5.00E-05	0.000104137
FP671120.3	2.221871	-3.45886	5.03E-05	0.000104687
AC021146.2	2.004892	-3.93014	5.03E-05	0.000104745
AL592528.1	2.098103	-3.71033	5.04E-05	0.000104867
ADAMTS19	2.608974	-0.53754	5.04E-05	0.000104911
SNORA35B	3.447262	-3.81444	5.05E-05	0.000105052
TSPY3	3.115761	-4.09769	5.08E-05	0.000105631
RNA5-8SP5	3.768821	-3.79063	5.09E-05	0.000105927
AC013467.2	2.398927	-3.7292	5.10E-05	0.000106056
RN7SL543P	3.204828	-3.90364	5.10E-05	0.000106086
AC092851.1	2.372493	-2.66081	5.11E-05	0.000106299
AC006461.1	2.721565	-3.96503	5.11E-05	0.000106325
OR9K1P	2.607208	-4.10432	5.12E-05	0.000106402
IGLV3-26	2.781228	-4.07969	5.12E-05	0.00010644
AL591501.1	2.674841	-3.22292	5.15E-05	0.000107047
FP236383.3	3.846592	-2.28709	5.19E-05	0.000107911
DANT1	2.53687	-3.91361	5.20E-05	0.000108084
AC009951.1	2.05536	-0.52592	5.23E-05	0.000108677
TMEM196	2.976905	-2.64376	5.24E-05	0.000108712

For Peer Review

LINC02228	2.25854	-3.2422	5.27E-05	0.000109508
AC117500.1	2.213551	-3.66237	5.30E-05	0.000109926
AC244131.1	3.078374	-3.65367	5.31E-05	0.000110287
AL136313.1	3.333889	-3.80475	5.33E-05	0.000110527
AC091046.2	3.109032	-3.68734	5.34E-05	0.000110779
AC111000.4	2.575932	-3.03632	5.34E-05	0.000110779
UGT2B4	2.050607	1.996214	5.40E-05	0.000111942
RARRES2P6	2.824131	-4.07768	5.49E-05	0.000113616
AC007012.2	3.322717	-3.98585	5.54E-05	0.000114511
AL161668.1	3.280619	-3.76608	5.56E-05	0.000114952
AC007161.2	2.720526	-2.94109	5.58E-05	0.000115483
KRT38	2.561205	-3.31879	5.72E-05	0.000118087
KCTD9P5	2.734721	-3.67611	5.73E-05	0.000118269
AC010480.1	2.577741	-3.32071	5.74E-05	0.000118502
AC253536.4	2.077226	-3.73025	5.75E-05	0.000118745
AL354943.1	2.305753	-3.28764	5.76E-05	0.000118818
C2orf69P1	3.287567	-3.88796	5.77E-05	0.000119105
AC000061.1	2.318989	-2.43247	5.80E-05	0.00011961
AC098592.2	2.704143	-3.96483	5.81E-05	0.000119825
SLC9A9-AS1	2.856452	-3.92245	5.90E-05	0.000121513
IFNL4P1	2.479672	-4.0107	5.91E-05	0.000121668
RPAP2P1	2.563215	-3.20691	6.02E-05	0.000123883
AC019072.1	2.20043	-2.84747	6.05E-05	0.000124489
AC134050.1	2.059854	-3.78069	6.09E-05	0.000125251
UBE2U	2.108634	-2.21513	6.13E-05	0.000126139
AC012339.1	3.063404	-3.95035	6.16E-05	0.000126544
HECW1-IT1	2.915341	-3.9869	6.16E-05	0.000126722
RNU4ATAC16P	2.460226	-3.42901	6.27E-05	0.000128828
AC016716.2	3.346479	-3.95764	6.29E-05	0.000129196
AC099313.1	2.954405	-3.98153	6.30E-05	0.000129396
AC002351.1	2.249962	-3.45913	6.34E-05	0.000130123
EDDM3B	2.442544	-3.43549	6.38E-05	0.000130852
SLC17A2	3.05632	-3.72169	6.62E-05	0.000135542
AL136320.1	2.642825	-2.12166	6.62E-05	0.000135559
OR8A1	2.741695	-4.10149	6.68E-05	0.000136783
TFAP2B	2.728755	0.327984	6.72E-05	0.000137566
LINC00440	2.496232	-3.82879	6.75E-05	0.000138118
KRT37	2.422555	-4.03886	6.86E-05	0.000140347
RNA5SP202	3.139476	-3.56387	6.93E-05	0.000141624
TNMD	2.633018	-0.59002	7.07E-05	0.000144384
SSX5	3.421107	-1.37059	7.08E-05	0.000144464
LINC02282	3.015667	-4.07256	7.08E-05	0.000144464

For Peer Review

MIR124-2HG	2.967166	-4.1404	7.09E-05	0.000144621
AC055733.2	3.014365	-3.9636	7.10E-05	0.000144841
AC009646.2	2.540594	-3.21411	7.17E-05	0.00014627
AC006504.4	2.872488	-4.00215	7.24E-05	0.000147717
SPINK8	2.086635	-3.69486	7.25E-05	0.000147853
SLC6A18	2.132987	-3.16143	7.26E-05	0.000148079
AC100763.1	3.262915	-3.27196	7.27E-05	0.000148174
AP000897.1	3.024918	-3.57148	7.27E-05	0.000148182
AC019070.1	2.679163	-3.30215	7.27E-05	0.000148261
C12orf40	2.1778	-3.35064	7.28E-05	0.000148335
AL513475.1	3.040317	-3.4871	7.35E-05	0.000149673
AL117341.1	3.04282	-3.86552	7.36E-05	0.000149946
AC092447.7	2.726744	-3.57114	7.38E-05	0.000150362
AC024475.1	2.256253	-3.31751	7.39E-05	0.000150517
LINC00200	3.15717	-1.98718	7.44E-05	0.00015133
AC010857.1	2.804499	-3.94691	7.45E-05	0.000151526
MIR3976HG	3.557269	-3.69142	7.47E-05	0.000152017
RN7SL498P	2.907146	-3.98376	7.52E-05	0.000152881
CTXN3	2.181307	-3.47927	7.54E-05	0.000153328
AC115621.1	2.611915	-3.3327	7.54E-05	0.000153365
AC092910.2	3.247372	-3.67955	7.58E-05	0.000154024
AC092608.3	3.096862	-3.83771	7.62E-05	0.000154863
AC008277.2	2.612181	-3.39716	7.64E-05	0.000155102
AP001011.1	2.129538	-3.17038	7.68E-05	0.000155846
RNU7-124P	2.907	-3.82129	7.71E-05	0.000156441
AL021395.1	2.792427	-2.40727	7.81E-05	0.00015848
LGALS14	3.188686	-3.18102	7.89E-05	0.000159924
KCTD4	2.239878	-2.52684	8.03E-05	0.000162669
LINC02128	2.317437	-3.5983	8.13E-05	0.000164511
AC241520.1	2.999529	-2.91299	8.25E-05	0.000166957
SERPINB13	2.576872	0.053487	8.31E-05	0.000167957
SMIM32	2.003615	-2.34385	8.34E-05	0.000168611
BX510359.2	3.217496	-3.8778	8.41E-05	0.00016991
SETP10	2.562857	-3.21895	8.43E-05	0.000170311
AC009044.1	2.843387	-3.40916	8.50E-05	0.000171628
AC016493.1	3.143656	-3.36785	8.53E-05	0.000172323
RN7SL493P	2.870716	-3.54461	8.57E-05	0.000173017
TRPC5	2.01338	-3.16855	8.61E-05	0.000173738
Z98749.1	2.031465	-3.3473	8.61E-05	0.000173747
LINC01574	2.176599	-3.98932	8.63E-05	0.000173996
AL807761.4	2.508096	-3.44905	8.72E-05	0.000175785
OR1N2	2.770435	-3.69344	8.73E-05	0.000175944
LINC01443	2.307654	-2.56154	8.95E-05	0.00018016

For Peer Review

GLRA4	2.4845	-2.78932	8.96E-05	0.000180419
AC012363.1	2.625475	-4.05469	8.97E-05	0.000180499
AC097721.1	2.23904	-3.78228	8.99E-05	0.000180891
CDY4P	3.273512	-3.69908	9.11E-05	0.000183082
SLC25A24P1	2.189584	-2.86426	9.14E-05	0.000183744
AC090802.1	3.076894	-3.64376	9.16E-05	0.000184086
SCARNA23	2.96945	-3.85379	9.24E-05	0.000185495
RN7SL792P	2.756756	-3.67653	9.31E-05	0.000186911
RNU2-59P	3.462813	-3.18291	9.43E-05	0.000189169
AC005828.7	2.00633	-3.49952	9.57E-05	0.000191853
CR589904.2	2.236882	-4.06534	9.57E-05	0.000191853
SELENOV	2.276533	-3.81306	9.67E-05	0.000193677
OR9A4	2.286767	-3.75089	9.86E-05	0.000197311
AC106872.1	3.096095	-4.02838	9.91E-05	0.000198301
AC022778.1	2.721255	-3.37923	9.99E-05	0.000199671
PRSS48	2.173532	-2.4368	0.000100614	0.0002011
WASF1P1	2.585568	-3.98164	0.000101184	0.00020217
AC138761.3	2.166178	-4.06293	0.00010278	0.000205218
AC092447.5	2.378092	-2.81122	0.000102927	0.000205465
AC079597.1	2.717489	-4.04578	0.000103107	0.000205799
AC105150.1	2.858301	-3.72446	0.000104111	0.000207614
AL596442.1	2.472756	-3.92312	0.000104732	0.000208733
CLCA1	2.881611	-0.97064	0.000104751	0.000208747
TBX20	2.015536	-2.6482	0.000105168	0.000209543
MIR670HG	2.780909	-3.6269	0.000105542	0.00021024
AC132825.4	2.635125	-3.86859	0.000105585	0.000210313
AL049637.1	2.053135	-2.68348	0.000105855	0.000210802
RNU6-1283P	2.575674	-3.67566	0.000105937	0.000210954
CLVS2	3.150468	-1.90021	0.000106217	0.000211474
AC092332.1	2.283935	-3.97137	0.000106296	0.000211619
TAAR1	2.519392	-3.47051	0.000107627	0.000214148
AC036222.1	2.462617	-2.70239	0.000108885	0.000216527
AC009365.2	2.512759	-3.11375	0.000109444	0.000217525
FSHB	3.384874	-3.84532	0.000109493	0.000217611
HIGD1AP17	2.248163	-3.85418	0.000109869	0.000218283
CACNA1C-AS4	3.112045	-2.9688	0.000110079	0.000218663
CRISP1	3.600858	-3.63511	0.000110232	0.000218929
AL513475.2	2.667912	-3.46993	0.000110609	0.000219628
RN7SL173P	2.45365	-4.01129	0.000113422	0.000224894
AGXT2	2.25633	-2.67064	0.00011405	0.000226048
HMGB1P23	2.496504	-3.8737	0.000114196	0.000226325
LINC02027	2.06087	-3.90822	0.000114874	0.000227605
EPHA5	2.164839	-0.15418	0.0001151	0.000228005

For Peer Review

VPS26BP1	2.729225	-3.26295	0.000115484	0.000228733
AC010967.1	2.191124	-4.00333	0.000115638	0.000229026
AC060834.2	2.738319	-3.9585	0.000116327	0.000230273
AC245128.1	3.338072	-3.46162	0.000116371	0.000230348
AL035410.1	2.083536	-3.47231	0.000116708	0.000230976
AC013652.2	2.249021	-3.86384	0.000116916	0.000231346
AC008517.1	3.050638	-3.93694	0.000117975	0.000233283
AC013444.2	2.208918	-3.74917	0.000119574	0.000236243
SNORD56B	3.104225	-3.65397	0.000120232	0.000237436
CBX3P3	2.477254	-3.82558	0.000121857	0.000240481
MTCYBP3	2.212869	-3.5566	0.00012243	0.000241502
RN7SKP103	2.878242	-4.00102	0.000123428	0.000243306
AIRN	2.255986	-3.32708	0.000123688	0.000243778
AC087286.1	2.826721	-2.03871	0.000124623	0.00024555
AC021517.1	2.206596	-2.0801	0.000125788	0.000247706
AC026894.1	2.023923	-2.53504	0.000126159	0.000248377
EIF2S2P5	2.350673	-3.50685	0.000126164	0.000248377
AL137779.3	2.594274	-3.47386	0.000127563	0.00025096
RN7SL390P	2.539887	-3.5961	0.000128091	0.000251985
AC011120.1	2.044437	-2.94058	0.000128922	0.000253518
DBX1	2.412067	-4.01581	0.000128993	0.000253643
AP001970.1	2.751918	-3.95807	0.000129611	0.000254758
LINC01425	2.798089	-3.85917	0.000131163	0.00025759
TLE7	3.205289	-3.63237	0.000131615	0.000258376
AC016576.1	2.884703	-3.75506	0.000131798	0.00025872
AL139393.1	2.399479	-3.11311	0.000133053	0.000261038
COX5BP3	2.36876	-3.74218	0.000134738	0.000264165
RN7SL297P	2.762453	-3.6002	0.000137254	0.000268795
LINC01179	2.009799	-3.9375	0.000138621	0.00027135
AC129502.1	2.080533	-3.85426	0.000139908	0.000273792
AC013437.1	3.085904	-3.7872	0.000140346	0.000274602
LINC02050	2.577591	-3.42643	0.000140577	0.000275019
RPS12P16	2.053388	-3.01088	0.000144398	0.000281914
GABRB1	2.250781	-2.45286	0.000145249	0.000283512
AKAIN1	2.611438	-3.43869	0.000145287	0.00028357
AL158801.3	2.089009	-2.99188	0.000145674	0.000284246
LINC01580	2.572287	-4.09418	0.000145703	0.000284285
CDC42EP3P1	2.082619	-3.66873	0.000147091	0.000286817
LINC02310	2.174391	-4.06213	0.000147192	0.000286998
AC090241.1	2.070982	-3.74402	0.000147919	0.000288255
AC136603.1	2.899298	-3.99174	0.000148065	0.000288507
CXorf67	2.211107	-1.57642	0.000148316	0.000288898
AC021237.1	2.530303	-3.39803	0.00014869	0.000289545

For Peer Review

Z93022.1	2.117639	-3.1185	0.000151	0.000293846
MZT1P1	2.43673	-4.00737	0.000151221	0.000294244
HMGB1P51	2.201972	-3.37764	0.000151249	0.000294282
HSPA8P19	2.183365	-3.58093	0.000154754	0.000300765
RNU1-2	3.033402	-3.64294	0.000156508	0.000303853
ATP5PBP6	2.410683	-3.93281	0.000156915	0.000304608
AP006259.1	2.238037	-3.2809	0.000158621	0.000307732
AC092612.1	2.21038	-3.94754	0.000158996	0.000308408
AC009884.2	2.204349	-3.95183	0.000160113	0.000310436
AC105114.1	2.594968	-3.95257	0.00016068	0.000311449
METTL21AP1	2.74261	-3.33764	0.000163971	0.000317386
RNASEH1P1	2.260944	-3.61202	0.000164873	0.000318971
AC022201.1	2.360276	-3.99263	0.000167049	0.000322948
RNU6-619P	2.807176	-3.92256	0.000169417	0.000327253
RN7SL555P	2.8154	-3.51297	0.000170825	0.000329809
RC3H1-IT1	2.013341	-3.31101	0.00017303	0.000333752
STX18-IT1	2.250331	-3.67863	0.00017429	0.000335996
AC011939.2	2.351668	-2.72679	0.00017558	0.000338332
RNU6-789P	2.377452	-3.95043	0.000176944	0.000340792
PNMA6B	2.021469	-2.95523	0.000180477	0.000347173
MTATP6P19	2.447932	-3.77167	0.000183071	0.000351735
ARHGAP23P1	2.378048	-3.45391	0.000183093	0.000351758
RNU6-251P	2.835318	-3.73953	0.000184586	0.000354391
BX510359.3	3.089136	-3.9674	0.000185062	0.000355266
ENO1P3	2.575797	-3.73962	0.000186095	0.000357172
NDUFB4P1	2.456063	-3.63111	0.000186518	0.000357924
AC022080.3	2.254778	-3.89134	0.000186573	0.000357989
AC103409.1	2.605542	-3.73007	0.000187645	0.000360007
AC114812.4	2.068761	-3.79612	0.000189888	0.000363969
AL645730.1	2.705726	-4.02215	0.000193976	0.000371191
PANTR1	3.08219	-3.25905	0.000194815	0.000372653
RN7SL554P	2.904555	-3.84029	0.000197406	0.000377278
ZBTB20-AS3	3.090646	-3.40206	0.000202166	0.000385909
AC105031.2	3.246999	-3.74733	0.000203571	0.000388529
AC005183.1	2.236525	-2.3614	0.000204234	0.000389686
AL358333.2	2.728411	-3.8011	0.000204897	0.000390864
RN7SL272P	3.137349	-3.91185	0.000204973	0.000390968
AC112196.1	2.147402	-3.02159	0.000207914	0.000396143
BACH1-IT3	2.704765	-3.65098	0.000208532	0.000397269
LINC01475	2.600645	-3.86272	0.000209069	0.000398235
AL356805.1	2.242162	-3.20367	0.000209662	0.000399299
AC069079.1	3.048267	-3.59454	0.000211527	0.000402565
RTP1	2.376299	-2.29768	0.000213597	0.000406393

For Peer Review

GPC6-AS1	2.745939	-3.78244	0.000214487	0.000407909
SERPINB3	2.05082	3.125959	0.000218449	0.000415035
AC012404.1	2.988514	-3.33197	0.000219301	0.000416518
AC009248.2	2.43031	-3.82547	0.000220754	0.000419071
GIF	2.424951	-2.7886	0.000221517	0.000420359
AP003108.1	2.126272	-2.92371	0.0002259	0.000428281
RN7SL346P	2.336492	-3.86413	0.000232484	0.000440163
USP12-AS1	2.551287	-3.53467	0.000233481	0.00044198
AL606534.2	2.696506	-3.92128	0.000234378	0.000443532
RN7SL778P	2.506607	-3.99918	0.000238469	0.000450783
RN7SL160P	2.204873	-3.92353	0.000239287	0.000452257
SUCLA2P1	2.066277	-2.9704	0.000241053	0.000455422
AC080128.1	2.650991	-3.77952	0.000244903	0.000462393
BX842568.1	3.728218	-3.15012	0.000248137	0.000468322
BRWD1-IT1	2.064996	-3.46867	0.000249001	0.000469852
NDUFB4P2	2.513663	-3.59469	0.000250176	0.000471888
MIR624	2.300248	-3.84526	0.000253615	0.000477988
RPL17P17	2.788671	-3.24315	0.000254746	0.000479988
AC130404.1	2.23703	-4.05663	0.000255493	0.000481318
NPM1P30	2.044004	-3.35546	0.000257393	0.000484602
LINC01040	2.897231	-4.08568	0.000257547	0.000484847
CCNJP2	2.626328	-3.44655	0.000258806	0.000486954
RNU6-608P	2.491187	-3.76385	0.000260973	0.000490818
SSX6P	2.820741	-2.60531	0.000261024	0.000490888
AF131215.2	2.09443	-2.45854	0.000265031	0.000497966
PRR13P1	2.167959	-3.73218	0.000266997	0.000501255
AC015522.1	2.536169	-4.0549	0.000267069	0.000501312
RNU5D-1	2.644264	-2.97846	0.000270133	0.000506759
AC016251.2	2.189766	-3.70114	0.000271758	0.00050956
AL589863.1	3.107505	-3.036	0.00027567	0.000516562
CR589904.1	2.434315	-2.46153	0.000277821	0.000520313
AC016866.2	2.406559	-3.79481	0.000285334	0.000533723
MARK3P1	2.250131	-3.78966	0.000286441	0.000535621
AC019270.1	2.371598	-3.9829	0.00028674	0.000536094
LINC02437	2.785746	-3.71888	0.000287035	0.000536529
SCGB1D2	2.794328	-2.78923	0.000288172	0.000538425
AP002001.1	2.59193	-3.98926	0.000294677	0.000549664
AC092608.2	2.951669	-3.88303	0.000294913	0.000549987
DEFA6	3.29547	-2.73282	0.000297527	0.000554534
LINC02316	2.119113	-4.01474	0.000299643	0.00055812
AC008833.1	2.861497	-3.64249	0.000303163	0.000564435
AC107886.1	2.246147	-3.88202	0.000303487	0.000564917
RNU2-6P	2.11153	-3.67946	0.000304294	0.000566298

For Peer Review

PROS2P	2.103395	-2.3406	0.000305567	0.000568515
SELENOOLP	2.156599	-1.82478	0.000309976	0.000576194
NBPF22P	2.091322	-3.92976	0.000310353	0.000576742
AL606517.1	2.596904	-3.04847	0.000311566	0.000578841
AL365338.1	2.237627	-3.53536	0.000312232	0.000579862
AP002453.1	2.947684	-3.77949	0.000312445	0.000580226
RN7SL183P	2.896936	-3.97825	0.000312974	0.000581086
AC016542.3	2.529426	-2.86262	0.00031435	0.000583578
AL133480.1	2.081986	-3.63573	0.000317966	0.0005898
AC093903.1	2.335937	-2.70055	0.000326183	0.000603966
DEFB4B	2.462889	-3.91602	0.000328983	0.000608762
RPL23AP40	2.358444	-3.9337	0.000330188	0.000610798
GP2	2.861312	2.750021	0.000331731	0.000613391
AL133384.1	2.657444	-3.99549	0.000333663	0.000616833
AC022537.1	3.314752	-3.48955	0.000334334	0.000617975
RNU11-2P	2.713136	-3.85878	0.000334747	0.000618639
PRR20G	2.572895	-1.26736	0.00033776	0.000623843
C10orf99	2.591456	-2.64009	0.000342305	0.000631869
AC013286.1	2.855411	-3.84774	0.000343185	0.000633461
AC098869.1	2.287071	-3.48672	0.000347752	0.00064138
FOXN3-AS2	2.698228	-3.09936	0.000348917	0.000643393
LCE3D	3.076132	-3.46757	0.000351898	0.000648478
AC007599.2	2.403531	-3.75949	0.000363407	0.000668095
AC106876.1	2.27236	-2.14992	0.000364828	0.00067053
DEFB4A	2.063549	-1.26128	0.000369598	0.000678616
RPL26P9	2.41198	-3.7693	0.000370854	0.000680702
AC090753.1	2.837625	-3.74801	0.000373016	0.00068453
TVP23CP1	2.157455	-3.70302	0.000375456	0.000688391
CR545473.1	2.159495	-3.88104	0.000377809	0.000692561
AC009135.2	2.508479	-3.86496	0.000388565	0.000711266
SI	2.474169	-3.60622	0.000389818	0.000713334
ENSAP1	2.750982	-3.50155	0.000391174	0.00071577
RN7SL683P	2.701879	-3.48491	0.00040499	0.000739428
RN7SKP299	2.190403	-3.9345	0.00040746	0.000743585
CYCSP4	2.154368	-3.94466	0.00041323	0.000753246
AC079316.1	2.40262	-3.52297	0.000413836	0.000754273
RPL23AP17	2.052354	-3.81018	0.000421595	0.00076753
RN7SL321P	2.336605	-4.00173	0.000424685	0.000772913
RBM22P4	2.516341	-4.00008	0.000434425	0.00078969
TMPOP1	2.761832	-3.49422	0.000435564	0.000791679
MAST4-IT1	2.741417	-3.32077	0.000444225	0.000806662
PAQR9	2.84829	-1.47877	0.000459635	0.00083269
AP001318.3	2.421304	-3.85261	0.000460074	0.0008334

For Peer Review

SPRR2G	2.86793	-1.57453	0.000460261	0.000833694
LINC02241	2.489788	-1.05754	0.000465438	0.000842243
AL138880.1	2.219004	-3.97541	0.000468731	0.000847889
RNU1-87P	2.663433	-3.83747	0.000469749	0.00084951
AC009303.3	2.521781	-3.69514	0.000473038	0.000855015
RNU6-998P	2.090279	-3.70951	0.000474538	0.000857504
AL121952.1	2.334305	-3.32582	0.000478607	0.00086435
AC018607.1	2.021796	-2.72035	0.000486646	0.000878016
AL031658.2	2.663226	-3.57805	0.000497273	0.00089575
AL512593.1	2.159411	-3.93895	0.00049966	0.00089977
AC090018.1	2.227424	-3.93762	0.000499972	0.00090024
PRSS3P1	2.218807	-4.03802	0.000504538	0.000908086
SMG6-IT1	2.324935	-3.93461	0.000523224	0.00093958
RN7SL660P	2.203689	-3.76438	0.000525204	0.000942747
AC011473.4	2.391268	-3.88217	0.000525703	0.000943544
PIN1P1	2.125584	-3.99722	0.000535501	0.00096014
AC110994.1	2.019525	-3.98361	0.000551635	0.000987283
PRAC1	2.714174	-3.8369	0.000574421	0.001025382
RIPPLY2	2.121895	-2.59877	0.000587273	0.00104682
RPL37P18	2.345647	-3.59264	0.000588664	0.001049032
AC090116.1	2.224425	-3.92086	0.000596397	0.001061921
AL662889.1	2.584514	-3.69818	0.000602799	0.001072958
AC008060.4	2.412894	-3.85132	0.000603885	0.001074671
AC098868.1	2.368862	-4.01592	0.000607331	0.001080526
AP003086.3	2.125216	-3.6359	0.000616958	0.001096702
RNU6-711P	2.101587	-4.0077	0.000622611	0.00110613
MTCO2P33	2.357368	-3.49442	0.000631638	0.00112108
RN7SL260P	2.679332	-3.77029	0.000637031	0.001130422
MC3R	2.55084	-4.09131	0.000637168	0.001130608
RN7SKP257	2.211058	-3.9471	0.000645377	0.001144065
AC009656.1	2.270038	-3.97531	0.000646965	0.001146646
AC013549.2	2.164141	-3.54193	0.000653461	0.001157452
AL022313.4	2.723719	-3.56747	0.000661142	0.001169867
AC023824.3	2.398706	-3.87448	0.000673262	0.001189862
DOCK9-AS1	2.24677	-3.63974	0.000683981	0.001207887
AC096558.2	2.501401	-3.60059	0.000686336	0.0012118
RN7SL575P	2.523762	-3.85801	0.000686481	0.001211994
AC005344.1	2.870615	-3.72532	0.000689932	0.001217655
COL18A1-AS2	2.031525	-3.56602	0.000698725	0.001232299
MPC1L	2.375684	-3.31606	0.00069936	0.001233357
LINC02008	2.364457	-3.87947	0.000706298	0.001244646
RNU7-79P	2.490236	-3.89216	0.000711299	0.001252888
LCN9	2.947496	-3.27952	0.000720232	0.001267404

For Peer Review

AL139002.1	2.620628	-3.47087	0.00072786	0.00128031
RNU6-1327P	2.061936	-3.93641	0.000738886	0.001298066
AC026725.1	2.113594	-3.70321	0.000743903	0.001306286
AC109635.4	2.664308	-2.84307	0.000773993	0.001355841
AP006248.4	2.076863	-3.43254	0.000794008	0.001388387
PABPC1L2A	2.17317	-3.97854	0.000794409	0.001388948
AC136424.2	2.519587	-3.64775	0.00079813	0.001394964
AL049820.1	2.004311	-3.66502	0.000803498	0.001403431
AL356488.2	2.027395	-3.60163	0.000817391	0.001425624
HTR1E	2.178485	-3.5608	0.000864323	0.001501649
AL137071.1	2.041594	-4.00845	0.000867935	0.001507435
AC023347.1	2.179744	-3.67467	0.000874318	0.001518141
AC090707.1	2.121874	-3.94587	0.00090455	0.001567144
SULT2A1	2.011781	-3.62176	0.000908228	0.001572548
MORC1	2.006046	-3.38135	0.00093906	0.001622385
AC067805.1	2.848157	-3.98184	0.000947799	0.001636914
AC036111.1	2.418977	-3.9305	0.000951246	0.001642543
VSX2	2.039123	-1.74776	0.000973499	0.001678221
AC104137.1	2.174806	-4.03592	0.00098628	0.001698487
LINC01205	2.217511	-3.6321	0.001025246	0.001761496
RERG-AS1	2.114603	-4.01066	0.001042132	0.001788831
AL357033.2	2.079724	-3.86859	0.001046054	0.001795122
HMX1	2.098904	-3.76882	0.001049506	0.001800513
AC092608.1	2.416666	-3.78949	0.001051469	0.001803791
AF131215.1	2.167959	-3.38396	0.001057295	0.001813071
RN7SL402P	2.145413	-3.85648	0.00106649	0.001828029
RNY4P7	2.139031	-3.73237	0.001123202	0.001918718
SPRR2E	2.638503	-0.81777	0.001142616	0.0019493
RSU1P3	2.562017	-3.5599	0.001147386	0.001956958
ZNF859P	2.362179	-3.91188	0.001171394	0.001994583
ZNF209P	2.112886	-3.31459	0.001185224	0.002015964
RHOXF2	2.495707	-3.65027	0.001204847	0.002046642
AC005230.1	2.093787	-3.75807	0.001214064	0.002061594
KPRP	2.71488	-3.45129	0.001220473	0.002071467
AC068643.1	2.196392	-1.61199	0.001307927	0.002210202
LINC01793	2.603305	-3.36113	0.00132285	0.00223466
RN7SKP185	2.030753	-2.9386	0.001324903	0.002237803
AP003168.1	2.145668	-3.98564	0.001398072	0.00235442
SPRR2C	2.126551	-1.59207	0.001463392	0.002456934
RN7SL471P	2.394679	-3.80651	0.00146797	0.002463908
AC106827.1	2.140818	-3.54267	0.001475658	0.002475619
AC020661.2	2.034012	-3.64748	0.00150062	0.002514467
AC106047.2	2.119469	-3.82894	0.001528999	0.00255894

For Peer Review

RN7SL597P	2.037364	-3.60273	0.001544942	0.002584131
AP001642.1	2.465853	-3.93505	0.001576038	0.002632856
AC107896.1	2.029852	-3.58814	0.001595293	0.002662211
TMIGD1	2.157795	-3.6761	0.001595569	0.002662545
AP005059.2	2.188842	-3.95556	0.001624119	0.002708109
AL391095.2	2.186948	-3.93542	0.00167311	0.002783201
AL139042.1	2.072187	-3.64446	0.001713097	0.002845165
AC097639.2	2.180865	-3.8538	0.001759882	0.002918133
AC008568.1	2.305072	-3.88179	0.00187867	0.003101364
AC103770.1	2.101448	-3.48137	0.001994905	0.003279874
AC055733.3	2.029866	-3.90053	0.002062897	0.00338447
BCAS2P1	2.18837	-3.93322	0.002063025	0.00338452
AC007608.2	2.117358	-3.78913	0.002157631	0.003530244
RNA5SP33	2.109189	-3.96181	0.002261027	0.003689395
AC147055.1	2.149564	-3.38378	0.002291862	0.003735823
PNMA6E	2.411257	-1.36933	0.002309539	0.003762877
E2F6P4	2.024159	-3.65567	0.002475256	0.00401409
AC092349.1	2.165812	-3.93494	0.002744972	0.004424694
RN7SL218P	2.326995	-3.942	0.002916145	0.004679812
VSTM2B	2.156329	-3.94026	0.002948019	0.004729001
AL136146.1	2.000788	-3.96225	0.003242917	0.005170372
AC005972.2	2.176644	-3.70674	0.003284947	0.005234267
CYP1D1P	2.269348	-4.00205	0.004078668	0.00641618
CELA3A	2.158825	-3.30258	0.004134963	0.006499746
AC136424.1	2.253794	-3.85549	0.004566439	0.007127141
CELF2-AS2	2.028941	-2.92176	0.004725556	0.007360637
AC008808.2	2.092292	-3.13576	0.004739226	0.007380279
SOX5-AS1	2.01634	-3.94574	0.004781177	0.007440283
LINC02106	2.401429	-3.31254	0.004909173	0.007624811
BX119904.2	2.225458	-3.72886	0.006293978	0.009622832

For Peer Review

Table S2. Gene-set enrichment analysis of differentially expressed genes.

Function	Size	ES	NES	NOM p-val	FDR q-val	FWER p-val	Rank at max	Leading edge
Regulation of defense response	78	-0.58497	-2.2047117	0	0.003892	0.041	784	tags=42%, list=14%, signal=48%
Regulation of immune effector process	48	-0.59547	-2.1086519	0.002375297	0.005186	0.108	784	tags=42%, list=14%, signal=48%
Negative regulation of defense response	17	-0.69827	-2.0278819	0	0.007378	0.201	340	tags=41%, list=6%, signal=44%
Immune system process	246	-0.41526	-1.9466684	0	0.010845	0.326	806	tags=34%, list=14%, signal=38%
Defense response	186	-0.41342	-1.9308381	0.002724796	0.011483	0.353	813	tags=31%, list=14%, signal=35%
Positive regulation of defense response	30	-0.54719	-1.887034	0.009259259	0.015034	0.417	784	tags=43%, list=14%, signal=50%
Negative regulation of immune effector process	15	-0.6599	-1.8660088	0.00422833	0.016669	0.454	366	tags=40%, list=6%, signal=43%
Negative regulation of immune response	19	-0.58674	-1.8186781	0.013422819	0.023016	0.543	796	tags=47%, list=14%, signal=55%
Immune response	152	-0.403	-1.7627841	0.002457002	0.030287	0.623	806	tags=34%, list=14%, signal=38%
Regulation of immune	156	-0.41914	-1.751054	0.005128205	0.032249	0.643	804	tags=32%, list=14%,

For Peer Review

system process								signal=36%
Regulation of innate immune response	20	-0.51772	-1.7426001	0.016317016	0.033976	0.654	320	tags=25%, list=6%, signal=26%
Positive regulation of immune system process	93	-0.46959	-1.7359775	0.018018018	0.035217	0.665	804	tags=40%, list=14%, signal=45%

For Peer Review

Table S3. Twelve immune biological processes and 353 corresponding immune genes.

Biological process	Gene
Defense response	PPP1R14B, LGR4, MDK, KCNN4, STYK1, IL1RL2, F12, HMGB3, ADAM8, TNFRSF18, SPP1, IGKC, PTGES, IGHG1, IL31RA, CYP27B1, IGLL5, IGHG2, IGHG3, IGLC3, SRMS, IGLC2, APOBEC3B, HIST1H3H, AIM2, IGKV3-20, HIST1H2BC, HCAR1, COCH, IGHG4, IGHV3-23, IGHV4OR15-8, IL23A, IL36G, PGLYRP4, IGHA2, GRIK2, IL36RN, IL20RB, CYP4F11, MUC5B, IGLL1, NR2E1, LCN2, TRIM15, C4BPB, CCL25, PLK5, IGHV1OR21-1, HIST1H2BJ, TRIM10, PGLYRP3, TFF3, TRIM31, LPO, CST11, APOBEC1, IL37, OPRK1, DEFB126, HIST1H3D, OPRM1, IL17C, HIST1H2BG, CARD18, IFNE, BPIFA2, BPIFA1, CFHR5, FGB, KRT16, FGA, CAMK2B, RAET1L, TAC4, AHSG, IL36A, CA7, GABRA5, HIST1H3G, HIST1H2BF, CHST4, APOA4, CALCA, HTR2C, HIST1H2BE, IFNK, APCS, KLRF2, GAGE1, PAGE1, HIST1H3J, GAL, VGF, HIST1H3I, DEFB4A, HIST1H3B, IL19, RNASE7, HIST1H3A, LBP, UGT1A1, CLEC2A, HIST1H3C, F2, HIST1H2BI, PYDC1, HIST1H2BA, KNG1, HIST1H3F, S100A7, GALP, SCG2, PRB3, CRP, APOC3, C8A, C10orf99, APOA2, REG3A, PSG3, HRG, DEFA6, DEFA5, TAC1, STAR, MSTN, INS, NPY, UMOD, KLK3, PTX3, DEFA4, ALOX15, PPBP, PGLYRP1, AGTR2, SELE, DEFA3, C6, PF4, CCL24, IL6, PROK2, IL5RA, CXCL2, C8B, FCGR3B, PADI4, CAMP, CXCR1, CLEC4M, FPR2, PLA2G1B, PDK4, EDN1, CXCR2, CCL14, LILRA5, VSIG4, IL33, FGR, CD300C, FCN3, MRC1, CFD, CCL23, SFTPD, SIRPB1, OLR1, MASP1, CFP, SELP, CDO1, ODAM, CD5L, MARCO, NLRC4, CD36, SPN, SCUBE1, CD93, CLEC1A, AOC3, AQP4, AGER
Immune response	PPP1R14B, LGR4, MNX1, STYK1, EXO1, IL1RL2, F12, HMGB3, TNFRSF18, IGKC, IGHG1, IL31RA, CYP27B1, IGLL5, IGHG2, IGKV3D-11, IGHG3, IGLC3, IGKV4-1, SRMS, IGLC2, APOBEC3B, IGKV1-5, AIM2, IGKV3-20, GCNT3, HIST1H2BC, ENPP3, IGHG4, IGHV3-23, IGHV4OR15-8, ADAMDEC1, FCRL4, IL23A, IL36G, PGLYRP4, IGLV7-43, IGHA2, IGHV2-70, IGKV3D-20, IL36RN, IL20RB, TNFSF11, IGLL1, VTCN1, LCN2, CXCL14, TRIM15, C4BPB, CCL25, IGHV1OR15-1, OPRD1, IGHV1OR21-1, HIST1H2BJ, TRIM10, PGLYRP3, CDH17, TINAG, TRIM31, IGKV1D-33, IL37, OPRK1, DEFB126, IGKV2-40, OPRM1, HIST1H2BG, IFNE, BPIFA1, CFHR5, CRHR1, FGB, KRT16, FGA, CAMK2B, RAET1L, CPLX2, IL36A, HIST1H2BF, CHST4, APOA4, CALCA, HIST1H2BE, IFNK, APCS, KLRF2, DEFB4A, IL19, RNASE7, LBP, NKX2-3,

For Peer Review

	CLEC2A, HIST1H2BI, PYDC1, S100A7, C8A, DEFA6, DEFA5, TAC1, STAR, CYP11B1, NPY, KLK3, COLEC10, PKHD1L1, PTX3, CEACAM8, DEFA4, PPBP, PGLYRP1, CSF3, DEFA3, C6, PF4, CCL24, IL6, IL1RL1, IL5RA, CXCL2, C8B, FCGR3B, PADI4, CAMP, NR4A3, CLEC4M, PLA2G1B, EDN1, CCL14, LILRA5, VSIG4, IL33, SMAD6, IL7R, FGR, FCN3, MRC1, CFD, SUSD2, CCL23, VIPR1, SFTPD, SIRPB1, MASP1, CFP, MARCO, NLRC4, CD36, SPN, TGFBR3, LAMP3, AQP4, AGER, EMP2
Immune system process	PPP1R14B, LGR4, KIF4A, TOP2A, KIF11, HELLS, MELK, PODXL2, POLQ, KIF2C, ESCO2, CENPE, SPTBN2, KIF15, KCNN4, MNX1, KIF23, STYK1, EXO1, CEACAM1, IL1RL2, ANLN, CCNB2, F12, HMGB3, ADAM8, TNFRSF18, SLC7A5, SIX1, COL1A1, IGKC, IGHG1, IL31RA, HOXB7, CYP27B1, IGLL5, MB, IGHG2, IGKV3D-11, SLC7A11, IGHG3, IGLC3, IGKV4-1, SRMS, IGLC2, APOBEC3B, IGKV1-5, AIM2, THPO, SLC16A8, IGKV3-20, EFNA2, GCNT3, HIST1H2BC, MMP9, ENPP3, ONECUT1, IGHG4, IGHV3-23, IGHV4OR15-8, ADAMDEC1, NKX3-2, FCRL4, IL23A, IL36G, PITX2, PGLYRP4, IGLV7-43, IGHA2, IGHV2-70, BARX1, IGKV3D-20, IL36RN, IL20RB, IL11, KIF5A, TNFSF11, IGLL1, VTCN1, LCN2, CXCL14, SLC7A10, TRIM15, C4BPB, ABCB4, CCL25, IGHV1OR15-1, OPRD1, ARTN, IGHV1OR21-1, HIST1H2BJ, TRIM10, PGLYRP3, CDH17, TINAG, PROC, PTPRZ1, RAB3B, TRIM31, IGKV1D-33, LY6D, FZD9, APOBEC1, SLC7A9, IL37, OPRK1, DEFB126, IGKV2-40, HHLA2, OPRM1, FOXE1, RET, HIST1H2BG, IFNE, MMP1, INHA, BPIFA1, CFHR5, CRHR1, FGB, KRT16, FGA, CAMK2B, RAET1L, CPLX2, IL36A, NKX2-5, HIST1H2BF, CHST4, APOA4, CALCA, HIST1H2BE, IFNK, APCS, KLRF2, DHRS2, CHRNB2, DEFB4A, IL19, RNASE7, LBP, NKX2-3, CLEC2A, F2, KRT75, HIST1H2BI, PYDC1, HIST1H2BA, S100A7, SCG2, CRP, C8A, CD177, DEFA6, DEFA5, APOB, TAC1, STAR, CYP11B1, INS, NPY, UMOD, KLK3, COLEC10, PKHD1L1, PTX3, CEACAM8, DEFA4, PPBP, TMEM190, PGLYRP1, CSF3, SELE, DEFA3, C6, PF4, CCL24, IL6, IL1RL1, MAG, IL5RA, CXCL2, C8B, EDN3, FCGR3B, PADI4, CAMP, NR4A3, SH3GL2, ZBTB16, ALAS2, CXCR1, CLEC4M, PLA2G1B, MYH2, EDN1, CXCR2, CCL14, FGF10, LILRA5, GPC3, VSIG4, IL33, SMAD6, KLF4, IL7R, FGR, CD300C, FCN3, MRC1, CFD, SUSD2, ANGPT1, THBD, CCL23, VIPR1, CD300LG, SFTPD, SIRPB1, OLR1, MASP1, CFP, SELP, CD5L, ANGPT4, MARCO, NLRC4, CD36, SPN, CD93, TGFBR3, TAL1, TCF21, EPAS1, S1PR1, LAMP3, EDNRB, TEK, WNT3A, RTKN2, AQP4, JAM2, AGER, PECAM1, CAV1, EMP2

Negative regulation of defense response	PBK, IL22RA2, IL20RB, PROC, ISL1, SERPINB4, APCS, IGF2, INS, PGLYRP1, KLF4, SPN, SLIT2, FOXF1, CALCRL, TEK, ADRB2
Negative regulation of immune effector process	CEACAM1, IL20RB, C4BPB, PGLYRP3, SERPINB4, APOA2, IGF2, INS, PGLYRP1, IL33, IL7R, ANGPT1, MASP1, SPN, FOXF1
Negative regulation of immune response	CEACAM1, COL3A1, IL20RB, C4BPB, PGLYRP3, SERPINB4, AMBP, APOA2, IGF2, INS, ALOX15, PGLYRP1, IL1RL1, IL33, IL7R, ANGPT1, MASP1, SPN, FOXF1
Positive regulation of defense response	F12, ADAM8, PLA2G4A, AIM2, COCH, IL23A, PGLYRP4, TNFSF11, TRIM15, PGLYRP3, EREG, AGT, IFNK, LBP, PYDC1, TAC1, CREB3L3, PGLYRP1, CCL24, IL6, IL1RL1, CCL14, IL33, LPL, CCL23, FABP4, MARCO, NLRC4, CD36, AGER
Positive regulation of immune system process	KCNN4, IL1RL2, ADAM8, TNFRSF18, IGKC, IGHG1, IGLL5, IGHG2, IGKV3D-11, MZB1, IGHG3, IGLC3, IGKV4-1, IGLC2, IGKV1-5, TESC, AIM2, THPO, IGKV3-20, COCH, IGHG4, IGHV3-23, IGHV4OR15-8, IL23A, PGLYRP4, IGLV7-43, IGHA2, IGHV2-70, IGKV3D-20, TNFSF11, IGLL1, VTCN1, CXCL14, TRIM15, C4BPB, ABCB4, IGHV1OR21-1, PGLYRP3, IGKV1D-33, EREG, F7, IGKV2-40, HHLA2, CFHR5, CALCA, THBS4, IFNK, CHRNB2, LBP, TEX101, PYDC1, S100A7, C8A, IGF2, HRG, TAC1, COLEC10, PPBP, PGLYRP1, CSF3, C6, PF4, CCL24, IL6, RBP4, IL1RL1, CXCL2, C8B, EDN3, NR4A3, ZBTB16, PLA2G1B, MYH2, FGF10, CD101, VSIG4, IL33, IL7R, FGR, FCN3, CFD, MASP1, CFP, SELP, MARCO, NLRC4, CD36, SPN, FOXF1, TAL1, WNT3A, AGER, CAV1
Regulation of defense response	LGR4, IL1RL2, PBK, F12, ADAM8, IL22RA2, PLA2G4A, HIST1H3H, AIM2, HCAR1, COCH, IL23A, PGLYRP4, IL20RB, TNFSF11, TRIM15, C4BPB, PGLYRP3, PROC, EREG, HIST1H3D, ISL1, SERPINB4, AHSG, CA7, GABRA5, AGT, HIST1H3G, IFNK, APCS, HIST1H3J, HIST1H3I, HIST1H3B, HIST1H3A, DUOXA2, LBP, HIST1H3C, PYDC1, HIST1H3F, C8A, IGF2, TAC1, CREB3L3, MSTN, INS, PGLYRP1, SELE, C6, CCL24, IL6, IL1RL1, C8B, FCGR3B, SCGB1A1, PDK4, CCL14, IL33, LPL, KLF4, FGR, CASP12, CCL23, FABP4, MASP1, CFP, MARCO, NLRC4, CD36, SPN, CD93, SLIT2, FOXF1, CALCRL, EDNRB, TEK, ADRB2, AGER, CAV1
Regulation of immune effector process	CEACAM1, MZB1, HIST1H3H, AIM2, HCAR1, IL23A, IL20RB, TRIM15, C4BPB, PGLYRP3, HIST1H3D, SERPINB4, CA7, GABRA5, HIST1H3G, HIST1H3J, HIST1H3I, HIST1H3B, HIST1H3A, LBP, HIST1H3C, PYDC1, HIST1H3F, C8A, APOA2, IGF2, MSTN, INS, PGLYRP1, C6, IL6, RBP4, C8B, FCGR3B, NR4A3, PDK4, PRAM1, IL33, IL7R, FGR, ANGPT1, MASP1,

For Peer Review

	CFP, CD36, SPN, CD93, FOXF1, AGER
Regulation of immune system process	LGR4, NME1, KCNN4, CEACAM1, IL1RL2, ADAM8, GREM1, TNFRSF18, COL3A1, COL1A1, HIST2H4A, FCRL5, IGKC, IGHG1, IL31RA, IGLL5, IGHG2, IGKV3D-11, MZB1, IGHG3, IGLC3, IGKV4-1, IGLC2, CDKN2A, HIST1H3H, IGKV1-5, TESC, AIM2, THPO, IGKV3-20, HIST1H4K, HCAR1, COCH, IGHG4, IGHV3-23, IGHV4OR15-8, IL23A, PGLYRP4, IGLV7-43, IGHA2, IGHV2-70, IGKV3D-20, COL17A1, HIST1H4H, IL20RB, TNFSF11, MUC5B, IGLL1, VTCN1, CXCL14, TRIM15, C4BPB, ABCB4, CCL25, HIST1H4J, IGHV1OR21-1, PGLYRP3, IGKV1D-33, EREG, F7, IGKV2-40, HIST1H3D, PLA2G2F, HHLA2, HOXB8, INHA, CFHR5, SOX11, SERPINB4, CA7, GABRA5, HIST1H3G, CALCA, HIST1H4E, AMBP, THBS4, IFNK, APCS, HIST1H4C, HIST1H3J, GAL, CHRNB2, HIST1H4B, HIST1H3I, HIST1H3B, HIST1H3A, LBP, HIST1H4A, HIST1H3C, HIST1H4D, TEX101, PYDC1, COL2A1, HIST1H3F, S100A7, HIST1H4L, HIST1H4F, OLFM4, C8A, APOA2, IGF2, HRG, FSHB, TAC1, MSTN, INS, COLEC10, ALOX15, PPBP, PGLYRP1, CSF3, C6, PF4, CCL24, IL6, RBP4, IL1RL1, CXCL2, C8B, EDN3, FCGR3B, SCGB1A1, NR4A3, ZBTB16, PLA2G1B, MYH2, PDK4, FGF10, CD101, VSIG4, PRAM1, IL33, OSCAR, IL7R, FGR, CD300C, FCN3, CFD, ANGPT1, CD300LG, SFTPD, MASP1, CFP, SELP, MARCO, NLRC4, CD36, SPN, CD93, SLIT2, FOXF1, TAL1, CLDN18, WNT3A, AGER, CAV1
Regulation of innate immune response	LGR4, ADAM8, AIM2, COCH, PGLYRP4, TRIM15, PGLYRP3, EREG, SERPINB4, IFNK, LBP, PYDC1, IGF2, INS, PGLYRP1, FGR, MARCO, NLRC4, CD36, CAV1

For Peer Review

Table S4. Univariate Cox regression analysis of 353 immune genes.

Gene	Hazard ratio	P value
ANLN	1.320307747	1.80E-07
EXO1	1.278327619	4.59E-06
AHSG	1.203153414	4.80E-06
F2	1.118851094	2.07E-05
KIF4A	1.243056193	5.12E-05
KIF23	1.250017038	8.61E-05
RAET1L	1.150392672	9.20E-05
CCNB2	1.254026764	0.0001
KIF11	1.28892496	0.000102
VIPR1	0.829341416	0.000111
PBK	1.206791779	0.000131
CENPE	1.239538053	0.000238
KIF2C	1.206019836	0.000481
MELK	1.195411728	0.000493
STYK1	1.23635514	0.000607
PKHD1L1	0.860673955	0.000662
CDH17	1.090875491	0.00071
CASP12	0.812064729	0.000729
SFTPD	0.911614769	0.000775
AQP4	0.928436751	0.000839
IL20RB	1.113054844	0.000858
ESCO2	1.223814511	0.001145
LGR4	1.262196027	0.001251
ADRB2	0.850657734	0.001286
C10orf99	1.138577176	0.001591
SUSD2	0.902958742	0.001649
PLA2G1B	0.914608097	0.002132
IL11	1.145761877	0.002147
GAL	1.083121012	0.002223
TOP2A	1.174490957	0.002224
PSG3	1.093268639	0.002586
STAR	0.840889444	0.002716
IFNE	1.107603932	0.002725
KRT16	1.079257672	0.00281
EREG	1.064943294	0.003003
C6	0.916896221	0.003359
F12	1.171855287	0.003551
ALOX15	0.915258336	0.003711
POLQ	1.163598446	0.004271
TCF21	0.877867455	0.004299
GRIK2	1.105941061	0.004335

For Peer Review

KIF15	1.18266656	0.004416
EDN3	0.873385337	0.004655
AGER	0.909653436	0.004882
APOC3	1.131690601	0.005368
SLC7A5	1.16853801	0.005574
GPC3	0.886305102	0.006381
EFNA2	1.096777581	0.006382
TAC4	0.88793173	0.006494
RAB3B	1.079971618	0.007865
HOXB7	1.116394615	0.007911
PPP1R14B	1.268495907	0.008305
WNT3A	0.904635034	0.008776
HRG	1.121822377	0.01008
CRP	1.112779926	0.010324
HELLS	1.199756192	0.010398
SLC7A11	1.095297982	0.010489
INHA	1.063051671	0.011266
ENPP3	0.92823726	0.011996
IL33	0.883876859	0.012013
LPL	0.909202702	0.012221
SERPINB4	1.058569457	0.012743
VGF	1.078288109	0.012852
TRIM15	1.065300175	0.0137
TRIM10	1.089837028	0.014404
APOBEC1	1.066330169	0.014827
SCGB1A1	0.958055041	0.015094
C8B	0.933523024	0.015167
HIST1H2BC	1.119098994	0.015169
SPN	0.865299367	0.015245
TINAG	1.065205716	0.015299
FCRL4	0.906389906	0.015542
ODAM	0.908954707	0.015926
IL5RA	0.908090124	0.016845
CLDN18	0.950611017	0.017007
COL1A1	1.117139329	0.017392
CD300LG	0.917184832	0.017942
NKX2-5	1.098109488	0.018156
CEACAM8	0.912541902	0.019999
APCS	1.130998641	0.021453
FCRL5	0.913827036	0.021674
IGLC3	0.919591599	0.022421
IL7R	0.884033617	0.02366
NLRC4	0.832991833	0.023837

For Peer Review

HIST2H4A	1.128221352	0.025509
PRAM1	0.873626004	0.025597
KLF4	1.134540385	0.025699
AMBP	0.94070138	0.026273
MMP1	1.0688233	0.026442
CD101	0.854986242	0.027115
GREM1	1.076961157	0.027462
NME1	1.191986378	0.029894
HIST1H3H	1.100894815	0.030126
SCUBE1	0.902448975	0.031971
S100A7	1.057453758	0.03354
CCL14	0.917294275	0.033622
JAM2	0.847937048	0.035081
IGKV4-1	0.931321568	0.035603
FGA	1.037666551	0.03656
PTX3	1.10817042	0.037255
PECAM1	0.824163161	0.037815
PTPRZ1	0.943781225	0.03881
FCN3	0.913246775	0.041874
EMP2	0.863278911	0.042177
KCNN4	1.089763245	0.043267
SIX1	0.919437523	0.045519
CAMP	0.918241837	0.045929
MRC1	0.921236385	0.046793
TNFSF11	1.074253341	0.047147
LAMP3	0.913025687	0.047371
KNG1	1.06729039	0.047485
OPRM1	1.145275725	0.048018
SELP	0.906316907	0.048092
PTGES	1.103421068	0.051113
TEK	0.884540671	0.052422
CDO1	0.899936134	0.053584
IGKV3-20	0.932004469	0.054041
COL3A1	1.098726062	0.054319
FGF10	0.922964068	0.054898
IGKV3D-20	0.936119808	0.055063
TMEM190	0.940290261	0.057066
IGKV2-40	0.901285314	0.057126
CAV1	1.103429867	0.059268
CLEC1A	0.850545903	0.063586
BARX1	1.035566703	0.064329
APOB	1.063549916	0.067481
HIST1H2BF	1.07074882	0.068151

For Peer Review

IGHV1OR15-1	0.886422726	0.072473
IGKC	0.936723773	0.075038
FGR	0.889710666	0.07531
SCG2	1.061626841	0.076611
AOC3	0.906909571	0.077855
CHST4	1.062848515	0.081317
TRIM31	1.057288972	0.081527
CREB3L3	1.09050615	0.082309
SPTBN2	1.135527375	0.086437
FGB	1.024545482	0.09019
IGHG1	0.945005579	0.091883
MZB1	0.934863096	0.093253
ANGPT1	0.920842043	0.098082
TAL1	0.890907842	0.100365
IGKV1-5	0.941212449	0.10104
IGHV3-23	0.938347169	0.102986
FOXF1	0.896748778	0.103031
CPLX2	1.030262955	0.10532
IGKV3D-11	0.944793189	0.110157
IGHG4	0.949577923	0.112567
CFP	0.907630077	0.115796
HOXB8	1.041146105	0.115875
IGHV1OR21-1	0.943076222	0.127713
OPRD1	0.933895454	0.131981
IL23A	1.089856888	0.133727
MUC5B	1.030097483	0.134005
IGLC2	0.944745241	0.139881
MYH2	0.928286232	0.142329
MASP1	0.935871744	0.143048
IL17C	1.054326022	0.143102
S1PR1	0.897584125	0.143202
SPP1	1.051800574	0.150779
OLR1	0.935500518	0.153816
CXCR2	0.93309426	0.164194
NKX3-2	1.051907983	0.169301
COCH	1.051399661	0.169502
CARD18	1.065346381	0.176388
SLC7A10	0.965235446	0.179013
CD36	0.936150145	0.180332
FOXE1	1.031560188	0.1805
OPRK1	0.963562482	0.18202
LBP	1.033320466	0.182579
HIST1H2BG	1.042329696	0.184852

For Peer Review

ONECUT1	1.046364689	0.186976
CST11	1.094111866	0.187104
HIST1H3D	1.051426919	0.187916
LY6D	1.030711876	0.188331
IGHG3	0.953654039	0.190251
AGTR2	0.970022823	0.192262
COL17A1	1.036699342	0.192745
CAMK2B	0.956591096	0.192779
THBS4	0.95356848	0.197253
EPAS1	0.89849623	0.19977
MSTN	0.932897359	0.201093
C4BPB	0.962868267	0.202603
MAG	0.945621047	0.20286
SMAD6	0.905190227	0.205729
GCNT3	1.042635153	0.20652
OSCAR	0.931856606	0.208397
IGHG2	0.953929916	0.209156
PGLYRP3	1.039580783	0.211015
IGLL1	0.946048074	0.212454
KLK3	0.921988918	0.213139
CXCL2	0.942712521	0.213554
IL36RN	1.031003903	0.214589
RTKN2	0.933027943	0.220207
CD300C	0.934820668	0.221334
HCAR1	0.963141559	0.228572
IGKV1D-33	0.96216234	0.237999
PODXL2	0.95645814	0.245726
HIST1H2BI	0.931332583	0.246157
IGLV7-43	0.961434206	0.250702
MNX1	1.04186093	0.251215
HIST1H3A	0.936364045	0.255844
IL22RA2	0.960002848	0.25746
HTR2C	1.032087956	0.262459
IL37	0.97464866	0.271967
HIST1H4C	0.945047905	0.278907
PLA2G4A	1.043869759	0.282344
MMP9	1.047671019	0.284374
RNASE7	1.045466279	0.296798
UGT1A1	1.03940621	0.298593
CDKN2A	1.033424274	0.306602
DEFA3	1.0719637	0.308448
UMOD	0.927932428	0.321042
BPIFA2	0.975340994	0.325367

For Peer Review

ZBTB16	0.968182079	0.325992
MARCO	0.967262523	0.32937
CALCRL	0.937870705	0.329649
CYP11B1	0.921644045	0.335115
IGLL5	0.964223524	0.341035
VTCN1	0.973028228	0.34522
GALP	1.074544937	0.347789
IL1RL1	0.965813962	0.349475
VSIG4	0.956029752	0.350257
CCL23	0.959301203	0.351198
RET	0.968751574	0.357742
IGHV2-70	0.974377732	0.358075
ARTN	1.040647424	0.361628
CD93	0.939668648	0.365531
CLEC2A	0.952795882	0.365608
CALCA	0.983957149	0.366869
GABRA5	1.040657268	0.367357
IGHA2	0.966569596	0.368405
COL2A1	0.975813494	0.370188
TFF3	0.977901024	0.377391
ABCB4	0.951861195	0.378179
DEFA4	1.0756847	0.378728
CEACAM1	0.947856568	0.393248
HIST1H4L	0.936679061	0.39939
HIST1H3B	1.033320982	0.399553
MDK	1.047746215	0.402095
KRT75	1.024967913	0.403688
HIST1H3J	1.037178072	0.414951
INS	1.053308917	0.421537
EDN1	1.043477837	0.428759
CA7	0.958201143	0.430505
DEFB4A	0.971360306	0.436423
CXCL14	0.980173303	0.437756
HIST1H4H	1.034088586	0.458153
CLEC4M	0.963508944	0.462292
APOA4	1.036999282	0.463909
CFD	1.037717377	0.464896
IL31RA	1.021586427	0.465289
PITX2	1.016364473	0.481293
ISL1	0.980404983	0.495143
C8A	0.977509433	0.495775
PROC	0.974596983	0.501668
SH3GL2	0.977042893	0.50382

For Peer Review

DEFA5	0.95491323	0.506014
HIST1H2BA	0.949988713	0.513977
TESC	1.018784904	0.52204
HMGB3	0.968182609	0.534269
EDNRB	0.965784309	0.537091
ALAS2	1.03052296	0.547379
ADAMDEC1	0.977552812	0.547529
RBP4	1.023114708	0.558732
IGF2	1.018471369	0.565091
PDK4	0.971804296	0.57383
CXCR1	0.976630096	0.578279
COLEC10	0.969024229	0.58016
HIST1H3G	0.981014911	0.586557
HIST1H4F	0.959471054	0.588247
CD177	0.983505464	0.590711
HIST1H4E	1.018728965	0.60144
HIST1H3C	0.975600619	0.604327
PGLYRP4	0.984232596	0.618684
TNFRSF18	1.023756915	0.624069
PPBP	1.015365699	0.624783
SLIT2	0.970932702	0.6279
APOA2	1.023028602	0.634428
SELE	0.979705498	0.646993
DHRS2	1.014410637	0.649098
TAC1	1.016243006	0.662128
PF4	1.018867227	0.6642
FCGR3B	1.017071842	0.66774
HIST1H4A	1.023986187	0.668807
IL19	0.980143785	0.673481
SLC16A8	0.980218097	0.675192
IGHV4OR15-8	0.980036009	0.675958
DEFA6	1.03108532	0.683251
IFNK	1.028324862	0.687994
CYP27B1	0.973852124	0.689373
NR2E1	1.01392701	0.693959
SIRPB1	1.018936505	0.7043
PLA2G2F	1.013349177	0.715478
IL6	1.014623002	0.721994
AGT	1.011944712	0.727406
TGFBR3	0.979758666	0.730133
THBD	0.978150481	0.733859
FSHB	1.039339977	0.742559
IL36A	0.972459828	0.744803

For Peer Review

SLC7A9	1.011840695	0.750791
APOBEC3B	1.01400921	0.751019
PGLYRP1	1.019990072	0.757225
AIM2	1.011394968	0.759564
IL36G	1.01052581	0.762218
CCL24	1.012225675	0.764506
CHRNB2	1.011834027	0.776493
F7	1.009512415	0.778209
KIF5A	0.989634981	0.779396
FZD9	1.00817516	0.793894
HIST1H4D	0.988500792	0.795738
NPY	1.0088434	0.806736
THPO	1.008108635	0.810124
MB	1.008632732	0.813401
IL1RL2	1.013781854	0.818763
CRHR1	0.991890513	0.820007
TEX101	0.99153644	0.830529
CFHR5	0.98545038	0.836872
HIST1H2BE	1.009290838	0.839175
HIST1H2BJ	1.008708563	0.840734
FPR2	0.99170664	0.841703
HIST1H4B	0.990075549	0.847365
GAGE1	1.010076067	0.847386
CD5L	0.991102542	0.851123
HHLA2	1.004091099	0.852158
KLRF2	0.989561371	0.854887
HIST1H3F	0.99149818	0.857796
PADI4	1.009247555	0.866026
PRB3	0.993815907	0.868413
LCN2	0.994830791	0.87099
REG3A	1.009914225	0.871125
PYDC1	0.991831684	0.878775
PAGE1	0.994803849	0.882743
LILRA5	0.9920511	0.884307
ADAM8	1.008986057	0.884704
OLFM4	1.003868205	0.888307
PLK5	1.005815657	0.892194
NR4A3	0.994167517	0.896634
CSF3	1.003836339	0.89752
NKX2-3	1.003790429	0.919896
CYP4F11	1.00211019	0.926266
BPIFA1	1.001521027	0.926791
HIST1H4K	0.995728176	0.935442

For Peer Review

DEFB126	0.996087215	0.937404
SOX11	1.00193251	0.94668
LPO	1.002992841	0.94805
HIST1H4J	0.99648491	0.949279
PROK2	0.997505192	0.958762
HIST1H3I	0.997351159	0.961366
SRMS	0.998193756	0.965942
ANGPT4	0.998693179	0.977659
DUOXA2	0.999258991	0.983108
CCL25	1.000699894	0.98732
FABP4	1.00010909	0.997554

For Peer Review

Table S5. Functional enrichment analysis of the highly expressed genes in the high-risk groups.

Biological process	Genes count	P value	Gene
defense response	15	1.03E-11	KNG1, HIST1H2BE, S100A7, CRP, TAC1, PSG3, IGF2, PYDC1, GAGE1, APOA2, DEFA6, HIST1H2BI, DEFA5, F2, LBP, SCG2
regulation of response to external stimulus	7	1.13E-06	KNG1, F2, TAC1, HRG, IGF2, LBP, SCG2
response to wounding	10	1.39E-06	KNG1, APOA2, FGA, F2, CRP, MSTN, TAC1, IGF2, LBP, SCG2
inflammatory response	8	5.48E-06	KNG1, APOA2, F2, CRP, TAC1, IGF2, LBP, SCG2
positive regulation of multicellular organismal process	7	1.34E-05	F2, TAC1, HRG, IGF2, LBP, PYDC1, NKX2-5
response to organic substance	10	1.72E-05	APOA2, APOB, STAR, MSTN, TAC1, IGF2, CREB3L3, LBP, VGF, NKX2-5
homeostatic process	10	2.38E-05	KNG1, APOA2, APOB, CYP11B1, F2, TAC1, IGF2, COL2A1, VGF, NKX2-3
negative regulation of multicellular organismal process	6	2.75E-05	KNG1, F2, MSTN, TAC1, IGF2, LBP
regulation of system process	7	5.06E-05	APOA2, STAR, MSTN, TAC1, IGF2, VGF, NKX2-5
response to bacterium	6	5.98E-05	HIST1H2BE, S100A7, DEFA6, HIST1H2BI, DEFA5, TAC1, LBP
acute inflammatory response	5	6.14E-05	APOA2, F2, CRP, IGF2, LBP
acute-phase response	4	9.20E-05	F2, CRP, IGF2, LBP
chemical homeostasis	8	1.02E-04	KNG1, APOA2, APOB, CYP11B1, F2, TAC1, IGF2, VGF
defense response to bacterium	5	1.03E-04	HIST1H2BE, S100A7, DEFA6, HIST1H2BI, DEFA5, LBP
response to hormone stimulus	7	1.31E-04	APOA2, STAR, MSTN, TAC1, IGF2, VGF, NKX2-5
response to endogenous stimulus	7	2.24E-04	APOA2, STAR, MSTN, TAC1, IGF2, VGF, NKX2-5
cell activation	6	3.83E-04	CPLX2, FGA, F2, IGF2, LBP, NKX2-3
wound healing	5	7.92E-04	KNG1, FGA, F2, MSTN, IGF2

For Peer Review

regulation of acute inflammatory response	3	9.73E-04	TAC1, IGF2, LBP
steroid metabolic process	5	9.75E-04	APOA2, APOB, STAR, CYP11B1, FSHB
positive regulation of transport	5	0.00140685	F2, TAC1, IGF2, PYDC1, NKX2-5
regulation of transmembrane transporter activity	3	0.00149483	APOA2, IGF2, NKX2-5
regulation of transmembrane transport	3	0.0016122	APOA2, IGF2, NKX2-5
positive regulation of response to stimulus	5	0.00173241	CRP, TAC1, IGF2, LBP, SCG2
regulation of lipid metabolic process	4	0.00190586	APOA2, APOB, STAR, IGF2
regulation of cytokine secretion	3	0.00198979	APOA2, IGF2, PYDC1
regulation of transporter activity	3	0.00240524	APOA2, IGF2, NKX2-5
leukocyte activation during immune response	3	0.00285807	CPLX2, LBP, NKX2-3
regulation of steroid metabolic process	3	0.00285807	APOA2, APOB, STAR
cell activation during immune response	3	0.00285807	CPLX2, LBP, NKX2-3
regulation of blood coagulation	3	0.00285807	KNG1, F2, HRG
macromolecular complex assembly	7	0.00297687	HIST1H3J, HIST1H4L, HIST1H2BE, ANLN, APOA2, APOB, FGA, HIST1H4A, HIST1H4B, HIST1H2BI, HIST1H3A, HIST1H4E, HIST1H3B, HIST1H4F, HIST1H3C, HIST1H4C, HIST1H4D, HIST1H3F, HIST1H3I
immune response	7	0.00357632	CPLX2, S100A7, CYP11B1, CRP, LBP, PYDC1, NKX2-3
regulation of coagulation	3	0.00369454	KNG1, F2, HRG
lipid transport	4	0.00396158	APOA2, APOB, STAR, LBP

For Peer Review

triglyceride metabolic process	3	0.0040573 3	APOA2, APOB, NKX2-3
macromolecular complex subunit organization	7	0.0041179 1	HIST1H3J, HIST1H4L, HIST1H2BE, ANLN, APOA2, APOB, FGA, HIST1H4A, HIST1H4B, HIST1H2BI, HIST1H3A, HIST1H4E, HIST1H3B, HIST1H4F, HIST1H3C, HIST1H4C, HIST1H4D, HIST1H3F, HIST1H3I
regulation of respiratory burst during acute inflammatory response	2	0.0044304 9	IGF2, LBP
regulation of lipid biosynthetic process	3	0.0044360 2	APOB, STAR, IGF2
regulation of hormone levels	4	0.0044379	STAR, CYP11B1, FSHB, VGF
lipid localization	4	0.0049476 8	APOA2, APOB, STAR, LBP
cellular macromolecular complex assembly	5	0.0050685 2	HIST1H3J, HIST1H4L, HIST1H2BE, ANLN, FGA, HIST1H4A, HIST1H4B, HIST1H2BI, HIST1H3A, HIST1H4E, HIST1H3B, HIST1H4F, HIST1H3C, HIST1H4C, HIST1H4D, HIST1H3F, HIST1H3I
glucose homeostasis	3	0.0052405 8	CYP11B1, IGF2, VGF
carbohydrate homeostasis	3	0.0052405 8	CYP11B1, IGF2, VGF
acylglycerol metabolic process	3	0.0052405 8	APOA2, APOB, NKX2-3
neutral lipid metabolic process	3	0.0054514 5	APOA2, APOB, NKX2-3
lipid homeostasis	3	0.0056661 7	APOA2, APOB, IGF2
glycerol ether metabolic process	3	0.0056661 7	APOA2, APOB, NKX2-3
organic ether metabolic process	3	0.0061071 2	APOA2, APOB, NKX2-3
positive regulation of respiratory burst	2	0.0066386 2	IGF2, LBP
regulation of protein secretion	3	0.0072757 9	APOA2, IGF2, PYDC1
regulation of cytokine production	4	0.0073341 5	APOA2, IGF2, LBP, PYDC1
cellular hormone metabolic process	3	0.0075207 6	STAR, CYP11B1, FSHB

For Peer Review

cellular macromolecular complex subunit organization	5	0.00759891	HIST1H3J, HIST1H4L, HIST1H2BE, ANLN, FGA, HIST1H4A, HIST1H4B, HIST1H2BI, HIST1H3A, HIST1H4E, HIST1H3B, HIST1H4F, HIST1H3C, HIST1H4C, HIST1H4D, HIST1H3F, HIST1H3I
ovulation cycle process	3	0.00827782	MSTN, FSHB, VGF
positive regulation of response to external stimulus	3	0.00880084	TAC1, LBP, SCG2
ovulation cycle	3	0.00961251	MSTN, FSHB, VGF
regulation of secretion	4	0.00989866	APOA2, TAC1, IGF2, PYDC1
regeneration	3	0.01017153	APOA2, FGA, MSTN
response to inorganic substance	4	0.01030256	APOB, FGA, STAR, S100A7
blood vessel morphogenesis	4	0.011139	APOB, S100A7, NKX2-5, SCG2
regulation of inflammatory response	3	0.01223883	TAC1, IGF2, LBP
opsonization	2	0.01323462	CRP, LBP
positive regulation of macromolecule biosynthetic process	6	0.01357155	APOA2, F2, MSTN, IGF2, NKX2-5, NKX2-3
positive regulation of cell differentiation	4	0.01388035	APOB, STAR, IGF2, NKX2-5
nucleosome assembly	3	0.01480707	HIST1H3J, HIST1H4L, HIST1H2BE, HIST1H4A, HIST1H4B, HIST1H2BI, HIST1H3A, HIST1H4E, HIST1H3B, HIST1H4F, HIST1H3C, HIST1H4C, HIST1H4D, HIST1H3F, HIST1H3I
steroid biosynthetic process	3	0.01514315	STAR, CYP11B1, FSHB
C21-steroid hormone biosynthetic process	2	0.01542386	CYP11B1, FSHB
regulation of respiratory burst	2	0.01542386	IGF2, LBP
chromatin assembly	3	0.01582517	HIST1H3J, HIST1H4L, HIST1H2BE, HIST1H4A, HIST1H4B, HIST1H2BI, HIST1H3A, HIST1H4E,

For Peer Review

			HIST1H3B, HIST1H4F, HIST1H3C, HIST1H4C, HIST1H4D, HIST1H3F, HIST1H3I
leukocyte activation	4	0.0160798 4	CPLX2, IGF2, LBP, NKX2-3
blood vessel development	4	0.0166138 6	APOB, S100A7, NKX2-5, SCG2
protein-DNA complex assembly	3	0.0172282 3	HIST1H3J, HIST1H4L, HIST1H2BE, HIST1H4A, HIST1H4B, HIST1H2BI, HIST1H3A, HIST1H4E, HIST1H3B, HIST1H4F, HIST1H3C, HIST1H4C, HIST1H4D, HIST1H3F, HIST1H3I
positive regulation of biosynthetic process	6	0.0172741 2	APOA2, F2, MSTN, IGF2, NKX2-5, NKX2-3
vasculature development	4	0.0177118	APOB, S100A7, NKX2-5, SCG2
nucleosome organization	3	0.0179490 3	HIST1H3J, HIST1H4L, HIST1H2BE, HIST1H4A, HIST1H4B, HIST1H2BI, HIST1H3A, HIST1H4E, HIST1H3B, HIST1H4F, HIST1H3C, HIST1H4C, HIST1H4D, HIST1H3F, HIST1H3I
acylglycerol catabolic process	2	0.0197882 7	APOA2, APOB
low-density lipoprotein particle remodeling	2	0.0197882 7	APOA2, APOB
neutral lipid catabolic process	2	0.0197882 7	APOA2, APOB
glycerol ether catabolic process	2	0.0197882 7	APOA2, APOB
positive regulation of locomotion	3	0.0198062 9	TAC1, IGF2, SCG2
response to insulin stimulus	3	0.0205709 8	STAR, IGF2, VGF
regulation of immune effector process	3	0.0209579 4	APOA2, IGF2, LBP
blood coagulation	3	0.0213479 5	KNG1, FGA, F2
coagulation	3	0.0213479 5	KNG1, FGA, F2
multicellular organism reproduction	5	0.0216584 4	APOB, MSTN, TAC1, FSHB, VGF
reproductive process in a	5	0.0216584 4	APOB, MSTN, TAC1, FSHB, VGF

For Peer Review

multicellular organism			
defense response to Gram-negative bacterium	2	0.0219634 6	S100A7, LBP
positive regulation of blood coagulation	2	0.0219634 6	F2, HRG
glucocorticoid metabolic process	2	0.0219634 6	STAR, CYP11B1
response to estrogen stimulus	3	0.0225361 7	APOA2, MSTN, NKX2-5
hormone metabolic process	3	0.0229382 6	STAR, CYP11B1, FSHB
positive regulation of developmental process	4	0.0231491 8	APOB, STAR, IGF2, NKX2-5
hemostasis	3	0.0237513 6	KNG1, FGA, F2
positive regulation of secretion	3	0.0241623 5	TAC1, IGF2, PYDC1
elevation of cytosolic calcium ion concentration	3	0.0245762 8	KNG1, F2, TAC1
regulation of protein transport	3	0.0262611 3	APOA2, IGF2, PYDC1
plasma lipoprotein particle assembly	2	0.0262998 5	APOA2, APOB
C21-steroid hormone metabolic process	2	0.0262998 5	CYP11B1, FSHB
glycerolipid catabolic process	2	0.0262998 5	APOA2, APOB
protein-lipid complex assembly	2	0.0262998 5	APOA2, APOB
positive regulation of acute inflammatory response	2	0.0262998 5	TAC1, LBP
DNA packaging	3	0.0275549 4	HIST1H3J, HIST1H4L, HIST1H2BE, HIST1H4A, HIST1H4B, HIST1H2BI, HIST1H3A, HIST1H4E, HIST1H3B, HIST1H4F, HIST1H3C, HIST1H4C, HIST1H4D, HIST1H3F, HIST1H3I

For Peer Review

cytosolic calcium ion homeostasis	3	0.02799189	KNG1, F2, TAC1
secretion	4	0.02818353	KNG1, CPLX2, VGF, SCG2
defense response to fungus	2	0.02846106	DEFA6, DEFA5
negative regulation of lipid catabolic process	2	0.02846106	APOA2, IGF2
positive regulation of coagulation	2	0.02846106	F2, HRG
regulation of establishment of protein localization	3	0.02931956	APOA2, IGF2, PYDC1
regulation of apoptosis	6	0.03027312	KNG1, F2, IGF2, COL2A1, NKX2-5, SCG2
killing of cells of another organism	2	0.03061764	DEFA6, DEFA5
regulation of programmed cell death	6	0.03142049	KNG1, F2, IGF2, COL2A1, NKX2-5, SCG2
regulation of cell death	6	0.03185786	KNG1, F2, IGF2, COL2A1, NKX2-5, SCG2
chromatin assembly or disassembly	3	0.03204942	HIST1H3J, HIST1H4L, HIST1H2BE, HIST1H4A, HIST1H4B, HIST1H2BI, HIST1H3A, HIST1H4E, HIST1H3B, HIST1H4F, HIST1H3C, HIST1H4C, HIST1H4D, HIST1H3F, HIST1H3I
rhythmic process	3	0.03251389	MSTN, FSHB, VGF
copulation	2	0.03276959	TAC1, VGF
regulation of interleukin-8 production	2	0.03276959	APOA2, LBP
response to metal ion	3	0.03298105	APOB, FGA, STAR
lipid biosynthetic process	4	0.03402616	APOA2, STAR, CYP11B1, FSHB
lipoprotein particle clearance	2	0.03491693	APOA2, APOB
negative regulation of protein secretion	2	0.03491693	APOA2, IGF2
immune effector process	3	0.03535656	CPLX2, CRP, LBP

For Peer Review

regulation of synaptic transmission	3	0.03632505	STAR, TAC1, VGF
innate immune response	3	0.03730385	S100A7, LBP, PYDC1
regulation of protein localization	3	0.03730385	APOA2, IGF2, PYDC1
positive regulation of macromolecule metabolic process	6	0.03839368	APOA2, F2, MSTN, IGF2, NKX2-5, NKX2-3
regulation of body fluid levels	3	0.03879111	KNG1, FGA, F2
myeloid cell activation during immune response	2	0.03919778	CPLX2, LBP
alpha-beta T cell activation	2	0.03919778	IGF2, NKX2-3
spleen development	2	0.04133131	NKX2-5, NKX2-3
regulation of transmission of nerve impulse	3	0.04183305	STAR, TAC1, VGF
negative regulation of apoptosis	4	0.04282917	IGF2, COL2A1, NKX2-5, SCG2
plasma lipoprotein particle remodeling	2	0.04346027	APOA2, APOB
macromolecular complex remodeling	2	0.04346027	APOA2, APOB
protein-lipid complex remodeling	2	0.04346027	APOA2, APOB
negative regulation of programmed cell death	4	0.04434757	IGF2, COL2A1, NKX2-5, SCG2
negative regulation of cell death	4	0.0446545	IGF2, COL2A1, NKX2-5, SCG2
regulation of neurological system process	3	0.04496261	STAR, TAC1, VGF
response to peptide hormone stimulus	3	0.04549253	STAR, IGF2, VGF
regulation of lipid storage	2	0.04558465	APOB, CRP

For Peer Review

glycerolipid metabolic process	3	0.04981537	APOA2, APOB, NKX2-3
negative regulation of blood coagulation	2	0.04981975	KNG1, F2
positive regulation of cytokine secretion	2	0.04981975	IGF2, PYDC1

For Peer Review

Table S6. Pathway enrichment analysis of the highly expressed genes in the high-risk groups.

Pathway	Genes count	P value	Genes
Alcoholism	16	3.35E-26	HIST1H4F, CREB3L3, HIST1H4C, HIST1H4E, HIST1H4D, HIST1H4L, HIST1H4B, HIST1H3B, HIST1H4A, HIST1H3F, HIST1H3A, HIST1H3C, HIST1H3I, HIST1H2BI, HIST1H3J, HIST1H2BE
Systemic lupus erythematosus	15	7.42E-26	HIST1H4F, HIST1H4D, HIST1H4C, HIST1H4E, HIST1H4B, HIST1H4L, HIST1H3B, HIST1H4A, HIST1H3F, HIST1H3A, HIST1H3C, HIST1H3I, HIST1H2BI, HIST1H3J, HIST1H2BE
Viral carcinogenesis	10	4.63E-14	HIST1H4F, HIST1H4D, HIST1H4C, HIST1H4E, CREB3L3, HIST1H4L, HIST1H4B, HIST1H4A, HIST1H2BI, HIST1H2BE
Transcriptional misregulation in cancer	6	6.72E-08	HIST1H3B, HIST1H3F, HIST1H3A, HIST1H3C, HIST1H3I, HIST1H3J
Complement and coagulation cascades	3	0.000112321	KNG1, F2, FGA
Ovarian steroidogenesis	2	0.001598553	STAR, FSHB
Aldosterone synthesis and secretion	2	0.004012277	CREB3L3, STAR
cAMP signaling pathway	2	0.021786021	CREB3L3, FSHB
Vitamin digestion and absorption	1	0.027890963	APOB
Neuroactive ligand-receptor interaction	2	0.040033981	F2, FSHB
Fat digestion and absorption	1	0.046420833	APOB
Vasopressin-regulated water reabsorption	1	0.049654764	CREB3L3

For Peer Review

Table S7. Functional enrichment analysis of the highly expressed genes in the low-risk groups.

Biological process	Genes count	P value	Gene
immune response	6	3.61E-05	C8A, SUSD2, CEACAM8, CD300LG, PLA2G1B, SFTPD
positive regulation of response to stimulus	4	4.07E-04	C8A, NPY, INS, PLA2G1B
negative regulation of immune system process	3	0.001302	INS, SFTPD, SCGB1A1
regulation of response to external stimulus	3	0.004681	NPY, INS, SCGB1A1
regulation of cytokine production	3	0.006025	INS, SFTPD, SCGB1A1
positive regulation of transport	3	0.009023	INS, PLA2G1B, SFTPD
regulation of cell proliferation	4	0.012648	INS, PLA2G1B, SFTPD, SCGB1A1
negative regulation of T cell proliferation	2	0.017825	SFTPD, SCGB1A1
positive regulation of DNA replication	2	0.01848	INS, PLA2G1B
glucose transport	2	0.019134	INS, PLA2G1B
hexose transport	2	0.019788	INS, PLA2G1B
monosaccharide transport	2	0.020442	INS, PLA2G1B
negative regulation of leukocyte proliferation	2	0.021095	SFTPD, SCGB1A1
negative regulation of lymphocyte proliferation	2	0.021095	SFTPD, SCGB1A1
negative regulation of mononuclear cell proliferation	2	0.021095	SFTPD, SCGB1A1
leukocyte chemotaxis	2	0.024355	PLA2G1B, SFTPD
cell chemotaxis	2	0.025656	PLA2G1B, SFTPD
positive regulation of protein secretion	2	0.026956	INS, PLA2G1B

For Peer Review

negative regulation of T cell activation	2	0.027606	SFTPD, SCGB1A1
negative regulation of lymphocyte activation	2	0.035367	SFTPD, SCGB1A1
positive regulation of DNA metabolic process	2	0.036656	INS, PLA2G1B
behavior	3	0.036738	NPY, PLA2G1B, SFTPD
negative regulation of leukocyte activation	2	0.037299	SFTPD, SCGB1A1
leukocyte migration	2	0.037299	PLA2G1B, SFTPD
cell motion	3	0.037607	NPY, PLA2G1B, SFTPD
regulation of protein secretion	2	0.037942	INS, PLA2G1B
carbohydrate transport	2	0.03987	INS, PLA2G1B
negative regulation of cell activation	2	0.03987	SFTPD, SCGB1A1
regulation of DNA replication	2	0.040511	INS, PLA2G1B
regulation of T cell proliferation	2	0.040511	SFTPD, SCGB1A1
positive regulation of protein transport	2	0.043714	INS, PLA2G1B
cellular response to insulin stimulus	2	0.044353	INS, PLA2G1B
regulation of inflammatory response	2	0.049455	INS, SCGB1A1

For Peer Review

Table S8. Pathway enrichment analysis of the highly expressed genes in the low-risk groups.

Pathway	Genes count	P value	Gene
Regulation of lipolysis in adipocytes	2	0.000114	NPY/INS
Prostate cancer	2	0.000281	KLK3/INS
Ras signaling pathway	2	0.00177	PLA2G1B/INS
alpha-Linolenic acid metabolism	1	0.007169	PLA2G1B
Maturity onset diabetes of the young	1	0.007443	INS
Linoleic acid metabolism	1	0.008267	PLA2G1B
Prion diseases	1	0.009913	C8A
Aldosterone-regulated sodium reabsorption	1	0.011009	INS
Regulation of autophagy	1	0.011283	INS
Fat digestion and absorption	1	0.011557	PLA2G1B
Type I diabetes mellitus	1	0.012378	INS
Ether lipid metabolism	1	0.012651	PLA2G1B
Type II diabetes mellitus	1	0.013471	INS
Ovarian steroidogenesis	1	0.014017	INS
Arachidonic acid metabolism	1	0.01729	PLA2G1B
Longevity regulating pathway - multiple species	1	0.017834	INS
Adipocytokine signaling pathway	1	0.019466	NPY
Prolactin signaling pathway	1	0.020009	INS
Complement and coagulation cascades	1	0.021908	C8A
Insulin secretion	1	0.023534	INS
Longevity regulating pathway	1	0.025967	INS
Glycerophospholipid metabolism	1	0.026237	PLA2G1B
Pancreatic secretion	1	0.026507	PLA2G1B

For Peer Review

Progesterone-mediated oocyte maturation	1	0.027047	INS
Amoebiasis	1	0.027586	C8A
HIF-1 signaling pathway	1	0.028395	INS
Insulin resistance	1	0.030011	INS
Vascular smooth muscle contraction	1	0.032966	PLA2G1B
Oocyte meiosis	1	0.033771	INS
AMPK signaling pathway	1	0.034307	INS
FoxO signaling pathway	1	0.036716	INS
Systemic lupus erythematosus	1	0.037251	C8A
Insulin signaling pathway	1	0.038052	INS
Phospholipase D signaling pathway	1	0.039386	INS
Non-alcoholic fatty liver disease (NAFLD)	1	0.041252	INS
mTOR signaling pathway	1	0.04205	INS
Phagosome	1	0.042316	SFTPD
cGMP-PKG signaling pathway	1	0.045503	INS
Alcoholism	1	0.04868	NPY

For Peer Review
